# Adaptation strategies for preparing for childbirth in the context of the pandemic: Roy’s Theory

**DOI:** 10.1590/0034-7167-2023-0159

**Published:** 2024-07-29

**Authors:** Letícia Pickler, Margarete Maria de Lima, Ariane Thaise Frello Roque, Laís Antunes Wilhelm, Felice Curcio, Dionara Guarda, Roberta Costa, Isadora Ferrante Boscoli de Oliveira Alves

**Affiliations:** I Universidade Federal de Santa Catarina. Florianópolis, Santa Catarina, Brazil; II Universidade de Estudos de Sássari. Sássari, Sardenha, Italy

**Keywords:** Pregnancy, Health Education, Parturition, Pandemic, Adaptation Strategy, Embarazo, Educación en Salud, Parto, Pandemia, Estrategia de Adaptación

## Abstract

**Objectives:**

to understand the process of adapting to childbirth during the COVID-19 pandemic from the perspective of a group of pregnant women.

**Methods:**

a qualitative, descriptive-exploratory study was conducted with 23 women. Data were collected between October and December 2021 through documentation and semi-structured interviews, which were analyzed using Minayo’s methodology and Roy’s Adaptation Model.

**Results:**

various types of stimuli - focal, contextual, and residual - were identified as influencing childbirth preparation. The online group was essential for facilitating pregnant women’s adaptation, offering significant support and generating positive feedback for childbirth preparation.

**Final Considerations:**

the importance of pregnant women’s groups as a strategy for improving adaptation to childbirth was identified, underscoring the effectiveness of this support among professionals and participants, as well as among pregnant women. This support network strengthened preparation for childbirth during a challenging period like the pandemic.

## RESUMEN

**Objetivos:**

conocer el proceso de adaptación al parto durante la pandemia de COVID-19 desde la perspectiva de un grupo de gestantes.

**Métodos:**

se llevó a cabo un estudio cualitativo, descriptivo-exploratorio con 23 mujeres. Los datos se recopilaron entre octubre y diciembre de 2021 mediante documentación y entrevistas semiestructuradas, analizados según la metodología de Minayo y el Modelo de Adaptación de Roy.

**Resultados:**

se identificaron varios tipos de estímulos -focales, contextuales y residuales- que influyeron en la preparación para el parto. El grupo en línea resultó esencial para facilitar la adaptación de las gestantes, ofreciendo un apoyo significativo y generando una retroalimentación positiva para la preparación para el parto.

**Consideraciones Finales:**

se identificó la importancia de los grupos de gestantes como estrategia para mejorar la adaptación al parto, destacando la eficacia de este apoyo tanto entre profesionales y participantes como entre las gestantes, fortaleciendo la red de apoyo y la preparación para el parto durante un período tan desafiante como una pandemia.

## INTRODUCTION

The pandemic caused by the 2019 coronavirus has become one of the greatest challenges of the 21st century, leading to a sudden increase in infections and deaths. Pregnant women were included in the high-risk group because they are more vulnerable to unfavorable outcomes due to physiological changes during pregnancy. As a result, new care protocols were implemented to reduce their exposure and ensure the continuity of prenatal care^(1-2)^.

Pregnancy is a period marked by intense changes and is permeated by a range of emotions. In a pandemic context, significant changes in the social structure lead to a sudden disruption of expectations, particularly concerning childbirth^(3)^.

Childbirth, which has accumulated sociocultural significance over the years, is often associated with pain and suffering for many women^(4)^. However, thanks to public policies and various social movements, Brazil is experiencing a growing trend to restore women’s autonomy and agency during childbirth. This shift aims to establish a new model of childbirth care in the country^(5)^.

Much of the fear, insecurity, and anxiety related to childbirth is linked to a lack of information during prenatal care^(4,6)^. Women who receive quality guidance and continuous support from pregnancy onward experience childbirth with greater confidence and awareness^(7)^. However, the pandemic has exacerbated fears and anxieties among pregnant women, particularly regarding childbirth. This makes it essential for them to seek adaptation mechanisms to ensure a positive experience of childbirth and delivery^(8-9)^.

In her Adaptation Theory, Callista Roy considers that an individual is an adaptable, holistic system that activates coping mechanisms in response to stimuli to generate adaptive or inefficient responses, which can serve as system reentry^(8)^. The system is defined as a set of interconnected parts functioning holistically through interdependence. “Holistic” refers to the system as a whole, not just the sum of its parts. “Adaptable” means the ability to adjust to environmental changes and, in turn, influence the environment. Systems have inputs, processes, behaviors, and outputs. Thus, perceiving an individual as an adaptive system encompasses four elements: stimuli, coping mechanisms, adaptive modes, and responses^(8,10)^.

In this framework, Roy’s theory helps understand how women faced the dual challenges of pregnancy and childbirth, especially in the pandemic’s first year, and how they adapted by adjusting to new changes. Expanding knowledge on this topic is crucial to recognize the contribution of educational activities during pregnancy and the importance of maintaining them during epidemiological crises. Highlighting pregnant women’s groups as a means of socialization and support encourages new health education strategies aimed at preparing for childbirth.

## OBJECTIVES

To understand the process of adapting to childbirth during the COVID-19 pandemic from the perspective of a group of pregnant women.

## METHODS

### Ethical Considerations

The study was approved by the Research Ethics Committee on Human Subjects of a Federal University in Southern Brazil. Participants signed the Free and Informed Consent Form (TCLE). The ethical aspects of research involving human beings were respected, specifically the resolutions 466/2012 and 510/2016, as well as Official Circular No. 2/2021/CONEP/SECNS/MS, which provides guidelines for research procedures in a virtual environment^(11-13)^.

### Type of Study

This is a descriptive and exploratory study using a qualitative approach. The Consolidated Criteria for Reporting Qualitative Research (COREQ) was used to guide methodological rigor.

### Study Setting

This study was conducted within a health education extension project for pregnant women and couples, affiliated with a Federal University and a University Hospital in Southern Brazil. The project aims to share experiences about the pregnancy and postpartum cycle, disseminate information based on scientific evidence, promote a support network, and prepare for childbirth. The multidisciplinary team consists of faculty, nurses, a psychologist, and nursing and psychology students. During the pandemic, activities were carried out virtually to comply with disease prevention measures. Women are typically grouped based on similarities in gestational age; these groups are numbered, and to date, 107 groups have been formed. Of these, 10 were conducted virtually, involving a total of 304 women and 281 companions.

Maternal-fetal care topics were discussed over seven meetings, in addition to participant monitoring by the team through a WhatsApp^®^ group, which allowed for increased interaction among participants. They also receive educational materials through a messaging group to facilitate interaction among families.

### Data Source

Women from the 96th and 97th virtual groups for pregnant women and couples, held from March to June 2020, were invited to participate in this study. After reaching out to 45 women, 23 agreed to participate, while 22 did not respond.

Inclusion criteria: Women over 18 years old who had their babies between March and December 2020, the first year of the pandemic.

Exclusion criteria: Women not listed in the WhatsApp^®^ contact lists of participants in the 96th and 97th groups.

### Data Collection and Organization

Data were collected using two techniques: document analysis and semi-structured interviews, conducted between October and December 2021. The women were contacted via WhatsApp^®^, where the research topic, objectives, and direction were explained. After accepting the invitation, their demographic and obstetric data were accessed from registration forms through the Pregnant Women’s Group database to characterize participant profiles.

Interviews were conducted via Google Meet^®^ and WhatsApp^®^, scheduled according to the participants’ preferred dates and times, and lasting around 30 minutes each. They were led by the primary author, an undergraduate nursing student at the time, who had been trained in data collection techniques and was already connected to the participants through her extension project activities. Most women were accompanied by their children, and privacy concerns did not interfere with the responses. Participants were informed that they could request to stop the interview at any time.

An open-ended question guide was used to explore the women’s perceptions and experiences regarding childbirth preparation and participation in the pregnant women’s group. Audio recordings were utilized to capture the data. None of the participants expressed discomfort during the interviews. The questions were not shared in advance, and no repeat interviews were conducted.

Data saturation occurred at the 21st interview, but all 23 women who agreed to participate were interviewed. The audio recordings were fully transcribed by the primary author and stored in documents labeled with participants’ names, while identifying information was stored in a spreadsheet showing absolute and relative frequencies as percentages.

### Data Analysis

The analytical procedure for this study was based on Minayo’s approach, adapted to the aims of this investigation. Data analysis included the following stages: pre-analysis, material exploration, results processing, and interpretation^(14)^.

During the pre-analysis stage, the transcribed interviews were thoroughly read to establish the initial stages of the interpretive process. Relevant excerpts were selected to provide a direction for understanding. Callista Roy’s Adaptation Theory served as the theoretical framework guiding the data analysis, positioning nursing as a facilitator in an individual’s adaptation process^(8)^.

In the material exploration stage, analytical categories were defined after thorough reading, based on the theoretical framework. Interview excerpts were coded with the letter “E” for interviewees, followed by an ordinal number (1 to 23) corresponding to the order of the interviews. In the results processing stage, the data were fully reviewed to identify patterns and inferences, continuing the analytical process in correlation with the theoretical framework.

After carefully analyzing the interviews, the data were grouped into three categories guided by Roy’s theoretical framework: “Focal, Contextual, and Residual Stimuli in Childbirth Preparation”; “Adaptive Modes: Pregnant Women’s Group as a Facilitator of the Adaptation Process”; and “Positive Feedback for Childbirth Preparation”.

## RESULTS

The women’s ages ranged from 19 to 39 years, with 4% aged 19 to 25, 35% aged 26 to 32, and 61% aged 33 to 39, giving an average age of 32.9. Regarding marital status, 22% were single, 61% were married, and 17% did not respond to this question.

In terms of pregnancy data, 78.3% were pregnant for the first time, 8.7% were in their second pregnancy, 8.7% were in their third pregnancy, and 4.3% did not answer this question. Regarding the number of deliveries, 91.3% of the women were nulliparous, 4.3% had one delivery, and 4.3% had two deliveries. As for pregnancy planning, 74% of the participants had planned their pregnancies. All the women reported receiving prenatal care, with 39% in the public network, 57% in the private network, and 4% in both networks.

### Focal, Contextual, and Residual Stimuli in Childbirth Preparation

This category discusses the focal, contextual, and residual stimuli characterized by Roy that influenced the preparation process for childbirth in these women. The participants reported that the pandemic context significantly impacted the environment, causing stress and insecurity.

*The pandemic had a negative effect on my pregnancy because I was doing well, healthy, under control, well monitored, and then this change, this significant stress, all this shifting scenario, and the insecurity it caused, I believe amplified my discomfort with my blood pressure* [...]. (E3)

Even though they used tools to maintain contact, physical restrictions were a challenge for these women, who had to navigate this period without the idealized presence of their support network.

[...] *it was truly a unique time because it’s like having a pregnancy and keeping it to yourself, that’s the feeling I have.* [...] *Even with video calls and photos, we couldn’t spend time together* [...] *it was very limited* [...]. (E6)

In addition to distancing from their support network, uncertainty about the presence of a companion during childbirth was also considered a new source of concern.

*My only worry was having to give birth alone; that scared me because I felt the support and help from my husband would be crucial to achieving the kind of delivery I wanted* [...]. (E17)

Childbirth holds different meanings for each woman; in some interviews, the fear was linked to previous negative experiences or the fact that it was an unfamiliar experience.

[...] *we read everything here at home because I was going to be a first-time mother, so naturally, I was facing many fears.* (E16)

*I didn’t have a good experience with my second childbirth; it was a bit difficult* [...] *and I always had significant fear of the end, of the childbirth part, which always made me anxious* [...]. (E18)

Fear of contracting the virus, uncertainty about the disease, and insecurity in seeking professional help were considered limiting factors in childbirth preparation.

*The pandemic affected this a lot because even in choosing a professional, you feel reluctant to keep looking around* [...]*. And even for perineal preparation* [...] *I did some, but then eventually I stopped because I was too scared* [...]. (E21)

[...] *when the pandemic started, the panic of something happening to me or my baby outweighed everything* [...] *so my preparation was very limited and full of fear* [...]. (E13)

### Adaptive Modes: Pregnant Women’s Group as a Facilitator of the Adaptation Process

This category addresses the four adaptive modes (physiological, self-concept, role performance, and interdependence) described by Roy as observable behaviors in the adaptation process. The physiological mode refers to how a person responds physically to environmental stimuli. Learning about childbirth physiology, relaxation exercises, comfortable positions, and pain management strategies were positive approaches for the women.

[...] *that material on pelvic massage* [...] *was perfect; I hadn’t been able to find a pelvic physiotherapist, so I taught myself by searching for videos. The material was clear.* (E15)

[...] *doing physical exercises throughout the pregnancy until the end and working my legs, arms, and pelvic muscles helped a lot. Every push made the baby descend easily. It didn’t take an hour for this part* [expulsive phase]. (E9)

The self-concept mode encompasses the psychosocial aspects of the individual through the physical self (body image) and personal self (ideal self, self-consistency, and moral-ethical-spiritual self) . Some women found the changes they faced during this period to be frightening. They also wanted to share their bodily changes and expectations for pregnancy.

[...] *dealing with the hormones, changes, I didn’t recognize myself; I didn’t know what was happening to my body. That was terrifying, and that part was horrible. Even though I knew I’d go through it, it was still unknown* [...] *I think the emotional preparation made the most difference for me.* (E19)

[...] *I had a different vision of what pregnancy would be like, things I wanted to do, like dancing with my big belly, but none of that happened* [...]. (E11)

The interdependence mode involves interpersonal relationships and emotional needs. In this regard, support from family, partners, friends, and other systems is essential for well-being.

[...] *I never felt so strongly that I wanted to be near my family, to have my mother next to me, my aunts, all these women. That’s why the group was so important, having these women by my side* [...]. (E12)

[...] *The group was crucial for us to maintain that exchange of experiences.* [...] *I wasn’t seeing anyone in person* [...]*. So we formed a strong bond there* [...] *because each of us knew the others were going through the same process* [...]. (E1)

[...] *I get emotional just thinking about it because it was a way to have some contact, something to pull us out of that moment of uncertainty from the pandemic. It was calming; it brought serenity and quality information, so it was a real anchor.* (E14)

Roy sees role performance as the process of recognizing oneself in relation to others. As such, people perform primary, secondary, or tertiary roles. Since planning their pregnancies, the women showed attitudes to solidify their maternal role, emphasizing the importance of partners participating in the learning process.

[...] *I did all the physical, mental, and psychological preparation I could, preparing myself for motherhood. My pregnancy was completely planned* [...]. (E8)

[...] *When we got the positive test, we started studying and thinking about the different birth possibilities* [...] *without information, everything would have been much harder* [...]. (E10)

[...] *various rights we have, even in the delivery room* [...] *to ensure my rights are protected.* (E4)

[...] *His participation in all the meetings was very important.* [...] *He didn’t attend ultrasounds or consultations to hear the fetal heartbeat; it was all done through video calls* [...]. (E5)

### Positive Feedback on Childbirth Preparation

This category encompasses the women’s perspectives on adaptive responses in preparation for childbirth. According to Roy’s Adaptation Model, adaptive responses are those that promote personal integrity.

In their interviews, the women reported that, despite experiencing pregnancy and childbirth during the pandemic, they sought to harness their potential as women, recognizing childbirth preparation as a crucial step.

[...] *There are many feelings, and understanding at least the process helps.* (E7)

[...] *You need to understand the transformation you’re going through. It’s not just physical; it’s also emotional and financial* [...] *which is why I believe information is fundamental* [...]. (E16)

The women considered information and emotional preparation essential to facing this process and felt more familiar with childbirth after accessing information in advance.

[...] *Preparing for childbirth is preparing emotionally* [...] *the first step for mothering is being prepared for childbirth.* (E22)

[...] *It was important that I was already familiar with everything. They had already told me how things would be, that the nurse would come in at certain intervals. I already knew the room, I knew what to expect* [...]. (E2)

When a changing environment stimulates someone to create adaptable responses, positive feedback is provided to the system, allowing them to decide whether to increase or decrease their efforts to handle the stimuli.

[...] *I wouldn’t read too much because I’m anxious, and I thought that might worsen my situation.* [...] *Now, I would do things completely differently* [...] *I would do everything differently. I would talk about childbirth, plan these things* [...]. (E23)

After going through this process, the women believed childbirth could be a positive and enjoyable experience when they feel prepared and supported.

[...] *Vaginal childbirth can be enjoyable* [...]. *Out of my entire maternity experience, the best thing was childbirth. I have such fond memories* [...]. (E19)

[...] *You need someone’s support to remind you that even in the most desperate moment, you’ll manage* [...] *it’s like a wave that comes intensely but then passes. Knowing this and having that preparation beforehand is crucial* [...]*; it’s not instinctive* [...]. (E20)

## DISCUSSION

COVID-19 has altered the organization of maternal care services, creating challenges for prenatal, childbirth, and postpartum care^(15)^. The pandemic subjected pregnant and postpartum women to restrictions and stressors, driving a need for adaptation. All people are adaptable, responding to both internal and external stimuli and using coping mechanisms to handle environmental changes. Thus, the interaction between this constantly changing environment and individuals or communities prompted adaptive responses^(1,8,15)^.

Pregnancy is a period of intense transformation that triggers a range of feelings, challenges, and internal conflicts. However, women who experienced pregnancy and childbirth in the pandemic’s first year were affected not only by pregnancy but also by external influences that interfered with their adaptation process^(1,16)^. According to the reports, the pandemic and pregnancy emerged as focal stimuli due to their significant impact.

Contextual stimuli can affect women’s behavior, their responses to challenges, and their ability to adapt to their environment. From this perspective, women’s behavior can influence their self-esteem and well-being while also transforming their surroundings^(8,10,16)^. The physiological changes inherent to pregnancy, combined with preventive measures against COVID-19, added extra stress, interfering with the adaptation process^(8,17)^. Thus, pregnancy during the pandemic heightened fears, uncertainties, insecurities, stress, and anxiety^(18)^.

In many cultures, specific beliefs exist about childbirth because it is imbued with meanings passed down through generations^(19)^. Roy identifies these aspects as residual stimuli, containing unique features in people whose individual impacts are unclear^(8)^. Therefore, previous experiences or unfamiliar situations are significant when facing childbirth.

Since the same stimulus can trigger different behaviors in individuals based on their level of adaptation, Roy’s theory recognizes that those facing challenges may sometimes have adaptive responses and other times inefficient ones^(20)^. However, the initial environmental stimuli women received heightened negative responses. As a result, they increased their efforts in childbirth preparation as an adaptive mechanism.

A person’s coping ability is proportional to their level of adaptation and stage in life. Some coping mechanisms are inherited genetically, while others are learned. They are expressed through behaviors characterized as adaptive modes, divided into four categories: physiological, self-concept, role performance, and interdependence^(8,21)^.

Women who receive continuous support during the perinatal period are more likely to experience positive psychological effects and, consequently, adaptive responses^(22)^. During prenatal care, pregnant women receive guidance on pregnancy and childbirth. Taking into account their values and desires, they develop preferences and make informed decisions about which obstetric practices to follow or avoid during delivery. This health education process not only strengthens women’s sense of security but also promotes autonomy, empowering them to play a central role in childbirth and exercise informed decision-making^(23)^. Thus, in a landscape full of uncertainties, the pregnant women’s group emerges as an essential facilitator in the adaptation mechanism, creating a support network that significantly enhances women’s emotional strength and confidence during prenatal care.

Increased awareness of the perineum and pelvic floor strengthening techniques during pregnancy are strategies for protecting the integrity of this area and enhancing understanding of the birthing process^(24)^. In the physiological mode, therapeutic practices used during pregnancy and childbirth were linked to increased comfort and autonomy for women during labor.

Pregnancy involves restructuring and readjustment across various dimensions, changes in identity and social roles, and new levels of integration and personality maturity. All these changes affect both partners, particularly the pregnant woman, and are intensified by physiological changes, demonstrating the importance of childbirth preparation as a stimulus for producing behaviors that facilitate parental roles^(24-25)^.

Changes in women’s self-concept help prepare them for motherhood and new roles. Thus, recognizing the bodily changes inherent in pregnancy progression involves body image and how women see themselves physically, which is defined as one of the components of self-concept described by Roy^(8,26)^.

Pregnancy is a physiological process that is planned and desired by many women, during which expectations are formed. The pandemic and subsequent social distancing brought significant behavioral changes^(3,16)^. Among this group of women, a feeling of frustration emerged, particularly due to the unrealized desire to share their growing bellies with their social circle or to engage in activities previously planned for this period. Family support, especially from partners and the pregnant woman’s parents, is a crucial pillar of maternal support. A strong support network is essential for effective adaptation^(25)^.

The opportunity to share experiences, be heard, clarify doubts, and express feelings, even in a physically restrictive online environment, helps pregnant women feel more supported and confident about childbirth^(27-28)^. In this context, the reciprocity of the interpersonal relationships formed in the pregnant women’s group, characterized in the interdependence mode as a support system, was fundamental to these women’s adaptive process.

Pregnancy is understood to be a biopsychosocial phenomenon that brings changes to women’s lives^(29)^. Thus, adapting to the maternal role can be challenging, especially when there is a lack of information and support^(30)^. In this direction, the pregnant women’s group was defined as a reassuring space for these concerns through the dissemination of reliable information, making it easier to adjust to motherhood.

The effective participation of a companion during pregnancy not only demonstrates support and strengthens role performance but also impacts women’s satisfaction with the support received during labor and their confidence in following through on planned actions^(31)^. Thus, the involvement of companions with the pregnant women’s group was positively perceived by women, contributing to the creation of a healthy and respectful childbirth experience.

The process of preparing for childbirth involves incorporating a range of care, measures, and activities that enable women to experience labor and childbirth as a physiological event, giving them ownership of this moment^(24,32)^. Prenatal care, through preventive measures and health education initiatives, aims to ensure a healthy pregnancy and facilitate the birth of a healthy baby, preserving maternal and infant well-being^(33)^.

With the right information and guidance, this period can be approached with greater security and peace of mind. On the other hand, a lack of information can compromise active participation and distort women’s perception of childbirth^(34)^. In this sense, women who found their preparation insufficient used this experience as a lesson and, based on that perception, felt more inclined to participate actively in their health care.

Callista Roy emphasizes that maximizing the use of coping mechanisms enhances one’s adaptation level and increases their ability to express adaptive responses to stimuli. In this sense, the adaptive responses developed in childbirth preparation became positive feedback to the system, boosting individual adaptation levels and expanding the range of stimuli to which they can respond positively with minimal effort^(8,10)^.

### Study limitations

A limiting aspect of this study is the nature and scope of the sample used. Data collection was restricted to a specific group of pregnant women from one region, which may limit the generalizability of the results to a broader, more diverse population. Additionally, voluntary participation may have introduced selection bias, as women who felt more engaged with or benefited from the project might have been more inclined to participate. Future studies should explore paternal perspectives and those of healthcare professionals involved in this adaptation process. This integration has the potential to improve understanding of the phenomenon and deepen findings on this topic.

### Contributions to the Field of Nursing and Health

This study offers valuable contributions to the field of nursing and health, emphasizing the importance of keeping women well-informed so they can actively participate in their childbirth process. The significance of involving pregnant women in activities that promote adaptive responses during pregnancy, especially those of an educational and collective nature, is highlighted. These activities not only enhance women’s knowledge and autonomy but also encourage the adoption of healthy and proactive maternity practices.

Furthermore, this study’s findings are expected to raise awareness among healthcare professionals, particularly nurses, of the importance of creating spaces that encourage interaction and learning among families. Such spaces are essential to help families adapt to the childbirth and postpartum environment, promoting a more positive and integrated experience. Through these initiatives, support and guidance for pregnant women can be significantly improved, benefiting the health and well-being of both mothers and newborns.

## FINAL CONSIDERATIONS

Our study emphasizes that Roy’s proposed adaptive modes are interconnected: learning, perception, information processing, judgment, and emotions were all integral to the adaptation process for childbirth preparation. This underscores the importance of an environment that fully encourages the use of coping mechanisms.

The pregnant women’s group was characterized as an environment capable of promoting adaptive responses, particularly by building an effective support system not only among professionals and participants but also among the women themselves. Thus, participation in educational activities starting during pregnancy is crucial for enhancing satisfaction in the childbirth process.

## Supplementary Material











## Data Availability

https://doi.org/10.48331/scielodata.Q5KQCH
