# Supplementary material for: Adaptation strategies for preparing for childbirth in the context of the pandemic: Roy’s Theory
Source: Rev Bras Enferm. 2024 Jul 29;77(3):e20230159. doi: 10.1590/0034-7167-2023-0159 (PMC11290742; doi:10.1590/0034-7167-2023-0159)
Supplement: Supplementary file 1 [file 0034-7167-reben-77-03-e20230159-suppl01.pdf]

ENTREVISTA 01

Data da entrevista: 25/10/2021
DATA DO PARTO: 13/06/2020

Entrevistadora: Como foi sua experiência de preparação para o parto no contexto de pandemia?

E1: Então, eu tava com seis meses de gravidez né, e aí a gente foi pego de surpresa pela pandemia. Em março, eu tinha acabado de começar a fisioterapia pélvica e tava começando a me preparar para o parto normal mas, sempre com a clareza assim, eu tinha medo da dor, das possíveis intervenções que poderiam acontecer, mas eu queria muito esperar o tempo do meu bebê né?! Entrar em trabalho de parto, esperar um dia dela chegar… isso era muito claro pra mim, não importava como ela viesse, eu não fazia questão, não "tinha que ser parto normal", não! E eu não queria optar pela eletiva, agendada assim sabe. Eu queria entrar em trabalho de parto, deixar a coisa acontecer, eu sempre falava isso "eu quero deixar acontecer". E aí em março a gente entrou na pandemia e foi muito difícil, tanto pra terminar a preparação para o parto, porque eu não consegui terminar a fisioterapia porque a gente (perda de conexão). E aí, não e aí assim, os médicos toda equipe de apoio tava começando a aprender a lidar com as gestantes na pandemia, agora ja ta mais ou menos todo mundo acostumado, os médicos já atendem online e o pessoal ja ta vacinado. E aí a questão do emocional pegou muito ali na primeira fase, ali em março abril que a gente não sabia como é que ia acontecer eu não sabia se eu ia continuar tendo atendimento da obstetra, quando foi chegando perto as maternidades fechando, sem poder entrar acompanhante, acho que em questão de dias assim teve um monte de rumor assim "Ah, lá na Itália as mães tão parindo sozinhas, na Holanda…" eu me lembro assim, bem disso, dos outros ter que ganhar o bebê sozinho, aquilo me apavorava assim, eu entrei numa crise ansiedade bem forte nessa fase e aí eu ganhei ela em junho, então foi um processo, até acostumar, ali em março, abril, às clínicas juntarem, acostumar fazer o ultrassom do bebê sem olhando, sem o pai acompanhando, sem poder visitar minha família. Todo mundo deve relatar isso assim, dessa parte do apoio assim, foi muito difícil, de não poder mostrar, compartilhar a gestação, de como estava sendo legal… Então, nessa parte o meu emocional ficou muito abalado, até ganhar ela. Então em junho eu entrei em trabalho de parto e em contato com o obstetra, em contato com a fisio, eu tive uma aula de parto com ela online e aí lendo né. Minha preparação foi leitura, vendo vídeos, é , o grupo também né, proporcionou ali até o final as aulas, então a gente trocando muita ideia com as meninas do grupo, assim que uma ia ganhando, ia parindo né, cada uma compartilhava o relato, falava dos medos, falava das angústias, de como que foi, se pode entrar, quem não pode entrar, se teve acompanhante. E no dia assim mesmo o que aconteceu que eu ganhei, tive ela no Ilha, e a minha fisio ia acompanhar o parto, só que com a pandemia a maternidade não tava cadastrando novas doulas e fsioterapeutas pélvicas, então só aquelas que tavam cadastradas, eles não tavam pegando novas, então eu não consegui o acompanhamento dela, eu entrei em trabalho de parto, tive contração, peguei o médico plantonista assim que o atendimento foi bem ruim e me mandou de volta pra casa, mas eu tava com muita dor e nenhuma dilatação.

Até que no meio da dor assim, em contato com ela, eu liguei pro meu médico e a gente fez a cesária, intraparto ali né.

Entrevistadora: Sim, chegasse a conhecer a maternidade antes?

E1: Não podia entrar, na época tava fechado os espaços ali na sala de parto, tava fechado para visita. Eu tinha plano de saúde então a opção que eu tinha era ali ou a Santa Helena e ali no Ilha eu ia ficar no apartamento sem custo nenhum né, por isso que eu optei pelo Ilha né. E aí, eu preferia pagar se eu precisasse, ah se fosse pra pagar alguma coisa eu preferia pagar o médico assim, do que pagar uma equipe pro HU… porque tava tudo muito incerto sabe, muito. Tudo isso somou pra eu tomar a decisão pra eu optar pela cesárea, o medo assim de daí eu voltaria pra casa mas também ficaria eu e meu marido ali sozinho, a gente não sabia… sabe? Tinha um medo de chamar a fisio em casa, nossa foi horrível, horrível, foi uma experiência péssima. Tanto o parto quando o puerpério né, foi bem difícil.

Entrevistadora: Imagino, foi sua primeira filha?

E1: Foi minha primeira filha.

Entrevistadora: E qual achas que foi a influência do grupo de gestantes nesse teu processo?

E1: Então, o grupo foi assim, foi bem importante pra gente manter essa troca de experiências assim. Como assim, a gente não tava podendo, tipo eu via minha mãe de março até junho eu via minha mãe pouquíssimas vezes, porque dai a gente já escutava muito relato de gestante internada… Não via ninguém assim, ao ar livre mesmo, acho que tu lembra como que foi ano passado até junho assim, a gente não saia de casa mesmo assim, ninguém saia. Então eu não tinha essa troca com mulheres mais velhas, com tias, com primas, claro a internet ajuda mas é diferente. Então ali a gente criou um laço muito forte, muito assim, a gente conversa coisas que às vezes a gente não conversa com marido, com pai nem com a mãe, porque cada uma sabe que a outra tá passando pelo mesmo processo, então foi bem importante. O grupo de gestantes eu digo que foi fundamental nesse contexto de pandemia, não sei se fora a pandemia, nos grupos anteriores se as mães mantinham essa troca forte assim sabe, se conseguiu manter um laço tão duradouro como a gente ta tendo assim, cada etapa a gente consegue, primeiro foi o parto, depois foi o puerpério, amamentação, depois introdução alimentar… agora assim as bebês tão indo pra creche, as doenças da creche, então tá sendo bem legal pra gente trocar essas ideias assim. Eu assisti muita aula assim, procurava no youtube… mas ao mesmo tempo eu fugia um pouco, porque eu tinha medo do parto normal então eu não conseguia ver video do parto normal, eu tinha muito medo assim, de não ter o apoio ali sabe, talvez se fosse em outro contexto e eu pudesse procurar mais ajuda, né, pudesse conversar com a doula, eu lembro que na época eu fazia terapia e a minha psicóloga falava “olha, quem sabe tu não marca uma consulta com a doula, uma conversa” e eu assim “não, eu não posso sair de casa”, eu tinha muito medo de sair de casa, porque realmente né. E então, tudo dificultou pra eu procurar outros meios pra me preparar sabe.

Entrevistadora: E chegasse a pegar aquela fase ali que que teve a restrição do número de consultas do pré-natal também? Seu companheiro chegou a te acompanhar nessas consultas?

E1: Nas primeiras só, ali na, acho que eu tava na metade da gestação ali, no sexto mês, quando chegou a pandemia eu tava com 24 eu acho. E ele acompanhou ali e depois era tudo me deixando na porta e eu entrava via tudo né, consultava tudo e ele também não conseguiu se fortalecer sabe. Ele também tinha muito medo do parto e ele não conseguiu se… também ter a segurança da médica, sabe, porque a gente não teve esse ao vivo ali né, até o final ali. O médico mesmo, eu tive que mudar de obstetra porque minha médica que iniciou tava grávida e parou de atender e aí eu tive que mudar de obstetra e eu conheci no parto meu obstetra. Ah, e outra coisa que eu fiz também, eu comprei um curso online com uma consultora de amamentação, é um curso de gestantes que ela tinha feito antigamente, era presencial só que foi feito online na pandemia, aí foi vários assuntos também, parto amamentação, cuidados com o recém-nascido, cada semana eu acho que era um tema assim.

Entrevistadora: Ah, que legal. E quando chegou o momento assim, que você identificou "Ah eu acho que agora eu tenho que ir pra maternidade", como você estava se sentindo?

E1: Então, pouco assim, até hoje eu fico me, vendo às vezes né, no instagram algumas mães ali video ali e relatos assim, e eu fico pensando se era mesmo o momento de eu ter ido, porque o que que eu fiz, eu fiquei controlando no aplicativo né, controlava ali a contração quando iniciava e quando parava, quando iniciava e quando parava, aí chegou o momento que o aplicativo começou a dizer "prepare-se, vá até a maternidade" e eu mandava pra minha fisio, mandava ali porque saia um relatoriozinho né, dos números ali, do tempo. Eu mandava pra ela e ela dizia "não, então vai", teve uma hora que deu uma contração muito forte ali uma dor muito forte, daí eu comecei me ajeitar e fui, não cheguei a ir pra chuveiro e nada, porque eu fiquei com medo de dai acontecer alguma coisa e eu não ter ninguém, não ter suporte, só eu e ele, completamente inexperientes ali né, a pandemia atrapalhando, ai a gente foi pra maternidade, foi o único suporte que eu tive, foi assim.

Entrevistadora: Sim, se quiseres colocar mais alguma coisa que achas que é importante falar, de como que foi que tu descreve esse processo assim da pandemia mesmo, do sentimento geral assim, de como que foi pra você.

E1: É então, pessoalmente pra mim foi super difícil, não falando só quanto ao parto, porque o parto, olhado o que eu já passei até agora, o parto é uma pequena parte de tudo que é, dessa loucura que é a maternidade assim, e o puerpério pra mim foi muito difícil, a questão da amamentação eu não consegui amamentar, eu não tive suporte do banco de leite, tava tudo fechado eu trouxe uma consultora da amamentação aqui umas três vezes, já tinha gastado rios de dinheiro e não conseguia resolver e eu acabei desencadeando uma depressão pós-parto, que daí eu tenho bastante consciência hoje que também foi por conta do contexto da pandemia né, que se não tivesse eu acho que tudo fluiria mais tranquilo, desde o parto, né pensando nas mães que tiveram, que pariram antes de mim, que eu acho que agora quem

engravidar a partir de agora todo mundo vai ser afetado de alguma forma né, apesar de que a gente já tá vacinado, já tem ali o estudo da amamentação que protege e tal, mas todo mundo vai ficar com resquício assim do que foi essa pandemia na vida de uma gestante, na vida de uma mãe é, especialmente ali no primeiro ano de março do ano passado até o final do ano de dezembro, foi muito difícil, de não ter a rede de apoio, de tu não pode compartilhar a gestação, de tu não ter a segurança de talvez procurar o profissional mais adequado, até assim coisa pequena, do medo na maternidade sabe, de não sentir segurança, sabe, de sair do, eu lembro que tava com problema na amamentação e eu queria chamar uma enfermeira, eu morria de medo de sair no corredor, porque ainda era tudo muito incerto assim, desconhecido, acho que com o tempo as coisas vão melhorar assim, mas pra gente que pegou primeiro momento ali foi muito cruel, foi muito difícil.

Entrevistadora: Sim, com certeza.

E1: Mas enfim, deu tudo certo. Passou, passa, mas é difícil assim. Não sei se queres perguntar mais alguma coisa, também específico do que tu precisas.

Entrevistadora: Não não, falasse muita coisa importante né, pra gente tá refletindo e era bem isso mesmo, da tua vivência mesmo, e assim, por mim a gente pode encerrar eu queria muito te agradecer. E se você achar que tiver mais alguma coisa que você queira falar, pode me mandar. Mas muito obrigada.

E1: Obrigada também, depois tu compartilha o teu trabalho pra gente ver como ficou.

Entrevistadora: Pode deixar, tá bom, obrigada, tchau.

E1: Obrigada, tchau.

ENTREVISTA 02

Data da entrevista: 26/10/2021
DATA DO PARTO: 03/06/2020

Entrevistadora: E como que foi, como você se preparou (para o parto)?

E2: Quando começou a pandemia eu já tava grávida dela né. Eu não engravidei na pandemia né, eu já tava grávida dela e quando eu tava com 15 semanas de gestação, foi em dezembro né, a pandemia começou e março, na metade de março assim, então antes em dezembro eu tive apendicite, eu tive que fazer a cirurgia, fiz no HU também e o pós-cirúrgico foi bem dolorido pra mim, foi bem complicado. E eu ja queria fazer, já pensava em fazer parto normal né, e ja tinha escolhido o HU por ser um hospital humanizado né, referência em parto humanizado no Estado e tudo e aí como o pós-cirúrgico foi bem complicado pra mim foi mais uma questão pra eu dar preferência, assim tentar né, fazer o possível pra que o meu parto fosse natural também, porque eu ficava pensando assim, eu não vou conseguir passar por outra cirurgia né, de cesária, e com o bebê recém-nascido, foi bem complicado mesmo pra mim. Aí então eu ja tinha me inscrito pra participar do grupo e aí o grupo começou, foi o primeiro dia de, foi bem na metade de março assim e aí naquele dia mesmo eu acho, foi numa quinta-feira eu lembro bem, o encontro foi na quinta-feira o primeiro encontro e na sexta-feira começo a pandemia, fechou tudo tudo tudo, então a gente só teve um encontro mesmo. E aí o meu objetivo de participar desse encontro né, uma que todo mundo já me dizia que era o melhor grupo de gestantes, era o do HU assim né, todo mundo que já tinha feito até o particular da UNIMED, sempre falava que o melhor era do HU. E aí como eu queria também informação, queria fazer parto normal, foi um dos motivos que me levaram assim né, pra mim foi ótimo assim porque durante os encontros elas explicaram tudo né, tudo tudo tudo, tiraram todas as dúvidas assim, foram super incansáveis mesmo né. Como a gente não pode fazer a visita elas mostraram a sala de parto, pré-parto e pós-parto, então assim eu sabia tudo tudo tudo né, porque eu queria muito que meu parto fosse natural e aí meu parto foi realmente, foi bem natural, evoluiu super rápido assim. A minha bolsa rompeu, não sei se queres que eu te conte...

Entrevistadora: Pode, pode contar!

E2: A minha bolsa rompeu eu tava com quarenta semanas exatas assim, quando eu ia pra quarenta semanas e um dia, na madrugada assim eu senti uma dor forte na lombar e aí virei assim na cama e rompeu a bolsa, aí eu levantei fui tomar banho, arrumar o que faltava pra arrumar e aí a gente foi. A bolsa rompeu eram 02h12 acho que eram e a gente chegou no HU umas 3h30 mais ou menos, levamos uma hora, e aí tava aquela história de um dia podia entrar acompanhante outro dia não podia, um dia podia e o outro não podia e uma das meninas do grupo tinha dado entrada no dia assim sabe, durante o dia, e não podia entrar acompanhante, então eu tava bem nervosa assim por causa disso. Só que como a gente foi de madrugada não tinha ninguém assim, só tava eu mesmo, aí o guarda ali pegou e já pediu pro meu companheiro entrar junto e aí ele já ficou junto comigo né, como não tinha ninguém. Aí

quando eu cheguei a médica que me atendeu era a [nome da profissional] que tava no plantão, ela avaliou ali e viu que eu já tava com quatro dedos de dilatação e aí já me levou pra sala ali, já fez a entrada né, pra sala de parto ali, aí eu fiquei ali, fiquei indo pra, eu ia tomava banho, voltada pro quarto ia pro banho e voltava pro quarto e aí foi indo indo indo até que numa delas assim eu senti que as contrações começaram a ficar bem bem bem forte mesmo, aí sim, no decorrer eu não sentia muita dor assim, mas chegou um ponto que ficou bem forte aí eu fui pro quarto aí nisso a enfermeira já tava ali pra fazer aquela ausculta cardíaca né que ela ia de tanto em tanto tempo, aí já tinha trocado plantão a médica já tinha ido se apresentar pra nós mas era só isso assim, daí depois eu voltei pro banheiro sentei no vaso e comecei a fazer muita força, muita muita, ai eu já não tinha mais dor, dai eu comecei a fazer força, dai eu me dei de conta "eu acho que eu ja to no expulsivo, eu acho que ela já vai nascer né" aí a gente foi pro quarto e chamou a médica, aí quando eu cheguei no quarto e já me sentei no banquinho de cócoras, era a melhor posição pra mim. Aí a gente chamou a médica e ela assim "Como assim já vai nascer?", porque ela só tinha ido ali se apresentar pra gente acho que ela não imaginou que ia evoluir tão rápido né, eu toquei assim eu já senti a cabecinha dela saindo, já senti o cabelinho assim, aí ela olhou e falou assim "É já vai nascer mesmo, pode chamar a equipe" aí ela só veio assim, amparou a [nome da filha] e ela já nasceu e, ah foi super tranquilo assim, muito tranquilo. A [nome da filha] nasceu, eu cheguei no hospital umas 3h30 e até avaliar, a gente foi pra sala e tudo, ela nasceu às 9h10, pra mim foi rápido assim.

Entrevistadora: E quando tavas nesse processo, conseguisse lembrar do que foi falado no grupo? O que achas que foi importante pra que você conseguisse passar por esse processo, teve alguma coisa?

E2: Eu acho que o que foi importante é que eu já tava familiarizada com tudo, elas já tinham me falado como que era, que a enfermeira vinha de tanto em tanto tempo alí, eu já conhecia a sala, eu já sabia o que que ia acontecer ali sabe. E tudo isso mais ou menos eu já tava familiarizada, sabe, foi isso assim.

Entrevistadora: Chegasse a fazer um plano de parto?

E2: Fiz, mas na hora eu nem apresentei, eu nem cheguei a apresentar, ficou dentro da minha carteirinha assim, eu não cheguei a entregar em mãos pra médica, porque foi tudo tão assim rápido e a gente fica tão em função assim, sei lá, eu fiquei bem… Eu fazia bastante as respirações assim, que a gente tinha conversado né, foi o que me ajudou muito foi ter feito as respirações, aí isso uma das colegas do grupo passou também umas vocalizações que eu fazia e foi o que me ajudou muito assim a ficar tranquila, relaxada, eu fiquei super tranquila.

Entrevistadora: E como foram as tuas consultas de pré-natal?

E2: Foram no posto de saúde, eu moro na Barra da Lagoa né e aí foram todas no posto de saúde o médico foi maravilhoso, que me atendia, o [nome do profissional], ele que diagnosticou inclusive que eu tava com apendicite e aí na hora ele ja me mandou ir pro HU e

eu cheguei no HU e todo mundo tinha dúvida e realmente, levaram um tempão pra fazer o ultrassom, e realmente era assim. As minhas consultas com ele foram ótimas, com a enfermeira [nome da profissional] também que era minha enfermeira aqui do posto ela é muito (perda de conexão). Pra mim foi tudo tranquilo assim o pré-natal, claro que quando entrou na pandemia eles deram uma parada né, mas aí depois voltaram aos atendimento e aí foi tudo bom, tudo bem assim. Não tinha problemas no pré-natal.

Entrevistadora: E o teu companheiro ele podia participar junto, teve alguma restrição por conta da pandemia que achas que tenha influenciado?

E2: Eu costumava ir sozinha porque ele trabalhava, e aí as consultas eram bem assim no meio do dia, era o horário que o posto marcava né, então ele não podia tá saindo do trabalho pra ir. Então eu geralmente ia sozinha, aí a gente fez dois ultrassom particular, que foi aquele da translucência e depois o morfológico e aí nesses ele foi comigo, ai eu marquei acho que foi no sábado de manhã que a gente foi, aí ele foi comigo mas nas consultas não, eu ia sozinha. Aí o nosso medo era esse de na hora do parto ele não poder me acompanhar né, porque nós os dois estávamos assim estudando bastante pra entender tudo assim, sabe, e aí eu falava pra ele "sozinha eu tenho medo de ficar sozinha né, eu preciso de alguém pra me ajudar, me acompanhar" e ele tava junto comigo ele sabia tudo que que eu queria, e era isso que tinha medo de ele não poder entrar na hora porque a gente tava ouvindo ali alguns relatos de alguns casos que o acompanhante não pode entrar, mas aí pôde.

Entrevistadora: Em que meios que vocês buscavam o acesso a informação, como que foi pra vocês, como vocês buscavam informação pra se preparar pro parto?

E2: A maior parte mesmo foi através do grupo, eu fiz antes desse do HU, nós os dois participamos do grupo do IFSC porque era a noite e ele podia ir, nesse do HU ele não ia poder participar então a gente participou do grupo do IFSC e aí elas passaram bastante vídeo, bastante informação assim, então pra ele a maior parte da informação foi essa assim, aí depois eu participei sozinha porque era de tarde os encontros do HU e o resto assim, elas colocavam materiais ali pra nós, porque a gente fez um grupo de gestantes também né, esse grupo se formou com as gestantes e a gente trocava informação através dali, bastante informação eu seguia muita obstetra também no instagram, grupo de parto então eu buscava bastante informação ali também.

Entrevistadora: Tu achas que conseguisse te empoderar no teu parto?

E2: Então quando a gente chegou no parto eu não tinha dor, eu tinha uma contração leve. Eu tinha contração bem leve, as minhas contrações quando vieram já eram no final mesmo do expulsivo assim, um pouquinho antes do expulsivo, aí quando a gente entrou na sala, nessa sala que é a sala de parto né, a enfermeira veio nos atendeu e aí ela deixou a bola, a bola de pilates, ela deixou um massageador, um olhinho pra massagem também, ela falou que a gente podia usar o banheiro à vontade, o chuveiro ali a vontade, então ela apresentou esses métodos assim né. Como eu não tive muita dor eu não precisei chamar elas em momento nenhum, nós

ficamos sozinhos, eu ia ficava um pouco no chuveiro e voltava pro quarto, ia e voltava, eu caminhava muito, eu não conseguia ficar parada, eu ficava caminhando assim, eu ia e ficava lá e ia no chuveiro e fazia uns movimentos assim sabe e que aliviavam assim a dor e aí voltava pro quarto e já voltava, e aí eu ficava transitando muito então elas só iam ali perguntavam se tava tudo bem, auscultava e já voltava pro, e já saia assim, a gente não precisava de nada e ja saia. E aí foi evoluindo muito rápido, tanto que quando a gente chamou elas pra avisar que a [nome da filha] já tava nascendo elas nem acreditaram porque foi muito rápido. Mas a gente sabia de tudo assim, eu sempre falava pra ele se vierem colocar acesso por algum motivo a gente não quer, a gente não quer nada nada nada farmacológico assim, eu queria totalmente natural. E aí o que me ajudou também, o que eu acho que me ajudou muito foram as respirações e as vocalizações que eu fazia na hora das contrações, isso eu já vinha treinando antes do parto.

Entrevistadora: Ah sim, então já fazias esse processo de respirar desde a gestação então?

E2: Já desde a gestação, eu fazia todos os dias, eu inspirava soltava o ar e relaxava o períneo, eu fazia as respirações associadas ao relaxamento do períneo e fazia as vocalizações também. Tem uma vocalizações, eu não sei se tu conhece mas tem umas vocalizações no youtube que eu acho que é canto carnálico se eu não me engano, que é assim associada também, faz a vocalização associada a respiração e aí relaxa. E foi o que me ajudou assim, na hora da dor mesmo. Eu seguia uma obstetra e um dia ela colocou um relato e aí ela falava alguma coisa assim de que a contração é como uma onda, ela chega no ápice da dor e aí quando ela chegou no ápice da dor ela começa a diminuir, então na hora eu colocava, aquilo ali era o que eu mentalizava assim sabe, eu ia aguentando aguentando aguentando e quando ela chegava no auge da dor eu pensava assim "ah agora ela vai diminuir" e aí eu ia fazendo as repirações e as vocalizações e ia relaxando, relaxando, relaxando. Eu fiquei bem concentrada, muito muito concentrada.

Entrevistadora: Que ótimo , pelo teu relato a gente percebe que tinhas muita consciência corporal asim, de tudo que tava acontecendo, de como que tu ia lidar com isso.

E2: Foi fundamental, fundamental. E aí ja ia fazendo esse treino durante a gestação né e aí na hora que eu tava lá pra ganhar a [nome da filha] eu não fiquei em momento algum pensando "ah será que ja tava na hora, será que ja to com tanto de dilatação", sabe, eu não fiquei racionalizando nada sabe, eu fui só tentando levar, quando vinha a dor eu só pensava em tentar aliviar aquela dor, e deixei, não fiquei prestando atenção "ai quando tempo será que eu ja to aqui, será que ela já vai nascer será que não vai", tanto que quando eu comecei no expulsivo eu comecei a fazer força força dai que eu me dei de conta de, peraí, eu acho que já não to mais nas contrações, eu acho que ja ta na hora dela nascer, então eu ia indo muito assim sabe, eu não racionalizei nada eu fui levando. E sobre aquela pergunta que tu tinha feito de informação né, quando a gente fez o curso aí elas falaram que a posição que eu ia ganhar a [nome da filha] era a melhor posição que fosse pra mim, eu escolhia a posição né, elas mostraram que tem disponível lá, isso no curso, elas mostraram que tinha disponível o banco de cócoras, que tinha a maca, mostraram fotos da maca como a gente não pôde

conhecer a sala elas fizeram um vídeo e tudo né, e o tempo todo que eu tava em trabalho de parto a melhor posição pra mim era sentada no vaso, eu sentava e ficava relaxando um tempão assim e depois eu ia pro chuveiro, então na hora que eu saí, que eu tava no banheiro eu tava no vaso e eu fazia muita muita muita força, ai com certeza eu tava no expulsivo. E aí quando eu fui pro parto eu só conseguia sentar no banco de cócoras, eu não conseguia nem sentar na bola a melhor posição pra mim foi no banco de cócoras aí quando a médica chegou ela falou assim "ah agora quando aliviar tua contração a equipe vai chegar e tu vai te deitar na maca" ai eu falei pra ela assim "não pra maca eu não vou ir, eu não vou conseguir ir pra maca" e ai ela assim "vai sim, a gente vai te ajudar e vai te levar pra maca" aí eu falei assim " não, daqui eu não saio e daqui ninguém me tira, eu vou ganhar ela aqui" aí ela pegou e disse assim "ah a tua sorte é que eu to com o joelho bom, traz lençol" aí mandou trazer lençol e ajeitou ali, então isso foi uma informação que me empoderou assim, porque uma que eu não ia sair dali porque eu não tinha condições realmente, ela ja tava quase nascendo era a melhor posição ali pra mim, eu não conseguia ficar deitada nem sentada na poltrona e outra também que eu já sabia que eu poderia escolher a posição que fosse melhor pra mim né.

Entrevistadora: Que legal ouvir isso! E voltando um pouco, como é que tavas se sentindo um pouco antes da [nome da filha] nascer assim, você percebia que tava chegando perto do momento, você conseguiu identificar, como que foi?

E2: Foi meio do nada assim, eu não tinha nada que eu achasse assim eu até ficava com medo que pudesse passar muito das quarenta semanas e tivesse que induzir, alguma coisa assim sabe, eu já vinha sentindo uma dorzinha na lombar, só que eu não sabia que aquilo ali já eram as contrações de treinamento, porque eu nunca tive cólica, até hoje, quando eu vou ficar menstruada eu sinto essa dor na lombar, tipo uma lombalgia assim, e aí eu já vinha sentindo essa dor na lombar e eu sentia também vontade até na última consulta com a enfermeira eu falei pra ela, eu sentia vontade de fazer cocô, mas eu ia no vaso assim ia no banheiro e não saia nada, era só uma vontade assim, parece que eu tava sempre com vontade de fazer cocô. E essa dorzinha na lombar que vinha de vez em quando, aí comecei a observar essa dor na lombar se ela tinha uma frequência, mas vinha ficava um pouco depois parava e depois pro final assim perto de eu ganhar a [nome da filha] ela já nem tava tão frequente, não sei se eu já tinha acostumado, então eu não tava com muitos sinais, o que aconteceu mesmo foi que rompeu a bolsa e eu fui e no caminho que eu comecei a perceber que essa dorzinha na lombar começou a ter uma frequência e eu comecei a contar e eu tinha, eu acho que eram três a cada cinco minutos que elas falam né, pra ir pra maternidade né. E aí eu comecei a contar e via que já tava três a cada dez mas não era dor, era aquela dorzinha na lombar só, não era uma dor assim.

Entrevistadora: Uhum, entendi. Então o momento que você decidiu ir pra maternidade foi porque rompeu a bolsa mesmo?

E2: Foi, é porque elas ja tinham falado né, que quando rompe a bolsa a gente tem que ir fazer avaliação, não pode ficar em casa esperando. Mas eu fui bem calma assim, eu tomei banho, ai como era de madrugada eu ainda falei pro meu companheiro né "toma café, come alguma

coisa porque a gente não sabe se vai demorar muito tempo né, aí depois lá não vai ter como comer" eu nem comi nada porque eu já não sabia como ia ser mesmo e eu não comi nada.

Finalização

ENTREVISTA 03

Data da entrevista: 27/10/2021
DATA DO PARTO: 23/04/2020

E3: Então, a minha gestação foi uma gestação planejada né, eu sou casada, minha gestação foi bem tranquila assim e fui uma grávida bem saudável assim, e tudo certo, tudo bem bom durante a minha gestação e durante toda a minha gravidez eu me planejei fisicamente, emocionalmente pra ter um parto bem natural assim, cheguei a inclusive cogitar o parto domiciliar, tava em acompanhamento com minha médica obstetra mas também com uma parteira que tava me acompanhando por fora né. E aí, tava tudo correndo bem e tal, e aí quando foi ali em fevereiro, final de fevereiro assim, a minha data provável do parto era 29 de abril, então assim quando foi acho que final de fevereiro quando eu tava entrando no oitavo mês, eu comecei a fazer um pouquinho de pressão alta, não chegou a ser uma pré-eclâmpsia mas era uma hipertensão gestacional e aí eu tive que cuidar, fiz algumas alterações na minha alimentação enfim, e tava tudo bem tava só monitorando e tudo certo, fazendo a consulta e tal. E aí quando veio a pandemia em março né, mais assim final de março acho que foi meado de março aí eu já logo parei de trabalhar, eu acho que o último dia que eu trabalhei presencialmente foi 15 ou 16 de março, trabalhava em uma empresa e deixa eu me lembrar... ata ai eu parei de trabalhar e aí assim, eu parei de fazer exercício, na época eu fazia pilates e tava fazendo pilates e yoga, ai parei de fazer os exercícios e parei de ter vida social e aí entrou num modo de preocupação intensa né, porque hoje, não que hoje esteja menos perigoso mas hoje a gente tem mais informação né, hoje a gente tem mais conhecimento do que que dá pra fazer, até onde dá pra ir, quais são os verdadeiros riscos né e naquela época não se tinha, então eu lembro assim que eu fiquei sem ver a minha família até depois da [nome da filha] nascer. Ai eu e o meu maridos fomos pra, a gente mora num apartamento aí nós fomos pra nossa cassa de praia da minha família e aí ficamos os dois lá sozinhos com meu cachorro e aí assim, uma solidão profunda né, final de gravidez assim, barriguda queria ver todo mundo e as coisas não foram possíveis né, e assim por sorte e eu agradeço, eu já tinha feito o chá de fraldas e já tinha feito books de gestantes então essas duas lembranças assim eu tive na hora certa porque muita gente não teve né, e aí eu lembro assim, até que minha mãe passou lá na praia um dia pra me entregar um bolo uma coisa assim e aí eu toquei na mão dela assim de longe, e logo já corri pra lavar a mão e acho que ela da mesma forma porque a gente não sabia né, eu não sabia se ela era doente, eu não sabia se, como é que aquilo ia funcionar pra mim e aí eu até me emociono de lembrar, e aí foi difícil!

Entrevistadora: Eu imagino, se quiser parar um pouquinho...

E3: E aí começou uma onda de não permitirem os maridos a entrar nos partos, começou muito forte no HU e aí eu soube até de uma conhecida que não pode o marido entrar, só entrou, eu acho que só entrou quando o bebê nasceu. Tinha algumas outras até, acho que algumas do grupo de mães que durante todo o trabalho de parto que um determinado momento ela já tinha ido pro hospital e ele só entrou quando começou praticamente o expulsivo assim, quando já tava saindo, então todo aquele momento ela ficou sozinha então

vai assim, afetando a gente né, e aí imagina que eu tinha toda uma preparação pra ser domiciliar, pra ser na minha casa, na minha família com meu marido assim e aí daqui a pouco eu não ia ter nem meu marido, não sabia nem onde que eu ia poder fazer, quem que ia ta comigo, aquilo me deixou bem bem mal. E aí nisso eu continuei com acompanhamento tanto da parteira, tanto do meu médico e aí minha pressão ferrou assim, porque aí mudou minha alimentação, parei de me exercitar e aí assim, muita preocupação dessa insegurança né, e aí minha pressão começou a subir muito já tava ficando em 15/10 mmHg tava bem alta e aí foi isso assim, e aí a pressão foi ficando alta daí eu comecei a ficar bem mas de repouso meu médico pediu pra eu realmente dar uma para, e eu consegui parar, aí fiquei bem mais de repouso de tal e aí eu tinha uma coisa que eu também não podia, porque se eu não me engano pras grávidas de hipertensão e de possível pré-eclâmpsia se recomenda tomar AS né, pra controlar a pressão, e eu sou alérgica a AS eu não posso tomar aí eu não pude nem tomar nenhum tipo de medicação pra controlar minha pressão e aí minha pressão começou a subir de descer muito assim e eu muito cansada, aí mas aí fui tocando assim, aí quando eu tava com 39 semanas, 39 semanas e 1 dia,e aí eu tive uma consulta, aí até foi engraçado porque eu e meu marido a gente tava na praia já fazia um mês e meio assim né, aí naquela semana eu falei assim " Vamos voltar de vez pra casa porque agora assim ja ta muito na reta final da gravidez, que aí ele tinha feito uma mini mudança pra lá né, de alimentação, de roupa de cama, produto de limpeza, comida tal, e eu falei pra ele "Vamos voltar pra casa, se a gente quiser vir a gente vem e passa um dia mas não vamos mais ficar aqui, porque a gente tá muito longe, imagina se no meio da madrugada acontece alguma coisa, imagina se o carro pifa aqui" sei lá, uma coisas que cai né, ai não então tá, aí acho que foi isso na quarta-feira de manhã nós fomos embora, não, minto, na quarta-feira à noite nós fomos embora porque na quinta de manhã eu tinha consulta das 39 semanas, e aí quando fui pra consulta, aí meu médico falou assim "Ah, não tem mais…", aí eu fiz um quadro também de polidrâmnio, que é um excesso de líquido na bolsa, e aí o médico falou assim "Ah, se tu fosse uma paciente que tivesse interesse numa cesárea eu já ia te recomendar cesária hoje, agora" né, porque aí minha pressão já tava em 16 assim, 15, 16, não tava mais baixando assim sabe, aí ele assim "Ah acho que agora deverias ir pra uma cesárea mas como tu não quer eu vou fazer a prescrição aqui" porque eu não… meu médico que me acompanhou durante o parto eu não chamei ele pra, minto, o meu obstetra eu não chamei pra me acompanhar durante o parto, aí ele falou " Ah, eu vou te prescrever aqui pro plantão pra tu ja se internar hoje porque tu precisa fazer a indução do trabalho de parto", aí eu concordei assim, entendi que não tinha mais razão pra ficar insistindo numa coisa que não tava boa pra mim porque eu já tava assim muito cansada, já tava… a reta final da gravidez a gente fica né, independente de ter pressão alta assim é bem puxado, e aí no dia foi uma correria né, porque eu só tinha uma consulta, eu sentia que a minha barriga tava alta ainda, eu sabia que não ia nascer por agora né, naturalmente, eu tava bem ciente assim, dos sinais que o meu corpo dava, ela ja tava de cabeça pra baixo mas ela não tava lá embaixo encaixada ainda. E aí eu fui pra consulta bem normal assim, fiz até um ultrassom naquele dia e aí, eu falei assim "puts", aí eu peguei eu já tinha consultado com uma outra médica que era assim do mesmo grupo de parteiras que eu já tava me consultando e aí eu liguei pra ela assim porque aí como surgiu essa situação de pressão de muita função, eu senti falta de ter um profissional de confiança ao meu lado, não que eu não confiasse no meu médico obstetra que me acompanhou na gestação mas eu sentia mais afinidade com ela assim, mais segurança

com ela, aí liguei pra ela falei "Ah doutora, assim assado, pode me ajudar?" e aí ela, "Não então tá, eu vou trocar meu plantão tal pessoa vai te acompanhar, vai tal hora" e aí acertamos tudo, aí foi uma correria, no dia assim né, porque ela falou assim "Ah tu podes ficar até 48h na indução de trabalho de parto e aí a gente vai colocando um remedinho la pra ver se dilata teu útero, mas pode ser que não dê certo também, mas a gente vai tentar e tal" e eu tava super tranquila, ai eu não então ta que bom, ai tipo pô aí a [nome da profissional] ia comigo, uma profissional…

E3: Aí a [nome da profissional] ia comigo eu tava de boa, a daí guria eu fui fazer supermercado, fui deixar meu cachorro em um lugar, fui até no correio pegar uns lacinhos que eu tinha comprado pra minha filha, dai meu Deus, tudo que eu tinha que fazer tinha que fazer naquela tarde, fui fazer exame de sangue, um monte de coisa. Aí internei cheguei lá tudo certo, aí combinei com ela 18h30 lá na maternidade, aí cheguei e comecei aqueles exames pré-internação, a ver pressão ver não o que, ver tipo sanguíneo "parara"... e fizemos aquele exame taquicardiográfico na minha bebê, aquele que bota uma fita assim na barriga e aí quando a [nome da profissional] chegou que já era assim umas 19h30 e ela percebeu que a [nome da filha] assim tava muito agitada, o batimento dela tava passando de 200 assim dentro da barriga e segundo o que la me falou o normal é assim 150, nessa variação, ai ela "ah vira de lado, faz assim, faz assado" e aí o batimento não baixava ai ela " Tais sentindo alguma coisa, ela ta se mexendo muito" eu não "Não, ta tranquila" ai ela assim "Tua bebê não tá bem ela ta com taquicardia, nesse jeito que ela ta, considerando teu quadro de pressão alta e esse polidrâmnio que tu tá, a gente vai ter que fazer a cesária agora", puts dai foi uma (suspiro), ai assim foi difícil. Ai eu disse "Ah então tá doutora, pra mim a saúde, o sucesso do meu parto é minha filha nascer com saúde né, não tem outro caminho", aí chamamos meu marido conversamos e fomos guria e fomos pra cesárea, a cesárea pra mim foi bem, bem ruim, porque a enfim, como ela tava muito alta assim, meu marido falou que viu ela enfiando o braço até o ombro assim pra pegar a [nome da filha] e aí aí, aí um monte de gente me sacudindo me apertando, aquele negócio todo que eu tinha lutado pra evitar, eu fiz fisioterapia pélvica, fiz pilates, tava fazendo massagem, um monte de exercício e tal pra me preparar pra um parto normal e não tive nem chance, e aí guria foi assim, me internei às 18h30 às 20h30 ela nasceu.

E3: Foi rápido, mas aí foi tudo certo, depois assim, aquele pós-operatório de cesárea, pós-cirúrgico de cesárea que é chato, e aí o pós o pós-nascimento da [nome da filha] que foi mais difícil porque aí a gente não abriu pra ninguém a visita, o que nós combinamos foi a minha mãe né, que minha mãe é aposentada, e ela ja vinha assim mais em casa com mais segurança, porque naquela época era tudo assim muito potencializado, então assim se minha mãe ia num mercado se ela ia numa loja, se ela ia, pra mim eu já tinha que ficar duas semanas sem ver ela porque ela podia ter sido contaminada e aí parece até meio infantil assim a gente olhando o contexto de hoje, mas naquela época era assim que a gente pensava, lá em casa a gente lavava todas as compras e aquela coisa. E aí a minha mãe foi lá em casa pra conhecer ela né porque a gente não pode ter visita na maternidade nos dias que teve lá o que eu achei foi bem bom sabe, porque eu não imagino uma maternidade cheia de gente e eu ali toda costurada toda, passando perrengue pra amamentar, enfim, e aí meu sogro e a minha sogra

foram la em casa mas nem pegaram ela no colo, tavam cheios de roupa cheios de luva, ai assim, muito triste, é triste assim de lembrar porque assim, a gente acabou privando as pessoas de conviver com ela, deu tudo certo sabe, porque tanto que até hoje nenhum de nós três pegou, nem meu sogro nem minha sogra, nem meus pais, ninguém teve covid, porque a gente se cuidou bastante mas teve um preço assim sabe, de convivência e tal. E aí a gente ficou assim, ai meu sogro e minha sogra iam assim, cada semana sim semana não, aí cheio de, até tava revendo uma fotos ontem, tô fazendo um albinho pra ela, eu comprei aquelas roupas, aí tipo de TNT sabe, de amarrar atrás que vinha até aqui assim, com manga cumprida, então todo mundo que ia lá em casa, todo mundo, meu sogro e minha sogra, usavam luva, usavam máscara, usavam aquela roupa pra pegar nela e sempre sem sapato tal e assim, na minha família a gente tem isso, de oferecer um cafezinho né de fazer uma social, um lanchinho e tal e aí não oferecia nada, mal e mal oferecia água né pra pessoa não ter que tirar a máscara e foi isso assim, e foi bem difícil sabe, porque por exemplo eu tenho dois irmãos, eu tenho três irmãos né, dois dos meus irmãos assim já desde o início não se cuidavam, tiveram uma outra maneira de encarar tudo isso, e aí assim eu não convivi com eles por meses assim, eu acho que um deles só conheceu a [nome da filha], aí um deles teve covid tipo ela nasceu em abril ele teve covid, ele e a família né, minha cunhada e sobrinhos tiveram covid em julho, aí quando passou o covid deles ai ta, eu sei que tem aquela janela imunológica do pós covid aí que eles foram lá conhecer ela, mas mesmo assim de roupa de luvas de tudo. É, e meu outro irmão acho que foi outubro quase seis meses ela já tinha e isso assim com muita pressão, muita pressão da família, a gente sabendo que já tinha até família, amigo que assim ria da gente, que nós estávamos neuróticos, exagerados, e isso assim, pra quem ja tá no puerpério, pra quem já tá com restrição e toda dificuldade que a gente tem de adaptação da vida de cuidar de bebê de acordar de madrugada de amamentação, daquela função que antes a vida era de um jeito e agora nunca mais vai ser, foi bem pesado, bem pesado. E foi isso assim, resumidamente né, ai eu acho que foi quando foi mais pra setembro, outubro do ano passado a gente foi conhecer mais algumas pessoas e tal aí minha vó foi lá visitar, aí aos pouquinhos a gente foi indo assim, mas muito aos pouquinhos, foi bem lento o nosso processo de convivência com as pessoas e isso foi bem difícil.

Entrevistadora: Falasse que um certo momento ali em abril né, parasse um pouco com as tuas preparações, como que funcionou as tuas consultas de pré-natal?

E3: A isso já foi assim, ó a minha preparação já desde de, desde bem cedo assim foi dupla né, que era frequentando o obstetra e fazendo as consultas com a parteira, ai isso seguiu normal assim e aí eu fui me cercando de orientação, tanto de amigas que seguiam a mesma linha que eu queria que já tinham passado por aquilo, quanto de profissionais de internet que eu fui descobrindo e que eu fui tendo uma afinidade né, toda essa preparação ela foi muito tranquila assim, ela foi bem encorajadora, eu tava, antes de eu engravidar, até quando eu me engravidei assim, na semana que eu descobri que eu tava grávida eu lembro que eu fui ver uns vídeos de parto assim porque nunca tinha visto realmente né, tipo agora tava em mim, tava ali o neném ia ter que sair e eu fui ver, eu pensava meu Deus nunca que eu vou fazer um parto normal, nunca que eu vou deixar um negócio daquele tamanho passar por aquele buraco ali e aí essa minha preparação foi bem engrandecedora do meu potencial enquanto mulher, de gerar,

confiar na minha natureza e no que eu sou capaz de fazer né, que hoje eu considero assim, que a nossa natureza é perfeita, claro que Graças a Deus existe a ciência que possibilita que em situações como a minha em que a minha bebê não tava bem, podia ser que se tivesse seguido o plano talvez tivesse seguido tudo certo, mas talvez não desse, então a gente pode escolher não correr aquele risco, mas que eu vejo assim e ouço de algumas amigas é as cesáreas desnecessárias, a má informação, a gente vê que há um interesse financeiro no controle do tempo, pra não ficarem muito tempo lá em trabalho de parto ocupando uma sala, um quarto no hospital, e aí isso, e aí eu ter tomado consciência disso pra mim já desde o início da minha gestação isso já me deixou muito forte assim, tanto por exemplo pra eu não querer que meu médico me acompanhasse porque eu sei que ele era muito nessa linha da cesárea, aí eu fui atrás de, conversei com enfermeiros que trabalhavam na [nome da maternidade], sempre que eu ia na consulta eu puxava papo com uma com outra, eu tive uma amiga que trabalhou lá e que me contou qual era a fama dele, então eu sabia que ele era um bom médico pra me acompanhar, pra medir, pra me indicar qual a medicação, o que fazer, qual exame e tal, mas não era o cara que eu queria que tivesse na reta final comigo. E aí a ajuda das parteiras também foi bem legal porque aí tipo, de fazer o exercício, tipo ó "Pegue esse caminho aqui", por exemplo, quando eu descobri que eu tive pressão alta a [nome da profissional] me falou assim "Come proteína a cada duas horas", isso é passado entre parteiras, porque quando tu tem pré-eclâmpsia se eu não me engano tu começa a perder proteína na urina, e aí quando tu repõe aquilo a cada duas horas tu pode minimizar esse efeito, então isso foi bem legal, eu tava muito consciente assim do que que eu queria, muito consciente assim, tanto que eu me senti forte o suficiente pra chegar lá na hora e dizer "Não, eu não vou com ele, eu vou chamar a [nome da profissional], eu vou aceitar a indução do trabalho de parto" e me senti tranquila pra receber o diagnóstico dela e saber que eu tava em boas mãos, porque essa insegurança em relação aos médicos eu também vivi e por isso que naquele momento que a coisa não tava no caminho que eu tinha planejado né, do natural, ai eu achei melhor ir nas mãos de uma profissional de confiança.

Entrevistadora: Sim, e qual tu acha que foi a influência do grupo de gestantes nesse teu processo de se preparar pro parto?

E3: Olha, vou ser bem honesta assim, porque como o nosso grupo ele não foi o presencial e eu já entrei no grupo de gestantes eu acho que eu ja tava com 34 semanas que foi ali em março né, a primeira reunião foi em março, então eu acho que assim, na minha preparação para o parto não foi tanto, mas no meu pós-parto e até hoje assim, ele mudou o meu maternar assim, eu acho que eu fui uma mãe melhor graças ao grupo. E assim, todas as gestantes que estão próximas a mim, todo mundo eu recomendo que faça, até as que moram em outras cidades assim eu falo "ah ve se vai continuar no formato online, porque se tu puder participar participa", porque a rede de apoio ali aquelas mulheres que, tanto as profissionais que nos auxiliam que trocam ideia ali, que nos dão conselhos e tal, elas são muito, ai assim elas passam uma confiança, tu vê que sabem do que tão falando, que tem experiência, que tem "know-hall", que tem um olhar assim de empatia com a gente, quanto as mães que tão vivendo a mesma dor, ou que já passaram ou que vão passar por aquilo, aquela troca ali é

muito boa, muito boa assim, foi maravilhoso, um dos presentes da minha gestação foi o grupo. Ai muito bom.

Entrevistadora: Muito legal, não sei se queres falar mais alguma coisa em relação ao teu preparo pro parto…

E3: Ah eu acho assim né, um comentário assim conclusivo, que a minha preparação pro parto também ela foi, logo depois que a [nome da filha] nasceu eu vivi uma frustração, porque eu me preparei muito pra um parto natural, muito, e aí foram muitos meses, todos esse processo que eu te falei, e aí não ter vivido aquilo num primeiro momento eu me senti assim, pô eu não fui capaz, pra mim é triste te dizer que eu sou mãe e eu não sei nem qual é a dor de um trabalho de parto, porque eu não passei né, tive uma contraçãozinha ou outra e tal mas, a maternidade ela ja vem assim avassaladora desde o primeiro momento então assim, é a natureza que decide e a minha natureza naquele momento foi de que a minha filha não tava bem e tinha que nascer, e por sorte, por Deus, pela ciência, por tudo, eu tive oportunidade de que ela nascesse em boas mãos, no momento certo. Então a preparação pro parto é bem importante eu acho, que a gente ter consciência, a gente ter esse empoderamento de que a gente pode estar a frente, tomar as decisões, ele é muito importante, mas entender que imprevistos acontecem e a gente não vai ta no controle de tudo também é importante, e eu levei um tempo pra isso. E eu considero assim, diante de tudo que eu te falei, eu considero que a pandemia teve um efeito negativo assim na minha gravidez porque eu tava bem, tava saudável, tava sob controle, tava bem monitorada e aí assim essa mudança, esse estresse muito grande, toda essa mudança de cenário e essa insegurança que me causou eu acho que potencializaram o meu mal estar da pressão, que resultou no que resultou. Mas assim hoje eu te digo com muita tranquilidade que o importante é a [nome da filha] ta ai, é a minha vida e deu tudo certo graças a Deus. Eu acho que a preparação pro parto ela tem que ser pro melhor, não pro que eu desejo, não pra um formato né, ela tem que ser pro melhor.

Entrevistadora: E chegasse a fazer um plano de parto?

E3: Chegue, cheguei a fazer, fiz junto com a parteira, mas nem precisou né, porque ai tudo mudou porque ai quem ia me acompanhar na indução do trabalho de parto era a [nome da profissional] que dai ja tinha muito a linha do que eu queria, inclusive ela até brincou comigo ela assim “O pessoal aqui da [nome da maternidade] sabe que quando eu vou pra cesárea é só porque realmente precisa, porque não adianta a mãe dizer que quer a cesárea porque eu não vou pra cesárea só se a mãe quer, não vou botar uma mãe numa cirurgia e depois ter que cuidar de um bebê toda cortada porque ela quer, eu só vou se realmente for necessário né”, então assim não… eu acho até que tava acho que a gente levou impresso naquele dia e ficou na mala porque não precisou.

Entrevistadora: E teu companheiro estava junto contigo na hora da cesárea?

E3: Tava, o tempo todo, não, podia entrar comigo, ficou comigo o tempo todo.
finalização

ENTREVISTA 04

Data da entrevista: 27/10/2021
DATA DO PARTO: 25/06/2020

E4: Na verdade o ponto principal dessa questão assim pra mim durante a gestação, tinha o medo né primeiro do vírus em si, tanto pra mim quanto pro bebê, e a segunda coisa que foi assim, que era o baque maior de tudo isso era a questão dos acompanhantes, que foi o que mais me deixava nervosa se eu ia ficar sozinha, se ia poder ficar o meu marido, ai as vezes, “Ah, o hospital tal não ta deixando nem o marido, aí só pode o marido mas não pode doula, aí minha família não vai poder visitar”, daí minha mãe ficou bastante chocada que ela não poderia entrar na maternidade pra me acompanhar, depois pra ver o bebê, depois aquela coisa de quem entra na primeira vez não sai mais e tal… era mais essa questão assim que me preocupava dos acompanhantes né, e o medo assim do vírus de tu pegar ou acabar o bebê pegando, ai ainda muitas notícias assim aleatórias sem comprovação científica, e aí tu ficava meio nervosa assim, né. Mas foram essas duas questões que pegaram mais pra mim durante o parto mesmo né. Aí depois tem outras questões assim de isolamento né, ali no começo de não pode sair, não poder o bebê, a família não pode conhecer, essas coisas que foram bem diferentes né do que é o normal.

Entrevistadora: Uhum, e como é que foi assim pra ti se preparar pro teu parto, como tu imaginava que ia ser e o que tu fazia pra se preparar? Tu lembra?

E4: O que eu fazia pra me preparar… aí fisicamente eu não fazia quase nada, eu só caminhava, caminhava bastante, ficava caminhando caminhando, e aí o próprio, procurar o curso né, já foi também uma, um passo assim pro preparo e fazia muita leitura, muita muita leitura na internet mesmo né, e assistir vídeo, tudo que é possível assim, isso até foi uma questão que a gente acabou ficando em home office e tudo se voltou pra frente de uma tela, então assim, a enxoval, assista vídeo de enxoval, a anestesia, assiste vídeo sobre anestesia, a parto normal, assiste vídeo sobre parto normal (risos). Tudo então foi muita leitura e video na internet mesmo né, aí o grupo também a gente só teve um encontro presencial né, todos os outros foram remotos, também foi muito útil assim e basicamente foi isso assim, e pesquisar sobre maternidade, ficar pesquisando sobre vários temas, eu acho que foi isso assim.

Entrevistadora: No dia do parto dela, tu lembra como é que você sentiu os primeiros sinais do teu corpo?

E4: Ah sim, é na verdade eu ja fiquei assim uns cinco dias sentindo contração só que elas não ritmavam, aí eu, das minhas leituras né, eu sabia que valeria a pena ir pra maternidade só quando elas tivessem ritmadas. Aí então na quarta-feira ela começou assim às seis da noite, começou a dar a contração de 5 em 5 minutos e bastante contração assim, aí foi quando a gente decidiu ir pra maternidade mesmo, foram esses sinais da contração que eu, que na verdade elas pegaram ritmo né, foi o maior sinal assim que eu senti que era hora.

Entrevistadora: E daí depois que tu sentiu que as contrações ritmaram ai tu decidiu ir pra maternidade, ai fosse sozinha, como é que funcionou?

E4: Não, foi eu meu marido e minha mãe, mas a gente ja sabia, a minha mãe foi só pra voltar com o carro né e a gente já sabia que ia poder ficar só nós dois né. Aí eu cheguei lá, aí uma enfermeira fez o toque em mim porque a médica tava numa cesárea né, que eu ia com a plantonista ali do [nome da maternidade] e aí ela me disse que eu tava só com dois dedos de dilatação, aí eu fiquei super desanimada, porque eu tava com muita dor, eu achei assim que eu já tava quase parindo. Aí quando ela falou que eu tava só com dois eu fiquei desanimada até olhei no vidro pra minha mãe e fiz assim [sinal negativo com a mão] vou voltar pra casa de novo. Dai esperei mais um pouco daí a médica veio fez um toque, um toque mais assim invasivo, aí ela não tu já tá com quatro, aí já deu uma animada assim, dai ja era umas oito horas da noite, daí foi indo bem assim, dai dez horas da noite a gente já tinha seis de dilatação aí meia noite eu já tava com oito, aí eu pedi a analgesia, ela me ofereceu eu aceitei e tal, ai quando ela veio fazer um toque também aí estourou a bolsa aí tava com mecônio né, porque a [nome da filha] ja tava de 41 semanas já, aí mas assim, ela falou assim mas não… porque pelas minhas leituras né, eu desanimei na hora, porque eu pensei ah mecônio cesárea de emergência né, na minha cabeça era isso. Aí ela não não, vamo descer lá e vamo ver os batimentos, às vezes dá pra continuar tal, tu já ficou tanto tempo tal, aí a gente desceu mas ela não tava mais legal assim, quando eu tinha uma contração ela não respondia tão bem , aí a gente decidiu ir pra cesárea, era seis horas da manhã de quinta-feira, deu praticamente 12 horas que eu tava lá, aí eu acabei indo pra cesárea.

Entrevistadora: E qual foi a influência do grupo de gestantes nesse teu preparo, nesse teu processo assim?

E4: Ai nossa, esse grupo foi assim, muito maravilhoso! Porque principalmente porque a [nome da filha] ela é a caçula do grupo então assim eu vi todos os relatos possíveis de parto ali no grupo, então assim, ali nas horas das contrações a questão da respiração eu tentava né, isso foi uma coisa que elas me passaram do grupo, tanto as colegas participantes quanto as organizadoras ali né, pra relaxar o máximo possível, a questão da respiração, aí assim logo que lá nasceu né, o meu direito de ficar com ela, de logo ter contato com ela, de botar no peito, tudo isso assim, de várias questões ali que a gente tem direito assim né, ainda ali na sala de parto, de cesárea né pra garantir esses meus direitos que foram elas que… e depois os primeiros cuidados com o bebê também né, questão de cordão umbilical, da higienização, que não era pra usar lenço, era pra usar algodão com aguinha, tudo isso foi do curso assim.

Entrevistadora: Uhum, e como tu estavas se sentindo nesse momento, você se sentiu preparada pro teu parto?

E4: Ai eu me senti assim, preparada, eu acho que sim assim, depois a gente fica pensando se realmente era o caso de cesárea e tal, mas só que na hora realmente assim eu fiquei pensando "não, aconteceu como era o melhor pra acontecer" porque é que daí a gente começa a ler "ah, tem médico que fica levando pra cesárea sem precisar, tem médico que leva pra cesárea" aí

eu fico pensando, será que meu caso era esse né, mas realmente eu vi o cardiotoco não tava certinho tal e enfim, mas a gente fica pensando assim, mas eu me senti segura sim pro primeiro banho pras trocas, as questões com o bebê, claro que tem alguns choros assim que a gente fica nervosa né, muito choro de colicazinha, mas assim, eu sabia o que fazer, uma massagem, botar uma bolsinha quente, também foram dicas ali do curso né.

Entrevistadora: Sim, você conseguiu ir visitar a maternidade?

E4: Não, eles não tavam fazendo visita por causa da pandemia.

Entrevistadora: E achas que isso influenciou de alguma forma?

E4: Não, eu (perda de conexão). As visitas iam fazer eu decidir entre uma ou outra, mas dai não deu pra visitar aí eu optei pelo Ilha, por sei lá, por achar que lá tinha menos cesárea, e bem no fim acabei fazendo cesárea (risos). Ai tudo coisa que agente ouve falar né. Mas as meninas do curso elas fizeram um tour pelo HU, pela maternidade do HU assim filmando, foi muito legal esse, eu não conhecia a maternidade do HU assim, foi bem legal esse vídeo pra conhecer como é que era, toda estrutura, bem legal.

Entrevistadora: Sim, e como é que foi as tuas consultas de pré-natal?

E4: Eu fiz no particular, com a doutora, todo mês eu ia.

Entrevistadora: Teve alguma influência da pandemia, por ter que ser online?

E4: Não, as consultas não, só nesse sentido de acompanhamento né, nem nas consultas e nem no ultrassom eu podia ter acompanhante. Essa questão do ultrassom também foi outro impacto, porque tem toda aquela coisa né, de ouvir coraçãozinho, de ver o bebê e assim, meu marido não pode ver nenhum ultrassom, minha mãe também queria ver algum só depois viam as imagens assim, mas eles não conseguiam me acompanhar, as clínicas não deixavam entrar, nem na recepção assim. Até depois, quando eu tava fazendo aqueles últimos ultrassons com barrigão, que teoricamente eu não poderia nem ta dirigindo, daí meu pai me levava assim e não podia nem subir assim, não podia acompanhamento mesmo.

Entrevistadora: O que achas que mais influenciou na pandemia no teu preparo pro parto?

E4: Ah eu acho que foram mais esses cuidados assim mesmo, que a gente tinha que ter né, e que já desde fazer mala, tipo, ah pega máscara, pega um monte de máscara, pega álcool em gel, pega álcool líquido e não é comum. E tipo, ah mas lá eles devem oferecer, mas a gente não tem certeza, então leva né, aí lá assim, mesmo que a gente saiba que os hospitais tomam todos os cuidados, tudo que eles davam pra gente de alimentação tudo a gente passava álcool em tudo, a gente tinha já esse cuidado de ficar pensando em tudo isso né, desses cuidados sanitários assim.

Entrevistadora: E como tu lidou com tudo isso, na tua cabeça, no teu sentimento de mãe, vivenciando isso, como é que tava tua cabeça nesse momento?

E4: É, na verdade eu acho que assim, eu acho que a ficha não caia, sabe. Porque quando começou em março isso, e daí tipo assim “a daqui dez dias isso vai acabar, né” ai ta “ah não sei o que blá, blá, blá, não pode visita" tipo assim “ah mas o meu é só em junho, até lá já vai ter…” ai derrepente começou “Ah porque o pico vai serem junho” “Ah o pico é maio, junho e julho” aí tu já começa a ficar, meu Deus, eu realmente não vou poder ter ninguém, sabe, a ficha não vai, não vai caindo assim que junho ia ter isso né, ainda. Que tava em março e vinha abril e maio e essa coisa só piorava e assim, a gente ia, a gente na verdade vai se adaptando até o ser humano se adapta né, muito fácil assim, porque se for pensar assim foram muitas mudanças, tudo diferente e hoje olhando pra trás eu vejo assim assim, passou, foi, aconteceu e passou. O enxoval da [nome da filha] foi praticamente todo feito pela internet, eu não ia em loja né, justamente porque grávida era grupo de risco e tal, eu não frequentava loja, não frequentava mercado, foi tudo diferente assim, só que foi, foi e passou e a gente nem… Então tinha todas essas preocupações de tipo “ah, não vai poder ter ninguém la e a gente vai se adaptando a isso né”, ata vai ser só meu marido “ah mas quem sabe até vai ser bom”, aí muitas pessoas até falavam “olha eu acho que tu vai acabar até achando bom porque é um saco tu ta lá na maternidade, toda cansada, toda pós-parto e aquela sala cheia de familiar fofocando e gritando e tu louca pra mandar todo mundo embora”, eu não passei por essa experiência né, era só eu meu marido a bebê e as enfermeiras, então não vou saber como é que é se é ruim também, depois em casa não tinha ninguém lá dando palpite, então tinha alguns pontos assim. Mas passou assim, eu até que considero que eu trabalhei bem a minha cabeça assim, sendo que o tema é pandemia né, se eu for falar o que eu penso do puerpério ai eu não lidei bem tá, eu surtei, mas eu acho que não tem a ver com a pandemia não.

Finalização

ENTREVISTA 05

Data da entrevista: 28/10/2021
DATA DO PARTO: 21/06/2020

E5: Agora tu falando começa a vim na cabeça todas as coisas né, que a gente passou, mas eu lembro que por ser enfermeira eu já tinha algum conhecimento sobre parto, fisiologia do parto, mas o grupo foi fundamental pra eu ter mais conhecimento e poder trocar informações, experiências, então minha preparação basicamente foi nas minhas consultas de pré-natal que eu fiz particular com a médica obstetra que também tem uma enfermeira obstétrica na equipe dela então algumas consultas de pré-natal era com a obstetra e outras com a enfermeira, que a enfermeira me falou mais em relação a amamentação e cuidados com o bebê, né. E o grupo assim foi uma rede de apoio importante, não só nessa parte de conhecimento técnico mesmo, mas de apoio mesmo entre as mulheres, sabe, de saber tipo ai as outras também tinham azia, sabe, os sintomas, de poder compartilhar desses incômodos da gravidez com as outras mulheres, e ver que a gente é normal que às vezes assim, eu como passei pela primeira gestação é tudo muito novo né, a gente apesar de saber que é normal a gente quer assim se identificar com o outro saber que também tem alguém vivenciando aquilo que a gente tá passando, então eu acho que a minha preparação foi mais nesse sentido assim, a parte teórica né, que eu tinha nas consultas pré-natal e essa parte emocional assim com o grupo e é isso e com a minha família também.

Entrevistadora: Uhum, o [nome do filho] nasceu quando?

E5: Ele nasceu dia 21 de junho.

Entrevistadora: Já tava assim naquele auge da pandemia né.

E5: Foi, foi no auge. É eu lembro que algumas semanas, um mês antes dele nascer foi bem na época que as maternidades proibiram a entrada dos acompanhantes, algumas maternidades aqui de Florianópolis, e isso me deu uma ansiedade assim muito grande, porque a gente vem assim de uma vida inteira planejando um parto, pensando que vai ter acompanhante, que vai ter o esposo, a minha mãe eu queria muito que tivesse comigo, então isso foi nossa, um choque muito muito grande de ter a possibilidade de ter que parir sozinha né, com uma equipe, apesar de eu ter parido com uma equipe que eu já conhecia presencialmente, mas de estar num ambiente desconhecido e ainda sem a presença do acompanhante, que eu acho que foi uma das partes fundamentais pra mim. Eu comecei o pré-natal em outubro de 2019, que ainda não era pandemia, ne, e aí eu me mantive no particular mesmo. E aí algumas consultas, por exemplo, ali no fim de 2019 eu fiz algumas consultas presenciais né, com o obstetra e depois quando começou a pandemia as consultas passaram pra online, então eu fui la mais no final mesmo, quando eu tinha que fazer o exame de BCF, o exame físico mesmo. Mas a parte de orientação e exames tudo ela me passava online.

Entrevistadora: Uhum, e além do grupo e da tua leitura e da tua experiência também já como profissional da saúde, teve outra forma que tu te preparou, fisicamente, emocionalmente?

E5: Ai eu fiz tanta coisa, a gente busca muita informação na internet também isso eu não posso negar, principalmente eu sou assim praticamente viciada em internet e instagram, então assim, comecei a seguir muita gente no instagram, de amamentação, de parto, de fisioterapia pélvica e até enxoval, de tudo tudo tudo, a gente fica doidinha assim, começa a procurar informação de tudo quanto é canto, e da internet então. E eu fiz fisioterapia pélvica no meu pré-natal que eu acho que também foi fundamental pra me preparar pro trabalho de parto.

Entrevistadora: Fizesse até o final da gestação a fisioterapia?

E5: Sim, eu comecei a fisio em torno de 25 semanas 27, não lembro direito, e aí foi até o final, até o dia do parto.

Entrevistadora: E no dia do teu parto assim, tu lembras como que foi, como que tu sentiu os primeiros sinais do teu corpo…

E5: É então, o meu parto foi induzido né, alí quando começou alí a 38 semanas eu já queria que entrasse em trabalho de parto né, passou de 37 que eu já sabia que não era mais prematuro eu já queria "ah então tá filho, ta pronto pode nascer, pode vim" e que nada né, caminhando e comendo tâmaras e chá e coisarada pra tentar que entrasse em trabalho de parto mas nada, aí fechou 41 semanas e aí foi um acordo assim com o obstetra, eu e o obstetra a gente achou que era interessante já começar a induzir, ai eu internei com 41 semanas e aí a gente começou a indução, foram usados dois comprimidos de miso e depois começou com a ocitocina intravenosa, e aí foi um trabalho de parto de 16 horas. Aí teve que romper a bolsa no meio do trabalho de parto foi uma, foi artificial né, a bolsa não rompeu sozinha, então eu não sei te dizer sinais de início de trabalho de parto então foi induzido, então não foi em casa nem nada.

Entrevistadora: Uhum, e mesmo nesse contexto de pandemia, tavas se sentindo como, não sei se tivesse a participação do teu companheiro no trabalho de parto, mas como tava tua segurança?

E5: Não, eu tava me sentindo segura até, depois como eu falei ali, tinha essa possibilidade de não poder entrar acompanhante mas depois deu uma amenizada né assim na pandemia, eu lembro que nos hospitais assim foi no início de "ai cancela tudo, ninguém pode entrar, é so gestantes e tal" e depois foi dando uma aliviada "ah pode ficar de máscara" e tal e foram liberando um pouco mais, então eu comecei a ficar mais tranquila. E também pra mim uma lado, eu não posso negar que um lado bom da pandemia foi que eu fiquei afastada do trabalho então, por ser gestante né, como eu trabalho em hospital, então foi um período que eu fiquei bem dedicada assim pra mim, fiquei em casa, convivendo mais com a minha família, então foi um período relativamente bom assim, né, dentro desse contexto louco que a gente viveu

foi um período que eu pude me concentrar mais no meu corpo e perceber as coisa e me dedicar só pra isso, então pra mim foi bom.

Entrevistadora: Que bom que conseguisse tirar um lado positivo da pandemia.

E5: Porque trabalhando a gente fica envolvido e acaba que as coisas vão passando e a parte de família e casa fica de lado mesmo, então eu consegui focar mais nisso mesmo.

Entrevistadora: Sim, em relação ao grupo de gestantes, eu não sei em que momento que tu entrou, no momento da tua gestação, mas qual foi a influência pra ti?

E5: Como assim influência, de eu entrar no grupo?

Entrevistadora: Não, a influencia do grupo no teu preparo pro parto, tu acha que influenciou em alguma coisa, não ter participado ou ter participado?

E5: Ah, eu acho que foi fundamental, se eu não tivesse participado eu estaria bem mais perdida assim. Apesar de eu já, antes de eu entrar no grupo eu já tinha decido assim que ia parir numa clínica particular que eu ia continuar com o pré-natal que eu já tava fazendo mas o grupo foi muito legal, inclusive porque a gente tinha um grupo no Whatsapp depois, então ainda é uma rede de apoio muito muito legal, a gente criou amizade mesmo entre os participantes e me fortaleceu sabe, eu acho que o grupo me fortaleceu pra decisões, até pra compartilhar informações de como é que estão as maternidades e pandemia e sabe, vacina e tudo mais, todas as informações em relação aos bebês e pandemia e tal. Foi bem importante.

Entrevistadora: Chegasse a fazer um plano de parto?

E5: Não, cheguei a fazer um plano formal, porque como te falei que e fiz o pré-natal particular então já sabia mais ou menos a linha de atuação da obstetra, da equipe dela e da enfermeira que participou do parto, então já tinha combinado préviamente como ia ser, então não foi nada escrito assim.

Entrevistadora: E chegasse a conhecer a maternidade antes de ganhar ele?

E5: Não, eu não conhecia.

Entrevistadora: E tu acha que influenciou de alguma forma no teu parto?

E5: Não, na verdade eu não conhecia presencialmente mas nessas de internet eles estavam oferecendo visita virtual, essas coisas, então eu vi algumas fotos de como era o ambiente físico, e fiquei tranquila assim, não me interessei muito também em ver o ambiente físico, acho que não fez muita diferença pra mim.

Entrevistadora: E falasse que se preparasse bastante pro teu parto né, com as leituras, tâmaras, fisio pélvica, e qual tu acha que é a importância do preparo pro parto?

E5: Ai meu Deus eu acho que foi fundamental,acho que precisa muito se preparar, precisa muito muito mesmo, fisicamente essa questão da fisio pélvica foi fundamental pra mim, eu sempre achei assim que eu era uma pessoa que eu tinha uma boa relação com meu corpo de me conhecer e saber sinais assim né, mas a fisio pélvica faz muita diferença, muita diferença, na hora das contrações, na hora do relaxamento do períneo, como fazer a força, posicionamento durante o parto, as posições melhores pro bebê passar no estreito superior no estreito inferior da pelve, foi muito muito muito importante. E a parte mais psicológica assim de, eu usei até um aplicativo pra meditação né durante a gestação que chama "gentle birth" tipo uma hipnoterapia que usa pra hipnose mesmo e que são afirmações positivas e muita conversa com a minha família sobre o que que eu queira do parto, o que eu imaginava, com meu esposo, com minha mãe, né, que eu queria tentar muito o parto normal até quando desse, então foi um acordo mesmo que a gente fez, equipe, eu, meu marido, então foi bem importante essa preparação de planejar mesmo, de planejar como que ia ser essa indução, até quando ia aguentar, como é que ia ser se precisasse de analgesia, todos os passos assim, as possibilidades dentro do trabalho de parto, então isso me deixou muito tranquila na hora.

Entrevistadora: Achas que algumas, no momento do parto, alguma coisa que tu aprendeu lá no grupo de gestantes tu lembrava la no momento do parto, te ajudou ou não?

E5: Específico do grupo de gestantes não porque todo conhecimento que a gente adquire durante a gestação vira uma coisa só, não dá pra dizer que foi só do grupo, claro que o grupo fez parte de todo esse conhecimento, mas não tem uma coisa que eu identifique assim que venha do grupo esse conhecimento mas eu lembro muito das técnicas de alívio da dor que foi uma aula que a gente teve online já, que era sobre as massagens e meu esposo participou dessa aula também e ele se sentiu bem motivado a participar das massagens durante o trabalho de parto, até na gestação a gente treinou depois da aula, foi bem legal assim, acho que mais a parte dele foi bem importante assim, porque eu como tu disse eu já tinha esse conhecimento mais técnico assim né e pra ele participar de todos os encontros foi muito importante. Ele não ia nem nos ultrassons, não ia nem nas consultas de ouvir BCF, era tudo por chamada de vídeo assim, então a parte do grupo pra ele eu acho que foi bem bem importante.

E5: Eu queria só dizer que esses encontros online do grupo que a pandemia trouxe, eles possibilitaram que mais mulheres que não moram em Florianópolis, que não são daqui enfim, que não tem fácil acesso a universidade, participassem do grupo, então eu acho que isso é um ponto positivo de ser online e de estar sendo online, só pra deixar registrado, que talvez seja uma possibilidade daqui pra gente em disponibilizar esses encontros online.

Finalização

ENTREVISTA 06

Data da entrevista: 28/10/2021
DATA DO PARTO: 25/09/2020

E6: O meu parto foi no dia 25 de setembro de 2020 e foi um parto assim, a proposta era ter um parto normal, que foi no HU e eu fui pra lá já tinha, ja tava com alguns dias com contração com os Pródromos e aí eu senti que naquele dia o bebê tava se mexendo menos e aí eu fui pra lá pra poder ver se tava tudo bem, tava aguardando um pouco mais porque eu queria entrar em trabalho de parto, eu queria que as contrações tivessem ritmadas pra poder ir né, por causa da questão da segurança com relação covid mesmo e enfim, o que foi orientado foi isso da gente esperar o ritmo das contrações pra poder ir pro hospital, mas aí naquele dia eu achei que ele tava mexendo menos e eu fui mais cedo, fui umas 11 horas tava tendo contrações mas tava um pouco variado e quando eu cheguei lá a médica achou melhor, achou por bem pela idade gestacional já internar pra fazer a indução, a indução não, esperar o processo. E aí acabou que foi passando o tempo quando chegou lá pelas 20 horas a médica achou que tava muito devagar e não sei se foi o aparelho do coraçãozinho que tava com problema mas eles não tavam conseguindo ouvir direito, toda hora tavam examinando sabe, o tempo todo tavam examinando, foi, aquilo deixou a gente um pouco preocupado né mas realmente ela chegou a fazer o procedimento que é romper a bolsa que ela achou que tava muito devagar o processo e quando ela rompeu a bolsa eu já tava com bastante mecônio né, e aí ela viu que o coração dele deu uma diminuída no batimento e já veio me mandou pra emergência, pra cesárea de emergência, aí foi uma cesárea que a gente fez né, eu tava em sofrimento que eles falam né, quando o coração diminui o batimento, foi isso que ela me explicou e assim aconteceu.

Entrevistadora: E desde a gestação, como é que você pensava no teu parto, como tu desejava, como você se preparou pro parto?

E6: Eu tenho um outro filho de 5 anos e desde que a gente teve ele a gente imaginou que logo, não ia demorar muito pra gente ter outro, mas acabou demorando, levamos quatro anos pra poder engravidar de novo, então, mas quando eu engravidei eu não esperava que eu ia engravidar novamente e aí em janeiro eu soube que eu tava grávida, ficamos muito felizes mas foram três meses intensos assim, passando muito mal e quando foi em março que eu senti que tava melhorando veio a pandemia, sabe. E a questão do parto assim, como o meu primeiro foi uma cesárea também e eu hoje eu percebo que não foi necessária, foi uma cesária muito induzida pelos médicos "porque chegou a idade, já tá na ta hora, não tem porque ficar mais aí, vamo tirar e tal" então foi muito assim nesse sentido, porque esse segundo a gente procurou se preparar melhor, eu me preparei fazendo curso, a gente procurou fazer o curso online e buscar todo tipo de orientação pra que pudesse ser um parto normal né, era o meu desejo assim, era o meu desejo desde que eu soube que eu tava grávida eu falei assim "não dessa vez eu gostaria de ter um parto normal" e foi um processo de aprendizado assim, eu acompanhei muitas lives, participei de grupo de gestantes da UFSC e de outro também, de outro grupo, e procurei orientações a gente fez um curso online como eu ja te

disse sobre o parto normal, todo processo, o que poderia acontecer e o que não poderia, como lidar com as situações mas acabou sendo necessário ter a cesárea né, então não foi bem como a gente imaginou mas deu tudo certo, acho que eu to bem satisfeita porque Graças a Deus ele ta bem, eu to bem, deu tudo certo.

Entrevistadora: Com certeza!

E6: Não sei se eu te respondi…

Entrevistadora: Com certeza, tá ótimo. É bem isso, a tua vivência, a tua experiência né, e mesmo não tendo o desfecho que vocês queriam como é que tu se sentiu assim, tu achas que foi importante ter se preparado mesmo indo pra uma cesárea?

E6: Eu achei muito importante sabe, assim é psicologicamente a gente se viu numa situação totalmente nova né, durante a gestação e eu acho que o fato da gente ter se preparado e ter tido esse contato com pessoas que tavam na mesma situação, ter tido contato com as enfermeiras, com as psicólogas do grupo que nos deram uma acalmada, vai dar tudo certo, enfiam a gente sentiu que tava mais seguro assim, apesar de toda situação que não foi fácil. Eu acho que o desfecho realmente era uma possibilidade sabe, era uma possibilidade que tava clara pra gente desde o início, sabe, poderia ser uma cesárea, então assim, desde sempre todo mundo foi, passou essa informação, não é que você se prepara tanto que vai dar no que você quer, não é assim a gente não sabe como vai ser, então assim apesar do desfecho ter sido uma cesárea eu acho que é isso que me tranquiliza assim, que me deixa feliz sabe.

Entrevistadora: Uhum, e foi permitida a presença do acompanhante no teu parto?

E6: Sim, o meu marido pode ficar até a hora, até o pós-parto assim, até eu ir pro quarto pra…

Entrevistadora: Pro alojamento conjunto

E6: Pro alojamento isso, ele não pode ficar mas durante o parto ele pode ficar, ele que ficou com o bebê logo que o bebê nasceu e aí eu tive que fazer os procedimentos de costurar e tudo, e ele ficou com o bebê o tempo todo, acompanhou todo processo, acompanhou todo procedimento com o bebê assim e isso foi muito bom, coisa que eu não tive no primeiro filho assim, que foi numa outra maternidade que a gente ficou separado, a gente só viu ele depois de algumas horas, eu fui sedada, e lá no HU eu não tive essa questão, não fui sedada, então fiquei o tempo todo acordada, pude acompanhar o tento que eu podia a presença do bebê, a presença do meu marido, então foi muito melhor, muito melhor.

Entrevistadora: Uhum, e qual achas que foi a influência do grupo de gestantes nesse teu processo?

E6: Ah sim, eu acho que foi isso, de dar uma tranquilidade pra gente, de saber, tinhas as informações, as últimas informações com relação o que tava acontecendo, não só com relação

ao que tava ocorrendo com relação covid mas o que tava acontecendo na maternidade e isso foi dando uma certa tranquilidade pra gente de saber que tinha, como é que tava sendo o procedimento com relação às mulhere que tavam tendo o parto naquele momento né, e isso foi, essa notícia sabe, essas notícias que tavam vindo, porque tudo era muito novo naquele momento né, a gente não sabia bem o que era esse vírus, o que podia ocorrer com a gente, principalmente com as gestantes, com o bebê e enfim, era tudo muito, então elas passavam pra gente, e sabendo o que tava acontecendo elas iam passando pra gente, isso nos tranquilizou bastante, sabe. E também outras mulheres né, compartilhar com outras gestantes aquele momento, que foi um momento muito, acho que foi um momento muito único mesmo, porque é você praticamente ter uma gestação e guardar só pra si, é essa sensação que eu tenho, que eu guardei só pra mim e pro meu marido e pro meu filho, assim sabe, parece que ninguém, de repente apareceu um bebê na família assim, na família de meu pai minha mãe, meus irmãos, de repente surgiu um bebê pra eles, porque embora tivesse chamada de video, foto, a convivência não foi possível sabe, a convivência foi muito muito restrita, foi uma vez que eu fui na casa da minha mãe assim, e a gente ficou muito pouco tempo e eu tava de barrigão, então assim, ela não teve esse vivência de me acompanhar, assim também com a minha sogra e de repente sugiu um bebê pra eles, foi bem diferente assim.

Entrevistadora: E no dia do parto, lembras como é que foi?

E6: É eu lembro que eu cheguei la eu ja tava tendo algumas contrações igual eu te falei né, mas fiquei ali no quarto, meu marido pode entrar depois, e aí a medida que eu senti que tava evoluindo, eu senti que as contrações tavam vindo com mais frequência, e senti que tava chegando a hora sabe, até pedi pra tomar um banho pra relaxar um pouco porque eu ja tava ficando um pouco assim, um pouco aflita de, eu não tinha passado pelo parto normal ainda, não sabia como era, e pra mim imaginar era tudo muito, a não sei, meio tenso, como será, será que vai dar tudo certo, será que eu vou conseguir então, tava tudo isso passando pela minha cabeça sabe. Embora eu tivesse uma tranquilidade em saber que as pessoas estavam ali auxiliando eu tinha esse receio assim de saber como seria sabe, e era novo né, então, mas fui muito bem atendida, a presença do meu marido me ajudou muito sabe, é o meu apoio e era isso, fui sentido ai com o banho, o banho parece que deu uma tranquilizada, ajudou, acelerou um pouco mais sabe, as contrações vieram com mais, mas eu acho que a médica achou que não foi o suficiente, ela achou que naquele momento já deveria estar muito mais acelerado e por isso ela pediu que a gente fizesse o procedimento de estourar a bolsa.

Entrevistadora: Aham, e nessa tomada de decisão, ela perguntava pra você e você sabia responder, tinha conhecimento?

E6: Sim, eu lembro muito a questão da ocitocina, ela pegou e falou da ocitocina né, pra poder acelerar, e isso pra mim assim, não sei se foi a visão de outras mulheres que eu absorvi ou se foi uma coisa que realmente foi crescendo dentro de mim, foi um pânico que eu tinha dessa ocitocina sabe, que eu pedi pra ela por favor, isso eu não queria sabe “não dá pra esperar mais? Eu acho que vai dar certo” assim, eu tinha muito receio de aplicar isso porque pelo que me falaram depende da mão sabe, tem mão que acabam colocando mais e a coisa fica absurda

de dor, então eu não sei se é isso mesmo, mas enfim, pra mim os casos que chegaram foi que as mulheres sofreram muito assim, quando foi aplicado essa ocitocina sintética, sintética não sei, não sei se é sintética que fala mas é artificial, sei lá, não sei. Eu sei que eu tinha como referência era o caso de pessoas próximas que passaram por isso e sofreram muito com a questão da ocitocina então ela falou “Ó uma outra opção seria romper a bolsa” e aí uma das enfermeiras até do grupo de gestantes já tinha falado até dessa possibilidade, quando chegasse em determinado momento que precisasse romper que essa era uma forma um pouco mais natural de desenvolver a questão do parto né, então eu falei "ah eu acho que essa pode ser uma boa” sabe, porque eu tava com medo dessa mão, dessa mão misteriosa né (risos), colocar um pouco a mais e eu ficar maluca de dor, porque não é fácil né. É uma loucura, uma dor assim, que não dá pra descrever sabe, essas contrações, aí eu já ficava imaginando isso aumentado, então eu acho que essa decisão sim ela me perguntou se poderia romper a bolsa eu “não, esse é o melhor caminho, vamo por aí", e aí foi feito.

Entrevistadora: Que bom, e lá na sua casa quando foi tomada de decisão, o que que tavas sentindo?

E6: Pois é, eu comecei a ter contrações era terça a noite e foi a noite e foi sexta-feira o parto né, então eu tinha muitas contrações de madrugada e durante o dia elas ficavam espaçadas de hora em hora, assim sabe, eu conseguia descansar, conseguia comer e vinha uma e parava então a decisão foi mesmo assim, vamo esperar vir as contrações mais ritmadas, mas próximas eu acho que de 15 ou de 10 minutos não sei, e quando chegar esse momento a gente vai pro hospital, porque o medo também da covid de pegar sabe, então a gente não queria dar bobeira de ficar ali exposto e como eu entrava no HU eu ficava sozinha ali naquela sala de espera eu ficava muito preocupada, eu fiquei bem neurótica sabe, eu fiquei como já chamam por aí a “louca do álcool” eu ficava com muito medo de pegar, de passar pro bebê, de não saber como seria com ele e comigo. Ai a gente esperou ficar mais próximo, mas quando esse dia amanheceu na sexta-feira no dia 5 eu realmente senti uma, que ele não tava mexendo tanto e foi isso, esse momento que a gente decidiu que, como as contrações já tavam mais próxima e ela não tava mexendo tanto a gente achou por bem que fosse a horra de ir pra maternidade definitivamente pra ver se, sobre uma avaliação, o médico a médica achasse que era a hora sabe.

E6: Eu acho que pela idade gestacional, porque houve um problema ali na questão do primeiro ultrassom, o primeiro ultrassom que eu fiz o médico que me acompanhou não achou muito confiável porque era uma clínica que dava muito problema de detectar o tempo errado então eles acharam melhor ir pelo segundo ultrassom, só que o segundo ultrassom já deu uma data bem diferente, aí quando eu cheguei no HU eles acharam que o primeiro ultrassom é que deveria ter sido considerado então é capaz que eu tenha dado a luz com mais de 42 semanas, com quase 43, porque se for olhar no primeiro ultrassom, só que não sei quanto tempo seria ao certo assim.

Entrevistadora: E vocês chegaram a fazer um plano de parto?

E6: Sim, chegamos a fazer, desde o primeiro a gente já tinha feito, porque no primeiro a gente já tinha feito, porque no primeiro a gente pesquisava, procurava saber, mas não foi uma coisa mais tão profunda como a gente fez dessa vez sabe, porque a gente fez o curso, que a gente acompanhou com as gestantes com o grupo de gestantes, então primeiro a gente, eu pesquisei muito mas não foi realmente uma coisa tão dedicada, eu acho que se eu tivesse me dedicado mais eu teria sabido lidar mais com as pressões né de entrar realmente, de fazer a cesárea por motivo de tempo sabe então eu acho que o segundo a gente tava mais preparado e sabia mais o que dizer no plano de parto sabe. Até teve no grupo de gestantes orientação de como fazer o plano de parto, teve muita informação muito importante, sabe.

Entrevistadora: Que ótimo, e no dia vocês chegaram a utilizar?

E6: Sim sim, a gente já tinha consciência de muita coisa, foi muito importante, muito bom, muito importante pra gente, tranquilizou bastante.

Entrevistadora: E conhecer a maternidade, vocês chegaram a conhecer?

E6: É, no nosso primeiro filho a gente chegou a conhecer, fisicamente né porque foi um outro momento, então a gente pode caminhar lá, na época tava uma reforma que tava tendo então a gente não pode entrar em muitas alas, mas dessa vez a gente não pode fisicamente né, mas eles mandaram fotos, contaram um pouco do que que acontecia em cada lugar, isso ajudou a visualizar assim, imaginar como seria, a e foi bom assim, no final teve, acho que a informação foi suficiente.

Entrevistadora: Das minhas perguntas são isso, se quiseres falar mais alguma coisa.

E6: Acredito que essa coisa da pandemia, acho que a influência é muito mais psicológica eu não sei se as outras gestantes do seu TCC como que você ta direcionando, mas eu acho que essa coisa da pandemia realmente é um fator assim muito, psicologicamente eu digo assim que nos trouxe muito, não sei se as consequências eu vejo assim o grupo, não sei, desculpa, não sei se tem a ver com o que você tá buscando mas assim eu acho que as pessoas ficavam muito mais resguardadas em compartilhar coisas, eu vejo até no grupo, nos grupos que eu participo mesmo as pessoas ficaram muito mais fechadas e lá no grupo de gestantes a coisa nem sempre flui muito bem, assim, até que tem uma informação ou outra quem sabe de alguma coisa fala mas é eu achei que ia ter um entrosamento maior sabe, pelo momento que a gente viveu e tudo, mas eu acho que então acabou dispersando também, como que a vida também pede a gente tem que buscar, tem as coisas pra fazer, tem o bebê pra cuidar, a gente já tem outro filho, mas eu achei que ia ter um entrosamento maior sabe, mas com tudo valeu sabe, foi um momento muito importante que o grupo foi muito necessário.

finalização

Entrevista 07

Data do parto: 07/07/2020

Entrevistadora: Como foi sua preparação para o parto nesse período de pandemia?

E7: O fato da gravidez, por mais que você se prepare e que já tenha ouvido muito de outras experiências, é bem complexo, pois você sabe que a mudança vai acontecer e qualquer mudança tem seu lado "assustador". A tensão já existia, com a pandemia, tentei encarar de forma positiva, consegui trabalhar em home office , consegui acompanhar o grupo de gestantes, que sem a pandemia não teria conseguido. Porém, o contato com a família ficou mais distante e o acompanhamento médico tb, tinha receio de sair e acho que fui um pouco prejudicada pela pandemia nesse sentido.

E7: Da minha parte, tentei me preparar lendo, vendo vídeos, o grupo de gestantes ajudou bastante. Fazia minha atividade física em casa mesmo. Porém em virtude da pandemia acabei não buscando ajuda profissional. Acho essencial uma preparação. São muitos sentimentos e você se sentir entendendo pelo menos o processo ajuda.

Entrevistadora: Qual foi a influência do grupo de gestantes no seu preparo para o parto? E como foi sua experiência de parto?

E7: Com 31 semanas, mesmo com todo meu cuidado, eu tive covid, foi um susto grande. Tive o acolhimento e ajuda da enfermeira e da psicóloga, do grupo de gestantes, que foram essenciais neste período. Fiz meu isolamento de 14 dias e apesar do desconforto, tosse, e cansaço físico e mental elevados, após esse período estava tudo bem. Porém, às 34 semanas minha bebe resolveu nascer. E acredito que o covid tenha sido determinante pra isso.
Eu senti um pouco de dor espaçada e um corrimento anormal, no dia anterior, mas nem imaginava. Porém no dia, logo que acordei as dores estavam mais próximas e intensificando e me assustei quando o corrimento foi sangue. Como estava de 34 semanas, fui na Santa Helena e lá me disseram que já estava 4 de dilatação ao meio dia. Pedi assustada, que fosse parto normal e às 20h ela nasceu de parto normal. Ela nasceu muito saudável, porém devido ao tempo precisou ainda ficar 1 semana na uti neonatal para acompanhamento. O acompanhante eu consegui ter mas era uma preocupação pois estava mudando o protocolo diariamente. O grupo ajudou muito pois pincelaram vários assuntos , pudemos trocar experiências e o que restava dúvida, tinha dicas de onde buscar mais. Pra mim foi essencial.

E7: A uti neonatal foi estressante pois tínhamos que ir pra casa e voltar. Mas no fim das contas, percebi que me ajudou a aprender a prática de tudo e me sentir mais segura com uma vida nos meus cuidados.

finalização

ENTREVISTA 8

Data do parto: 25/04/2020

E8: É, a minha preparação pro parto foi bem antes da pandemia né, porque quando começou a pandemia eu já tava com 8 meses de gravidez e eu me preparei muito assim, eu fiz, estudei tudo que eu podia, li vários livros, eu tinha doula e fiz toda a preparação física, mental e psicológica que eu podia fazer, me preparando pra essa questão da maternidade. Minha gravidez foi completamente planejada né, a gente, eu realmente decidi para com o anticoncepcional e a gente tava tentando, a gente tentou por uns dois mesinhos e eu já fiquei grávida, então a gente fez bastante essa preparação, o que eu tinha mente eu sempre quis um parto domiciliar e eu tinha uma amiga da minha doula, ela era médica, ela é médica e a gente tinha planejado de fazer o parto aqui em casa na água né, que era o que eu desejava fazer então esse foi o meu planejamento, um pouco antes da, ai logo em seguida teve a questão da pandemia que foi uma coisa bem complicada mas eu fiquei tranquila porque eu já tinha planejado de fazer o parto em casa então não tinha essa coisa do hospital, não tinha que ir pro hospital, e aí eu tive alguns percalços bem sérios assim, primeiro foi porque essa médica falou que ela descobriu que não poderia fazer o parto porque os médicos perderam, enfim, teve uma resolução que os médicos não poderiam fazer o parto, tinha que ser uma enfermeira obstétrica enfim, e ela poderia perder o registro dela então ela não pode fazer e aí um pouquinho antes da pandemia começar e toda essa coisa assim, uns dois meses antes de eu, do parto eu tive que trocar pra uma parteira que eu não queria na verdade que é uma parteira mais tradicional, que é uma senhorinha que chama Marisa e eu já tinha ouvido algumas coisas em relação a ela mas eu não tava muito a vontade assim do que eu tinha ouvido dela mas eu aceitei porque eu realmente não queria ir pro hospital, não queria fazer um parto no hospital, e aí logo em seguida também teve a pandemia então eu achei que o ideal seria continuar com ela.

E8: Enfim, quando eu tava com 36 semanas ali oito meses, começou a pandemia, um pouquinho antes né, foi um mês antes do meu parto e aí eu fiquei bem nervosa por causa disso, de receber as pessoas aqui em casa e toda essa coisa né mas, enfim, quando eu entrei em trabalho de parto foi no dia 23 de abril, numa quinta a noite, e aí eu entrei em trabalho de parto, chamei minha doula e a parteira chegaram e mais uma amiga minha que eu chamei pra me ajudar, estava meu marido aqui, a gente tava já se preparando já tinha comprado tudo que tinha que comprar e tudo que tinha que, tava tudo a preparação já pronta pra ter esse parto em casa e 12 horas depois eu já tava com a dilatação completa mas a minha filha não encaixava de jeito nenhum então eu continuei tentando um parto ativo eu fiquei dois dias inteiros num parto ativo quando chegou no sábado de manhã eu não aguentava mais, não tinha mais força, não conseguia mais comer e não conseguia descansar por causa das contrações que não paravam e aí eu decidi ir pro hospital, cheguei la no HU fiquei mais a tarde lá de novo, dilatação diminuía depois voltou e aí eu comecei a sentir uma dor muito forte, muito forte, que não era de contração, era uma dor num lugar específico da minha barriga e eles faziam exame de toque o tempo todo e tavam o tempo todo visualizando onde ela tava e eu não

aguentava mais e aí eu tive que ir pra uma cesárea de emergência e aí eu fui pra cesárea e ela nasceu e ela ao contrário, ela tava com a cabeça pra baixo mas ela tava virada pra frente ´né, e não virada pra trás, então ela tava numa posição que seria muito difícil fazer um parto normal e isso foi muito traumático porque além de todo cansaço e de toda idealização que eu tive, de toda preparação que eu tive, tanto no grupo de gestantes né, que a gente continuou conversando, quanto na questão da minha preparação de antes de tudo que eu fiz, tinha o medo dessa questão da pandemia, a minha doula não pode entrar tinha toda essa questão de medo e eu não conseguia ficar muito tempo de máscara né, tava tudo começando então a gente tava com muito medo.

Entrevistadora: Você conseguiu identificar os sinais do seu corpo?

E8: Sim, eu tava tendo contrações de treino a muito tempo, já umas semanas, e aí eu percebi a diferença, eu tava bem ligada assim, porque a minha filha nasceu com 41 semanas e 1 dia então já tinha passado da data provável de parto então eu tava bem atenta pra ver o que que ia acontecer né, quando que ia acontecer eu já tava bem esperando. Em relação ao grupo de gestantes, foi bem legal, a gente teve só um encontro presencial infelizmente porque logo em seguida teve já a pandemia, ma so grupo de whatsapp virou uma rede de apoio muito boa, a gente conversa e principalmente no começo, a gente conversa até hoje bastante assim, pra trocar ideias e falar sobre as coisas e tirar dúvidas e desabafar, fiz umas amizades muito boas ali, principalmente no começo quando a gente é mãe de primeira viagem a gente acha que tudo tem que ir pro hospital e essa era a parte mais difícil porque às vezes eu ficava pensando "será que realmente eu preciso ir pro hospital e não to indo e aí tem essa coisa da pandemia, eu posso ir lá e pegar covid" então era uma das coisas mais absurdas assim, mas do medo era de não ir no posto ou não ir no médico com medo da pandemia e achar que tava negligenciando algum cuidado que realmente precisasse né, um cuidado médico, porque a gente não sabe né, criança chora o tempo todo, bebê chora o tempo todo quando é pequeno e a gente sempre acha que tem alguma coisa de errado o tempo todo então eu acho que essa foi a parte mais complicada assim no começo.

finalização

Entrevista 09

Data do parto: 31/07/2020

Entrevistadora: Como era pra você desde a gestação quando você projetava o seu parto como aconteceu esse processo de espera? Quais eram seus sentimentos?

E9: Eu estudei sobre parto durante a gestação e fiz exercícios pensando em um parto normal, mas sabendo que não sou eu quem controla tudo para escolher isso, ou seja, poderia ser de outro jeito e estava tudo bem. Quando estava com 31 semanas de gestação foi descoberto um problema no coração do bebê e, inicialmente um parto normal estava fora de questão, talvez fosse até necessário fazer uma cesariana prematura. Me decepcionei um pouco, mas estava mais preocupada é se ficaria tudo bem com minha filha. O problema dela evoluiu bem, não precisou ser um parto prematuro nem agendado e perto de nascer a cardiologista fetal liberou parto normal desde que não ficasse muito tempo em trabalho de parto. Só que aí eu mesma fiquei com medo de essa decisão prejudicar minha filha e fiquei em dúvida do que fazer. Minha médica me passou bastante segurança e deixamos para decidir na hora. Acabou sendo um lindo parto normal.

Entrevistadora: Pensando no contexto de pandemia, como foi para você a preparação para o parto?

E9: A preparação, como falei na pergunta anterior, foi com estudo e relato de uma amiga que já teve duas bebês por parto normal. Conversei com ela para saber bem como era e tirar minhas dúvidas. A pandemia, nesse caso, só mudou o fato de a conversa ser online e não pessoalmente. Além disso meu marido estava se cuidando muito para não pegar covid para não me passar, claro, mas também para poder acompanhar o parto.

Entrevistadora: Você consegue lembrar do dia do seu parto, como foi que aconteceu desde o início? Como você lidou com esse momento? Você conseguia identificar os sinais do seu corpo?

E9: Sim, consigo. 3h15 da madrugada levantei pra fazer xixi e não consegui segurar, escorreu pelas pernas. Achei estranho aquilo, porque tava conseguindo segurar até em espirro e tal. Mas ok. Aí deitei de novo e vi que tava escorrendo mais líquido. Aí que me toquei que talvez não fosse xixi. Levantei de novo, escorreu mais. Aí acordei o [nome do companheiro]. Não sabia se já ligava pra obstetra, afinal vai que era só xixi mesmo, era pouco que escorria assim, não um monte que eu imaginava que seria quando rompesse a bolsa. O [nome do companheiro] falou pra ligar e nesse momento senti uma dor na barriga, mas não vindo das costas, como sempre falam que é a dor das contrações, só na frente mesmo. A obstetra pediu pra contar de quanto em quanto tempo eu estava sentindo essa dor e irmos nos arrumando pra maternidade que ela ia avaliar o que tava acontecendo. Nos arrumamos e nisso só escorria mais líquido e as dores já estavam de 2min e meio em 2min e meio. Pensei, acho que vai nascer mesmo, mas se isso é contração é muito tranquilo!

Chegamos na maternidade já estava doendo um pouco mais cada contração e tava com 2cm de dilatação. Pensei vixe... podia estar com mais já. E a bolsa realmente rompeu mas foi saindo aos poucos o líquido porque a bebê ainda estava bem alta, segundo a médica.
[...] Coração ótimo, fomos pra sala de parto normal. Contrações já doendo mais, mas eu ainda pensando... ah, da pra aguentar várias horas disso. Um tempo depois a médica viu a dilatação, estava em 3cm. Nessa hora pensei vixi... assim tá muito devagar. Será que aguento tanto tempo?! Tomei um banho quente e as contrações resolveram apertar e ficar realmente doloridas. Falei pro [nome do companheiro] que se fosse cesárea eu tava bem tranquila com isso e ele me lembrou que eu queria parto normal e ia dar tudo certo [...]. Fui pra bola me mexer um pouco como tinha ensaiado com a fisioterapeuta no pilates, mas foi ficando muito difícil. Vomitei, sentia que ia desmaiar a cada contração. A médica dizendo que já já ia pra analgesia, mas esse momento nunca chegava. Aí examinou de novo, eu pensei, se a dilatação não tiver evoluído vai ser cesárea e deu. Estava com 6cm! Agora a médica liberou a anestesia. Saindo da sala de anestesia já estava com 8cm. Sentia só a minha barriga se contrair e mais nada. Que paraíso! Consegui comer e fazer bastante exercício na bola e caminhada pra ela descer, porque apesar da boa dilatação a bebê ainda não tinha descido. Comecei a sentir uma pressão embaixo, tipo uma vontade de fazer cocô mesmo. Partiu escolher onde eu ia querer a parte final. Já tinha sentado no banquinho durante as contrações e não tinha gostado. E não sabia se o [nome do companheiro] ia ter força pra me segurar muito tempo. Primeiro parto... isso poderia durar umas 2hrs. Fiquei na maca. A médica e o [nome do companheiro] já foram montando o apoio pro pé e um negócio pra eu me puxar com o braço e ajudar a fazer força. A pressão embaixo aumentando. Médica olhou, já estava com dilatação total. Agora era só fazer força. Como estava com analgesia, não sentia a dor da contração, só a bebê descendo. Não era uma sensação agradável, mas também não tão ruim assim. Fiz bastante força. Vi nessa hora que ter feito exercício físico a gestação inteira até o final e trabalhado perna, braço e a musculatura da região pélvica ajudou demais. Cada força que eu fazia ela descia bem. Tanto que não chegou a durar 1 hora essa parte. Nos 20 minutos finais que foi mais desagradável porque eu já estava com as forças esgotando e pensei de novo que talvez não desse conta. Mas a médica falou que já via os cabelinhos dela e eu sabia que nesse momento não tinha mais como "engolir" o bebê de volta e ir pra cesárea, então tirei força do além e coloquei ela pra fora. Também pensei que precisava ajudar muito ela por causa do coração que se manteve super bem o parto todo, mas, eu sabia que, especialmente pra ela, ficar muito tempo "entalada" ali não era legal. E ela saiu. A sensação é um alívio profundo assim e pra mim felicidade por apesar de todos os percalços da gestação. Corona, trabalho estressante, o coração dela, ter conseguido manter os exercícios, o coração dela deixado e ela ter nascido quando quis da forma mais natural que consegui!

Entrevistadora: O que significou participar do grupo de gestantes em relação à sua preparação para o parto?

E9: O grupo foi mais uma fonte de conhecimento onde pude conhecer e tirar dúvidas sobre os momentos, os mecanismos... foi onde mais detalhadamente ouvi sobre todas as fases e pude fazer meu marido ouvir também para estar preparado.
finalização

ENTREVISTA 10

Data da entrevista: 01/11/2021
DATA DO PARTO: 30/03/2020

E10: Bom a gestação foi bem tranquila, durante todo período da gestação, a gente não teve nenhum tipo de intercorrência, foi uma gestação bem saudável, bem tranquila mesmo. De preparação pro parto, eu e meu marido a gente sempre leu muito, a gente buscou também algumas fontes confiáveis, algum material do IFSC e também depois o material que a gente recebeu, material de suporte do HU também, nós fizemos parte também do grupo de gestantes lá no IFSC que daí foi antes da pandemia né, e quando a gente tava começando a gente fez um encontro presencial com o HU né, e aí depois a gente precisou interromper porque a gente entrou em lockdown, a gente não pode dar continuidade. Eu senti bastante falta desse material, na verdade dessa troca que acho que poderia ser muito rica né, a gente fez alguns encontros online mas eu não pude participar de todos porque a minha bebê nasceu prematura, então com 34 semanas ela nasceu e aí o pessoal ainda tava fazendo os encontros e eu já tava ali na, no meio do "vuco vuco". Então não deu pra aproveitar muito assim, dos encontros né, mas do grupo de gestantes nossa, foi muito bom depois porque tava todo mundo no mesmo barco né e então teve uma troca, uma partilha muito grande, em relação ao parto foi assim que a gente se preparou, eu vi outros relatos também, outras partilhas. Ela nasceu no dia no dia 30 de março, então nós estávamos há duas semanas em lockdown, em casa, absolutamente em casa e foi um período assim muito incerto né, porque a gente tem uma expectativa que vai acontecer algumas coisa mas geralmente acontece tudo, na verdade sempre acontece como o bebê quer né, então foi bem inesperado assim a prematuridade dela, a gente já tinha assim algumas coisas preparadas mas a gente não tinha bolsa de maternidade pronta, a gente não tinha nada pronto né e a gente foi pra maternidade, a gente foi pro Ilha porque a gente achou que podia ser só uma intercorrência ou algo assim né, a bolsa estourou e eu não sabia se era realmente a bolsa estourando, se era tampão ou sei lá o que, a gente foi pra checar na maternidade e aí quando chegou lá a médica analisou, avaliou e "não, a bolsa estourou, vamo ficar pra ver se entra em trabalho de parto" e logo em seguida a gente já deu continuidade no trabalho de parto já evoluiu e ela nasceu 9h30 da manhã, a gente chegou a bolsa estourou era próximo da meia noite e ela nasceu de parto natural às 9 e pouco da manhã. Mas toda essa informação a gente pode colocar em prática, muita muita muita informação que a gente tinha, com relação a posição, com relação a não entrar em desespero quando a bolsa estoura né, se manter mais centrado durante o trabalho de parto também, tudo isso foi muito bom, tanto pra mim quanto pro meu marido, e relacionando à pandemia a parte difícil veio depois assim, porque nós ficamos 15 dias no hospital, a gente ficou cinco dias primeiro a bebê foi pra UTI neo e a gente ficou 5 dias e depois a gente, naquele período ainda não tava obrigatório o uso de máscara, mas então já se ouvia falar de alguns poucos casos de covid, um ou dois casos no Estado, aquela coisa muito ainda incerta assim, a gente não sabia o quão próximo a doença estava da gente né, e aí a bebê ficou 15 dias no hospital de só depois disso ela saiu, e aí foi assim que ela nasceu.

Entrevistadora: Teu marido pode te acompanhar no parto no começo da pandemia?

E10: Acompanhou, isso, mas uma semana antes eu acho, a gente teve aquele decreto que não podia entrar doula né, a doula enfim, um terceiro acompanhante, segundo acompanhante, mas meu marido pode me acompanhar sim, ele ainda pode me acompanhar, algumas do grupo acho que ainda tiveram que ir sozinha, não sei, não sei como é que foi, mas ele pode ir nesse começo, fazia muito pouco tempo que a gente já tava em lockdown então ele pode me acompanhar durante esse período, ele ficou durante todo período, inclusive depois quando a bebê ela voltou pra, saiu da UTI e foi pro quarto, eu fiquei com ela, internada com ela e a gente deu um jeito do meu marido também ficar pra dar um suporte, a maternidade foi bem receptiva assim, eles fizeram meio que uma, sei lá uma vista grossa e deixaram ele lá comigo, ainda foi possível.

Entrevistadora: Que ótimo! Lembras o que fizestes pra se preparar fisicamente e psicologicamente?

E10: Ah sim, bom durante toda a minha gestação eu me preparei, eu fiz o curso, eu treinei durante toda minha gestação, já treinava antes então continuei treinando até a gente entrar em lockdown eu continuei treinando assim, a minha alimentação também, fazia monitoramento com o nutricionista então tava tudo bem certinho é, eu tava bem relaxada assim, mentalmente também, eu meditava algumas vezes e conversava muito com a [nome da filha] também, lia algumas coisas, alguns livros sobre criação de filhos que ajudaram bastante, então esse foi assim um preparo do meu corpo para o momento ali, é um trabalho mental bem intenso, eu quis desistir em alguns momentos mas é bom que a dor vem, a dor vai e a gente logo muda de ideia, então foi mais tranquilo. Mas o que mais eu lembro de ter aproveitado nos encontros as meninas explicando sobre a fisiologia do parto e sobre as posições, o que a gente poderia optar né, elas falaram da posição de quatro apoios que era uma posição muito adequada que facilitava muito, mas que as mulheres hoje em dia tem optado bastante pela cócoras pelo banquinho e tudo e aí na hora que eu fui pra sala de parto o médico perguntou, ele ofereceu o banquinho e aí eu disse “não, eu vou primeiro tentar então ficar de quatro apoios” e aí isso foi assim uma das informações que deu super certo pra mim, porque então eu fiquei de quatro apoios estava super relaxada, meu marido tava ali comigo e aí eu me senti bem confiante nessa posição e ela nasceu assim. A gente teve laceração de grau 1 não precisou dar ponto, não precisou de nada, foi bem tranquilo, então essas informações foram muito úteis que eu consegui colocar em prática né durante o trabalho de parto eu fui pro chuveiro em alguns momentos, foi um trabalho de parto bem tranquilo, eu fiquei a maior parte do tempo deitada, tentando dormir e aí eu fui pro chuveiro, fiquei um tempo no chuveiro, aliviou bastante também pedi pra ele fazer massagem na região da lombar, ajudou bastante também, ele foi meu “doulo” né. É e daí foi assim que eu pude aproveitar das informações.

Entrevistadora: Chegasse a fazer plano de parto?

E10: Cheguei, cheguei a fazer mas não deu tempo nem de imprimir, não deu mas a gente já tinha assim tudo muito claro né, do que a gente queria e do que a gente não queria, é uma

coisa muito louca porque assim, porque quando chega na hora também a gente se abre pra todas as opções porque a gente começa a vivenciar aquilo de uma forma que ok se a gente se prepara, eu me preparei né, pra um parto natural mas ok se tiver que vir uma anestesia, se tiver que vir uma interferência enfim pra acelerar o parto enfim, a gente se abre pra isso também, porque a gente sabe o que ta acontecendo com o corpo né. Mas enfim, não precisou, a gente não precisou de ocitocina, não precisou induzir, foi bem tranquilo, não precisou anestesia também, mas a gente sabia desses recursos né, por conta do preparo do plano né, a gente se informou disso.

Entrevistadora: E vocês já conheciam a equipe que estavam com vocês nesse momento ou não?

E10: Não, a gente não conhecia, umas duas semanas antes eu tinha combinado com uma enfermeira obstetra pra me acompanhar, mas eu não tinha acertado nada com ela ainda, faltava fazer contrato essas coisas todas, mas a gente já tinha conversado, e aí depois quando a [nome da filha] nasceu a gente não deu continuidade, o médico eu fui com o médico do plantão, então toda equipe foi a equipe plantonista assim, não tive contato com ninguém antes. Eu tive um médico que me acompanhou durante o pré-natal, a gente fez no particular e a gente optou por não fazer o chamado, não chamar ele e fazer o atendimento no plantão né.

Entrevistadora: É eu ia te perguntar agora como é que foi tuas consultas de pré-natal, mas já falasse que foi um acompanhamento na rede particular.

E10: Não não, não teve nenhum problema, meu marido conseguiu ir em todas as consultas, a gente assistiu todos os ultrassons, não teve problema não. Era uma coisa muito recente ainda quando a [nome da filha] nasceu. Agora depois que ela nasceu a gente sentiu muito os efeitos da pandemia porque a gente optou por ficar realmente bem distante dos nossos familiares né, então eles visitaram a bebê ela já tinha quase um mês, um pouco mais assim, porque a gente não sabia exatamente como o que a gente tava lidando então a gente optou por isso, a gente optou por esperar um pouquinho mais pra eles nos visitarem e aí isso foi bem doloroso pra gente porque a gente ficou sem suporte, sem rede de apoio, e a parte que eu senti mais dificuldade foi na amamentação, porque não deu da gente estudar muito antes, é e aí a gente, mas se virou.

Entrevistadora: Como era teu sentimento em relação a isso, como imaginavas, já tinhas tido alguma outra experiência?

E10: Não, a [nome da filha] é minha primeira filha, na verdade aconteceu tudo exatamente do jeito que eu esperava, quando eu penso na parte técnica do negócio sabe, tipo eu não queria induzir, achava que não era legal o uso da ocitocina, achava que queria o parto totalmente natural e tudo mais, e essas coisas todas aconteceram né, mas o andamento da coisa, de como o cenário inteiro ele muda daquilo que a gente visualiza, daí as minhas expectativas eram essas, eu não sabia se eu ia pro Ilha, eu não sabia ainda se eu ia pro HU eu ainda tava em dúvida, então ficou uma coisa assim que a gente ia decidir na hora dependendo como fosse, a

gente ia contratar essa enfermeira obstetra pra ficar com agente pra gente ficar o máximo de tempo em casa, ficar mais tranquilo em casa durante o trabalho de parto e tudo mais, não sabia quanto tempo ia levar mas, no geral aconteceu tudo dentro do esperado né.

Entrevistadora: Sim, foi muito bom assim vocês terem conseguido levar de uma forma tão leve né, apesar de que tenha sido inesperado, mas deu tudo certo, vocês estão bem e saudáveis. Das minhas perguntas são essas, só queria te perguntar também qual que achas que foi a importância de vocês terem se preparado pro parto?

E10: Ah foi essencial, nossa foi muito importante, muito importante, claro, a gente só começou a estudar quando a gente recebeu o positivo, a [nome da filha] veio de uma FIV então a gente recebeu o positivo, a gente esperou pra receber o positivo primeiro né, a gente já veio de um tratamento e tudo mais, e aí e a gente recebeu o positivo e começou a estudar as pensar nessas possibilidades do parto, mas foi assim primordial porque sem informação acho que tudo seria muito mais difícil, do ponto de vista de conseguir contornar as dores, de conseguir se manter estável sabe e até depois, talvez se eu tivesse optado por uma cesárea eu não tivesse conseguido dar tanta assistência pra ela né, então, então foi nossa muito bom, primordial.

finalização

ENTREVISTA 11

Data da entrevista: 04/11/2021
DATA DO PARTO: 09/10/2020

Entrevistadora: Desde a gravidez, como foi sua preparação pro parto, como você se preparou?

E11: Eu acho que acabou sendo mais difícil, pelo fato de estar na pandemia, porque já começa por consulta, consulta já era mais difícil, tinha lugares que não atendia ainda bem no comecinho da pandemia foi quando eu descobri minha gravidez então eu queria logo fazer a ultrassom, queria logo começar o pré-natal e ninguém tava atendendo, o posto não atendia, dizia que não tinha nem previsão pra isso, foi bem difícil assim.sabe e daí nas consultas também não podia ter acompanhante, meu marido queria participar mas não tinha como. Eu acho que pelo fato da gente ter se isolado também dificultou um pouco, porque a é um turbilhão de emoções na gravidez né e tu tá ali meio sem ninguém, tu ta totalmente fora da tua rotina, nossa passa um monte de coisa pela cabeça, e eu fiquei muito pra baixo no começo da gravidez e eu acho que isso influenciou muito. Até porque eu tinha uma visão diferente de quando eu tivesse grávida, de coisas que eu queria fazer de sabe, de estar na minha rotina de barrigão sabe, eu dançava por exemplo então eu queria ta fazendo dança com barrigão e não aconteceu nada disso sabe. Eu acho que nessa parte assim foi mais complicado, sabe.

Entrevistadora: E você lembra assim o que você fazia, se chegasse a fazer algo específico pra te preparar, seja fisicamente ou psicologicamente pro teu parto?

E11: O que eu fiz foi estudar bastante o assunto, tipo o grupo em si me ajudou demais, demais mesmo, coisas que eu nem imaginava sabe falaram lá e material que foi enviado tudo, eu acho que o meu principal a minha principal preparação foi isso e daí por fora eu tentava pesquisar um pouco, tentava manter a calma, não pensar que nem todo mundo pensa que "ah vai ser horrível, não vou aguentar, não sei o que" eu já penso o contrário, eu penso "não o corpo foi feito pra isso, então vai dar tudo certo" era esse o tipo de coisa que eu fazia mais pra me preparar sabe.

Entrevistadora: E como é que foi o dia do teu parto, você lembra os primeiros sinais?

E11: Sim, eu lavente 5 da manhã pra fazer xixi e a bolsa tinha estourado, eu nem tinha percebido que era, eu demorei ainda pra entender que era a bolsa daí a gente foi já pro hospital e foi bem tranquilo sabe, foi com calma eu ainda acordei meu marido e ele falou assim "como que tu me acorda com essa calma" que eu cutuquei e falei assim "olha eu acho que estourou a bolsa, mas eu não tenho certeza" e ele já deu um pulo, já começou, enfim. E eu na maior tranquilidade do mundo, e aí chegando lá daí foi mais complicado né, porque já tava com bastante contração, evoluiu muito rápido só que dai quando era pra ele sair ele não saia, então demorou muito por causa disso, então eu achei a questão do parto eu achei muito difícil, o que tranquilizou foi que pelo menos ele pode estar comigo do lado na hora do parto,

então ajudou porque tranquilizava porque tinha massagem enfim, tudo isso né. Aí foi, em partes foi horrível, em partes foi maravilhoso, depois que ele nasceu eu esqueci de tudo na mesma hora.

Entrevistadora: E falasse que teu companheiro te ajudou com massagens, vocês já conversaram sobre isso desde a gestação, como foi isso?

E11: Sim, e tudo isso ajudou pela questão do grupo de gestantes porque daí eles mandavam material, explicavam como fazer inúmeras coisas e ele sempre participava comigo então ele tava ciente de tudo. Tinha coisas que eu nem lembrava e ele vinha puxar tipo "ah não, lembra que foi falado isso" e daí essa parte ajudou muito assim, porque por exemplo massagem essas coisas eu não pesquisava, posição de segurar coisa aqui na barriga de lençol, toalha enfim, alguma coisa pra diminuir o peso, eu nunca tinha visto isso e foi a coisa que mandaram e ajudou muito sabe então, foi mais parte do grupo mesmo que veio isso.

Entrevistadora: E chegasse a fazer um plano de parto, como foi?

E11: Não, não, eu meio que deixei pra o que tivesse que ser, como fosse na hora e nem idealizei muita coisa também, porque é uma coisa que não tem como prevenir nada, não tem como deixar nada tão certo então eu ia acabar ficando frustrada se eu fizesse de um jeito e não acontecesse então nem planejei nada disso.

Entrevistadora: E a maternidade vocês chegaram a conhecer antes de ganhar ele?

E11: Não, por questão da pandemia. A gente queria muito mas não teve como, então também foi coisa de na hora saber, dai na hora enviaram um vídeo mostrando um vídeo de como era, mas pra mim não mudou muita coisa porque chegando lá foi tudo novo, então eu fiquei meia perdida. Que a ideia também era ter mostrado, ter visitado aliás, pra conhecer pra saber onde era, onde tinha que ir, enfim, daí só fiquei um pouco perdida na hora de ir lá.

Entrevistadora: E na hora das massagens, vocês lembravam de outros métodos pra alívio da dor, na hora do teu parto?

E11: Olha, até sim mas meio que apagou da minha memória o que aconteceu lá, então era muita coisa pra pouco tempo, tava muito exausta e meio que apagou assim, eu lembro que teve bastante massagem nas minhas costas, eu ia pro chuveiro, tinha, aí pior que essa parte eu não lembro muito sabe, eu acho que posições de ficar meio abaixada também ajudou, é o que eu consigo lembrar aqui agora. Não lembro de muita coisa, porque até ele me lembrava de coisas que pra mim não tinha acontecido lá, se ele tivesse aqui ele ia poder te falar mas eu não lembro desses detalhes.

Entrevistadora: Não, tranquilo, é o que você lembrar mesmo. E o que significou pra você ter participado do grupo de gestantes, nesse teu processo?

E11: Nossa, eu não sei nem te dizer porque eu sou muito grata sabe, porque realmente ajudou muito, foi meu principal apoio, do geral, porque ensinaram a dar banho no bebê, ensinaram muita coisa que eu não sabia, de massagem no peito de não deixar empedrar, porque era uma coisa que eu tinha muito medo, da amamentação, porque todo mundo falava "ah, empedrou, que doía, isso, aquilo" e gente, eu não passei por nada disso, não vou dizer que não passei mas quando ia acontecer inclusive eu lembrava de coisas que tinham ensinado, e até num dia que eu passei perrengue, nossa eu chorei dai no mesmo dia mandaram mensagem do HU perguntando se tava tudo bem, agora eu não lembro de que parte que era, mas perguntando questão de amamentação e por coincidência foi no dia que eu tava precisando de ajuda sabe. Nossa o grupo pra mim foi muito muito importante mesmo, eu inclusive até indico, todas as minhas amigas engravidaram, muitas eu não sei se acham que é besteira mas é que realmente pra mim foi muito bom, muito bom mesmo.

Entrevistadora: Tinhas muita consciência corporal por ja dançar antes né.

E11: Eu sabia de cada detalhe que tava acontecendo no meu corpo, era até meio chato assim, mas eu acho que é isso assim, não tenha nada muito específico pra te falar.

*finalização**

ENTREVISTA 12
Data da entrevista: 10/11/2021

DATA DO PARTO: 27/05/2020

E12: Eu tenho grupo até hoje, a gente conversa, formamos uma grande rede de apoio, eu sinceramente ja to um pouco cansada de tanta informação de maternidade e puerpério, neném assim, é um assunto que chega uma hora que eu preciso dar umas pausas mas é um grupo que desde o começo a mulherada engrenou, e se fala tudo ali, desde onde você compra um bode até como é tua sexualidade com teu parceiro.

Entrevistadora: Sim, e assim, qualquer momento, se tu não se sentir confortável, isso eu falo pra todas as mulheres né, se tu não se sentir confortável e quiser que eu interrompa a entrevista e não utilize os dados, isso é muito aberto assim, ta bom?

E12: Ta bom.

Entrevistadora: O intuito é realmente ser uma conversa, de forma leve, não tem um roteiro pronto né, porque cada mulher tem sua individualidade, então geralmente eu vou seguindo assim. Não sei se queres começar contando desde a tua gestação como foi o teu preparo pro parto, como que tu imaginava o teu parto…

E12: Ta bom. Bom, na real eu comecei a ter vontade de ser mãe a partir dos 30 anos mais ou menos, 31 anos, antes eu sempre falava que eu não queria ser mãe, que eu não queria ser mãe, que eu não queria ser mãe e naquela época eu tava fazendo uma pós-graduação em psicologia corporal e aí a gente já começou a ver bastante coisa, em relação a gestação, em relação à maternidade, e aí eu comecei a me interessar e já comecei a pensar numas coisas, eu trabalho com o corpo né, então pra mim toda essa região do períneo, de toda essa preparação que a mulher faz eu já tinha feito muito trabalho porque eu sou professora de canto e a gente trabalha muito com essa musculatura, e eu já tinha ido numa fisioterapia pélvica. E daí quando eu engravidei, bom os primeiros quatro meses eu só vomitei assim que foi, tipo foi terrível, não conseguia fazer nada, tava escrevendo TCC nem conseguia escrever e depois, fazia pouco tempo que eu morava aqui em floripa então eu não conhecia muito mas eu fui pesquisando assim a respeito do parto humanizado, eu tava muito afim de parir e eu tava muito afim de parir com esse lugar de respeito, de carinho, de cuidado, surgiu pela minha cabeça fazer um parto em casa então eu visitei o grupo “humanascer” mas vinha o medo de a ter que ir pra neonatologia e sair de casa correndo, recém-parida com o bebê… aí decidimos fazer no HU, ai eu não tinha plano de saúde, eu comecei a fazer tudo pelo SUS e achei ótimo e referências do HU cada dia eram melhores, e eu comecei a assistir partos e só chorava, chorava, chorava, e eu comecei a ler algumas coisas sobre, livro sobre parto, sobre essa questão do parto ativo, eu lembro que eu li o livro “parto ativo”, li um livro sobre a “eutonia do parto” que é um livro que foi escrito por uma argentina sobre o trabalho da eutonia no

processo de gestação e de parto, a questão da vocalização no parto, enfim, e essa coisa do parto humanizado, e meu companheiro foi junto também a gente fez um curso pra pais sobre gestação, parto e puerpério, que era pra homens, mas eu acompanhei ele algumas vezes, a gente leu todos os processos do parto, nossa cara eu me preparei tanto pro parto que deveria ter me preparado pro que vinha depois, que é muito mais difícil. Todo mundo fala “ai se preparem pro, pra amamentação, pra isso…” mas meu, tu tá grávida lá, voando numa nuvem de corações, com a libido super alta, com os hormônios super, e você tá lá naquele momento super, que na real, realmente, é um momento muito forte né o parto, e aí eu falei “[nome do companheiro] vamos se inscrever…”, meu companheiro, “vamos se inscrever no…”, eu sou uma pessoa muito agilizada, que estuda, que gosta de saber o que tava, então eu estudei muito, eu li muita coisa, mas principalmente eu já tinha um trabalho de consciência corporal muito grande, então todo mundo me falava “nossa você vai parir fácil, você tem quadril largo, então você vai parir fácil” e eu falava “hmmm, não deve ser isso, não deve ser isso”, mas enfim dai eu me inscrevi pro grupo do HU porque eu queria conhecer o HU e eu queria fazer esse grupo que tinham me falado que era muito bom, então a gente tinha dois cursos, o curso dos pais, do [nome do companheiro], e o curso do HU, só que o curso do HU durou um encontro, e depois era assim, passando informações, algumas coisas e tal e daí o que aconteceu, daí começou tudo o negócio da pandemia, isso que era em março, eu ia ia parir em maio e eu fiquei apavorada, e daí eu olhei pro meu companheiro e falei “Eu não vou parir no hospital”, porque naquela época já estava se falando que não podia ter acompanhante, foi bem no começo, então assim, foi um pânico, eu lembro que ele saia pro mercado parecia um extraterrestre assim, era tipo um negócio muito louco, aí eu falei assim “não, eu sozinha, parindo no hospital no meio de uma pandemia, não” e daí a gente decidiu ter um parto em casa. E dai eu me lembrei a [nome da profissional], uma parteira reconhecida mundialmente assim, ela viaja pela Europa, pelo mundo, ela é mexicana, ela mora aqui, tem até na Netflix que tem uns documentários de parto que a gente assistiu, e ela tá ali, e ela não sei uma mulher… pesquisa sobre ela porque ela é uma mulher incrível, e ela é famosa assim, qualquer pessoa que fale de parto domiciliar, fala dela, é tipo uma figura muito conhecida. Quando a gente foi no grupo humanascer ela tinha falado que em maio ela ia viajar, então eu falei cara, a [nome da profissional] não vai nem estar pra essa essa data do parto, e eu tinha medo do parto domiciliar e eu falei não, aí eu me lembrei que claro, começou a pandemia maio ela não vai viajar, ai eu me comuniquei com ela e já, ela fez um encontro por zoom e já topou. Então a gente fez um parto domiciliar planejado, que nesse momento foi a melhor decisão que eu consegui tomar e realmente foi maravilhoso, foi um trabalho muito, muito, muito, com muita dedicação, com muito carinho, com muito respeito, com muito profissionalismo. Ela e mais uma enfermeira obstetra me acompanharam, a gente fazia uma vez por semana um pré-natal com elas, elas ensinaram o meu marido a escutar o batimento cardíaco da [nome da filha], eu também fazia acompanhamento pelo SUS, mas elas nos deram muita autonomia pra entender todo processo que tava acontecendo, elas nos ensinaram a sentir a posição da [nome da filha] na minha barriga, o meu marido marcava os batimentos cardíacos e ela ia contando, foi um processo maravilhoso, ela ensinou ele a entender como ela tava posicionada e fazer o desenho na barriga, do meu bebê, que ficou lindo, e depois a gente teve só um encontro presencial, elas tinham sumamente cuidado por causa da pandemia, então foi tudo muito, muito, muito cuidado mesmo e a gente se encontrou toda semana até o dia do parto. E o dia do parto foi

aqui em casa. A gestação foi tudo certo assim, a única coisa que eu tive foi muitos vômitos no primeiro trimestre e muita azia no último, muita azia no último mas assim, minha gestação foi linda sabe. E daí, numa terça-feira de madrugada eu comecei a sentir assim o que chama de pródromos né, eu comecei a sentir os pródromos e daí meio dia saiu meu tampão mucoso e esse dia eu tinha consulta no SUS então eu fui lá e elas falaram "ah, se não é hoje é amanhã, que possivelmente é amanhã, tá começando", tinha algumas contrações mas não era muito fortes e eram bem espaçadas, beleza daí passaram as contrações tu vê, quarta de madrugada comecei a ter contrações fortes e seguidas umas das outras, não era mega fortes mas ja sentia mais e eram mais seguidas, dai a gente chamou a [nome das profissionais], e elas vieram aqui umas três horas da manhã, duas horas da manhã, não me lembro, mas eu tava conversando né assim, eu tava bem, ai ela falou "Olha eu acho que ta bem no início mas mesmo assim vamos fazer um tato pra ver como que ta" e ela fez o toque eu eu tava com dois centímetros de dilatação dai ela falou "O melhor que tu pode fazer agora é descansar" mas ai eu "Ah mas ta doendo como é que eu vou fazer" e ela falou "Não, entra na banheira, entra no chuveiro, quando você sentir muita contração você entra no chuveiro, se você ainda não entrou em trabalho de parto ativo mesmo né se ta nesse período que chama, como é que é o nome, de latente uma coisa assim" ó nem me lembro "entra no chuveiro que vai passar a contração e você volta a dormir, você dorme, dorme, dorme" e assim foi eu dormi, eu entrava no chuveiro tãnãnã, passava um pouco e ia tentava dormir, assim numa postura meio esquisita mas tentava dormir, e assim nesse processo de manhã também eu comecei já perto do meio dia ir muito no banheiro, ir muito no banheiro, fiz uma limpa assim, o pouco que eu tinha comido vomitei dai meu companheiro me deu açaí e eu vomitei, daí lá pela uma da tarde começaram as contrações assim violentas que eu falei pro meu companheiro assim "vou entrar na banheira, pra ver se passa" elas falaram assim, se não passa na água é porque você já tá num período mais avançado e você vai saber quando tem que chamar a gente e dai uma hora ou duas horas da tarde eu falei "Chama as parteiras!" e dai eu ja entrei na partolândia né, e dai eu ja tava um bichinho, ai foi isso mesmo, eu fui um bicho, eu lembro que entre as contrações eu dormia assim, capotava dormia e quando elas chegaram, chegaram era três da tarde mais ou menos e eu tava na banheira, com contrações muito fortes, berrando, berrando, cada contração assim foi um berro que eu nunca senti na minha vida, eu trabalho com a voz e foi um berro insólito assim, e elas aí me jogando água mas muito muito, assim, principalmente a [nome da profissional], ela tinha um papel ativo, mas ao mesmo tempo não, ao mesmo tempo era muito assim me deixar à vontade, mas aí quando ela fez o tato eu já tava com nove, dez centímetros de dilatação, primeiro eu fiz o tato quando elas chegaram eu já tava com oito ou sete acho e aí depois ela fez um tato de novo eu já tava com dez era tipo cinco da tarde só que na banheira eu não conseguia fazer força, dai ela começou a falar "ah vem aqui, sai da banheira, talvez te ajude, vai ser mais rápido o expulsivo aqui fora, que tem mais terra, eu vou poder te ajudar" e eu não queria sair da banheira falava "não não" eu tinha medo, tinha medo de ficar em pé, sei lá, dai ela me falou de novo e eu fui, e elas tem um puff assim, é um puff que você fica meio acocorada no puff sabe, e aí eu fui lá pro puff e aí ela começou a passar uns óleos no meu períneo a fazer uma massagem o tempo inteiro, o tempo inteiro, e daí ela me falava pra abrir as pernas e eu lembro que eu fechava as pernas ela me falava pra eu abrir as pernas e eu fechava as pernas, dai meu marido numa hora falou "Amor, abre as pernas" eu falei "Cala a boca", tadinho levou uma assim, mas ele foi ótimo super me

acompanhou, meu a gente ficou sozinho praticamente o maior tempo de trabalho de parto, porque elas chegaram às três da tarde e eu pari as seis. E daí o expulsivo foi a parte mais difícil, mais medo da né, de se quebrar em cinco mil pedaços, eu tinha pavor assim e eu fazia força eu ficava com minha mão assim na vagina e eu sentia a cabecinha vindo e daí quando eu parava de fazer força ela meio que volta, ela meio que faz esse movimento natural mas eu falava “não, volta não, fica aqui pelo amor de Deus” e eu olhava pra parteiras e falava “ela ta voltando” (risos), nossa me dava um desespero, essa parte foi a mais crítica assim, mas a [nome da profissional] cara foi uma mulher assim fundamental, principalmente nessa hora do expulsivo, que ela me explicava como fazer a força e passava, me massageava o períneo, ela me deu a mão pra eu me jogar no abismo, ela falava assim “Ta doendo? Vaai! Ta sentindo que queima? Vaaai!” eu lembro que eu falava assim “Essa mulher ta doida”, mas enfim era confiar ou arrebentar, e aí eu confiei e a [nome da filha] nasceu, eu peguei a [nome da filha] quando ela nasceu ela falou “Pega com as duas mãos” e eu peguei a [nome da filha] nasceu ótima, ela foi avaliada com… como que é esse nome de avaliação na maternidade?

Eu: É o apgar.

E12: Isso, dez e dez, ela tava, ela nasceu rosinha, chorando, berrando, nasceu ótima assim, nasceu super bem, já foi no peito na hora assim, minha placenta nasceu cinco minutos depois, muito rápido. Não lacerei, não tive laceração, o que eu tive foi um pequeno corte mas assim, sabe quando você abre a boca muito e faz um, mas que não precisava de ponto, só que foi numa veia então não precisava de ponto, então ela parou assim o sangue e tal, mas assim foi tudo certo e daí a gente ficou ali com o bebê, com a placenta, dai depois de bastante tempo meu marido foi cortou o cordão da [nome da filha], elas pesaram ela, mas não fizeram mais nada nela, eu tinha feito já os exames pra ver seu eu tinha algum tipo de, se tinha que colocar colírio ou não sabe, elas fizeram eu fazer todos os exames, então não precisou de nada assim, elas me deram um shake pra mim de açaí com banana e placenta que eu topei comer e eu comi, e uma sopa deliciosa, esperaram até eu fazer xixi, me falaram pra fazer xixi tãnãnã, o que eu tive que eu não sabia que poderia ter era tipo uma espécie de hemorroida interna que me deu, que me doeu muito, o pós-parto os primeiros dez dias assim nossa cara, sentar era tipo um horror assim e toda essa sensação que fica, a barriga e tal, mas foi muito tranquilo elas ficaram até a noite e elas ficam tipo de plantão e tal e no outro dia vieram e ficaram praticamente o dia inteiro assim, me ajudando com a questão da amamentação falando da questão da amamentação, daí fizeram todos os dados da [nome da filha], botaram o pezinho, cortaram as unhas, enfim, um monte de coisas assim, e no outro dia também quando desceu o leite elas vieram também me aconselharam, elas vieram mais umas quatro ou cinco vezes depois do parto, foi um acompanhamento muito bom.

Entrevistadora: Que legal, pelo que tu falas assim de toda a sua preparação, na verdade já tens uma experiência muito grande de consciência corporal né.

E12: É, eu estudei teatro e música, mas eu fiz depois vários cursos sobre educação somática, que tem a ver com trabalhos de consciência corporal, pilates, yoga, eutônia, alexander, são

todos trabalhos assim com o corpo e depois eu fiz uma pós-graduação de três anos em psicologia corporal onde também trabalha bastante o corpo e as emoções.

Entrevistadora: E qual achas que foi a influência do grupo de gestantes no teu preparo pro parto?

E12: No preparo eu acho que não tanto assim porque a gente, a gente trocava alguns livros né, mas a gente era mais as coisas do enxoval sabe, do que vai precisando, alguns vídeos sobre aleitamento, mas estávamos todas muito na expectativa, eu lembro que cada vez que nascia uma criança era aquele relato de trabalho de parto, que às vezes dava algum medo né, porque tinham uns relatos que meu Deus (risos). É porque teve umas meninas que tentaram parto em casa e não rolou, dai deu aquela, bateu aquele medo assim né, mas a gente se conversava bastante assim, eu acho que o grupo foi muito mais importante pra mim no pós-parto, pra mim assim marcou mais, claro que antes o parto foi bom por exemplo eu peguei uma banheirinha que tinha doado por uma mãe, pra pegar uma banheira aqui, a gente se doava roupas e coisas, a gente tava, mas assim tava naquela ansiedade do parto mas cara, quando mais você precisa de uma rede de apoio mesmo é quando o bebê nasce, quando tu tá o "pó da gaita", cansada, estressada, com medos né, e aí se formou uma rede muito legal, o que eu achei que faltou um grupo de pais né, porque o grupo era pra mães e pais né, era pra casais na real né, e entendo que talvez no grupo de whatsapp não estejam os pais juntos, porque tem um monte de conversações das mulheres que não vão se sentir à vontade talvez com os homens juntos, mas talvez ter feito um grupo pra pais porque eu acho que eles precisam disso sabe, eles precisam também de troca e precisam de incentivo pra isso porque os homens tem muito mais dificuldade, então nesse aspecto eu acho que os pais não tiveram essa rede que a gente teve, essa oportunidade de rede, a gente depois nós mulheres tentou criar os grupos de pais mas não rolou mas foi um tempo depois.

E12: Então no nosso grupo éramos só um grupo no whatsapp de mães só um grupo de mães, não teve grupo de pais e isso achei meio estranho, porque o grupo originalmente era pro casal né, mas eu entendo que foi bem no começo da pandemia que foi tudo meio caótico, e é isso eu acho que o grupo pra mim de whatsapp que até hoje a gente se fala, minha filha ja ta com um ano e meio praticamente, meu Deus como passa rápido, mas é uma rede até hoje, hoje eu já tô um pouco cansada, mas assim é uma rede importante, pra tudo, pra tudo assim, não só pra coisas de bebês, às vezes a gente conversa sobre outras questões e é importante, porque o que mais faltou nessa questão da pandemia foi isso, não ter uma rede de apoio, assim como pais de primeira viagem e é muito difícil, talvez nos primeiros meses não porque a gente tá muito assim né, nós três estávamos aqui, foi até legal que não veio gente visitar, que eu nem queria mesmo que viesse muita gente assim, mas depois foi muito duro, foi muito duro, meu pai morreu de Covid na Argentina e teve uma mãe do grupo que veio pra cá no meio da pandemia pra me trazer um bolinho, me dar um abraço sabe, então realmente assim o grupo pra mim no pós-parto foi muito importante, a gente compartilhou muita coisa e compartilha até hoje, quando o bebê ta doente todo mundo manda força então, se criou, claro que não são todas que estavam antes, não são todas as que ficam constantemente falando mas são bastante, somos umas 15 mães que estamos aí conversando eu ultimamente converso menos

mas eu to meio assim, com o mundo cibernético já meio cansado assim, preciso de relações ao vivo assim sabe, mas pra mim foi muito importante nesse pós-parto. Porque é isso a gente se prepara muito pro parto mas o que vem depois meo, é muito intenso, muito difícil, e você está num grau de vulnerabilidade gigantesco assim, então, esses apoios assim que eu acho que tanto mais como pais tem que ter e eu acho que hoje em dia dimensiono muito mais como que as mães precisam de cuidados gigantes nesses primeiros meses assim e que eu não tinha pensado, hoje por exemplo to lendo um livro que fala sobre o quarto trimestre, que fala sobre isso, os cuidados no pós-parto, que eu acho que é tanto quanto importante que uma mãe que se prepara para o parto, principalmente para as mulheres entenderem que tem que ser cuidadas, que eu acho que isso na mulher é uma coisa que, como a mulher cuida sempre, não tem muito cabimento, a gente não dimensiona o quanto a gente precisa de cuidado nesse momento, cuidado com o bebê a gente que vai fazer, porque o bebê precisa da mãe isso é claríssimo, não precisa de um pai, talvez você troque todas as fraldas mas ter o pai ou quem estiver ai cuidando, tem que te levar água, fazer uma massagem, tem que pegar o bebê enquanto ele dorme pra você dormir, tem que fazer as coisas da casa, tem que te dar carinho, tem que te dar um abraço quando você chorar né, esse lugar é fundamental e os pais também não sabem cuidar do jeito, não dimensionam, porque os homens da nossa cultura eles não estão acostumados ao lugar do cuidado, assim como a mulher não tá acostumada ao lugar de receber, então nesse sentido eu penso que seria muito importante nessa preparação da gestação colocar muito ênfase nisso, o papel de cada um, a mãe vai cuidar do filho, o pai vai cuidar da mãe e é isso, o pai vai ter que sustender, sustentar esse lugar por muito tempo, porque também as pessoas acham que o puerpério dura quarenta dias e meo eu to saindo mais ou menos agora do meu puerpério, então às vezes a gente acha que uma viagem de 200km e não se prepara pra uma viagem de 3500km, se você já sabe que a viagem vai durar 3500km mesmo que seja uma viagem longa, você já vai psicologicamente mais preparado pra isso, mas não, até na questão de recuperação física do períneo mesmo, não sei a gente espirra e faz xixi, coisa que antes não acontecia, na relação sexual doi, é um lugar que fica dolorido por muito tempo, muito tempo mesmo e os homens tem que entender isso, a libido muda muito você amamentando se você quiser seguir uma amamentação prolongada o que que vai ter de consequência, tipo sabe, um monte de questões que está relacionada com o pós-parto que assim, o parto tudo bem a gente se prepara eu acho ótimo que tenha toda essa questão do arto humanizado, da mulher se preparar mas assim passou, o parto vai passa e o que vem depois é pra vida toda e é um lugar que nós enquanto sociedade não estamos preparados pra isso, em outras sociedades a mulher ficava com outras mulheres que cuidava da mulher, que tem esse lugar maternal, então a mãe precisa ser maternada nesse momento, essa é a palavra, a mãe precisa de alguém que materne ela pra ela poder maternar solidamente seu bebê, porque a gente tá quebrada mesmo, a gente tá renascendo, a gente teve um bebê junto assim , então eu acho que, eu por exemplo eu sou Argentina e nunca senti tanta vontade de estar perto da minha família assim, sabe de ter a minha mãe do lado que eu não tive, de minhas tias, todo esse mundo de mulheres assim, por isso o grupo foi tão importante, ter essas mulheres do lado, por que os homens é um outro lugar e a gente precisa dessa ressonância com outras mulheres que passaram por isso, e os homens aprenderem muito mais a cuidar e abrir mão dos próprios cuidados, abri um pouco mão por um tempo das coisas pra ele, de abrir mãe da questão do sexo, aprender que a sexualidade pode ir pra um outro lugar, porque quanto mais

eles cuidam dessa criança, dessa mãe, menos eles vão obssecados se querem transar ou o que for, então eu acho que pra mim eu tinha lido, tinha falado mas a gente não dimensioan eu acho o quanto isso é dificil e o quanto é difícil resgatar o casamento, o quanto que é difícil trazer de novo esse mundo do casal, principalmente da mulher de voltar e o quanto que o homem pode chamar carinhosamente pra esse lugar de novo, porque a gente entra, se você entra no puerpério e entra nessa simbiose meo, dura muito tempo, a parada é muito forte. Não sei se eu to falando demais, mas você pode me parar.

Entrevistadora: Eu te agradeço pelo teu tempo, se tu achares que lembra de mais alguma coisa, eu estou aberta pra isso.

finalização

ENTREVISTA 13

Data da entrevista: 10/11/2021
DATA DO PARTO: 15/12/2020

E13: O meu parto aconteceu no dia 15 de dezembro, do ano passado de 2020, eu já, como a minha empresa tem convênio eu utilizei o convênio pra fazer o parto. Eu entrei em trabalho de parto no dia 14, meu tampão estourou no dia 14 de manhã, fui fazer consulta com o médico e ela disse que tava tudo bem, que não ia evoluir tão rápido, que eu podia ficar tranquila, fui pra casa da minha mãe almoçar bem de boa, e as contrações começaram a acelerar, então o negócio começou a pegar, não tive estouro de bolsa nada nesse momento, aí fui pra casa comecei a acompanhar de acordo com as orientações do curso que a gente fez né, pra acompanhar o ritmo, o tempo das contrações, utilizei um aplicativo porque na hora a gente não consegue pensar e contar, não existe isso, é muito difícil e o aplicativo dava essas orientações, chega um certo momento que elas tavam próximas e ritmadas que ele dizia "vai pra maternidade, vai pra maternidade" mais ou menos assim, mas eu fiquei segurando porque era bem suportável assim as contrações, não era nada absurdo. Aí fui pra maternidade isso já no final do dia lá pelas cinco horas da tarde mas eu já tava meio desconfiada que não tava ainda, sabe, que podia esperar um pouco mais, mas como a gente vai nessa tecnologia, o aplicativo pedia pra ir pra maternidade falei "ah então vou né" sei lá, foi meu primeiro parto, ai fui fiz o exame lá eu tava com 1 centímetro de dilatação e aí mas o médico já falou que pelo ritmo assim que ele já tava acompanhando realmente eu já estava em trabalho de parto e ele já ia me internar, internar e aí começou a evoluir bem rápido assim, às dez da noite estourou a bolsa, aí realmente evoluiu, passou pra seis de dilatação e aí assim a [nome da maternidade] tava bem lotada e eu não tava na sala de parto eu tava num quarto nesse momento sabe, aí como eu queria parto normal né, eles tentaram achar uma sala de parto, não tinha disponível aí eu fui pro centro cirúrgico sentar no chão pra tentar fazer o parto, só que isso a gente vai ficando tensa, eu confesso que eu fui ficando bem tensa porque não tinha nenhuma acessoria assim, era eu e meu marido e a equipe de enfermagem me acompanhando e tal, e aí fui aquilo ali me deixou bastante tensa em virtude da pandemia, foi então assim, eu tinha muita neura da sujeira do chão, dessas coisas na minha casa eu cuidava muito, imagina eu tentar ter um parto no chão do centro cirúrgico então eu tava assim bem bem nervosa, mas tá fui evoluindo evoluindo, aí depois de um certo horário lá pela uma da manhã uma enfermeira falou que ia tentar liberar uma sala de parto pra mim porque a pessoa que tava tentando disse que relaxou ia dormir enfim não ia evoluir o parto aí fomos pra sala de parto e lá começou realmente a, as dores começaram a, a não, lá embaixo quando eu tava lá embaixo eles me ofereceram a analgesia, eu acho que tudo pela minha tensão e tudo mais, até então eu não ia aceitar mas acabei aceitando, muito pelo meu nervosismo da situação, daí cessou as dores só que dai eu não conseguia fazer com que elas evoluíssem né, daí fui pra sala de parto ela foi voltando gradativamente o ritmo das contrações e tudo mais, enfim assim, acho que até às três e meia da manhã eu cheguei a nove de dilatação, nisso tava rolando um outro parto normal um ou dois ao mesmo tempo só tinha um médico obstetra na sala ao lado, e eu fui ficando muito tensa, ficando mais tensa ainda né, pandemia e tudo mais e aquilo ali foi me

deixando super nervosa, eu já não sabia mais trabalhar com a dor, fazer a força e tudo mais, trabalhei várias posições e nada nada e nada e chegou uma hora que eu gritei “Eu quero que alguém faça uma cesárea!” mesmo com nove de dilatação, daí o médico correu né porque foram chamar ele, porque o bebê nasceu ele foi ali e fez os pontos tal, e a outra pessoa gritando mas mesmo assim ele foi ali pra me atender ai ele falou “Não, agora é muito tarde tu podes ter uma hemorragia, etcetera, etcetera" aí eu falei “Então o senhor tem que me ajudar de alguma forma porque eu não sei mais o que fazer, eu ja to no meu nível aqui de estresse máximo” se com nove eu pensei poxa, se eu já tô a tanto tempo nesse ritmo eu não to conseguindo parir, alguma coisa pode dar de errado, tava com muito medo, a pandemia deixa a gente bem enlouquecida assim sabe, daí eu tomei a decisão eu falei “Não, muito tempo cara, eu não sei mais o que fazer” eu não sabia mais que movimento fazer pra me ajudar a ter o parto normal, e eu não sentia ela encaixar ali pra descer, cara eles falavam com nove “Ah tu vai entrar no expulsivo, tu ta no expulsivo, a gente tá vendo" eu falei “Mas não vai entrar nunca? Eu já to cansada” e chega uma hora que tu não aguenta mais, a dor lá no final ela é bem punk, só que assim eu sentia dor e eu não sabia fazer a força, se eu soubesse fazer a força eu acho que eu iria finalizar tranquilamente, mas o meu emocional por eu estar sozinha, sem nenhum apoio, meu marido não sabia como me ajudar, o médico mandando fazer força de cocô, ridículo assim que a gente sabe que não é assim, e eu falei e eu gritei “Eu não faço força pra fazer cocô” eu gritei na hora, porque eu fiquei muito chateada assim, porque a gente sabe que pô isso é padrão falar e ele não é mulher ele não sentiu, ele não sabe, o que que ele ta falando, e aí todas as enfermeiras eram novas, elas não tinham experiência de parir, elas tinham experiência de parto, isso elas tinham, mas não de parir, então eu acabei ficando muito nervosa, e a pandemia foi algo que me deixou, foi a gota d'água porque eu sempre me cuidei bastante na pandemia, a gestação foi uma gestação muito desejada e tudo mais então ela me deixou bastante tensa assim, aí eu optei por fazer a cesárea, fomos pro centro cirúrgico, fiz a cesárea e de tão cansada de tanta força que eu fiz ela nasceu, minha bebê nasceu foi aquela emoção eu vi e apaguei, eu cheguei a roncar na mesa assim, apaguei de tão exausta e eu não tive aquela hora mágica, dourada e tal, contato com ela pele a pele e tal, não tive, fui ter na sala de recuperação e eles colocando ela ali mas eu não tinha força nem pra segurar, eu não conseguia, meu marido ficou ali tentando e ele ficou com ela, a gente tentou dar o peito amamentar ali mas eu realmente não tinha força e meu marido graças a Deus ficou com ela, daí depois fui pro quarto, ai depois sim, ai eu fui voltando, fui recuperando as energias, devo ter tirado um cochilo de sei lá, quarenta minutos assim, aí seguiu aí voltei, mas essa é minha experiência de parto.

Entrevistadora: E em relação ao grupo de gestantes, qual tu achas que foi a influência do grupo nesse teu preparo pro parto?

E13: Então assim, como eu não fiz atividade, com relação às informações foi muito válido, muito, super válido assim, essa questão de controlar contrações, saber até que ponto eu poderia ir, no plano de parto eu não fiz plano de parto mas na minha cabeça eu sabia falar lá o que eu queria e o que eu não queria então assim, o pós o nascimento, o banho os cuidados com o bebê, depois com ela em casa, eu anotei, muitas das aulas eu fiz várias anotações né, dai recapitulei dias antes e assim foi super válido porque né, o cuidado com o banho, umbigo,

como fazer o charutinho, etcetera, etcetera, etcetera, foi bem importante. Apesar de que o charutinho eu esqueci nos primeiros dias, então ela tinha muito reflexo de Moro então ela acordava e eu nossa, eu chegava a dormir grudada nela pra ver se ela reduzia, ai depois que eu fui lembrar "Nossa, mas tem o charutinho" (risos) e aí fui fazer. Mas super importante porque a gente é mãe de primeira viagem e a gente "Ah a gente tem contato com bebê" mas é diferente do nosso, então se a gente vai visitar alguém que tem bebê é muito diferente, tu só vai ali, tem contato rápido e depois tu volta, quando tu tem filho é 24 horas, depende exclusivamente da gente, então as aulas são muito válidas, todo conteúdo super importante, pra mim foi ótimo.

Entrevistadora: Faltou alguma coisa no teu preparo?

E13: É eu acho que faltou esmiuçar melhor o momento ali do parto, porque até exercícios que a gente poderia fazer para facilitar o parto né, então depois eu sabia porque alguém falou "nossa mas tem um movimento ali dos pés que tu vai abrir ou fechar o teu quadril pra facilitar a passagem do bebê e tal" e eu não sabia fazer isso, então acho que essas coisa são muito importantes, eu acho que facilita, porque hoje tem doulas, tem maternidades que realmente tem muita experiência que acompanha e tudo mais mas ali comigo não tinha e nem podia ter, era proibido ter qualquer outro tipo de acompanhante então assim, eu tava no escuro, eu tava pensando minha natureza vai me ajudar, mas cara se alguém te der uma orientação ali facilita, é menos sofrimento pra mãe, pro bebê, acho que as coisas vão ser mais rápidas talvez, não sei, e assim, preparação eu digo desde os movimentos, até a roupa assim sabe, porque a gente podia ficar de top mas tem mãe que fica sem porque elas, na maternidade elas não te orientam com relação a isso, elas só falam que tu tem que tirar a roupa e deu, e eu queria ficar de top porque ela nasceu no inverno e era muito frio, então eu queria sei lá, se eu posso ter um jaleco, eu não sabia de nada disso, também ninguém orientava e foi uma semana anterior a neve que teve então, imagina, claro que ali no calor a gente vai trabalhando e não percebe tanto mas são coisas assim mínimas que a gente não sabe assim, pode tomar água ou não pode tomar água? pode comer ou não pode comer? eu não sabia, eu não sabia de nada disso, claro eles me davam uma água de vez em quando mas eu também não tinha essa instrução, será que é bom ou ruim? Esses detalhes são bem válidos, acho que bem importantes, pra gente ter segurança. E outra coisa, o médico realmente não vai fazer o parto, ele vai assistir e tal, mas deixar a mãe tranquila, ele vai ali ele vai te orientar, nesse sentido, elas falam mas eu acho que na aula pode esmiuçar um pouco mais o momento do parto, acho que seria bem válido.

Entrevistadora: Sim, e como é que foi assim pro teu companheiro ele conseguiu participar desse momento?

E13: Sim, eu pedia pra ele fazer pressão né na minha bacia porque isso ajuda bastante realmente a aliviar as dores da contração, porque por mais que seja rápido mas aquele minuto ele é muito intenso, ele fazia esse movimento me ajudava bastante, e ele conseguia me ajudar super bem ele no dia seguinte a gente fez uma troca, a maternidade autorizou a fazer uma troca de acompanhante porque ele tem trombose nas pernas e aí por ele ter ficado tantas horas

fazendo força me ajudando ele ficou muito ruim, mas ele me acompanhou todos os momentos, tentou me acalmar e ele disse que quando eu tomei a decisão de pedir a cesárea ele deu graças a Deus porque ele falou que por ele ele ja tá teria pedido muito tempo antes né, porque pra ele com certeza deve ser uma visão, a gente tá ali no calor da emoção não se percebe né, mas ele disse nossa, ele ficou muito chocado.

Entrevistadora: Achas que teria sido diferente se tu tivesse feito uma preparação diferente na gravidez, o que tu achas em relação a isso da tua decisão?

E13: Com certeza, com certeza, tanto físico quanto fisioterápica né, fsioterapeuta tudo mais isso ia ajudar sem dúvidas assim, visto que eu não fazia nenhuma preparação porque de novo, eu tinha medo por causa da pandemia, eu tinha medo de sair e aí na aula talvez elas pudessem ter acalmado a gente também, claro que é uma decisão muito pessoal. a pandemia também é muito nova, todo mundo tinha medo e tudo mais, mas pra gente decidir e pesar, será que não era importante sair e caminhar ao ar livre, não esbarrar com ninguém? né, que pra gestação era muito importante, que iria ajudar no trabalho de parto, essas orientações também são muito válidas, eu acho que seria muito importante ter, então pra preparação e tudo mais eu acho que faltou assim sabe, de novo é tudo novo pra gente e o óbvio precisa ser dito e às vezes a gente nossa tinha medo assim de sair, mesmo que hoje a gente sabe que não ao as livre com todo cuidado não teria problema, lá a gente tinha muito medo.

Eu: Sim, se puder falar um pouquinho mais assim em relação ao que comentasse né, que tinhas medo com relação a pandemia, qual foi a influencia da pandemia nesse teu preparo pro parto?

E13: Sem dúvida, sem dúvida assim, eu fiz a inseminação pra engravidar né, então eu tinha muito desejo de fazer o parto normal, de correr tudo bem e me preparar e fazer, tentar fazer hidroginástica e eu tentar fazer vários exercícios pra eu estar super preparada, e quando veio a pandemia o pânico de acontecer qualquer coisa comigo, com meu bebê era superior a tudo, então assim, a minha preparação foi “não, vou esperar acontecer de acordo com o que a natureza me proporcionar e vou me cuidar o máximo, não vou arriscar” eu pensei em não arriscar de fato, não saia de casa, não encontrava ninguém nada, eu ia até o médico e voltava, tava trabalhando em home office ainda estou minha empresa me proporcionou isso, mas vi a minha família só um núcleo muito pequeno e outras pessoas da minha família eu avisa não posso encontrar ninguém, não posso ver ninguém porque eu não posso correr risco, então foi bem limitada a minha preparação, com muito medo, confesso pra ti o medo era gigante, e acho que todo mundo tinha muito medo no início né, claro que agora a gente vê que a situação já tá melhorando e tudo mais mas não tinha vacina, não tinha nada.

Entrevistadora: Sim, bem complicado isso né, e só vocês que vivenciaram isso que vão poder falar realmente como é que foi né mas o grupo de gestantes tu acha que o apoio mesmo foi em relação aos materiais que eles passaram e foi válido nesse sentido?

E13: Uma orientação básica né que a gente teve assim, a orientação foi fundamental sem dúvida, alguns pontos que eu tô citando pra ti que podem ser aprofundados mas no geral é maravilhoso eu achei super importante, depois eu me matriculei no da Unimed mas no da Unimed era muito power point , elas explicando ali aí eu pude comparar, e o da UFSC não né, vivência, experiência, então muito mais válido, uma pena que foi online né, a gente perde muito com certeza trocas e tudo mais, a gente acaba não tendo tempo pra perguntar acaba atrapalhando e ali no ao vivo a interação é muito mais rica, mas mesmo assim eu achei muito bom, muito válido, eu super recomendo assim, quem tá gestante tem que fazer, orientações básicas que a pessoa tem que ter sabe, a rede de apoio ela ajuda mas às vezes a rede de apoio ela tem conhecimentos muito ultrapassados e tudo mais e é bom a gente ouvir pessoas com experiências e vivências mais atuais também.

Entrevistadora: Pra finalizar e não tomar muito teu tempo, a última pergunta é lá no dia do teu parto, tu conseguia identificar os sinais de início de trabalho de parto, sinais do teu corpo?

E13: Quando saiu o tampão eu já, elas ensinaram isso né, eu fui lá e cheirei e aí eu senti o cheiro né de "qboa" que elas falavam né, e eu falei nossa entrei em trabalho de parto. E é uma intuição, os sinais e intuição, porque não poderia ter entrado né, porque não quer dizer que às vezes sai um pouquinho do líquido que a gente tá entrando em trabalho de parto, mas a intuição e um pouco do físico sim, porque a contração já não era mais o treinamento já começou a, claro que demorou algumas horas não foi no instante que eu percebi, demorou das 8 da manhã até ao meio dia, que aí começou a ter contrações um pouco mais ritmada mesmo que não era tão próxima mas tinha eu logo pensei "não, é hoje que vai nascer" e olha que eu tinha conversado com ela pra nascer 20 dias depois porque ela nasceu de 37 semanas, mas assim foi, a gente sente, a gente sente com certeza, o corpo te fala que ta vindo, não tem como não, e não tinha estourado bolsa nada, mas pelas contrações e pelas aulas realmente né, elas tinham sinalizado né, elas tinham falado de tampão que pode ser pode não ser, a contração se é ritmo tal, assim assim, então eu já tava ciente dessa informação, as aulas foram bem importantes por isso.

finalização

ENTREVISTA 14

Data da entrevista: 10/11/2020
DATA DO PARTO: 24/07/2020

Entrevistadora: Tu lembras como que era desde a gestação pra ti, como foi pra ti se preparar pro teu parto?

E14: Bom, na verdade assim pra mim a gravidez em si já foi no susto porque eu tinha um diagnóstico de endometriose de nível grave assim, e eu tava me preparando pra fazer um tratamento em relação a isso quando eu me descobri grávida, foi também num período de final de doutorado então tava no finalzinho da escrita da minha tese ali quase entregando e aí foi esse momento assim de muita expectativa da gravidez com essa questão profissional e logo depois no início da gravidez começou a pandemia então isso gerou muita ansiedade né de quando veio o lockdown eu tava completando três meses, cerca de 12 semanas então não tem como responder essa pergunta sem falar do período que a gente viveu como pandemia né, porque hoje quando a gente fala da pandemia parece uma coisa mais tranquila, mas quando chegou foi um susto muito grande mesmo, no primeiro momento assim foi muito impactante todo mundo trancado em casa e a gente com muitas incertezas, e eu vivi o momento do meu primeiro, desse final de primeiro trimestre de gravidez, por conta ja de um quadro de miomas e tudo mais, de sentir muitas dores assim, então eu precisei ir três vezes pra emergência obstétrica da Carmela Dutra nesse momento que tava tudo fechado, não tinha uma pessoa na rua, não sabia o que era esse vírus, depois precisei fazer tratamento de suplementação do Noripurum no posto de saúde quando, bem ali no início era a segunda ou terceira semana de lockdown, não tinha nem máscara sabe, fazia máscara improvisada de papel toalha pra poder ir pro posto com muito medo do que viria ser né, muito medo de pegar, de morrer, enfim toda essa ansiedade assim. E a minha preparação foi muito privilegiada por justamente também pelo período da pandemia porque daí eu pude trabalhar em casa, eu acho que o trabalho remoto privilegiou isso, o fato de eu tá em casa eu pude me dedicar então a procurar lives, palestras, cursos e nisso eu cheguei no curso do HU né, foi um dos cursos que eu fiz, o curso mais sistematizado assim que eu fiz mas eu fiz outros cursos eu assisti várias palestras e eu acredito que esse período de preparação tenha sido um lado bom digamos assim, desse período da pandemia né, de ta em casa e ter mais tempo pra olhar pra minha gestação, diferente do que se fosse o momento que eu não tivesse a pandemia e eu tivesse trabalhando, eu acho que eu não conseguiria ter me dedicado tanto a esse lado da espera das meninas.

Entrevistadora: Sim, e no início você já descobriu que eram gêmeas?

E14: Sim, eu descobri a gravidez e menos de duas semanas depois eu já descobri que eram gêmeas, bem no comecinho.

Entrevistadora: Uhum, e dai qual foi sua primeira preocupação em relação a como tavas projetando teu parto, tuas expectativas?

E14: Eu tenho muito medo de sentir dor né, então uma das coisas que eu pensava era como é que eu ia fazer um parto de gêmeas, porque eu sou da área da educação e penso muito essas questões do desenvolvimento humano e é importante que seja um parto normal, assim é, pra saúde, pro bem-estar, então a gente também tem uma romantização em relação a isso né então eu pensava muito que eu queria ter, sempre quis né, a quando engravidar ter um parto normal e aí com gêmeos já, opa, tem que pensar diferente, vamos ver o que que vai acontecer, então ja me fez pensar um pouco diferente e eu sentia muito medo do parto assim, eu queria me preparar, eu também sou da educação, sou da área da pesquisa eu tinha essa coisa de querer me preparar ao máximo e depois se eu contar o que aconteceu, não teria preparo nenhum que resolvesse a situação, porque às vezes as coisas acontecem então todo preparo não é suficiente nessas horas.

Entrevistadora: Travou um pouquinho aqui pra mim, mas pelo que eu entendi tu te preparou mas as coisas saíram um pouquinho fora do planejado, é isso?

E14: Bastante.

Entrevistadora: Se quiseres contar um pouquinho como foi?

E14: Sim, então uma coisa assim em relação ao preparo né que eu acho uma coisa que não foi falado nem no curso, nem nenhum dos cursos que eu fiz, a questão do que que significa ter um filho na UTI neonatal, que pode acontecer, na gravidez gemelar é mais comum né no caso de um parto prematuro.

(perda de conexão)

E14: Por mais que eu tenha feito vários cursos em nenhum momento eu ouvi falar sobre o que significa como se preparar em caso de você ter um filho que precisa de uma assistência numa UTI neonatal, então assim, desde o início eu sabia que a minha gravidez era de risco né, por ser gemelar, era de risco e a minha médica falava que tinha chance de serem bebês prematuros mas em nenhum em momento eu liguei o fato delas serem prematuras com o fato delas terem que ficar numa UTI neonatal, que pra mim isso na época foi o mais difícil de tudo, as minhas filhas ficaram 25 dias na UTI neonatal, então não fui preparada pra isso nem (perda de conexão) se teria preparo suficiente, se alguém consegue se preparar pra isso, mas nem informações nos livros que eu li, não foi algo que, me passou batido assim, não pensei seriamente sobre isso e eu participo de um grupo no whatsapp de mãe de gêmeas como é tocado nesse assunto tem algumas que são gestantes e que já pensam “Ah vou visitar a maternidade tal e vou ver se naquela maternidade tem UTI, como é que funciona” sabe, isso é uma forma de se preparar, e eu nem sabia se tinha vaga na UTI quando eu fui ter o meu parto sabe, assim também de última hora, não foi nada pensado na verdade.

(perda de conexão)

E14: Então, queres que eu repita alguma coisa, queres retomar alguma coisa?

Entrevistadora: Tavas falando que participas de um grupo no whatsapp com mães de gêmeas, isso?

E14: Isso, uhum, Tem mais ou menos umas quarenta ou cinquenta mães de gêmeos de vários lugares do brasil mas o grupo foi criado aqui em Floripa e a maioria é daqui, então esse grupo acaba sendo uma rede de apoio direta assim bem importante então eu vejo ali que às vezes são comentados assuntos como a questão da UTI e algumas gestantes ja se tocam e vão atrás disso de "ah, vou ver se o hospital que eu to indo fazer meu parto tem UTI, ou possibilidade de vaga, como é que é o atendimento" enfim coisas que eu não pensei na época, não tive essas informações né.

E14: Então, aí assim, tive a gestação toda bem legal bem preservada até justamente por ser pandemia, eu pude ficar em casa, fazer todo repouso porque eu precisei ficar mais em repouso assim a maior parte do tempo e tava indo super bem até ali 32 pra 33 semanas, eu tava indo super bem, todos os meus exames ótimos, inclusive indicando que as meninas poderiam nem ser prematuras né, nesse momento do exames dela tava normal e os meus exames estavam todos ótimos mas eu comecei a sentir uma dor muito estranha, que e achava que era dor delas se mexendo e tudo mais, até que com 34 semanas eu fui pra emergência né, tive elas no hospital Ilha, falei com a minha obstetra no dia em que eu tive uma alteração importante nas fezes, na coloração das fezes e da urina e ela se atentou pra um problema de fígado e me pediu pra ir direto pra emergência, pro hospital e aí eu fui já internada, eu tava até com minhas coisas prontas porque como falaram que elas podiam vir antes e tal, ja tava com tudo pronto organizado pra ir, só que assim, é o que eu te falo, entre racionalmente pensar que elas podem vir antes, eu não sabia que elas ficariam na UTI sabe, não sabia o que que significava ter um filho na UTI, não sabia o impacto disso pra amamentação por exemplo né, de ter contato com elas 24 horas depois do parto, enfim, toda uma situação que assim, psicologicamente até hoje é difícil de lidar e aí eu ganhei elas com 34 semanas e dois dias, depois de dois dias internada, duas noites e um dia internada, e aí eu fiquei ao todo seis dias internada, eu fiz cesárea né, elas assim que nasceram já foram levadas pra respiração e já foram direto pra UTI e eu só fui ter contato com elas no dia seguinte, e aí tive alta do hospital quatro dias depois do parto e elas ficaram mais 20 dias lá, e aí a gente tinha a rotina de fazer as visitas né, pra tentar estimular a amamentação e tudo mais, acabava passando o dia la na UTI e vinha dormir em casa.

Entrevistadora: E lá na gestação tu fazias alguma coisa pra te preparar pro teu parto?

E14: De atividade física por exemplo?

Entrevistadora: É pensando em tudo assim né, pensando no tipo de parto, de preparação como um todo assim.

E14: Ah sim, fiz, eu fiz o curso do HU, eu participava de uma roda de gestantes de São Paulo também que daí como teve a pandemia eles acabaram fazendo virtualmente e daí possibilitou que pessoas de outros lugares participassem, e essa roda de gestantes é com pessoas que trabalham com medicina antroposófica então tem uma perspectiva bem do parto humanizado né, e também participei de rodas de gestantes de parto humanizado aqui de Floripa, assisti alguns vídeos, aí em casa eu fazia um pouco de yoga, fazia um pouco de dança, mas tudo dentro de casa né, sem sair e é isso assim, sempre tentando estudar, lia um pouco mas foi isso que eu fiz de preparação assim, nada muito específico. Mas eu tinha ideia de que seria difícil né, dificilmente eu teria um parto normal mas eu queria muito aquele momento, aí com minhas filhas depois do parto sabe tipo a hora dourada ali, amamentação e essas coisas todas eu nenhum momento achei que não pudessem acontecer.

Entrevistadora: Sim, e qual achas que foi a influência do grupo de gestantes nesse teu preparo pro teu parto e na forma que aconteceu teu parto, nesse sentido?

E14: O grupo pra mim assim, aquele momento do, o grupo tu diz o curso mesmo né, as aulas, pra mim assim foi bem importante porque embora eu participasse desses outros eventos e rodas eu sentia que o curso do HU ele me ancorava mais, ele trazia mais conhecimento científico, mais conhecimento técnico, até por eu ser ali da Universidade então eu sempre me sentia muito segura naquele ambiente que eu tava lidando com pessoas que são da área, que pesquisam, desse vínculo da pesquisa, o ensino e a extensão, então eu me sentia segura nesse ambiente falando com pessoas que são professoras da Universidade ou bolsistas como vocês, vocês que estão ali na ponta no HU mas ao mesmo tempo pesquisam, estão atualizadas, então ali eu me sentia mais segura do que participando de outros eventos que eu participava né. E o acolhimento ali principalmente da [nome da profissional] que conversou comigo inclusive no privado assim várias vezes, teve uma conversa assim bem longa comigo no dia que eu tive uma, não bem uma crise de ansiedade mas tava muito nervosa porque ficava pensando “ai gêmeas, não vou saber criar gêmeas” sabe, minha ansiedade muito grande era pelo fato de serem gêmeas, o que que eu vou fazer com duas crianças, daí ela conversou muito comigo, ai teve a [nome da profissional] que trabalha com a amamentação depois que as meninas nasceram ela me deu um suporte assim, depois que as meninas nasceram não, depois que as meninas vieram pra casa e tiveram alta da UTI, e elas tinham muito refluxo e eu não sabia se eu tava fazendo certo a questão da amamentação, ela também, embora ja fizesse um tempo do final do curso e tal, ela disponibilizou o contato dela e eu conversei e tal, ela foi super querida comigo, conversou várias vezes, falou da experiência dela que ela é mãe de gêmeas, então assim eu acho que o curso em si possibilitou esses conhecimentos que eu me sentia segura durante as aulas, eu tenho tudo anotado, eu acho que eu anotei umas 30 páginas do que era conversado ali e de vez em quando ainda consultava mas assim também esses contatos assim, tu saber que poderia contar com essas pessoas de certa forma como uma rede de apoio depois né, a [nome da profissional] ainda conversou comigo no período do curso mas a [nome da profissional] veio depois, e aí o grupinho que se formou ali com as outras mães também que a gente, agora tá bem parado assim, mas eu lembro que nos primeiros seis meses depois do curso o grupo foi bem movimentado, tinha muita troca de informação, pessoal mandava foto

dos bebês, depois vai dispersando acho que é normal, mas o início ali que é bem importante que a gente tá se sentindo sozinha e foi legal porque mesmo a gente não ter se encontrado pessoalmente né, ninguém se conhece pessoalmente mas o grupo ficou um grupo forte assim. O material disponibilizado também pelas facilitadoras do curso no outro grupo que tem de envio de material, esse material era muito bom, algumas coisas me ajudaram bastante, então pra mim assim eu recomendo pra todo mundo o curso do HU.

Entrevistadora: Que bom, que ótimo que foi bom pra ti né.

E14: Foi bem significativo, eu acho que, é porque é como eu falo, não tem como eu falar dessa gestação sem falar da pandemia sabe, a pandemia deixou a gente muito sozinha, muito isolada, então aquele momento, aquela quinta-feira a tarde do curso era meu Deus eu to com outras pessoas sabe e essas pessoas que tão compartilhando o mesmo momento que eu era, fico até emocionada, porque era um momento que tu conseguia ter um pouco de contato, acesso, sei lá alguma coisa que te tirava daquele momento de tanta incerteza que a gente tava tendo da pandemia, acalmava, trazia uma serenidade, trazia informação de qualidade, então foi uma âncora mesmo.

Entrevistadora: Sim, que bom! Que bom, porque só vocês que passaram por isso na pandemia né que vivenciaram isso na prática que podem dizer mesmo né, por isso essa conversa aqui, porque eu não poderia colocar outra coisa sem ter a fala de vocês né, que são as que mais sabem sobre isso, que passaram por isso na prática.

E14: É não sei se as outras tem falado sobre essa questão da pandemia, mas eu acho que como estais fazendo esse recorte com o grupo que passou por isso, não tem como no teu TCC deixar de fora, esse contexto histórico do momento que o mundo parou, digamos assim né.

Entrevistadora: Sim, com certeza, e eu até pergunto qual foi a influência da pandemia no teu preparo pro parto já falasse algumas coisas do medo de se contaminar, não sei se queres falar mais alguma coisa…

E14: É tem o que eu queria deixar bem claro é que teve os dois lados, teve o lado do medo, da incerteza da ansiedade, de uma frustração muito grande porque a gente engravida a gente quer passear com a barriga na rua, quer tirar foto, quer ta com as pessoas que a gente gosta e daí eu fiquei meses sem poder ver os meus pais e os meus irmãos, a gestação inteira eu não vi uma amiga, eu não compartilhei com ninguém sabe, então teve esse lado, que eu não pude sair pra fazer foto, enfim, teve esse lado todo das restrições. Mas por outro lado também privilegiou eu tá em casa focada quase que cem por cento na gravidez porque por mais que eu trabalhasse no remoto porque eu sou professora e ano passado até eu ganhar as meninas eu tinha 40 horas semanais na prefeitura de Florianópolis mas com o ensino remoto isso se flexibilizou bastante e ficamos eu e meu esposo só em casa cuidando, curtindo essa gravidez, que se não fosse por essa pandemia ia ta cada um no seu emprego, só se encontrando e vivenciando muito pouco né, então o foco seria outro, então assim a gente conseguiu se cuidar, se curtir e aproveitar, eu conseguia todos os dias pegar sol na barriga, cantar pras

minhas filhas e eram coisas que se fosse a rotina normal né trabalhando até próximo ao parto eu não conseguiria, então acho que tem esses dois lados da pandemia.

E14: É isso, eu acho que o curso teve essas três questões de influência, a questão de vir da Universidade que eu por ser uma pessoa da Universidade valorizo muito assim, então nesse sentido era meu porto seguro, que eu sabia que tava recebendo informação de qualidade, de confiança, atualizada né, essa ponte da pesquisa, ensino e extensão, então o momento do curso acontecendo em si, esse momento dos contatos que o curso abre, possibilita e a forma que as facilitadoras, não sei se chama assim enfim que nem todas são professoras né, facilitadoras ali que ministram as aulas, a forma super acolhedora como elas se disponibilizaram a cuidar da gente, eu não sei as outras meninas mas eu me sentia cuidada, amparada por elas, tanto nos encontros quanto nesse pós encontro quando eu precisei eu realmente encontrei ajuda ali nas situações pontuais que eu precisei e o grupo de mães que se forma, as que continual ali ainda trocam bastante informação então tem esses três pontos assim que são pontos de destaque.

E14: No curso falaram temas que pra mim foram bem importantes, a questão do Baby blues, da depressão pós-parto na aula sobre puerpério e realmente, precisa até ter um investimento maior de tempo pra falar um pouco sobre isso, porque só quando a gente passa mesmo que, nessa parte das intercorrências, do que pode dar errado e a gente tem que se preparar né, eu vi a menininha que tava do leito do lado das minhas na UTI falecer, tanta coisa pode acontecer, mas tem muito tabu ainda em muitas coisas, a própria amamentação, a amamentação em uma situação de UTI é totalmente diferente, tu não tem autonomia, tu é ordenhado por outra pessoa, eu nunca imaginei passar por isso então coisas que poderiam ser incluídas nos próximos cursos, falar um pouquinho do que pode não dar muito certo e que a gente precisa ter um pouco de informação pra passar de uma maneira mais leve.

finalização

ENTREVISTA 15

Data da entrevista: 12/11/2021
DATA DO PARTO: 23/09/2020

E15: Foi dia 23 de setembro de 2020, foi lá em Biguaçu, na maternidade de Biguaçu, foi um parto natural, foi por uma enfermeira obstetra, foi bem bom, só que a única coisa que teve foi que teve que ser induzido, foi induzido pela, não foi pela ocitocina, foi por aquele comprimido, eu sempre esqueço o nome.

Entrevistadora: Misoprostol?

E15:: Isso, foi dois comprimido e foi dez horas de parto, porque ela nasceu com 41 mais 3 ja tava se encaminhando para 42 aí o obstetra que tava lá, a gente conversou certinho e ele me convenceu.

Entrevistadora: E lembras como é que foi desde a tua gestação, como que foi pra ti se preparar pro parto, se quiseres contar um pouquinho

E15: Sim, quando, ela foi planejada né, o que não foi planejado foi o Covid né, a pandemia, então eu tive certeza mesmo foi, fiz o exame em janeiro e aí logo em março a gente entrou em pandemia né aí fechou tudo, todo mundo pra casa, e eu tava bem no começo da gestação então eu peguei ali o começo de pré-natal em janeiro, fevereiro, e março já não teve, poucas, não teve consulta, fechou e a gente retornou acho que ali finalzinho ali de abril, no meio de abril, tenho anotado isso, depois se tu precisar eu te mando pelo whats. Mas foi bem acompanhado, o que que tinha naquela época, não tinha muitos dados sobre gestante com Covid, no começo que não tinha nada que afetava o bebê, ou prematuro porque as coisas que a gente sabe que acelera o parto hoje em dia né, mas naquela época não tinha tanta informação, então o que que eu fiz a gente tentava não, pra me preparar pro parto eu li bastante, eu pesquisei bastante, li livro, pesquisava, o grupo de apoio do HU que teve todo aquele respaldo, foi online, foi ótimo, além disso pesquisa, pesquisa, me preparei pro parto de maneira fisioterapia pélvica, em casa, tipo bem autodidata, eu fui atrás das informações além do que vocês já tavam passando né, livros e tentei não focar na pandemia, por mais que aquele momento tivesse ali existindo né, a única coisa que eu tentava entrar mais por dentro era se tinha alguma coisa em relação a gravidez, a parto, os riscos e naquela época bem no começo não tinha tanta informação, aí foi isso que eu fiz pra tentar uma gravidez mais tranquila, não via jornal, tentava não focar porque sabia que o psicológico ia abalar né.

Entrevistadora: E aí, falasse que em março deu uma parada nas consultas de pré-natal né, achas que isso afetou em alguma coisa?

E15: Foi, foi bem em março, logo ele já remarcaram por conta do, por conta que era pré-natal então tinha prioridades ali né, e aí logo retornou então não fiquei tão preocupada assim né. Foi uma gravidez tranquila, sem, não teve, só engordei mais no final assim da ansiedade do

parto mas foi sem pressão alta, foi sem diabete, então eu já chegava nas consultas, como a pessoa pesquisa, vai atrás, sabe o que que vai ter, o que que ta preparando, eu ja chegava pedindo exame tal, quero esse exame, então foi bom por isso, tu não chega esperando que o médico te falei ou a enfermeira te fale que te atenda, tu já chega questionando, perguntando qual o próximo passo, o que que precisa o que não precisa, esses exames, tal, tal, quero fazer, então eu acho que nem só em tempos de pandemia mas em qualquer gravidez sem ser pandemia eu acho que as mães deveriam fazer isso né.

Entrevistadora: Sim, e daí logo no mês de março começou o grupo de gestantes né, e achas que qual foi a influência do grupo de gestantes no teu preparo pro parto, te ajudou de alguma forma?

E15: Muito, aquele material de massagem pélvica, outra que vocês mandaram agora, massagem pélvica, fisioterapia pélvica, aquilo ali meu Deus, aquilo ali acho que foi perfeito, no caso eu não tava conseguindo arranjar uma fisioterapia pélvica, fisioterapeuta pélvica, então eu mesma fui autodidata, pesquisa vídeo, ver vídeo e material mesmo tava bem claro.

Entrevistadora: Uhum, e no dia do teu parto, tu consegues lembrar um pouquinho mais como que começou os primeiros sinais?

E15:: Então, como foi induzido a partir de 40 semanas o médico começou a dizer "Ò, tem que começar a ir na maternidade", como eu já sabia até a maternidade que eu queria ganhar que era lá, eu comecei a ir lá, lógico que tem médicos bons e médicos não tão acolhedores, médicos frios, e aí logo que eu cheguei lá eu peguei um médico mais desse e os outros todos não tenho o que reclamar, nem enfermeiro nem nada. Então, ele queria que eu fizesse cesárea, ele queria que eu chegasse e ja fazer uma cesárea e disse assim "Se tu chegar aqui com 41 e se tu não quer induzir tu nem vem pra maternidade e tal tal" bem assim desse gênero, e aí só que como era uma única pessoa aquilo ali não me abalou muito, quando chegou com 41 semanas e dois dias teve um médico que disse "Ó, seria ideal induzir pelo risco de mecônio e outras coisas que a gente já sabe né, e aí então eu disse "Não então eu vou, mesmo não querendo eu vou induzir, vamos tentar com comprimido" convenceu e foi, aí teve aquele toda hora, foi dez horas né, ai tentei fazer o parto mais ativo possível, cavalinho, bola e tudo mais, peguei mesmo todas as dicas, todas as informações de estudo e tentei fazer um parto mais ativo possível, meditação, meditação muito na hora ali né, pra tentar amenizar a dor e posições eu escolhi, não foi um parto, não foi deitada, foi meio de cócora, eu sabia até a hora de pedir anestesia, eu sabia que eu podia apelar pra anestesia, aí a enfermeira obstetra me deu um remédio pra amenizar a dor que não adiantou de nada né, aí depois ela me explicou "Geralmente quando a gente dá uma anestesia, mesmo sabendo que tu poderia, é teu direito, geralmente a gente acaba perdendo a sensibilidade e acaba indo pra cesárea, coisa que tu não queria né" ela me falou, conversou bem, ai tu quer detalhes ali na fase expulsiva?

Entrevistadora: Pode falar, se tu lembrar né.

E15:: Era cinco e pouco da manhã, não, três e pouco da manhã ela estourou a bolsa pra acelerar aí foi mais, a parte mais intensa porque começou a dilatar mais rápido, já tava com sete ali quase seis, seis pra sete alí, aí ela disse "não, vou estourar pra dar uma acelerada", aí só 5h52 foi a hora que ela nasceu, tava num quarto sozinha só com meu esposo. Essa pandemia uma coisa que me deixou bem ansiosa ali no final foi que a gente tava no próprio grupo com informações que os esposos não tavam podendo entrar, só na hora do, e depois ficariam sozinhas e daí foi um dos motivos que eu escolhi a maternidade lá de Biguaçu que tava podendo ficar, ficava um dia se fosse normal ai o que eu optei também, foi uma das coisas, então aquilo ali me deixou um pouco ansiosa em tempo de pandemia, imagina né primeiro filho tu tá sozinha, não é que tu ia ta sozinha ia ter ali a equipe mas depois o enfermeiro ia sair tu ia se virar né, isso me deixou muito ansiosa. Só que ai eu fiquei tranquila porque no caso de lá não tava sendo assim ainda né, ali em setembro. Ela esperou aquele, 5h52 ela nasceu, não teve laceração, não teve violência obstétrica, ela nem tocava em mim, nada, nada, nada e ela foi bem paciente ali, ela nasceu com 3 quilos cento e pouquinho, aí esperamos o batimento do cordão, mamou na primeira hora, teve aquela hora dourada, teve tudo e aí esperou expulsar ela não saiu nenhum minuto de perto de mim, no máximo foi pra fazer os exames da pediatra ali do lado, como eu tinha feito o exame da clamídia e gonorreia eu optei por não pingarem o colíriozinho, nitrato de prata, mas a vitamina K ela tomou, eu assinei um termo. Fui pro quarto, depois eu acho que de algumas horas que ela, não, acho que só no outro dia que ela tomou banho, esperamos por causa, ela não nasceu com tanto vernix mas fui eu que dei o primeiro banho, eu quis dar o primeiro banho, então as enfermeiras "como queres dar o primeiro banho tu pode dar a hora que tu quiser" ai eu esperei né, porque elas tinham o horariozinho pra elas darem né, pra ficar no padrão delas ai elas "não, queres, da a hora que tu quiser", mas foi muito muito bom assim, no meu ver foi um, mesmo em pandemia foi tranquilo, pra mim por optar não ver noticiários, querer cuidar da minha mente, eu deixei essa parte mais pro meu esposo, ele que se informava né, a não ser na parte de gestante que eu sempre tava vendo informação e tal, então isso me tranquilizou. Depois do meu parto que aí teve vários casos de bebês que nasceram prematuro e tudo mais né.

Entrevistadora: Qual tu achas que foi a importância do teu preparo pro parto? Se quiseres falar um pouquinho

E15: A importância pro meu trabalho de parto fez total diferença pra que fosse do jeitinho que eu queria, foi tudo do jeito que eu queria, a única coisa que foi induzido que né, era uma parte que eu queria que fosse mais natural ali, mas foi perfeito, e cheguei com meu plano de parto que foi falado no grupo levei o plano de parto mesmo sendo um hospital público e eles respeitaram totalmente assim né, como eu digo aqui pra, eu tenho meu espaço que eu faço as minhas vendas da herbalife eu tenho meu espaço, então eu digo pras minhas clientes né que não me traumatizou, muitas se traumatizam com o parto, pré-parto né, não me traumatizou nenhum um pouco assim, não foi traumatizante, lógico que eu saberia que ia doer mas justamente o preparo nesse período foi essencial, como no grupo a gente falava a dor é inevitável mas sofrer aquilo ali que não é o legal né, não é legal nem pro bebê. Então aproveitei bastante cada momento, tanto do parto, tentei ser o mais ativa possível.

Entrevistadora: Falasse que chegasse a fazer o plano de parto né, e falasse que chegasse a fazer no grupo de gestantes então como chegasse a conhecer o plano de parto?

E15: Na verdade, o grupo de gestantes me deu bastante apoio e informações tudo, o plano de parto eu já sabia que existia porém o que me encorajou a levar um plano de parto pra um hospital público foi o grupo, porque né, a gente tirou todas as dúvidas, perguntou se podia, tirou cada aula que tinha ali a gente esclarecia nossas dúvidas, então foi um grupo bem ativo, foi um grupo de troca de muita informação.

Entrevistadora: Que legal que conseguisse passar por esse processo de uma forma mais leve mesmo diante de um contexto de pandemia, deu pra perceber que estavas conectada, sabia cada etapa do teu parto e conseguias decidir conscientemente, conseguias participar ali né, como falasse do parto ativo, isso é bem importante, então que bom que foi assim pra vocês.

E15:: Foi foi, foi pra mim bem maravilhoso, eu não posso falar pela experiência dos outros mas eu tenho conhecidas minhas que até hoje eu não sei que nível de informações que elas ficaram vendo televisão porque o neném delas nasceu agora e só os avós e padrinhos chegam na casa dela ela se faz as pessoas se paramentar, aquela proteção toda pra chegar na pessoa e tal, sim tem que ter o cuidado mas acho que o excesso mesmo em tempos de pandemia acho que não é legal né, não é saudável, então até hoje ela é assim, eu tento ter esse cuidado todo tanto foi na pandemia no começo ali a gente cuidava bastante pra ir no mercado, esses cuidados que não é normal do nosso dia a dia né, que a gente teve que ter, mas tentei não focar na doença, procurar jornal pra poder ser mais leve.

Entrevistadora: Uhum, e ali sobre a maternidade, só pra gente finalizar pra não ocupar muito teu tempo, sobre a maternidade, falasse que escolhesse a maternidade por causa ali do acompanhante né, como é que foi pra ti essa escolha?

E15: Eu moro em São José, na Forquilhinhas ali, teria o Regional mas na maternidade de Biguaçu três amigas minhas já tinham tido ali e ela ta toda novinha, ela ta toda reformada, por fora não é tão bonita assim mas por dentro já é outro mundo ali né, tudo novinho, tudo, ali tudo, tá toda reformada e nossa ta bem maravilhosa lá, então tipo ai foi por isso também foi por indicação e por ela estar toda reformada, toda certinha né e também pelo fato do acompanhante que eu fiquei mais ansiosa assim.

Entrevistadora: Das minhas perguntas são essas, não sei se tens mais alguma coisa que queres colocar.

E15: No meu caso eu tentei deixar a forma mais leve possível, pelo fato de eu saber, antes de engravidar eu tenho amiga né, psicóloga, parapsicóloga, então esse processo antes da gravidez, mesmo não tendo pandemia eu sabia que se eu tivesse uma gravidez estressada, agitada, ia refletir tanto pra mim quanto pra saúde dela né, então eu tentei não focar nisso tanto.

E15: Eu faço, outra coisa que eu ia te falar, eu gosto muito e eu faço geralmente todos os meus acompanhamentos na UNISUL na Palhoça mas devido a pandemia também não foi do jeito que eu queria tanto, porque teve que ser mais pelo posto do que pela UNISUL, peguei mais o começo e o final do acompanhamento da gestação né e foi mais pelo posto e também ainda bem que como tu falou né, informação é tudo, porque eu já chegava no posto ja com as coisas que eu queria fazer né, pedia ultrassom, pedia exame de isso ou daquilo, mesmo não sendo normal do SUS, não ser padrão, isso eles falavam pra mim "Isso não é padrão mas se tu quer" eu falava "Não, eu quero fazer", inclusive exame da clamídia gonorreia né, que coisas que eles não pedem a não ser que eles tenham desconfiança, e aí foi isso também me atrapalhou um pouquinho a pandemia mas foi, num contexto geral foi tranquilo.

E15: O grupo de gestantes, inclusive foi eu que fui atrás né do grupo de gestantes, deu todo um respaldo por trás, mesmo que tu pesquise, leia livro né, que eu tava lendo alguns livros sobre essa parte ali, depois eu te mando alguns livros que eu pesquisei fora a internet, o grupo em si são mais detalhados né, com certeza, era um grupo só pra isso, na consulta tu acaba só exames, coisas padrão como eles falam né, tira uma dúvida ou outra que tu possa perguntar, mas a própria enfermeira lá do posto que ela já chegava "Meu Deus se todas as gestantes fossem que nem tu, que pesquisasse que quer exame que quer tudo, seria uma maravilha" eu dizia a deveriam né, deveriam tá todo mundo num grupo de gestantes, eu até passei o número do grupo do HU dessa parte de grupo de grupo pra ela passar pra outras mães, que ela também não tinha.

*finalização**

ENTREVISTA 16

Data da entrevista: 15/11/2021
DATA DO PARTO: 12/07/2020

E16: Meu filho nasceu dia 12 de julho de 2020.

Entrevistadora: E como que foi assim desde a tua gestação pra se preparar pro parto?

E16: Então, eu descobri que estava grávida precisamente no feriado de 15 de novembro de 2019 só que eu estava viajando a gente estava em São Paulo e já tinha feito o teste de gravidez e voltando aqui em Floripa fomos lá no posto, aí eu falei que tinha feito o teste e aí foi o primeiro atendimento com a enfermeira lá e ela falou assim "ah tá, testes são super efetivos, vamos começar o processo de pré-natal", então eu comecei ali no posto, eu moro aqui no campeche e a gente fez essa primeira parte do pré-natal aqui no posto que foi até março quando foi declarada a pandemia né, e essas primeiras consultas foi tudo tranquilo, ai se não me engano era uma por mês, aí eu ja fiz os primeiros exames que tinha para ser feito né, os primeiro encaminhamentos para a ecografia para esses exames mais especializados, só que quando veio a pandemia precisamente eu tinha marcado uma consulta no dia que mudou tudo assim, eu fui lá acho que era 16 de março por aí e a enfermeira falou "não, desculpa mas não vamos conseguir te atender, a gente esta se organizando pela questão da pandemia, vamos tentar fazer os atendimentos pela telemedicina, computador enfim" e aí parou, só que nesse mesmo momento eu estava com muitas infecções urinárias, eu tive várias na gestação e me encaminharam para ginecologista, obstetra, não me lembro bem, e aí eu fiquei em alto risco, então meus atendimentos mudaram daqui do Campeche pro Continente pra, não me lembro como se chama, a UPA do Continente eu acho que chama mas passaram a ser muito mais espaçadas assim, as consultas normais eram uma por mês e depois eu passei a ter consultas a cada dois meses, muito muito espaçadas e isso foi um pouco complicado. E nesse ínterim também eu tava com várias infecções então eu tive que ir lá pro HU várias vezes por essa questão das infecções, enfim, acabou que meu pré-natal foi muito, enfim, eu quase não tive consultas no final por exemplo a primeira ecografia que é pra ser com 12, 13 semanas eu fiz com quase 22 semanas, imagina, ou seja, muito muito avançada a gestação e até perigoso né porque se, ainda bem que o bebê tava tudo certo com ele e tudo mais mas, como também financeiramente não tinha como arcar o pré-natal no privado e tal, então ficou meio assim complicado para ter os atendimentos em tempo, foi mais ou menos isso.

Entrevistadora: Uhum, e aí nessas consultas mesmo que espaçadas vocês conseguiam abordar alguma coisa sobre o parto e tuas dúvidas?

E16: Não muito, bem mais pro final assim, porque eu comecei então diante da situação da pandemia que eu estava impossibilitada de sair pra procurar atendimento, e acima de tudo eu tinha muito medo e tava muito recente a questão do vírus, a gente não sabia, tinha medo de pegar, tinha medo de passar pro bebê, tinha medo de morrer, então eu comecei a procurar

ferramentas aqui em casa para tentar levar uma gestação o mais saudável possível aí comecei a fazer yoga, assim de tutoriais no Youtube que uma cunhada passou pra mim, ela é médica mas eu sou da Colômbia e ela mora lá na Colômbia e ela me passava tutoriais e eu comecei a fazer, depois ela me recomendou uma doula de lá e a gente marcou uma consulta com ela só que eu não me senti bem de ficar com a abordagem dela, porque era assim tipo muito mística sabe, muito os chacras e essas coisa e na verdade eu não curto muito esse papo sabe, eu sou mais voltada para as coisas objetivas, científicas, enfim, então isso não deu certo, e eu já tava sabendo do pré-natal da UFSC por uma amiga que já tinha tido uma experiência e o bebê dela nasceu em 2019 e ela tinha me falado muito bem do grupo de casais e gestantes da UFSC, então eu procurei as informações e eu me inscrevi, mas tive que esperar um pouquinho porque vocês exigiam períodos né, então no meu caso era pros bebês que iam nascer entre julho e setembro. E aí quando começou o grupo foi legal, mas também né a gente fica meio desapontado porque não é o mesmo que se ter o contato direto com as pessoas, a questão da virtualidade também muito, às vezes é muito supérfluo assim, você tá ali tá na tela a interação é outra coisa então você acaba também perdendo coisa que talvez no presencial teria e a gente aproveitou, foi bem bacana o espaço ali com vocês, mas eu sempre senti falta de uma coisa mais, sei lá mais ao vivo.

Entrevistadora: Uhum, e achas que o que foi abordado sobre o parto assim nos encontros te ajudou de alguma forma?

E16: Sim, ajudou, ou seja, a forma como o curso está estruturado era muito boa assim, porque em cada encontro era uma temática diferente, um material de apoio também, a gente lia tudo aqui em casa, porque claro eu ia ser mãe de primeira viagem então a gente tá com todos os medos em cima, então líamos os materiais e sobre a questão do parto especificamente eu sentia que estava super preparada assim com as informações que tinha, a gente já tinha decidido que o bebê ia nascer lá no HU e também por toda essa questão de ser uma referência em parto humanizado e tudo mais, a gente queria ter uma experiência dessas, evitar passar por uma cesárea, enfim, qualquer coisa que fosse mais natural possível e acho que essas informações ajudaram muito pro momento do parto.

Entrevistadora: Uhum, e além dessas livros que você lia e a a Yoga, fazias alguma parte física de preparação ou era mais conhecimento?

E16: Não, fisicamente eu fiz a yoga e no último trimestre eu comecei a dar caminhadas assim aqui pelo bairro, então eu tenho cachorras e aproveitava e pegava ele dava uma volta assim uma meia hora, devagar, eu não ganhei muito peso, eu sou magra né então não ficava assim muito cansada, não era uma coisa que me demandava muito esforço físico mas eu sentia uma diferença sim no estado físico como um todo né, era uma coisa hoje de fazer.

Entrevistadora: Sim, e lembras no dia do teu parto como foi, se lembrares de contar dos primeiros sinais como é que aconteceu?

E16: Isso foi outra coisa, a data provável do parto era dia 10 de julho e eu fiz aniversário dia 06 e no dia seguinte eu tinha marcado uma consulta no posto porque aconteceu outra coisa que nos últimos, no último mês eu consegui voltar nos atendimentos aqui no posto porque eu me queixei que os atendimentos estavam sendo muito espaçados, que não estava tendo um atendimento bom, considerando que estava próxima do parto, então voltei aqui no posto aí eu tive que fazer novos exames, enfim e comecei a marcar consultas semanais e no dia seguinte no meu aniversário era uma consulta marcada eu fui lá e eu estava com a pressão muito alta, então o médico me viu a enfermeira, sei lá, e eles se reuniram e tal e eles me encaminharam pro HU, por conta da pressão e também porque naquele momento os batimentos cardíacos do bebê aparentemente estavam indo muito devagar então eles queriam ter certeza que eu estivesse bem, que o bebê também estivesse bem, então me encaminharam para lá, isso foi dia 07, aí eu fui lá cedo e foi feito o protocolo para pré-eclâmpsia né, se fala assim?

Entrevistadora: Isso.

E16: Então eu fiquei lá sendo monitorada o dia todo, a pressão tava alta mesmo, tava com picos assim 14/15 por 10, tava alta mesmo mas ficaram monitorando o bebê, tava tudo certo, ali eu estava com 39 semanas e naquele dia os médicos também se reuniram e decidiram que iam esperar até a semana quarenta para induzir o parto, então eu fazia as quarenta semanas na sexta feira e eles me falaram pra eu voltar lá na sexta-feira que era dia 10, que era a data que eu tinha, então eu voltei lá dia 10 já com todas as coisas, com tudo pronto e assim que cheguei eles já me internaram pra induzir o parto, só que naquele dia tava muito lotada a maternidade, enfim, eu fiquei lá o dia todo esperando uma vaga, enfim, foi um pouco pesado assim e cansativo e só naquela noite, perto da meia noite que consegui ir para a parte dos parto que eu já não me lembro como se chama, aí ja tinham um quarto com outra moça e eles me induziram o parto, começaram a induzir o trabalho de parto, então colocaram ocitocina, e eu passei essa noite de sexta pra sábado, na verdade madrugada de sábado, já sentindo as primeiras contrações e na manhã do sábado começaram a se intensificar e a enfermeira passava me perguntando a cada quanto tempo eu estava com as contrações, umas três horas da tarde eu já comecei a sentir umas dores mais fortes assim e aí eu já pedi pra que eles deixassem entrar meu companheiro porque tava restringido também o acesso deles, então aí ele conseguiu entrar e aí eu já fiquei também um pouco mais tranquila porque já tava me sentindo muito sozinha nesse dia, dá muito medo né, aí ele conseguiu entrar e o problema é que tava dilatando muito devagar, então o trabalho de parto estava indo mas tava indo lento.. Mas as contrações foram e as horas foram se incrementando e eu comecei a ja me sentir muito desconfortável, aí eles me recomendaram para começar a caminhar ali pelo espaço que tinha disponível eu fui tomar banho de chuveiro, tava fazendo movimentos na bola, todas essas coisas que passavam no pré-natal que era pra fazer eu fiz, aí meu companheiro fazia massagem para mim enfim, e umas dez horas da noite eu já estava com muitas dores fortes assim mas a dilatação estava indo devagar, depois o tampão saiu meia noite, umas duas da manhã a médica passou ela me fez um toque e ela falou assim “Não, estamos indo muito devagar, se você não melhorar nas próximas horas vamos ter que fazer uma cesárea" mas era tudo que eu não queria ouvir sabe, eu não queria, não queria, não queria, e daí eu acho que com essa pressão que ela me botou fez com que o corpo começasse a ir mais rápido porque

uma hora após ela veio de novo, fez o toque de novo e aí eu já estava com oito e meio assim, tinha aumentado bastante e umas três da manhã eu já comecei a sentir assim aquela necessidade de fazer força, então comecei o período expulsivo que chama né, comecei comecei só que não tava dando certo não sei, eu estava muito estressada e também eu estava fazendo a força no, acho que estava fazendo errado porque falam pra você fazer uma força como se fosse pra fazer coco, mas na real não é isso, sei lá eu fiz muitas coisas, sentei no cavalinho, peguei aquela barra de cima da maca, mas nossa eu ja tava muito muito cansada, tava quase dormindo enfim, e depois veio uma enfermeira, eu acho que ela percebeu que eu tava muito estressada, que não tava conseguindo e ela botou uns óleos essenciais, sei lá, ali no quarto, umas músicas pra eu relaxar e enfim, sei lá eu comecei a me sentir mais confiante e chegou um momento que a gente ficou sozinho porque tava tendo outro parto na sala ao lado e aí eu falei com meu companheiro, não, vou fazer dar certo, vamos agora. Aí num momento que a gente tava sozinha eu peguei aquela coisa da maca, fiz a força e aí ele começou a vim, então meu companheiro chamou o pessoal e aí veio todo mundo, e aí eles me direcionaram já pra fazer o bebê nascer e aí eu fiz aquela força, saiu a cabeça e a metade do corpo dele aí a enfermeira falou "tu vai sentir muita dor agora mas não é para voltar, faça toda força que você puder que ele ta vindo já" então tá, e aí quando eu senti a contração eu fiz aquilo e ele nasceu, e aí aquela coisa né, aquele alívio e eles colocaram ele aqui em cima no meu peito, só que ele tava com desconforto respiratório, então deixaram só uns segundos assim, eu me lembro que eu vi o ombro e um braço dele que tinha muito pelo, eu não consegui ver muito ele, só essa parte aqui, a cabeça ele também é super peludo e tal e aí tiraram ele e levaram, então eu fiquei nesses procedimentos posteriores que fazem na gente tirando a placenta, enfim, todas aquelas coisas e meu companheiro foi acompanhar o bebê na UTI enfim, porque ele ficou o primeiro dia na UTI.

Entrevistadora: Uhum e nesse teu processo assim tu conseguias identificar o que tava acontecendo, entender todo processo?

E16: Era muito novo assim, e aquelas, claro eu estava com esse pacote de informações do curso, as coisas que tinha ali, eu até tinha levado um plano de parto, eu tinha colocado que eu não queria remédio sabe, que eu não queria coisas mas no final como estava indo muito devagar com a dilatação eles ficaram colocando ocitocina e por exemplo, eu não queria mas acabei aceitando sabe, porque a gente também não sabe como lidar com os médicos, não sabe se posicionar porque você pensa que são eles que sabem né, que você não sabe e isso é um erro porque a mulher sabe, a mulher sabe parir, mas você não sabe que sabe e acha que tem que deixar tudo nas mãos deles, o tempo todo, então eu senti isso depois, que eu poderia ter me, talvez me posicionado mais, talvez deixar mais claro meus desejos sobre o parto, teve momentos que eu me senti muito vulnerável assim, quando me induziram o parto foi via vaginal e sabe, eu não perguntei nada mas eu deixava que eles fizesse porque era isso né, eu também não tinha como perguntar se aquilo tava sendo certo se não, então você fica muito nas mãos deles,e você vai com uma ideia de como quer seu parto, como quer que aconteçam as coisas mas também às vezes por causa mesmo do processo do parto isso muda muito, e você não tá no controle e eu acho isso uma pena porque deveria ser um pouco ao contrário, deixar a mulher estar no controle e ali os médicos intervirem caso precisar e não ao contrário.

Eu acho que tá ainda muito ao contrário, a questão do médico que tá as coisas no controle e a mulher meio que fica quieta, deixa que a gente sabe, um pouco isso.

Entrevistadora: Uhum, sentisse isso então no teu parto, que não conseguias colocar muito teus desejos e a equipe meio que decidia as coisas, é isso?

E16: É, mas também é isso, porque a gente tem medo, a gente não sabe, é a primeira experiência né, eu acho que agora se eu fosse ter outro filho aí eu ia saberia muitas mais coisas, eu diria não, não quero isso, quero isso, sabe. Mas como é a primeira experiência você tá presa no medo, você realmente sabe que deve deixar nas mãos deles, pensa que deve deixar nas mãos deles e é muito difícil que aconteça o que você está esperando que aconteça, então mas assim eu acho que o momento foi uma experiência boa, o meu filho digamos que nasceu bem, aquela coisa do desconforto foi só no momento dele nascer porque eu demorei muito pra parir né, ele ficou muito tempo no canal de parto e parece que isso gerou alguns desconfortos nele mas no dia seguinte eu já estava lá com ele, e essa questão do pós-parto foram coisas que tempo depois com mais informação, porque o bebê nasce a gente continua na trilha do conhecimento né, aí eu já me dei conta que aconteceram coisas que também eu quisesse ter evitado sabe, porque por exemplo ele ficou na UTI naquele primeiro dia e ninguém me falou que era pra eu ir lá para amamentar ele, para tirar o colostro, sabe, não, eles deram fórmula, e eu não queria isso sabe. Então coisas assim, depois eu fiquei, é pra você sair da maternidade depois um ou dois dias depois, o bebê nasceu na madrugada de domingo e eu fiquei até meio dia da quarta-feira porque não tava conseguindo amamentar ele de uma maneira efetiva, então o pessoal lá não me dava a alta por causa disso, mas assim aconteceram outras coisas que também com mais informação talvez não tivesse permitido.

Entrevistadora: Sim, e linkando isso que tais falando da importância da informação, se puderes falar qual tu achas que é a importância do preparo pro parto?

E16: Eu acho fundamental, porque assim é uma experiência talvez única na vida de uma mulher e para além digamos das questões técnicas, sei lá, dessas questões médicas, eu acho que você precisa entender a transformação pela qual você vai passar, que não é só física, que é muito importante, não só emocionalmente, financeiramente, ninguém fala pra você que ter um filho, claro todo mundo fala que vai mudar a vida mas isso é retórico, ninguém fala como de certo sua vida vai mudar e especificamente com essa questão do parto eu acho fundamental que a mulher saiba como que isso vai acontecer, quais as opções que ela tem, que ela saiba que ela é sim capaz de parir, eu acho que isso está mudando, mas é como se uma coisa que acontece historicamente tivesse que ficar nas mãos dos especialistas que no caso são os médicos mas, nós mulheres estamos totalmente preparadas para isso, então como te comentei antes, eu acho que o processo deveria se encaminhar pro contrário, deixa a mulher que ela experimente todo o processo e caso seja necessário então que venha a intervenção médica, e não que seja a intervenção médica que decida como que a mulher vai parir, eu acho que é isso, por isso eu acho que a informação é fundamental, porque se você se sabe essas coisas você vai lá na maternidade e pode se posicionar e pode falar diretamente com as pessoas “olha, eu quero isso, eu desejo meu parto de tal forma”, por exemplo a

questão da indução do meu parto, sim eu entendo que foi pela questão que a pressão estava alta e isso realmente pode ter desfechos negativos mas se fosse por mim eu teria esperado até o momento do processo começar naturalmente, eu já estava na semana 40 mas agora que sei que poderia ter esperado até a semana 42 sabe, mas naquele momento eu não sabia, eu achava que eu já tinha completado o processo e que eles estavam certos e que era o momento dele nascer, então é isso, eu sei que tinha esse fator de risco que era a hipertensão no momento que aumentava a indução mas se não fosse por isso eu tivesse esperado até que o processo começasse naturalmente e coisas do tipo, mas é só informação que faz a diferença, se você não sabe disso você fica nas mãos do pessoal que faz um ótimo trabalho, também não estou dizendo que eles tivessem me tratado mal ou coisa para nada, mas você fica muito a mercê do que acontece lá o tempo todo, coisas que são procedimentos corriqueiros para eles, mas para gente é um momento que muda a vida né, então é isso.

Entrevistadora: E chegasse a comentar que vocês chegaram a fazer um plano de parto, tivesse conhecimento do plano de parto por onde, se quiseres falar um pouco sobre isso.

E16: Pois é, ali no curso com o pessoal do HU foi sugerido que a gente fizesse um plano, inclusive passaram um material, um modelo de plano de parto e a gente fez, a gente imprimiu e naquele plano a gente colocou o que queria, depois eu fui o mais natural possível, então eu pedi ali especificamente para só intervir em caso de necessidade, evitar remédio, eu não queria tomar epidural por exemplo e eu passei por muita dor, ou seja, só naquele momento você sabe quanta dor você é capaz de suportar. E eu senti, passei por muita dor, muita, muita mesmo mas assim, até o final eu abri a mão dessa opção de aliviar a dor com medicamento, cesárea era uma coisa que tava bem específica ali no plano de parto, eu não queria cesárea porque assim, enfim, me parece uma cirurgia muito complexa a recuperação é mais demorada, enfim, e eu tava, como a gente é estrangeiro aqui nossa rede de apoio é mínima e numa pandemia, imagina, muito mais, e eu tive que pedir pra minha mãe vir da Colômbia e ela veio em um voo humanitário porque naquele momento não tinha voo comercial então sabe, muitas coisas envolvidas que estavam ali naquele plano de parto, com a ideia de tentar fazer o mais leve possível aquele processo todo. E eu tava lá, meu plano de parto tava lá com meus documentos, com todas as minhas coisas nas pasta que a gente sempre carrega na gestação mas assim, quando eu fui internada eu pensei em esperar para passar pelo quarto e tal e meio que eu fui esquecendo do plano sabe, porque eu vi, é como se eu tivesse me entregado aos procedimentos que estavam predispostos e ninguém também me perguntou se eu tinha plano de parto, ninguém me perguntou “Você quer isso, você quer aquilo”, eu fui muito bem tratada o tempo todo, mas isso foi uma coisa que ninguém me perguntou se eu queria isso, se eu queria aquilo, se a indução eu preferia vaginal, não sei qual outra forma existe mas se tinha outra opção, enfim essas coisas então o plano ficou lá na pasta o tempo todo, eu voltei para casa com o plano na pasta, guardado, e simplesmente não sei, depois eu pensava nossa mas porque eu permiti, eu devia ter manifestado isso né, mas como a gente fica, como eu te falei, presa no medo, você não sabe o que vai acontecer os médicos às vezes eles ficam em cantos assim falando baixinho e você não sabe se estão falando coisa boa ou coisa ruim, então como que você fica condicionado ao que eles escolhem pra você.

Entrevistadora: E chegasse a conhecer a maternidade, como que foi, vocês tiverem essa oportunidade de conhecer?

E16: Não, assim eu conhecia o espaço mais ou menos por fora porque como eu te comentei eu fui várias vezes antes pela questão da infecção urinária eu sempre ia lá, eu acho que fui umas duas ou três vezes pela infecção urinária, então eu já tinha uma familiaridade com a maternidade e tal mas o espaço dos quartos, as salas de parto e tal e só conheci naquele dia eu me lembro que a [nome da profissional] e a equipe toda do grupo de gestantes passaram umas fotos dos últimos encontros como era a maternidade, quais equipamentos estavam à disposição das mulheres enfi, e tava tudo lá assim como na foto e eu achei assim, eu acho que eu pari em umas boas condições assim, que eu consegui aproveitar os recursos que tinha lá pra amenizar a dor, sei lá, porque no final das contas eu fiquei 26 horas em trabalho de parto, muito tempo, mas a equipe estava disponível, isso foi uma coisa boa assim.

Entrevistadora: Uhum que bom assim que foi assim, apesar de ter alguns detalhes pra se ajustar que pra ti quem sabe faria diferente mas que bom que no geral foi tranquilo.

E16: Sim, no geral foi, sim e eu adoro, uma coisa que eu gosto muito do HU assim especificamente da maternidade é que tem uma equipe interdisciplinar, porque bom ali na parte da maternidade estão os enfermeiros, obstetras, enfim, mas depois você recebe atendimento de pediatra, psicólogo, das assessoras de lactância, enfim, e eu acho isso fantástico porque você tem uma equipe à disposição para resolver as dúvidas assim, para fazer daquele momento uma coisa digamos mesmo impactante que é né, porque às vezes a ideia da maternidade ta muito romantizada, as pessoas acham que parir você tá ali com o bebezinho no colo e tá tudo certo, mas é bem mais complexo que isso.

finalização

ENTREVISTA 17

Data da entrevista: 16/11/2021
DATA DO PARTO: 17/06/2020

Entrevistadora: E como é que foi pra ti desde a gestação pra se preparar pro parto? Se quiseres começar falando disso.

E17: Sim, eu sou estrangeira né, eu sou Chilena, eu moro no Brasil há seis anos mais ou menos, quando eu casei, meu marido é brasileiro eu sou Chilena então dai, é a gente meio que queria se preparar e fazer muitos cursos e muitas rodas de pais e tudo isso só que, entre aspas desculpa o sotaque ta se você não entender qualquer coisa é só perguntar.

E17: A gente queria se preparar muito só que por conta da pandemia tudo mudou né, tudo que a gente queria fazer até o curso da UFSC, que eu tava super empolgada que ia ser presencial e tudo mudou de um dia pro outro, a gente teve que se adaptar às novas condições, eu tinha doula e com ela eu tava me preparando né, com a minha doula, porque assim eu sou mãe de primeira viagem, então eu não conhecia nada não sabia nada, e até as consultas com a doula eram online, então a gente teve que se adaptar a essa nova modalidade de fazer tudo online em um contexto de muita incerteza com o primeiro filho que ja traz muita incerteza, muita coisa é nova. No início foi meio difícil né, porque cada vez a gente tinha esperança que ia mudar, que ia melhorar e não aconteceu, então a gente teve que se virar com o que a gente tinha nesse momento né.

Entrevistadora: E dai tua preparação foi mais no online né como falasse e estudando assim?

E17: É eu fiz assim, a minha preparação foi basicamente o curso da UFSC que foi online, com a minha doula que foram consultas online, ai ela passou um monte de material pra eu ler e eu fiquei em casa só estudando, entrei em grupo de mães pela UFSC, pelo grupo da UFSC e por conta da minha obstetra que ela me adicionou num grupo, a minha doula também me adicionou num grupo de whatsapp, então ai tudo online tudo meio virtual sabe. E também eu fiz um curso de, com uma consultora de amamentação pra saber as coisas de amamentação e tudo isso né mas também tudo online e estudando em casa, lendo livros e material em casa.

Entrevistadora: Uhum, e as tuas consultas de pré-natal, como é que elas foram?

E17: Chegou uma época, assim tudo normal, porque quando começou a pandemia que foi março eu estava é, ela nasceu em junho né, eu tava de seis meses uma coisa assim, cinco ou seis meses, então até então tudo normal,só que chegou uma época que eu tive que fazer consulta online também, que também é bem diferente, eu não tinha nem balança em casa pra me pesar por exemplo, eu não tinha nem maquininha de pressão pra medir a minha pressão, então eu tinha que comprar ou ir na farmácia pra pagar pra me pesar ou pra que tomarem

minha pressão, tomarem, medirem não sei, por que eu não tinha como fazer isso pra acompanhar sabe.

Entrevistadora: Ah, então era assim ela pedia pra você se pesar e você se pesava em casa era isso?

E17: É, mas acho que isso aconteceu em duas consultas, depois retomou, eu acho que depois retomou presencial só que dai foi mais difícil porque tinha que ir sozinha, meu marido não conseguiu ficar por dentro de nada, nem das consultas, nem das ultrassons, tudo isto foi eu sozinha, eu acho que foram só duas ou três que foram online e o resto já foi tudo presencial, até porque acho que no finalzinho da gestação não tem como muito fazer online né, tem coisas que tem que ser feitas bem presencialmente né.

Entrevistadora: Sim, e como é que estavam as tuas expectativas pro parto, tu já tinha tido, era teu primeiro filho que comentasse né?

E17: Desculpa travou, não te escutei, as expectativas do parto?

Entrevistadora: Isso é, como é que tu projetadas teu parto, como tava teus sentimentos em relação ao parto?

E17: Bom, eu assim, o que eu tinha me preparado né, minha expectativa é que fosse um parto normal, só que eu também sabia que grande parte da situação não tava no meu controle né, a gente quer, a gente se prepara mas se na hora o bebê decidir vir de outro jeito ele vai vir de outro jeito né, minha única assim preocupação era eu ter que parir sozinha, acho que isso me gerava um medo sabe, porque eu senti que aquele suporte, aquele apoio do meu marido ia ser fundamental né pra conseguir aquele parto que eu queria, normal, sem ter a força e bom, graças a Deus deu certo ele conseguiu entrar mas eu não sei se teria sido do mesmo jeito se eu tivesse que parir sozinha porque chegou um momento que naqueles grupos de mães, até no whatsapp, até no grupo da minha doula, não estavam permitindo nem doula, nem acompanhante, nem ninguém, então tipo começou a crescer esse caos, aquela sensação de "putz, e agora a gente faz o que né". E meu filho nasceu em junho e acho que ficou pior antes e depois mas na época que ele nasceu tavam ainda aceitando doula e acompanhante, acho que antes não e depois também teve uma época que não mas bom, mas essa era minha expectativa que fosse normal, mas meu medo era que não conseguisse ter meu marido perto né.

E17: Logo depois ninguém permitiu mais nada, foi bem na época que tava conseguindo, tavam aceitando acompanhantes e doulas e graças a Deus porque depois isso mudou tudo.

Entrevistadora: Sim, e lembras assim do dia do teu parto, os primeiros sinais que ia começar a desencadear o trabalho de parto, tavas consciente disso?

E17: Ah sim, eu me lembro só que assim, isso foi meio ruim porque minha bolsa estourou e eu me lembro que foi na segunda-feira de madrugada já terça-feira na verdade e eu ia fazer

xixi e não parou de sair xixi então eu tinha lido que esse era um sinal que a bolsa tinha estourado, eu falei pro meu marido “olha, eu acho que a bolsa estourou” e ele falou “não” eu estava de 38 semanas e quatro dias, meu marido falou “não, a obstetra falou que podia ser secreções que até dava pra confundir a secreção de tanto que era né” e eu falei “não, eu tô molhando o pijama, eu to molhando a calcinha, eu tive que trocar três vezes de pijama, isso não é normal, não é secreção” dai ele ligou pra nossa doula né e eu enviei foto porque até o xixi tinha aquelas manchinhas brancas do vernix sabe, ela falou “ah, estourou a bolsa mesmo, só que o líquido está transparente” meu streptococcus tava negativo então ela falou “pode ficar até começar a evoluir as contrações e tudo isso” só que dai a gente tentou dormir um pouco porque era uma da manhã, uma e meia da manhã e daí eu ia ganhar na [nome da maternidade] só que quando eu cheguei tava super lotado, aí eu comecei com contrações mas super de leve, nada muito forte, nada, super de leve, mas eu fui mesmo porque eram sete e meia da manhã, minha bolsa tinha estourado uma e meia então já tipo, tinha que saber se tava tudo certo e enfim, só que tava super lotado, super, super, não dava pra ficar um metro de distância com ninguém e o cara que tava ali, o obstetra que tava ali na emergência que me fez um check up de tudo falou “sim, tua bolsa estourou só que você ainda não ta suficientemente dilatada, ainda vai começar a evoluir e eles fizeram um cardiotoco meu filho tava super bem só que eles não tinham como me internar porque não tinha vaga, não tinha leito, não tinha nada, eles falavam que eles queriam tipo me ajeitar ali no centro cirúrgico, me deixar ali num lugar que eu não ia ter nem banheiro, nem conseguia comer e não podia fazer nada, só pra me deixar ali porque eu tinha que ingressar só que não tinha leito e eu não sei falei “cara, meu parto não vai evoluir, não vou ter um parto normal que eu quero num lugar que eu não vou ter nem banheiro, nem vou conseguir comer, nem vou conseguir fazer nada, então dai eu liguei pra minha doula minha doula falou “não aceita isso pelo amor de Deus, liga pro teu convênio” e aí começaram a brigar e nessa briga que minhas contrações pararam né, meu trabalho de parto não evoluiu mais porque a maternidade não queria me liberar depois o obstetra foi fazer uma cesariana, depois voltou, imagina que ele só me liberou a uma da tarde e eu tinha chegado sete e meia da manhã, eles queriam fazer meu marido assinar, aí eles perceberam que eu era estrangeira, acharam que ai eu não podia entrar com acompanhante tinha que entrar sozinha, dai começou a falar que meu filho ia ter sequelas porque eu devia ficar esperando ali, ele não podia garantir que ele iria nascer bem que eu tinha que ingressar mas, eu comecei a ficar meio revoltada e chamei meu marido e falei “cara, pelo amor de Deus” meu marido falou “mas qual o problema?” e meu marido é brasileiro e mesmo assim não estava entendendo né ele falou “ela precisa ser ingressada só que não tem leito”, “perfeito, então a gente não vai ficar aqui” ele falou “não o Ilha não vai receber vocês, ninguém vai receber vocês”, meu marido falou “cara, isso não diz respeito a vocês, você tem que escrever” porque o convênio precisava de um documento assinado pelo médico que falasse que eu precisava se internar só que não tinha leito, só que o cara não queria assinar isso, acabou assinando mas eu acabei chegando duas da tarde no [nome da maternidade] e aí o cara falou “tem doze horas que você estourou a bolsa, teu trabalho de parto não evoluiu então você vai ter que fazer com indução mesmo” dai ele me falou que tinha que colocar quatro comprimidos a cada quatro horas só que consegui, cheguei a usar só três, porque dai já evoluiu já nasceu mas, não sei se tivesse evoluído sem essa briga mas por conta dessa briga tudo parou eu cheguei tinha um centímetro ou dois quando chegou no Ilha então não ia

evoluir mais e depois de doze horas o protocolo mesmo era induzir, então aí eles me colocaram primeiro as duas da tarde depois às seis da tarde depois às dez da noite depois às duas da manhã, a duas da tarde, seis da tarde e dez da noite só isso, e daí ele nasceu às quatro da manhã.

E17: Não, e o pior de tudo é que velho eu acho que foi uma violência obstétrica né, o cara me falar que meu filho poderia nascer com problema por conta disso, que eu deveria esperar, eu sinto que realmente ele queria ganhar com o parto, que ele queria ganhar o dinheiro de eu ganhar meu filho ali, de ter o [nome do filho] ali, então eu comecei a me sentir assim, que o que ele quer é que eu ganhe porque pra ele é um cheque, mais um parto, só que ele nunca olhou pra mim, nunca olhou pra questões importantes que eu não tinha um lugar, não ia ter um banheiro, estava toda molhada, tava com um absorvente pós-parto só que vazando inteiro, eu tava super desconfortável, não dava pra ficar um metro de distância de ninguém numa pandemia que a gente não conhecia muito como ia reagir a grávida e o bebê, porque ainda tava sendo estudado tudo isso, a gente tinha que ficar com essa máscara que não conseguia respirar o tempo inteiro, tudo sabe, e até a questão de um minuto né que eles me trataram como se eu não tivesse entendendo, eu falava pro meu marido "eu sou estrangeira mas não tenho problema de entendimento" sabe, até a enfermeira falava "é você não vai evoluir, você vai, quase que teu parto não vai evoluir, você não vai ter contração, você não vai dilatar" e eu "cara, vocês não tem porque falar isso pra mim" sabe, aí depois eu já não queria ganhar ali porque já não tinha gostado da vibe sabe, mesmo, nosso convênio foi super legal eles pagaram o [nome da maternidade] mesmo que não cobria, meu convênio só cobria a [nome da maternidade], por isso a gente escolheu a [nome da maternidade], e aí a gente foi super bem tratado no [nome da maternidade], não tinha ninguém, foi super tranquilo, o outro lugar tava superlotado porque também foi o auge daquelas sala de parto normal que eles tinham inaugurado a pouco tempo, então eu acho que tava todo mundo querendo parir ali né, mas eu fui super bem tratada no [nome da maternidade], tive a melhor experiência, não tinha ninguém até por conta da pandemia foi super mais tranquilo porque na sala de espera do outro hospital a gente não podia ficar, não tinha lugar nem pra eu me sentar.

Entrevistadora: Vocês já tinham cogitado outra maternidade?

E17: Assim, a gente pensou na [nome da maternidade] só pelo convênio se não eu tinha pensando na UFSC, no HU, a única questão é que a gente tinha doula e eles não tavam aceitando doula mesmo então por isso eu tipo a gente foi direto no [nome da maternidade] e porque o convênio cobria então tava super bem. Mas depois deu tudo que deu né.

Entrevistadora: Então a escolha foi principalmente pela participação das doulas né, que nos públicos não estava sendo permitido?

E17: Sim, e em tanta incerteza eu acho que ela era o meu porto seguro sabe porque eu escolhi ganhar com um obstetra de plantão porque não estava em condição de pagar minha obstetra que ela me acompanhou eu todo meu pré-natal, mas como eles cobravam a parte o parto e

tudo né, a gente não tava em condições de pagar então em tudo isso a doula virou meu porto seguro sabe, que essa pessoa conhecida né, que aliás eu não me arrependo, ela foi muito boa.

Entrevistadora: Sim, não tenho dúvidas disso né, uma pessoa preparada do teu lado é super importante, então no teu parto o teu marido pode te acompanhar e a doula? E aí como é que foi assim, mesmo no meio desse contratempo ali no comecinho vocês conseguiram desencadear um trabalho de parto?

E17: Bom, assim primeiro comprimido eles colocaram às duas da tarde, das duas às seis tudo bem, o segundo foi às seis da tarde até às oito e meia da noite tava super bem, oito e meia começaram as contrações fortes, fortes, fortes, daí eu acionei minha doula dai eu falei “olha as contrações estão ficando super fortes e vou precisar de você aqui” aí ela chegou e daí o terceiro comprimido que foi às dez da noite daí pra frente foi muito forte assim, nossa até vomitei de dor, tive muita dor, eu tinha falado com o anestesista só que sei lá, só que finalmente eu fiquei com muita dor só que fiquei aguentando aguentando, fiquei no chuveiro, na bola de pilates, doula foi me fazendo massagens, meu marido também, foi indo indo e quando não aguentava mais falei pra minha doula “pelo amor de Deus chama o obstetra porque eu acho que to morrendo” eu já tava com sete, oito aí ela falou “nem adianta colocar anestesia porque agora você vai perder tudo que você ganhou e caminha pra sala de parto que tinha que subir, descer sei lá, você vais saber o que falta” acho que tava com sete dai eu fui né, e foi sem anestesia, daí eu cheguei na sala de parto fiquei um tempo mais no chuveiro, um tempo na banheira né e eu ia, não tava conseguindo direcionar a força na banheira então eu desci e fiquei num banquinho, num banquinho de parto e eles nasceu.

Entrevistadora: Ai que ótimo, e assim né, pensando no grupo de gestantes que vocês participaram, não sei se teu marido participou também, e como foi pra vocês no preparo pro parto, influenciou de alguma forma?

E17: Sim, principalmente eu acho que informação, informação é tudo né, eu acho que empodera muito, o fato de você saber o que tá sentindo, o que vai sentir, o que vai vir, é muito, alivia muito, alivia muito saber o que vai vir. Espera um pouquinho. Tá desculpa, tive uma emergência com o bebê.

Entrevistadora: Não, imagina!

E17: Tá é que tá com os primos e ele ta jogando coisas na cabeça dos primos mais pequenos, então ta meio dificil. Mas é isso, eu acho que informação é tudo.

Entrevistadora: Uhum, na verdade assim já falasse muita coisa em relação ao teu preparo pro parto, não sei se quiseres complementar dizendo, dizendo que achas que foi a importância do preparo pro parto.

E17: Assim, principalmente acho que isso, acho que é fundamental a gente ter conhecimento do nosso corpo, do que vai acontecer, porque acho que ninguém te ensina isso isso não

aparece em nenhum lugar e muitas vezes a consulta pré-natal é muito rápida, você tem muita dúvida de muita coisa, não da pra aprofundar cada coisa né, acho que além de. (perda de conexão)

E17: Ah, sim, dai não da pra tirar todas as dúvidas né, dai a gente fica meio que tentando perguntar muita coisa e não conseguindo aprofundar em nada, então o bom é que a gente consiga entender muita coisa, do que vai acontecer e do que esperar, isso ajuda também no emocional né, porque a gente fica nervosa mais ainda numa pandemia, então ajuda pra gente se acalmar, pra gente se preparar psicologicamente também, porque eu acho que informação é isso, ajuda né, empodera né, e fica melhor quando a gente sabe o que vai acontecer, porque já é desconhecido né, já ta fora do nosso controle né então ter o mínimo de conhecimento ajuda com certeza. Por isso eu recomendo e fiz tudo quanto é curso que tinha por aí.

Entrevistadora: Isso é, e fizesse muito bem, talvez se não tivesses se preparado tanto não terias esse empoderamento de falar “não, eu não quero parir aqui eu quero parir onde eu me sinto segura”.

E17: Bom, acho que isso que fez a diferença porque eu sabia que o que os médicos estavam me falando não era verdade sabe, não ia ter, até porque além de eu ter estudado tudo isso tinha também o “pacape” da minha doula que tava falando comigo o tempo inteiro, então eu sabia que não ia, então é.

finalização

ENTREVISTA 18

Data da entrevista: 17/11/2021
DATA DO PARTO: 11/07/2020

Entrevistadora: Como é que foi pra ti desde a gestação pra se preparar pro parto? se tu puder contar um pouquinho.

E18: Ai a minha gestação no começo foi bem difícil não pela (perda de conexão) mas por conta do meu emocional, eu fiquei com o psicológico um pouco abalado porque eu ja tinha preparado minha mente pra só dois filhos, quando eu percebi que eu ia ter o terceiro eu fiquei super abalada eu disse “Como assim, como saiu do meu planejamento, como é que isso saiu do meu controle?” isso me deixou super abalada no começo, depois ficou bem tranquilo, depois que eu disse “Não, tem que aceitar, foi bem tranquilo” aí veio a pandemia, mais uma coisa que me deixou um pouco mais né, a gente sempre fica sensível na gravidez, com a pandemia não foi diferente e eu fiquei também mais sensível mas não tanto quanto eu fiquei quando eu descobri que eu tava grávida, eu sabia que ia ter modificações em várias coisas e apesar de tudo tava participando do grupo e tinha várias coisas que poderiam ajudar na parte do emocional para relaxar que era a meditação, fazer um pouco de yoga, então tinha várias outras coisas que eu fazia pra evitar um pouco essa tensão e esse estresse.

Entrevistadora: Uhum, e aí já descobrisse o grupo ali no começo da tua gestação em março né?

E18: É, eu descobri o grupo por uma amiga minha, já tava um pouquinho já, já tava um pouco acho que uns três, quatro meses já e uma amiga falou “Ai, tem um grupo de gestantes, eu participei é bem legal” aí eu disse ai eu quero participar também, aí eu consegui entrar no grupo logo no começo mas já tava, não sei se chegaram a ter presencial mas já tava online e mesmo assim foi bem legal.

E18: É já entrei no que já era online já, não cheguei a ver ninguém presencialmente, peguei na parte do online.

Entrevistadora: Uhum, e como é que foi pra ti assim, em relação ao teu preparo pro parto, quais eram tuas experiências anteriores e o que tu pensava pra essa nova experiência?

E18: Não tive uma boa experiência no segundo parto, foi um pouco difícil foi um parto normal mas foi um parto difícil e eu sempre tive muito medo do final né, da parte do parto, sempre foi uma coisa que me deixou ansiosa e só um instante. Dando uma atenção aqui.

Entrevistadora: Sim, pode parar quando precisares aí.

E18: Pronto, então quando foi na parte do parto era uma parte que eu tinha muito medo mas, com o tempo, com as conversas, aquilo foi diminuindo meu medo, porque eu vi que eu

poderia ter um parto realmente humanizado aqui, então foi uma experiência boa, eu não tive uma experiência de parto ruim, foi uma experiência muito boa.

Entrevistadora: E lembras o que tu fazias pra se preparar mesmo, tanto fisicamente, psicológicamente, se fazia alguma coisa ou não?

E18: Fiz, eu precisei, quando fechou tudo que ai não pode sair de casa pra nada, só uma pessoa, tira a roupa na volta, toma banho, isso traz uma ansiedade, uma sensação esquisita, eu lembro que me trouxe uma sensação de me sentir presa, só que eu fui trabalhando o meu psicológico de que "não, você não ta presa, você pode sair a qualquer momento se você quiser, mas só que você não pode porque no momento isso traz um risco pra você, então eu fui trabalhando o meu psicológico, eu usei muito meditação no youtube, yoga, fiz muita yoga do youtube, tinha também eu lembro que passaram no grupo uma cartilha de exercícios também pra fazer em casa, que eu acho que também eram exercícios de yoga mas eram exercícios pra próximo do parto, ou desde o começo, acho que desde o começo da gravidez ja podia fazer assim, lá depois dos quatro meses é claro, eu fiz esses exercícios e esses exercícios me acalmavam muito, principalmente quando eu fazia assim meditação com a yoga, então eu fazia os dois, fazia a meditação e depois alguns minutinhos de exercícios pra yoga.

Entrevistadora: Sim, e aí chegasse a fazer o plano de parto também.

E18: Fiz, fiz o meu plano de parto e levei no dia.

Entrevistadora: Se quiseres contar um pouquinho do dia do teu parto mesmo, como é que foi os primeiros sinais, como é que tudo aconteceu?

E18: É então, no dia do parto, no dia anterior o dia 10 eu tava, eu ainda tava trabalhando e eu lembro que eu comecei, aí uma gravidez que eu mais fiquei ansiosa, eu lembro que eu comecei "ai amor, já passou 39 semanas e o bebê ainda não nasceu" e ele "calma que ele tem o horário dele, calma que ele tem o horário dele" e aí quando foi no dia 10 eu lembro que eu comecei a sentir uma contrações logo de manhã cedo aí eu disse "eu acho que o bebê vai nascer hoje" e aí foi aumentando durante ao longo do dia, como eu já tive né, experiência do segundo filho que foi normal eu disse "ah, vou esperar quando ficar mais próximo cada contração" e aí quando foi no final da tarde, acho que umas cinco, seis horas eu resolvi mas ainda não tava na hora, ainda tive que ficar lá no hospital um tempo, ele falou que tava com a dilatação mas eu precisava um pouco mais de dilatação aí eu disse "ta bom", ele me pediu pra ficar fazendo caminhadas que caminhadas sempre aceleram o parto, ai ta fiz essas caminhadas, fiz que eu não aguentava mais de tanto caminhar, eu só queria que acabasse logo. Caminhei , caminhei, eu lembro que esse dia tava um dia bem agitado no HU, tava um dia bem agitado no HU e eles estavam pensando em me transferir pro Carmela, aí eu falei como eu já tô aqui não quero ir mais pra lugar nenhum, quero ficar aqui, mas eu lembro que deu tudo certo, eu consegui ficar lá, liberou o quarto pra eu ir e logo depois quando ele viu que eu consegui, ai eu não lembro quanto tem que chegar pra subir pro quarto, não lembro

quantos de dilatação acho que 8, só se sei que eu logo subi pra finalizar o processo lá no quarto, e aí como já tava nessa parte bem restrita meu marido não pode subir comigo, ele teve que ficar lá embaixo me esperando enquanto eu fazia essas caminhadas, ele só pôde subir pra acompanhar esse processo do parto quando eu já fui pro quarto, ai sim que ele pode subir pra acompanhar, aí ele se trocou e ele subiu, fizemos o processo deixamos as coisas e fomos pro quarto, troquei de roupa e fomos pro quarto e lá no quarto eu já tava tão cansada de andar que eu só queria eu falei "ai eu posso escolher o método que eu quero que o bebê venha? eu quero deitada que eu já to cansada", aí eu fiquei esperando, sentindo as contrações esperando, aí veio a enfermeira né, a enfermeira me acompanhou tudinho, conversou comigo, foi bem prestativa a equipe que eu peguei foi bem maravilhosa comigo, foi bem atenciosa e eu falei pra ela que meu segundo parto tinha sido com ocitocina pra agilizar o processo né, e ela falou "ah, mas esse não vai precisar, vamo lá, vamo continuar o processo e você vai ver que esse não vai precisar" e realmente não precisou, ai teve um determinado e eu tava deitada ela falou "vamos acelerar mais esse teu, essas contrações, vamo acelerar mais a vinda do bebê?" ai ela disse "vamo lá no chuveiro, senta na bola e pega um pouco de água no corpo, você vai ver que isso vai ajudar bastante" e realmente ajudou muito, quando ela pediu pra ir no banheiro com essa bola para que foi uma coisa, ajudou mais ainda, quando eu voltei ele já veio meio sair, já rompeu mesmo pro trabalho de expulsão depois disso só mesmo já veio acho que o doutor, tinha o doutor que tava só acompanhando, quem fez o parto (perda de conexão) processo, aí o doutor ficou assistindo, tinha uma ou duas pessoas na sala, não sei se era enfermeira ou estudantes também, eu tava com tanta dor que eu não lembro e foi, não foi demorado não, depois da parte da bola foi bem rápido, eu lembro que ele veio logo, eu lembro que teve uma hora que o doutor disse "tá sentindo contração? porque tu tá fazendo a parte de, empurrando o bebê" eu disse "não doutor, é que tá doendo, só quero que ele nasça" (risos) e depois ele veio, nasceu meu bebê, aí logo em seguida colocaram ele no meu colo pra ele ficar no meu quentinho, ficar só sentindo aquele bebezinho gostoso e já, eles falaram pro meu marido se ele queria cortar o cordão umbilical ele já tava traumatizado ele não queria mais, eu já tinha conversado com ele em casa eu disse "amor, tu vai querer cortar o cordão" ele "vou, vou cortar" quando chegou lá ele desistiu ele "não não, eu achava que aquilo ia te machucar mais, eu não quis" aí depois eles cortaram o cordão umbilical, houve laceração fizeram uma sutura e depois o doutor saiu ficou só estudante fazendo a parte da costura, bem tranquilo, ela foi também bem prestativa, fez a anestesia local e falou que se tivesse doendo era pra eu falar pra ela, teve pouco incômodo e ela fez bem, com bastante calma, foi bem tranquila também essa parte e depois foi só o bebê pro meu lado, do meu ladinho naquela parte que ele fica bem quentinho, foi tudo bem tranquilo. A, logo em seguida perguntaram se eu queria comer alguma coisa, se eu tava com fome eu disse "a, quero sim" tava morta de cansada, morta de fome, tinha trabalhado um monte que já era desde a manhã, isso era madrugada do dia 11, ele nasceu acho que três, não uma e meia eu acho da manhã, três horas era o horário que eu já tinha tomado banho, que eu também tinha o que comer, já tinha tomado banho, parto normal é bom por isso, porque depois você já pode tomar um banho, você pode ficar mais à vontade pra comer e enfim.

Entrevistadora: Uhum, e chegasse a se alimentar e tomar água no teu trabalho de parto ali?

E18: Não, eu nem me lembrei se eu pedi alguma coisa, eu acredito que eu nem pedia, só queria fazer aquela parte, não tomei e não comi.

Entrevistadora: Sim, mas e como é que foi pra ti né que comentasse que teu companheiro só pode entrar na hora do expulsivo mesmo né, ali quando ja tava na hora de sair o bebê praticamente né, como é que foi pra vocês?

E18: Se eu te disser que eu fiquei feliz porque pelo menos ele tava do meu lado, vi que foi mais difícil ir pro quarto sem ele, porque não podia mais ir pro quarto, foi na época que não podia ter mais nenhum tipo de acompanhante mesmo no quarto, foi mais difícil ir pro quarto sem ele do que essa parte, essa parte pelo menos eu falei "ai mô, pelo menos tu tava aqui", agora a parte do quarto assim que foi mais triste essa separação, ai porque a gente precisa de uma certa ajuda no quarto, nem que seja "olha o bebê", porque a gente tem essa preocupação "ah fica aqui olhando meu filho" quando alguma coisa ruim aconteça, sei lá ele se engasgue, alguém sequestre, a gente vai, a mãe a gente pensa em todas essas loucuras mas é ruim essa parte do quarto de não ter ninguém, agora eu falei "pelo menos tu tava aqui do meu lado nessa parte do trabalho de parto" que é bom até ele verem, é importante eles verem o que a gente passa e o nosso terceiro filho foi o único filho que ele pode assistir o parto, os outros dois ele não teve essa oportunidade, e foi bom pra ele ver e ver como é esse processo, que não é um processo fácil, dar valor pra gente, eles acabam nos valorizando mais pelo menos o meu me valorizou muito mais depois desse parto do que ele valorizava antes, ele já me valorizava agora ele valoriza muito mais, que ele já sabe que não é fácil trazer uma criança, nascer, gerar um criança, dar a luz, não é fácil. E aí depois o quarto foi mais difícil, eles deixaram a gente bastante tempo nesse quarto se recuperando, deixou ali tocar no bebê, abraçar o bebê, só que depois chegou um momento da gente ir pro outro quarto, tipo um alojamento aí ele teve que ir, nessa parte foi triste, mas ainda bem que não foi tão demorado, a gente só passou um dia no hospital, porque tem pessoas como é o primeiro filho que acabam ficando mais tempo, tem pessoas que têm cesárea que tem que carregar o filho e não é nada fácil, ainda bem bem que eu tinha a oportunidade de ficar pouco tempo e pra mim não era muito difícil carregar o bebê porque eu não tinha feito cirurgia, então foi bem tranquilo o final, esse final não foi tão difícil pra gente só mesmo essa parte dele não poder ficar com a gente no quarto.

Entrevistadora: Uhum, e agora pensando um pouco ali de novo no teu trabalho de parto, no teu parto, em algum momento sentisse dúvida de alguma coisa ou não tava entendendo o que tava acontecendo ou tu conseguia ter consciência de todo processo, entender cada etapa?

E18: Essa foi uma parte boa, eu consegui ficar consciente de todo processo, meu maior medo era que fizessem a episiotomia como fizeram no meu segundo filho, sem me perguntar, não tive aqui ta eu tive em outro Estado, só que eu achei muito ruim porque nem me perguntaram só cortaram né "ah, vamo cortar aqui pra acelerar" não né, nem me perguntaram, só cortaram, e meu maior medo aqui era que fizessem de novo a episiotomia sem o meu consentimento só que não, foi bem tranquilo, quando precisava, eles devem ter me auxiliado mas como eu te

falei essa parte foi bem a parte do final assim, uma parte que a gente não pensa muito mas eles foram bem, não tive nenhuma dúvida, fui bem auxiliada.

Entrevistadora: Sim, e sobre o grupo de gestantes, qual tu achas que foi a influência do grupo no teu processo de se preparar mesmo pro parto?

E18: Olha, muitas coisas do grupo mesmo sendo terceiro filho eu nem sabia. Plano de parto então, gente, o que é plano de parto? Eu não ia saber nunca na vida o que é um plano de parto, eu fui saber pelo grupo, aí que eu fui pesquisar e falei "olha, existe plano de parto" aí nisso também teve outras coisa que eu aprendi lá no grupo que passaram, teve muitas coisas legais, livros, a pega do bebê que eu ja ia começar a fazer que ele tivesse pega errada mas ai lá no grupo teve um auxílio legal também sobre a pega do bebê no peito, deixa eu ver o que mais, eu gostei muito, o grupo auxiliou de todas as formas, por exemplo teve uma parte no final do meu parto que eu tive uma dúvida dai eu procurei a enfermeira no privado e ela me ajudou bastante também, eu acho que eu tava começando a ter os sinais do parto, e eu não tava associando com sinal do parto e acho que passei uma semana assim, com dor de barriga mesmo assim e tava tendo diarreia e eu "sei lá, isso é normal?" ela falou "olha, provavelmente isso é um sinal do parto" eu nem imaginei que isso poderia ser um sinal do parto, ela também prontamente respondeu minha dúvida e o grupo acho que ele é realmente pra isso, ele é pra tirar suas dúvidas, ele é pra te dar o apoio umas às outras, contar a experiência como ta sendo e te orientar nas coisas que você não sabe, eu não sabia muitas coisas e eu fui ver que eu não sei onde é que eu tava pra não receber essas informações.

Entrevistadora: Uhum, que bom né que conseguisse receber essas informações.

E18: Acabou que eu não fiz laqueadura, porque quando a gente colocou os papéis pra fazer vasectomia como é período da pandemia se a espera de três, cinco, a espera aumentou por um bom tempinho que ainda não saiu.

Entrevistadora: Em relação ali a escolha da maternidade, vocês chegaram a escolher a maternidade antes de ir pra maternidade?

E18: A gente chegou a conhecer só pelas fotos e vídeo que foram apresentados no grupo, que não podia fazer visita por causa da pandemia e aí apresentaram as fotos no grupo pra gente poder conhecer né, que antes falaram que poderia fazer as visitas né com a gestantes só que por conta da pandemia não da, ai a gente viu nos video e fotos que foram apresentados.

Entrevistadora: Uhu, e pra finalizar assim né, num geral, qual tu achas que é a importância que é a principal questão né, a importância do preparo pro parto, hoje na tua experiência assim olhando tudo que tu já passou?

E18: É, a gente pode até pensar que tá preparada mas acho que mesmo a pessoa mais preparada do mundo ela precisa de um grupo desse onde ela se sente inserida, onde ela vê que tem outras pessoas que estão lá pra te dar apoio, como foi o caso que eu falei com a

enfermeira, mas também pelas orientações que a gente tem, acho que é muito importante a gente trabalhar essa parte em grupo. Olha, nunca tive a experiência de “ah, vou conhecer a maternidade antes de ter o bebê” eu não tive essa experiência antes, mesmo por fotos “vou te apresentar mesmo que for por fotos” eu não tive essa oportunidade, já cheguei pra ter todos os dois e isso é bem, tanto por exemplo a orientação pra exercícios e até mesmo porque conhecimento nunca é demais “ah eu já tive" se fosse por exemplo “ah não, já tive dois filhos, eu não vou entrar nesse grupo” não, eu vou entrar, eu preciso mais ainda porque ja fazia cinco anos que eu tive meu segundo filho eu disse “não, eu vou entrar, pode ter mudado alguma coisa, tem coisas que eu não sei” orientação a mais nunca é demais, podem ter coisas que no grupo deve ter passado lá que eu ja sabia mas a gente fica na nossa, fica guardado e pega a orientação de novo, pra mim não tem problema e aprendi muita coisa que eu não sabia do plano de parto por exemplo é uma delas, que eu podia aprender com o grupo e foi muito bom, não tenho o que reclamar do grupo, pena que a gente não conseguiu fazer presencialmente nenhum encontro, só a única coisa que eu fico triste é esse encontro que a gente não pode ter, assim de se conhecer pessoalmente, mas tive uma experiência com uma pessoa que tava no grupo que teve o bebê e ela falou”ah, você ta no grupo de gestantes? o grupo de gestantes tal” e eu disse "tô" acho que é 97 o nosso “eu também, o meu nome é fulana” eu não lembro agora “meu nome é tal” e eu “prazer” ela tava la no mesmo quarto que eu, então teve uma pessoa que eu pude conhecer, o bebê dela nasceu com problema eu não lembro qual foi e teve que ficar internado e ela tava lá, mas eu tive oportunidade de conhecer alguém do grupo la na maternidade, teve o bebê bem próximo do meu.

finalização

ENTREVISTA 19

Data da entrevista: 19/11/2021
DATA DO PARTO: 02/08/2020

E19: Eu pari dia 02 de agosto de 2020, então ela ta com um ano e três meses agora.

Entrevistadora: E como é que foi assim pra ti desde a gestação né, se conseguires relembrar lá do começo o teu processo assim de preparação pro parto?

E19: Então, eu acho que essa questão eu refleti muito durante todo o processo da gestação e depois principalmente nos grupos assim né, eu participei do grupo da UFSC e também eu fiz parto domiciliar então eu tinha outro grupo da preparação pro parto domiciliar né, então eu tive meio que duas preparações assim e eu refleti muito sobre a questão de que as pessoas gastam muito tempo na questão do enxoval, de preparar o quarto, de preparar toda essa parafernalha que no geral a gente não usa e não se prepara emocionalmente né, porque pra mim foi muito difícil o começo da gravidez assim os quase quatro primeiros meses foram bem difíceis assim, tava com uma depressão bem intensa assim e aí fiquei me tratando com psicóloga e comecei a tomar remédio também, tomo desde então. Então, essa parte de lidar com a parte hormonal assim né, as mudanças, eu não me reconhecia eu não sabia o que tava acontecendo com o meu corpo né, isso foi muito assustador, foi horrível essa parte e por mais que, que eu soubesse que eu ia passar por aquilo parecia tipo, era algo desconhecido né, então foi bem, eu acho que a palavra melhor é assustador mesmo né, mas ao mesmo tempo tinha seus lados bons né, ver ela mexer, conversar com ela e ela responder, tipo eu falava em ela e ela mexia era muito gostoso esse processo assim e eu acho que preparar pra essas questões das oscilações o puerpério assim tipo, saber que eu ia ter esse momento pós-parto também foi bem importante né, acho que a preparação emocional foi o que mais, o que mais fez diferença pra mim nesses grupos né e o parto em si eu tinha com a questão da pandemia né, nesse processo do parto, eu pensava em ter em hospital e coisa e tal quando eu pensava na possibilidade de, na época que eu pensava na possibilidade de engravidar, ai me vi grávida na pandemia, pensar em hospital "e se acontece alguma coisa comigo? e se eu pego covid? e se ela pega covid?" tipo aquela, porque tava muito incerto né naquela época, a gente não sabia nada sobre nada e aí pensar no parto domiciliar foi meio tipo "tá, é mais seguro tá em casa né" tipo nesse sentido, mas eu me sentia muito insegura de "ta, e se acontece alguma coisa comigo ou com ela, a gente não tá no hospital" tipo "como é que lida com isso?" então tipo foi pesando assim, o que que era mais seguro pra esse processo e aí a gente decidiu, conversou bastante né, teve essa preparação com o grupo do parto domiciliar também e decidiu ter em casa por isso, ela falou que as intercorrências são muito pequenas assim de casos, no parto assim e eu me preparei muito pra, pra tipo saber que eu dava conta assim, eu acho que a melhor, a melhor preparação é isso, de saber que eu dou conta, dou conta se tiver que ir pro hospital, dou conta se tiver que, eu queria parto normal né, eu consegui, mas também me prepararei pra ter cesariana, me preparei pra ir pro hospital, me preparei pra essas possibilidades assim, eu não sonhei, eu acho que tem diferença sim, penso né, que tem muita

diferença entre as outras mulheres que eu conversei assim de idealizar como que vai ser seu parto assim, eu não idealizei sabe, eu tinha uma insegurança muito grande porque eu tenho questões com o coco assim, e aí tinha uma insegurança muito grande de fazer coco na hora do parto, que é bem comum né, e aí a enfermeira que me atendeu que fez o meu parto falou "ah, isso é muito comum assim" e eu pedi pra ela pra ver se a gente não podia fazer uma lavagem e coisa e tal pra não acontecer dai ela falou "Olha, eu fiz lavagem no meu parto e mesmo assim aconteceu né, então tipo eu não recomendo mas se tu quiser a gente faz" e eu tipo tá, vai ser uma coisa que eu vou ter que enfrentar assim, então eu tive esse momento também de me preparar pra isso, de lidar com essas questões né, tipo meio que tabu pra mim, de eu fazer coco na frente de outra pessoa e eu me senti muito acolhida assim nesse processo, ter esses grupos, de ter as dúvidas, das angústias poder compartilhar foi bom assim, uma coisa que fez muita diferença pra mim e acho que faltou um pouco nos grupos de preparo é a questão da amamentação no sentido de, de ponderar todas as questões que envolvem a amamentação sabe, porque não é só tipo "ah o leite é importante e rico pro bebê, essencial e tudo mais" sim é, mas todo desconforto da mãe, todo trabalho porque é um trabalho de amamentar tipo não foi muito preparado do quanto que eu ia não é sofrer, sofrer é uma palavra forte mas do quanto que eu ia me desgastar né, amamentando, eu acho que essa questão é difícil e eu acho que naturalizam muito também a questão da amamentação como se fosse uma coisa que a mulher e o bebê sabem fazer assim e não é bem assim, eu fui numa consultora de amamentação depois que minha bebê tinha um mês porque eu e meu companheiro a gente queria dividir, fazer amamentação compartilhada, e aí eu queria ver o melhor jeito, se era mamadeira, se tinha outras coisas, dai ela apresentou a sonda dedo a gente deu durante uns três meses com sonda dedo depois passou pra mamadeira, a gente tentou copinho, tentou colher dosadora, foi tentando se adaptar assim e quando a gente foi na consultora de amamentação eu descobri que tava com hiperlactação assim, tipo e ela falou "ah, tua filha é guerreira assim, do tipo que tens leite assim jorrando, não sei como é que ela não se afoga, não sei como é que ela tá conseguindo mamar" e coisas que eu não tinha ideia assim, eu imaginava, eu só sabia na verdade não imaginava, eu só sabia da falta de leite não do excesso né tipo, então coisas que eu não sabia muito lidar, as posturas da amamentação no sentido de pensar mais no meu conforto e também nesse, por exemplo, a questão da hiperlactação eu quanto mais inclinada era melhor e ela me deu umas dicas nesse sentido assim, eu senti que principalmente no grupo da UFSC assim, tinha uma pegada na questão da amamentação muito pensando só no bebê assim e não pensando na mãe e no bebê, porque ta a gente sabe da importância e eu acho que é importante reforçar o aleitamento materno mas ver as condições dessa mulher de amamentar é uma coisa essencial né, porque fica uma culpa se a mulher não consegue amamentar, não consegue dar conta porque é tanto trabalho que ela tem né, de cuidar do bebê e de outras coisas, de cuidar da casa e de fazer um monte de cosias, muitas vezes, eu pude né meu companheiro dividiu realmente as coisas comigo durante o puerpério tipo os quarenta, mais do que o puerpério né, os quarenta e poucos dias ali de resguardo né, ele que fazia todas as coisas assim com ela sabe, então eu tinha esse apoio mas a gente sabe que a realidade é que a maioria das mulheres não tem, então amamentar é muito trabalhoso é tipo, eu amamentei, eu tirava leite pra ele dar, ela amamentou exclusivamente com o meu leite até os seis meses depois disso a gente começou a introduzir fórmula porque eu não dava conta, tava muito cansada e hoje ela toma basicamente fórmula assim.

Entrevistadora: E já chegasse a falar um pouquinho sobre a influência ali do grupo de gestantes né, mas como conhecesses o grupo, como foi esse processo e o que realmente influenciou no teu parto?

E19: Eu conheci o grupo porque uma amiga minha já tinha participado né, daí ela me indicou assim é, eu assim, na questão do parto em si, bom na verdade como eu tive a questão do parto domiciliar ai meio que eu participei dos dois grupos, eu não vou conseguir lembrar exatamente assim tipo de mais direcionado pro parto sabe, mas assim, eu lembro tipo do grupo trazer várias questões nesse sentido, que tu falou, de reforçar as questões ideais vamos dizer assim, a questão do parto normal tipo de optar porque muitas mulheres ficam com medo de parir e aí optam pela cesárea só por medo, não por alguma situação necessária pra ela ou pro bebê e também lidar com essas questões assim tipo de que não é assustador, não é assustador não, é que a gente tem que enfrentar né mas, não é essa coisa horrorosa tipo pra mim de toda minha maternidade a experiência mais legal que eu tive foi o parto assim, tipo eu tenho uma lembrança muito boa sabe, pra mim eu me senti renascendo mesmo é uma entrega muito, muito única assim, eu não teria, eu não penso em ter outro filho de barriga assim mas eu queria muito parir de novo, a experiência foi muito boa.

E19: Nossa, é difícil ouvir isso, é raro uma mulher dizer, querer parir novamente, muito legal, porque realmente se é uma experiência vivida integralmente é transformador, como falasse ali renascesse né mas, antes de falar um pouquinho do teu parto se quiseres falar né, lembras como é que foi tuas consultas de pré-natal se teve alguma influência da pandemia?

Entrevistadora: Eu fui atendida ali no posto da trindade, meu pré-natal foi todo ali e na verdade tipo eu acho que teve uma ou duas que não foi todo mês assim que a enfermeira e a médica meio que optaram, deixaram aberto pra gente, se a gente sentisse a necessidade né de ir ia né, e eu acho que a gente teve uma ou duas que pulou mas no geral continuei indo certinho tipo todas as consultas, fiz todos os exames, ali não alterou muito essa questão assim tipo, eles meio que deram uma prioridade pras gestantes, não sei se aconteceu isso em todos os postos né mas ali teve até uma época que eles criaram, separaram assim, fizeram uma parte isolada assim só pra gestante mas o pré-natal foi ótimo assim nesse sentido.

Entrevistadora: Falasse do teu parto que foi domiciliar planejado, mas chegasse a fazer um plano de parto?

E19: Na verdade assim, eu acho que não exatamente a gente conversava, não cheguei a escrever sabe mas eu conversava muito com a parteira de tipo, das coisas que eu pensava, tipo ela colocava algumas sugestões assim tipo desmistificava algumas coisas tipo, por exemplo, uma coisa que ficou muito marcada pra mim que elas me falaram assim foi que tipo a tem sempre uma ideia que depois do parto tu vai ver o bebê e vai se apaixonar loucamente e querer tá com o bebê, tipo meu Deus amor da minha vida assim, e ela falou que muitas mulheres tipo não, mei que tipo ai ta fazendo aquele esforço todo e tipo “Deu, nasceu, deu, tipo não quero, tô cansada, né” e é isso, ela falou que acho que não criar esse ideal também

que tu vai se apaixonar nesse primeiro momento também foi muito importante assim pra mim né, de pensar o parto, eu pensava na questão das posições tipo, uma das coisas que eu pensava assim, mas como eu te falei eu não tinha um ideal e aí eu tinha dúvida assim de qual que era a melhor posição, tipo mais confortável coisas nesse sentido e aí parteira falava "ai, isso na verdade vais é muito do momento, é muito instintivo assim, é como tu vai se sentir confortável, tem mulheres que parem em pé, tem mulheres que é de cócoras, deitada, enfim, cada uma vai de um jeito assim" e aí foi eu realmente consegui sentir e fui muito respeitada assim no meu processo né, isso também fez muita diferença assim, é uma das coisas que eu penso é como seria no hospital assim, porque tipo eu vivi muito meu tempo, tipo assim "Ah, agora eu quero deitar. Agora eu quero tomar banho. Agora eu não quero que ninguém fale. Agora eu quero escutar música." tipo eu fiz tudo no meu tempo, em como eu fui sentindo meu corpo assim né, então não sei se no hospital, eu já escutei vários relatos que tem uma pressa então acaba não sendo tão no teu ritmo assim, e aí isso é uma das coisas que eu também eu acho que também por eu ter uma boa lembrança, um dos fatores de eu ter uma boa lembrança.

Entrevistadora: Mas como que foi assim no dia do teu parto, lembras dos primeiros sinais, como é que acontteceu?

E19: Eu comecei no dia anterior a ter contração de manhã cedo assim e inclusive eu tenho um relato de parto, se tu quiser eu te mando. É, aí eu comecei no dia anterior a sentir começou de manhã e aí ficou aquela coisa inconstante assim né, senti um pouco no meio da tarde e aí de noite deu uma parada, dai no dia seguinte de manhã cedo começou e aí foi bem gradativo assim, uma das coisas, meu parto foi no domingo, na sexta-feira a gente teve consulta com a parteira pra tipo conversar sobre como, o que poderia acontecer assim, como poderia ser e aí outras coisas que ela desmistificou e eu não lembro se foi falado sobre isso no grupo lá da UFSC né de que essa questão das 42 semanas, tipo pode ser a mais, tipo é importante é ficar monitorando o bebê ver como é que tá a mãe mas não é uma coisa que passou de 42 semanas é obrigada a tirar o bebê, tipo né, tem que avaliar cada situação é uma situação, tem bebês que nascem depois mesmo e que o trabalho de parto pode demorar bastante tempo no sentido de as contrações podem começar e tu ficar dias nessas contrações assim, coisas que eu não imaginava assim, porque sempre que relatam é como se tipo começou as contrações, daqui a pouco vai estourar a bolsa e daqui a pouco vai… e tem gente que não estoura a bolsa, várias coisas que eu não fazia ideia assim, e aí eu comecei de manhã cedo, fiquei o dia todo assim tipo, ai isso umas seis sete horas da manhã assim, aí fiquei o dia todo tendo contração leve até umas duas três da tarde assim começou a intensificar e eu comecei a realmente me preparar ajeitar as coisas, eu queria gravar, bater foto, ai fui esvaziar meu celular, pensar nas questões tipo de música que eu queria ta escutando, preparar realmente as coisas que elas pediram de pano, lençol e essas coisas assim e aí fui organizando com minha mãe e meu companheiro essas coisas assim e aí depois lá por umas quatro da tarde eu ja tava meio que imersa, ai eu já não tava mais pensando em nada, eu tava lá vivendo o momento assim, daí eu fiquei escutando música e me movimentando, uma hora eu andava depois eu deitada, dai tipo a parteira me recomendou o quatro apoios pra fazer por causa da lombar né, dava muita dor na lombar e posição tipo agachada, ela falou "ah, essa posição

pode ajudar” então eu fui tentando, ai toma banho tipo, ai elas foram indicando coisas que poderiam ajudar na questão da dor e aí às sete e pouco a minha bolsa estourou e eu fui ter ela dez e vinte e cinco da noite, foi rápido comparado a outros partos e foi bem gradativo assim, bem processual.

Entrevistadora: Se quiseres trazer alguma coisa que elas falavam assim em relação ao parto que tu achou importante?

E19: Eu acho que essa questão de desmistificar alguns ideias assim é o principal ponto, sabe, tipo por exemplo a questão da dor, a lógica de sentir prazer durante o parto não é uma coisa trabalhada e muitas mulheres sentem né, eu acho que esses pontos não são muito falado, não pra criar um ideal né, porque dai as mulheres podem ir pra um caminho tipo “Ah, eu preciso sentir prazer durante o parto” e não né mas, pra ver que não é uma coisa assim nossa, porque muitas mulhere seu acho que não tiveram um parto bom muito por essa questão de não ter seu tempo respeitado sabe, e tipo por exemplo a minha mãe quando me pariu né, ela disse que tipo tinha um monte de gente em cima dela e cada um dizendo um negócio tipo “Ai, respira assim. Ai anda” tipo as pessoas indicando o que que ela devia fazer e não ela sentindo o que que ela devia fazer, o sentir o próprio corpo eu acho que é uma coisa essencial assim. Mas também tem um ponto de autoconhecimento assim né, eu acho que eu não sei o quanto que só a informação muda tudo isso, tipo porque como eu te falei eu tinha muito conhecimento sobre o meu corpo então foi muito assustador o começo da gestação porque eu não conhecia mais meu corpo né tipo “O que que é isso que eu to sentindo, o que é essas sensações, isso tudo é novo assim” e tem pessoas que não se dão conta assim do que tão sentindo de como que ta, de saber como é o funcionamento do corpo assim né, então acho que tem vários pontos né, mas eu acho que desmistificar esses ideais eu acho que é o principal sabe., trabalhar tipo por exemplo, trabalhar que às vezes tem que ser cesárea né, pra mulheres que idealizam um parto normal e “eu preciso ter parto normal” assim como o contrário, pra mulheres que “não, meu Deus eu vou morrer, tipo de dor, não quero passar por isso” que na verdade o parto normal pode ser prazeroso, pode ser um momento bom e todos os benefícios né, tanto pra mulher quanto pra criança né do parto normal, ter os dois contrapontos de tudo assim pra tá preparada pras duas situações.

E19: Só pra completar essa questão que tu falou de ressignificar o parto que eu fui parida assim, a minha mãe participou eu parindo né, e ela também tava muito insegura assim e aí elas, as parteiras conversaram com ela assim, do que que é essas angústias, quais os medos, quais os receios e tiipo se colocaram a disposição pra ela também, de tá ali ali tá todo mundo meio que junto pra se dar apoio assim, eu acho que se colocar nessa escuta ativa também é importante assim tipo, acho que escutar a mulher é uma coisa essencial nesse processo de construção do parto, às vezes vão ter angústias que a gente nem imagina né tipo, não sei se essa questão do coco é uma coisa comum de ser trazida assim, no geral eu acho que as mulheres se preocupam com outras coisas e eu.

*finalização**

ENTREVISTA 20

Data da entrevista: 22/11/2021
DATA DO PARTO: 26/08/2020

E20: A [nome da filha] nasceu no dia 26 de agosto de 2020, ela nasceu na Carmela e dai mas na verdade ela era pélvica (perda de conexão)

Entrevistadora: Aqui travou um pouco.

E20: Travou né, aqui tá caindo ta meio instável pra mim. Dai a bolsa rompeu e agente foi pra Carmela, só que foi cesárea, porque ela tava pélvica.

Entrevistadora: E como é que foi pra ti desde a tua gestação em relação ao preparo pro parto, tu lembras o que tu fazia pra se preparar?

E20: Sim sim, é que na verdade eu queria muito o parto normal né, eu acho que com 30 semanas o grupo foi um pouco antes eu acho que eu tava com 24, 25 semanas mais ou menos eu não to bem lembrada qual o tempo da gestação que eu tava né e eu queria muito o parto normal só que dai logo em seguida, acho que com 30 semanas eu já descobri que ela tava pélvica, eu fiz acupuntura, andei de cabeça pra baixo (risos), fiz de tudo pra tentar ela virar mas não teve jeito. E acho que umas duas semanas antes dela entrar ela deu uma boa movimentada na minha barriga e eu pensei agora ela virou, mas não virou não.

Entrevistadora: Ela quis ficar pélvica mesmo.

E20: É, porque eu sempre achei bem interessante, eu sou enfermeira também né, então desde a faculdade foi algo que me chamou bastante atenção e achava bem legal assim sabe, parecia que passar por isso seria, apesar de ter as dores, sei que não seria fácil a questão das contrações tudo mas, eu gostaria muito de ter passado, só que ao mesmo tempo o fato de eu saber que já ia ser cesárea me tranquiliza do que nos casos de às vezes ter tido todo trabalho de parto, todo preparo e de repente ter que necessariamente ir pra uma cesárea sabe, eu acho que ali talvez eu ficaria mais frustrada, como eu sabia que seria uma cesárea eu fui mais preparada então eu sentia a contração, a bolsa rompeu e isso pra mim foi fundamental sabe, pelo menos teve todo esse processo mesmo, me senti bem mãe (risos).

Entrevistadora: Como foi pra ti conhecer o grupo de gestantes?

E20: Então, mesmo sendo enfermeira eu trabalhei na gestão da prefeitura, tava na coordenação a bastante tempo então eu tava um pouco longe da assistência, um ano antes de engravidar eu voltei pra assistência então eu tava relembrando a questão do pré-natal, gostava bastante, e eu quis muito fazer o curso pra ser esse outro lado sabe, me permitir ser cuidada também né, então receber orientações sim, relembrar orientações porque por mais que a gente fale, quando a gente tá no outro lado tem uma outra visão né. Então eu achei bem legal, desde

a graduação, eu fiz na UFSC também e eu já existia o grupo de gestantes então a gente teve uma participação no grupo e eu achei muito legal, achei sinceramente incrível assim o trabalho que era feito e gostei eu ainda lembro que assim que eu passei no concurso eu fiquei seis meses só na unidade e eu tentei fazer grupo de gestante porque eu tinha o grupo do HU como base assim e infelizmente na unidade não tinha adesão depois eu vim pro Campeche a gente teve a mesma dificuldade de tentar fazer o grupo e não ter a mesma adesão, porque mesmo que tu vincule hoje em dia a gente não tem mais um dia te atendimento de gestante né mas era muito difícil de conseguir a participação delas mesmo e eu sempre fui bem encantada então eu falei "Ah não quando eu engravidar então pelo menos comigo eu vou conseguir fazer".

Entrevistadora: Que legal, e como é que foi pra falando como mãe, a influência do grupo assim nesse teu processo, o que que tu achas assim da importância?

E20: Pra mim foi fundamental a questão até do preparo do RN assim sabe, dos cuidados, os primeiros cuidados com a [nome da filha] porque até conversando com outras duas amigas que são enfermeiras também e eu falava pra elas "Meninas, eu não sei o básico, eu não lembro como é que é mesmo dá banho, dei na faculdade e nunca mais encostei numa criança" (risos) que dava um friozinho na barriga assim sabe então quando teve ali a parte dos cuidados com o bebê eu falei gente ainda bem pra relembrar mas tudo sabendo que quando ela nascesse na maternidade também ia ter sabe mas ter esse preparo pra mim foi muito importante e a questão da amamentação, que a amamentação eu tinha bastante medo achei que não fosse conseguir assim em alguns momentos, mandei mensagem pras minhas amigas, falei muito com meu marido eu falei "Ah, se algum momento eu pensar em desistir, achar que eu não sou capaz me lembra que é possível, que vai ser difícil mas que a gente vai passar juntos esse desafio né" e foi bem importante ter essa rede de apoio sabe então não tive uma dificuldade assim eu não cheguei a ter fissura, a [nome da filha] sempre teve uma boa pega mas teve um pouco de sensibilidade mesmo da mama, então no primeiro mês tem, apesar de não ter lesão nenhuma tinha aquela sensibilidade do primeiro contato ali né e dai eu acabei fazendo um pouco de infravermelho que auxiliou e persistência assim, eu queria muito então não era uma hipótese parar.

Entrevistadora: Mas assim que bom que foi bom pra ti e que foi uma rede de apoio que o teu marido também te ajudou e isso é muito importante pra vocês que vivenciaram um processo de muito de isolamento né na gestação pela pandemia né, se puderes abordar um pouco isso como que foi pra ti, pra vocês, a espera do parto nesse processo.

E20: Então, a gente tava no meio da pandemia mesmo não podendo sair pra nada só que a gente mora do lado da minha cunhada então a gente tinha esse apoio dela que foi bem importante né, então a minha mãe vinha mais raramente mas a minha cunhada tava aqui presente então ela ajudou muito nesse início então eu não senti essa falta realmente do momento da pandemia, eu não me senti sozinha, então tinha o apoio da minha cunhada em seguida veio a minha sogra também pra cá então mesmo que tivesse na pandemia a gente acabou tendo esse apoio, senti mais diferença das pessoas conhecerem a [nome da filha] né

então esse contato dela foi mais difícil, sair na rua com ela foi mais difícil então teve muitas coisas tipo o carrinho que a gente comprou a gente percebe que foi um pouco desnecessário porque não, praticamente não usou.

Entrevistadora: Sim, uhum, e a tua consultas de pré-natal fazias acompanhamento por onde, teve alguma influência?

E20: A gente fez o pré-natal com o particular daí, era com o [nome do profissional] e a consulta com ele ainda tava sendo presencial, ele fez só no auge ali mesmo em março uma consulta online em seguida em fez, continuou no presencial, então era ali a saída eu brincava que era o único momento que eu me arrumava que sair de casa (risos)

Entrevistadora: Era o evento (risos).

E20: Eu acabei trabalhando home office né, então durante o pré-natal acabou sendo home office.

Entrevistadora: Teu marido pode te acompanhar também?

E20: Sim sim, durante o parto ele pode estar presente e foi fundamental também né. O que não teve, o que eu senti assim foi a questão das visitas né mas que teve o lado positivo que dai eu não tive que dar atenção quando eu não tinha como dar atenção sabe. Foi muito tranquilo em nenhum momento eu percebi que tava, eu não tive essa dificuldade apesar de ter lido muita gente falado sobre isso “Ah cuidado com os palpites” foi tranquilo, não cheguei a perceber. Deixa eu só tirar ela da cadeirinha, só um pouquinho.

Entrevistadora: E agora se quiseres falar um pouquinho do dia do teu parto, como que foi sentir os primeiros sinais e falasse que a bolsa estourou então provavelmente passasse por esse processo de início de trabalho de parto, mas se quiseres contar um pouquinho como foi?

E20: Então a gente já tinha ido dormir e eu passei um dia normal assim, não teve nenhum acontecimento eu tava com 38 semanas, 38 é, 38 e dois dias e mas elas até brincaram de comer tâmaras pra ajudar, eu falei “Ah, vou começar a comer porque já tô com 38 vai” acho que eu comi por uma semana assim e não é uma fruta que eu goste muito então era aquilo “Tá, eu vou comer pra ajudar, então vamo lá” e daí a gente acordou, aquele dia a gente acabou dormindo bem cedo e daí era onze e meia e eu fui no banheiro fazer xixi e quando eu voltei na cama e deitei peguei e senti um líquido assim aí eu falei “Ué, eu acabei de fazer xixi será que” e daí eu fui me mexer de novo na cama e quando eu me mexi daí veio muito líquido mesmo e meu marido é enfermeiro também e juntou exatamente no dia que ele tava em casa porque ele ia pra uma sequência de plantões e daí eu olhei pro lado e “Meu amor, a bolsa estourou” dai ele “A bolsa estourou? Que bolsa?” (risos) e daí a gente levantou eu fui me arrumar com bastante calma assim que dai eu ainda não tava com contração, fui tomar um banho bem relaxado, aí banho que eu comecei a sentir um pouco mais de contração mas assim, é uma contração e sei lá a cada dez minutos eu acho tava bem espaça sabe e ao longo

do caminho depois de ter tomado banho, me arrumado que a gente foi pra Carmela e daí ao longo do caminho eu tive um pouco mais de contração assim mas não chegou a ritmar em nenhum momento mas eu achava o máximo toda vez que vinha porque eu queria né, eu queria sentir eu “Ai que bom filha, ta no teu tempo”.

E20: É diferente assim sabe, hoje eu percebo o quanto eu sou já uma profissional diferente também, muda muda bastante depois que a gente tá no outro lado assim né, tu vê o outro.

Entrevistadora: Eu imagino, eu imagino porque a gente às vezes pensa que sabe como é mas não sabe porque nunca viveu, eu por exemplo não posso dizer que eu sei como é né porque eu nunca passei por isso. E mesmo assim, voltando né, sendo falasses que já sabias que seria uma cesariana, como que você tentaram se planejar pra esse momento assim né, lembras de alguma coisa?

E20: Eu cheguei a fazer plano de parto também, então eu fiz uma pasta com as músicas pra colocar no parto e eles deixaram eu colocar, a luz não tava excessiva também e foi algo bem tranquilo assim sabe, apesar de saber da cesárea foi planejado, foi com plano de parto e foi bem aceito, não tive dificuldades com eles.

Entrevistadora: Uhum, e a escolha da maternidade, vocês pensaram?

E20: Uhum, então, é porque o [nome do companheiro] trabalha lá também né, então a gente acabou.

Entrevistadora: Ah, sim legal, ai por ele trabalhar lá já conheciam mais ou menos a rotina.

E20: Exato exato, ajudou também.

Entrevistadora: Sim sim, ai ele pôde entrar, participar desse momento?

E20: Sim, ele tava presente o tempo inteiro, desde a entrada até no centro cirúrgico ele pode estar comigo o tempo inteiro, ficou lá comigo também no dia seguinte.

Entrevistadora: Ah, sim, uhum. E como é que foi a abordagem da equipe, achasses que fosse respeitada e conseguias te impor nesse momento?

E20: O tempo inteiro, super bem respeitada assim, eu cheguei até a fazer um elogio pra eles, foi um atendimento muito bom sabe, todo o cuidado, o carinho, o respeito, a empatia mesmo com o momento sabe, mesmo estando em pandemia fui muito bem acolhida por todos né.

Entrevistadora: Sim, e assim só pra gente finalizar pra eu também não passar muito do nosso tempo, se quiseres comentar um pouco pra fechar qual tu achas que é a importância das mulheres e das famílias se prepararem pro parto?

E20: Olha, é fundamental porque eu senti muito pouco as contrações mas tive momentos que já sentia ela e eu acho que a mulher tem que acreditar assim como na amamentação que tem que ter esse apoio de alguém pra lembrar ela que no momento de maior desespero ela vai conseguir sabe, que é uma onda eu acho que quando falam que é uma onda é exatamente isso é uma onda que ela vem intensamente mas ela passa mas, tu saber isso e tu ter esse preparo antes é fundamental porque eu fico pensando quem simplesmente só chega e acontece eu acho que deve ser muito difícil sabe, não é instintivo, tem gente que diz que é instintivo mas eu acredito que não, precisa de um trabalho assim. Eu não lembro se eu te falei, eu acabei fazendo fisioterapia pélvica também e me ajudou a ter o conhecimento da região, de conseguir poder trabalhar a musculatura, acredito que seria bem importante pro momento do parto assim, acho que se pudesse ter mais esses momentos do SUS seria bem legal pras mulheres sabe, o que a gente orienta ainda é pouco e com certeza a partir de agora com o que eu tive posso orientar diferente né, acho fundamental ter esse preparo.

E20: É, e a linha que a gente segue depois, uma coisa que me chamou muito atenção é que eu achava que a [nome da filha] ia pro quarto já logo em seguida com uns três meses eu ja ia conseguir colocar ela no quartinho dela e no grupo de gestantes tem a psicóloga que agora eu não lembro o nome dela. Ela comentou que seria muito importante ficar até os seis meses e pela questão dos cuidados né pra gente observar e a questão do afeto mesmo com a criança né, e daí ali eu falei não então vamos até os seis meses. E quando ela nasceu essa questão de amamentação de acordar de hora em hora ela dorme na cama com a gente, a gente faz cama compartilhada até hoje, então é muito da vivência que tu ta tendo no momento sabe e assim, não tenho medo nenhum, eu sei que vai ter um momento que a gente vai ter que tirar, ter esse processo de sair, mas não vejo que ela é uma criança mimada ou é uma criança que tem alguma dificuldade por estar dormindo ali com a gente, pelo contrário ela é uma criança muito mais seguro e sabe, e que tem a confiança de que tá aqui com a gente com afeto, então é muito diferente assim eu não consigo nem te dar algo específico do quanto esse afeto, acreditar que (perda de conexão), é incrivel.

E20: Ah, eu lembrei agora, foi legal ter mantido o grupo ta, vire e mexe, eu não sou tão participativa mas eu vejo que é muito legal porque tem muitas dúvidas daí as mães trocam muita experiência ali e nesse momento de ser mãe é muito legal essa troca, então tem muita troca de conhecimento e é bem legal, pequenas coisas tipo “ah, meu filho não tá dormindo a noite” aí todo mundo fala “ ah, mas isso é normal, aqui em casa acontece também”, só o fato de dizer que isso é normal nas dificuldades eu acho que nos estimula a seguir adiante sabe, porque tem dias difíceis, tem dias bem difíceis que pensa assim “ Meu Deus, como que eu vou aguentar mais duas noites sem dormir” mas as noites melhoram (risos), elas melhoram.

finalização

ENTREVISTA 21

Data da entrevista: 24/11/2021
DATA DO PARTO: 06/08/2020

Entrevistadora: E desde a gestação como é que foi pra ti esse processo de preparação pro parto, se conseguires lembrar lá o que tu fazia na gestação.

E21: Ah, o que eu fiz na verdade foi um programa que eu ia uma vez por semana, o nome é PPM, Physical não sei o que lá mommy uma coisa assim, que é já no preparo pro parto, um serviço que tem aqui em Joinville que é o nome do lugar é Espaço mãe bebê então trabalha com a fisioterapeuta né pra fazer fortalecimento da musculatura tudo essas coisa né, só que não trabalhava tanto, trabalhava um pouco o assoalho pélvico mas era mais em geral assim né, alongamento e tudo mais, fiz também a fisio pélvica mas eu fiz poucas sessões porque eu ficava com receio de ir né por conta da pandemia, dai também não botei muita fé que ia fazer algum resultado daí achei que seria mais exposição do que tá ajudando assim, ficar saindo de casa naquele momento.

Entrevistadora: Uhum, e era o teu primeiro filho né, o que tu projetavas desse momento, é o teu primeiro filho?

E21: Primeiro filho, uhum.

Entrevistadora: E como é que tu pensavas no teu parto quando tu ouvia essa palavra, como tu projetavas isso?

E21: É, eu queria que fosse um parto normal né, com mínimo de intervenção possível, se eu quisesse que tivesse analgesia e tudo mais mas eu tinha diabetes gestacional né então muita gente já ficava me alarmando pra isso mas mesmo assim eu tava bem decidida. O próprio sistema ele trabalha muito de forma que a gente caia numa cesárea porque eu fazia acompanhamento no alto risco e inclusive numa das últimas consultas a médica falou “Ah, a partir de agora se não controlar tua glicemia daí a gente vai marcar pra cesárea” dai ela falou assim já e eu falei “Não, mas eu não quero cesárea, eu vou induzir” dai ela “Ah tá, é é pode ser” tipo assim já, ai eu “É é, eu vou querer induzir”, “Ah então tá tá bom, a gente fala cesárea porque a maioria quer cesárea” em vez dela fazer o contrário né, não, se eu não fosse com a cabeça preparada né, isso deveria ser um movimento contrário delas falar “Ah, você poderia ta induzindo se não der certo você vai pra cesárea” mas não fosse partir de mim ela já queria agendar.

Entrevistadora: Com certeza. eu acho que isso também é bem como tu falasse se eu não tivesse preparada, eu acho que isso também veio muito da tua formação né como enfermeira mas por isso que eu também acho importante o preparo pro parto né, justamente por isso por esse sistema mesmo que coloca as mulheres em situações vulneráveis e ainda estimula essas

cirurgias desnecessárias. E em relação ali ao grupo de gestantes, como que foi pra ti essa experiência, influenciou de alguma forma no teu preparo?

E21: Eu consegui ligar agora porque ele tá brincando (sobre a camêra). O grupo foi bem legal, eu tinha participado já como bolsista quando tava na faculdade então eu já conhecia, sabia como é que funcionava, foi muito bom pra poder trocar ideia com outras mães, com outras famílias, o grupo de Whatsapp que eles criaram mas assim de conteúdo eu confesso que não foi muita coisa que eu aprendi porque eu já sabia, também eu sou da área da neonatologia, foi mais pra trocar ideia mesmo da experiência e a parte do parto que era o que mais me interessava já que eu sou da neo e não obstétrica né, eu não tava ainda ano grupo foi um dos primeiros né, primeiro encontro, então eu não tava ainda nessa parte então, dai eu entrei a partir da amamentação, cuidados com o bebê e puerpério que é a arte que mais ou menos eu já sei mas eu gostei bastante que fez diferença pra mim foi a parte da [nome da profissional] ali, parte psíquica a gente sempre acaba aprendendo porque é outra área da psicologia.

Entrevistadora: E aí como que foi assim o dia do teu parto, se quiseres falar um pouquinho, os primeiros sinais?

E21: É, na verdade eu não entrei dai em trabalho de parto porque eu tava com 38 e 3 e minha glicemia não tava mais controlando né, eu ja tava com insulina três vezes ao dia, metformina três vezes por dia se eu não me engano e tava dando ainda alto né, o percentil do bebê que eles ficavam controlando abdominal tava bom só que a glicemia não tava boa né, que daí o limite de corte eles são mais radicais na gestação, enquanto a gente não grávida até tolerava uma glicemia dessas mas grávida eles são mais chatos, dai sugeriram interrompeu dai eu internei numa terça pra induzir com 38 e 3 né, isso era uma quinta-feira ai eu fiquei dois dias, por 48 horas induzindo com misoprostol seis comprimidos, mas não entrei em trabalho de parto, dai no terceiro dia eles perguntaram se eu queria continuar induzindo, eu fiquei com plantão né cada vez passava um médico, daí tem aquele que são mais do parto, outros não tanto, dai tinha aqueles que passavam e meio que me assustavam “Ai, vais ficar tentando até quando?” ai eu “Não, mas to de boa aqui tentando, me deixa aqui de boa” mas alguns até falaram “É, tem que cuidar que esse neném vai entrar em sofrimento, não sei o que” mas isso eu tava ok né, eu ia falando com minhas colegas obstetras enquanto eu tava lá internada e tudo mas no terceiro dia a minha intuição que pegou né porque eu pensei “Ai não, não vou tentar o terceiro dia não né” tentei ja fiz minha parte, não entrei em trabalho de parto mas foi pelo cansaço e pela ansiedade que eu meio que venci assim, eu não consegui, porque é ruim a gente ficar dormindo naquelas camas do pré-parto e já tava dois dias, duas noites dormindo lá e também se eu já tivesse engrenado um pouco eu taria mais animada, se ele falasse “A, ja ta com dilatação, já começou algumas contrações” mas poxa eu fiquei lá dois dias inteiros e nada, não dilatou, não teve contração, dai eu ficava pensando só nisso “Meu, quando que vai começar né, entrar em trabalho de parto” dai fora que quando entrar a gente tem mais um chão ai pela frente, sabe lá Deus quanto tempo que vai levar e eu ja tava me sentindo bem exausta assim e tava preocupada também porque na última noite eu senti ele mexer pouco e ele sempre mexia bastante toda noite dai sei lá, enfermeira neo né, dai a gente ja fica

pensando em tudo quanto é merda que acontece ai eu falei “Ai não, não vou correr com a sorte” dai eu pensei “Vamos interromper mesmo porque ja deu”.

Entrevistadora: Dai fosse pra cesárea?

E21: Isso.

Entrevistadora: E aí a cesárea foi tranquila, como é que foi?

E21: É, foi assim tranquilo o procedimento mas eu tava muito nervosa, eu não queria né, eu só fui porque eu tava muito cansada e sem saber assim quanto tempo ia ficar em trabalho de parto quando entrasse né, mas eu tava em pânico, nunca tinha feito nenhuma cirurgia, morro de pavor e eu até desmaiei quando eu fui pro centro cirúrgico, quando começaram a abrir os campos. Isso que é enfermeira né, já tinha visto umas quinhentas cesáreas, já sabia como é que funcionava mas eu fiquei pensando “Ai meu Deus, daqui a pouco é minha pança que eles vão abrir” dai eu fiquei apavorada e desmaiei e o tempo todo eu tava em pânico, pedia pro anestesista me sedar, porque eu tava morrendo de medo e ele não queria por causa do bebê daí eu falava “Ai me seda, pelo amor de Deus, porque eu to com muito medo, muito medo” ai eu sei que eu fiquei a cesárea toda chorando de medo né, daí entrou minha doula e foi bom que ele nasceu e já foi pele a pele comigo né, dai ele mamou ali enquanto eles tavam me suturando ainda, ficou comigo daí eu fiquei feliz por conta disso e também tocou minha playlist que eu já tinha feito uma playlist pro parto, pro trabalho de parto e eu pedi pra eles colocar desde quando começou a montar os campos já tava tocando a minha playlist, na hora de ele nascer tudo e ter o pele a pele tava tocando as minhas músicas então fiquei bem feliz com isso.

Entrevistadora: Ai que legal que fizesse uma playlist então, e não comentasse né, mas onde teu bebê nasceu?

E21: Foi num hospital particular aqui do Joinville, no dona Helena.

Entrevistadora: Ah, você é de Joinvile né. E chegasse a comentar né que muito disso, muito das decisões que foram tomadas levaram em conta o teus conhecimentos né, se puderes falar um pouquinho qual tu achas que é a importância do preparo pro parto né e da mulher tá preparada mesmo e participar dessas decisões, principalmente quem não tem esse conhecimento prévio.

E21: É importante a gente ter conhecimento, nem que seja ou não profissional, estar informada ali bastante mas infelizmente isso não garante também nada, infelizmente tu vai tá a mercê dos profissionais que vão te atender mas o bom é pra tu ter uma ideia, como eu falei, eles já estavam indicando a cesárea né, então com tantas semanas a gente vai interromper, se eu fosse uma mãe qualquer eu ia falar “Não, então tá, o médico falou que tem que fazer cesárea né, por causa da minha diabete” e eu não ia saber “Ah, mas dá pra induzir né” e outra coisa que eu ficava bem incomodada era com essa questão de suplemento porque eu

trabalhava na maternidade pública aqui de Joinvile e a gente tinha um protocolo certinho né pra complemento, então não dava a torto e à direita, e a gente sabe que nesses hospitais particular dependendo do plantonista do pediatra acabam "intochando" leite nas crianças, então esse foi um cuidado que eu tive no meu plano de parto, de colocar que se meu bebê precisasse de complemento que eles primeiro falassem comigo, isso era pra ser uma decisão em conjunto equipe e comigo, que eu não autorizava que dessem leite pra ele que não fosse o meu, que dai eu ia ver claro se não tivesse com sinal de hipoglicemia, muito ruim, beleza mas eu não queria que eles dessem assim, eu ia tentar tirar o meu primeiro se precisasse né, mas no fim nem precisou a glicemia dele deu ótima mas essa foi uma preocupação e o que eu tinha muito medo que eu coloquei no plano de parto era a questão da episio mas o ruim é isso que não depende da gente, depende muito do profissional, dai eu falava muito pro meu marido "Não deixa me cortarem, não deixa me cortarem" dai ele ficava ansioso também com isso ele falava "Meu mas o que que eu vou fazer, vou saber se tem que cortar, não tem que cortar" é porque eu no caso tive um rolo lá com uma médica sabe por isso que eu fiquei com o plantão, eu tinha uma médica dai deu altos rolo e troquei de uma pra outra dai essa outra também abandonou barco porque a outra ficou braba porque ela pegou né tipo meu caso, dai ficou briga de comadre ali "Ah, roubou minha paciente" sabe esses negócios, dai acabou que eu cai no plantão mas a minha ideia não era cair no plantão, ainda bem que deu tudo certo assim, fui falando com as colegas enfermeiras elas foram falando, até foi uma enfermeira obstetra amiga minha que me incentivou ir pra cesárea no caso, ela é bem parto normal mas quando eu tava lá na segunda noite de indução eu mandei uns audios pra ela chorando, que ja tava com bastante medo né, eu falei "Ai eu to com medo, eu to cansada, eu não sinto ele mexer direito, não tô com nada de contração, nada de dilatação" dai ela falou "Vai tranquila pra cesárea, tu fez tua parte" ela falou "Tu quer continuar" dai eu falei "Ai não sei, meu coração ta afito" eu falei pra ela dai ela "Então vai tranquila pra cesárea, não fica se culpando" mas mesmo assim eu fui mas até hoje eu penso, meu devia ter tentado mais um pouquinho né, que seria melhor. Porque na verdade a cesárea é bucha, até hoje eu sinto um pouco os pontos né.

E21: A médica que fez a minha cesárea era uma que me atendeu um pouco no pré-natal porque ela era da minha unidade de referência né. embora eu não fizesse o pré-natal com ela né, alguma consulta ou outra eu ia na atenção primária, até pra pegar o glicosímetro, as fitas né essas coisas assim eu fui, e ela era minha médica antes também de engravidar já, tanto é que depois eu troquei de plano e ela não atendia no meu plano porque ela era da prefeitura e do particular né, do convênio. E daí eu fiquei feliz quando eu fui pra cesárea que dai eu não sabia quem que era o plantão né do dia logo cedo da cesárea, dai eu cheguei lá e vi que era ela dai eu falei "Ai, não acredito que é você" a gente tinha sido colega de trabalho também né, ela trabalhou comigo, eu falei "Ai que bom, agora eu to mais tranquila que é você" eu sei que né, to em boas mãos assim e ela depois que fez a cesárea que veio falar comigo na recuperação ela falou "Ah, ele ta bem alto, o útero tava bem grosso ainda" ela falou que normalmente essas induções são boas pra preparar também né, por mais que não vire o parto normal mas já vai preparando o útero mas ela falou que tava bem grosso ainda que não tinha afinado né então ela falou "Olha, ia demorar um pouquinho pra ir esse trabalho de parto" e eu fiquei com medo de ficar dias e dias, só que fico me cobrando que às vezes eu penso assim de

repente eu não deveria ter internado naquela data pra induzir, porque falaram o pessoal do alto risco né, que eu deveria interromper com 38 e 3, talvez tivesse visto uma outra opinião, mas não dá pra levar até os 39 né, embora eu falei com algumas colegas, falei também com a [nome da profissional] até, com a professora e ela falou que no HU também o protocolo é assim, não passa muito de 38, se ta com diabetes descompensada, eles vão tentando induzir mesmo e dai também não adianta ficar pensando né, priorizar ou não o parto, e dai pelo menos ele não foi pra UTI né porque eu tinha outra colega que tava grávida que também era diabética, o bebê dela fez hipo bem severa né, precisou ir pra receber soro na UTI então, bem ou mal interromper naquela hora foi bom pra ele assim né, porque ele não teve distúrbio glicêmico nada por conta da minha alteração.

Entrevistadora: Uhum, e em relação a vivenciar isso num contexto pandêmico, o que tu achasse, como que foi pra ti?

E21: É, a gestação nem tanto ruim mas o puerpério eu achei horrível, bom a gestação também achei frustrante porque eu vejo assim que a gente ficou com muito medo né, eu já tinha medo na gestação por tudo por conta da nossa profissão né e daí cada semana ai 25 semanas, 26 semanas, eu ficava pensando “Já cuidei de um neném com essa idade né” dai eu ficava imaginando ele todo desmilinguindo pequeninho lá e vai se nascer agora né, também tinha bastante medo, dessa história do Covid que não tinha vacina né então a gente não saia de casa fiquei dois anos trancada dentro de casa e é bem ruim, dai ninguém me viu grávida da família, os amigos, ficava trancada então isso foi bem frustrante mas o pior ainda eu acho que foi o puerpério porque tu fica sozinha com o neném por mais que o pessoal fale “Ai que é bom, não tem ninguém dando pitaco mas é não que você precise de alguém na tua casa mas o sair de casa é importante né, com o neném dar uma volta, eu não me sentia segura pra sair dar uma caminhada, eu achava muito arriscado, dai eu morava em um apartamento na época também pequeno dai isso tudo deixava mais estressada ainda né, o puerpério pra mim foi o pior na verdade mas claro que dai teve outras coisas que eu queria ter feito na gestação tipo a hidro né, que o pessoal fazia hidroterapia, essa aula ainda que eu fiz do PPM que eu te falei eu fiz porque era né seco, dava pra usar máscara dai na hidro o pessoal não usava máscara, tinha um pouco de receio mas eu sempre tive expectativa de fazer tudo isso quando ficasse grávida né mas dai não, dai a drenagem que eu ia lá fazer toda semana eu pedi pra elas irem na minha casa fazer porque eu fiquei com medo de sair e mesmo assim PPM eu fiz muito pouco porque eu ficava acompanhando na tv como é que tava o mapa de santa catarina dai eu ficava com medo, uma hora eu cancelava outra hora voltava, ficava assim, toda hora muito ansiosa.

Entrevistadora: Sim, esse PPM era o que um grupo?

E21: É um programa feito por esa fisioterapeuta ela tem uma capacitação lá internacional não sei o que que dai faz esse preparo pro parto sabe e daí era isso que eu fazia com ela.

Entrevistadora: E dai ela trazia esses assuntos ou era mais parte prática mesmo?

E21: É prático mesmo, é exercício né, dai fortalecia braço, perna, tudo. Exercício focado pra gestação né. Dai eu fazia um pouco em casa também, eu comprei uma bola de pilates lá e tentava fazer.

Entrevistadora: Comentasse ali que no teu parto entrou tua doula né, nesse momento já podia entrar a doula então? E teu parceiro ele também entrou?

E21: Isso, a doula foi assim um perrengue que a gente passou pra colocar ela dentro porque eu fechei a doula muito cedo, sei lá eu tava com doze semanas, minha amiga já me indicou essa doula e eu fechei, e daí não deixaram entrar lá pelas tantas, até uma colega que ganhou bebê antes ela fez a parte de internação dela video chamada né, dai eu falei "Meu, vamos pagar uma grana pra doula e vai ser video chamada" mas dai como eu fui pra induzir eu falei pra ela que não precisava ir na indução né, eu falei "Quando eu entrar em trabalho de parto eu te chamo né" só que ali eles tinham deixado mas o que que foi, a gente teve que fazer um pedido formal pra direção, entregar um papel, cheio de documento não sei o que, e a gente só conseguiu porque tinha uma mãe no grupo que a gente tava ali que era juíza e ela mandou pra gente as coisas que tinha que colocar nessa carta pra meio que pressionar eles e a gente fez tudo mais ou menos a mesma carta pro hospital, dai eles deixaram, só que dai quando eu pra cesárea ele acharam que eu não ia querer ela, porque eu tinha fechado ela pra parto normal ou cesárea o que fosse né, dai eles até vieram me perguntar a enfermeira lá falou "Tua doula tá lá embaixo, ela quer entrar, é isso mesmo? Você quer a doula, mas é cesárea" eu falei "Não, mas eu quero, ela sabe que ela tem que tá aqui, ela vai subir" daí eles deixaram "Ai, então ta bom, então a gente vai deixar" e foi bom porque senão ninguém ia por ele pra mamar eu sei como é que é, o pessoal não coloca, mas ela sabia que eu queria e ele nasceu ela já me deu ele e colocou no peito, foi muito bom, e meu marido entrou também sim, ele tava comigo todos os dias da indução só que dai eu pedia pra ele ir embora a noite, eu falei a não tem sentido tu ficar aqui também se eu nem to em trabalho de parto, eu falei fica tranquilo que se eu entrar em trabalho de parto eu te chamo né dai ele ficava, ficou o primeiro dia inteiro, segundo dia inteiro mas de noite ia embora, quando chegou no terceiro dia ele chegou logo cedo dai eu ja falei que eu ia, tinha optado de ir pra cesárea. Foi tudo muito rápido, perguntaram "Cê quer mesmo cesárea" eu falei "É vou querer" porque eu ja tinha ficado em jejum até porque tava imaginando que faria isso, ela falou "Ah, se você quiser tem uma vaga agora pra primeira ou então tu vai ser das onze da manhã, meio dia" e eu falei "Não então eu quero já" foi tudo meio no susto assim.

E21: É, eu penso que a pandemia afetou muito isso né porque até na escolha de profissional né, porque dai tu fica meio assim de ficar peregrinando por ai né, já mal queria sair pra ver um médico quem dirá ficar olhando vários né, pegando uma segunda opinião e tudo mais. E o próprio preparo perineal tudo né, porque como eu te falei eu fiz um pouco mas dai lá pelas tantas já interrompia porque eu ficava com medo, eu acho que eu não fui em quatro sessão de, bem pouco assim, de fisio, porque me dava medo de sair de casa assim.

E21: Mas em compensação no puerpério a gente se ferra né quem é o primeiro filho porque as pessoas falavam "Ai tem que se preparar pro puerpério, se preparar pro puerpério" e eu

sempre achava que o medo do povo no puerpério era cuidar do neném né, que é o principal de não saber o que faz né, chora não chora, não sei, dai eu pensei bom eu sou enfermeira neo, tenho experiência eu sei que tá sossegado, puerpério vai ser leve, dai quando a gente vê aquela queda hormonal tudo, nossa, dai ve que cuidar do neném é o de menos. O ruim é todo o resto, que eu não me preparei psicologicamente até da dinâmica assim, da comida de tudo, isso não fiz assim, então vivi num emaranhado, na hora que o bebê nasceu eu pensei "Meu Deus e agora" do resto tava tranquilo, só não tinha me organizado na minha dinâmica da casa, como é que eu ia fazer.

finalização

ENTREVISTA 22

Data da entrevista: 30/11/2021
DATA DO PARTO: 30/08/2020

E22: A [nome da filha] nasceu no dia 30 de agosto de 2020, no auge da pandemia.

Entrevistadora: E como é que foi pra ti desde a gestação, é a tua primeira filha, como é que tu pensavas no parto?

E22: É a minha primeira filha e pretendo que seja a única (risos). Na verdade foi um desafio porque gestar é realmente um turbilhão de emoções, hormônios e tudo mais e a gente descobriu que estávamos grávidos em janeiro e em março veio a loucura da lockdown ali, todas as incertezas a respeito dessa pandemia, quando ela nasceu eu lembro que quando começou lockdown todo mundo falou que ia ser pouco tempo, que até o período em que ela nascesse teria tudo normalizado e aí as coisas foram ficando cada vez mais complexas né, a gente se isolou porque não se tinha conhecimento especialmente nas gestantes, porque né, quais eram os riscos pra gravidez, pro bebê, enfim. E aí nós ficamos isolados a família tem um sítio em Rancho Queimado e a gente tava em home office então passou praticamente toda a minha gravidez eu passei lá, a gente voltou em julho já e ficou até a [nome da filha] nascer, então eu não tinha nenhuma, não romantizei muito nada a respeito do parto mas a gente se informou muito assim, a gente estudou bastante e queria que fosse parto normal né, por todos os benefícios pra ela e pra mim teoricamente né. No fim a gente teve um parto normal mas cheio de complicações, foi bem difícil, eu tive uma hemorragia bem feia também, fiquei o primeiro mês praticamente de cama assim, foi bem difícil mas foi um parto normal, ela ta aí ta tudo certo, nos preparamos e dentro do possível, com todas as orientações da minha obstetra e com os grupos de apoio, o grupo do HU, foi uma pena ter sido online porque eu busquei o grupo justamente porque tinhas ótimas referências né, inclusive por que acaba sendo, as pessoas se conhecem acaba formando uma rede de apoio legal, no nosso caso a gente tem o grupo ali mas como ninguém se conhece pessoalmente acaba não sendo tão bacana né assim, mas foi a título de informação com certeza né, a gente teve bastante esclarecimento.

Entrevistadora: Uhum, e as tuas consulta de pré-natal, foram, influenciaram alguma coisa estar na pandemia ou pra ti?

E22: Não, no começo influenciou porque eu fiz tudo pelo meu plano, eu tenho plano de saúde então não foi pelo SUS, então eu tinha a minha obstetra que foi a que a gente chamou depois pra fazer o parto e no começo todos eles né, até os médicos estavam um pouco receosos, então algumas consultas foram online também e aí é muito diferente, não tem como tu comparar, tu não faz um exame físico né tu te pesa e passa pra ela, passa as informações mas ela não ta te avaliando então assim, foi um período bem ruím assim, um período que eu fiquei bem insegura sabe, bem insegura mesmo. Mas logo depois ela resolveu assumir o risco que ela viu que não tinha condições de cuidar de uma gestante à distância, muito complicado,

logo depois a gente voltou então a gente ficava vindo de Rancho Queimado só pras consultas mesmo. E é diferente, é mais impessoal, por mais que as pessoas sejam legais, que tentem, é muito diferente, sabe.

Entrevistadora: Qual tu achas que foi a influência do grupo falando do teu preparo pro parto ne, falasse que vocês liam muito, estudavam muito, como é que era assim, de onde vocês buscavam essas informações?

E22: Eu falei referente a eu e meu marido no caso, né?

Entrevistadora: Isso.

E22: Ah, a gente gosta de ler, gosta de se informar então assim a partir tanto dos grupos que foi formado pela minha obstetra, ela forma um grupo sempre das vidinhas que ela vai gerar naquele ano né, então ali a gente trocava ideias também, ela mesma passava algumas bibliografias assim a título de sugestão, é muito louco porque tu começa a seguir uma ou outra no instagram dai ja vai aparecendo outras coisas quando tu vê tu ja ta seguindo só perfil sobre isso, médicos e pessoas que falam sobre maternidade, e aí no grupo também acabo, não lembro quem sugeriu algumas leituras, e no fim tu vai ver são quase sempre algumas dessas leituras coincidem sabe, é bem engraçado, tem alguns livros que são meio que clássicos da maternidade que é aquele “A maternidade um encontro com a própria sombra” e o “Besame mucho" acho que são os dois principais assim e esses são os clássicos, que eu não gostei nenhum pouco de um deles ainda. Inclusive eu acho que no grupo elas até disponibilizaram algumas leituras também, é eu acho que ali naqueles momentos de tirar dúvida com a enfermeira, eu acho que era uma enfermeira chefe eu não vou lembrar do nome porque já faz bastante tempo, porque foi no começo do ano passado ali né, metade do ano passado e assim dos cuidados com o bebê eu acho que foi legal sabe, embora sabe fosse muito melhor se fosse pessoalmente a gente vendo né, ensinando a banho, isso aquilo, assim, são coisas que tu busca na internet, tu vê video mas saber que tem uma pessoa ali com propriedade, profissional e tal te falando, uma pessoa que trabalha com isso né diretamente, te da um pouco mais de segurança assim né e assim, não foi tão agregador quanto eu imaginava mas por isso porque a gente ja tava indo muito atrás de informação, a gente fez outros cursos nesse mesmo estilo assim né, eu acho que a diferença maior é essa assim, o HU é uma instituição super respeitada, é saber que são profissionais dali que trabalham com isso há anos, então tipo as informações te dão uma segurança maior sabe, que eu me recordo mais que umas das aulas que, dos encontros, que foi mais interessante foi esse assim dos cuidados com o bebê talvez até pelo fato de ser uma das principais inseguranças que a gente tinha assim né.

Entrevistadora: E chegasse a fazer um plano de parto?

E22: Sim, a gente fez um plano de parto, de parto e de pós-parto, eu fiz um curso, um outro curso falando sobre um plano de pós-parto que achei super legal também, que todo mundo só fala do parto né mas o perrengue mesmo vem depois e aí a gente fez, eu acho que no grupo

eles disponibilizaram também um modelinho né e na verdade eu peguei vários modelos e adaptei pro nosso assim sabe e aí junto com a nossa obstetra também, a gente também tinha uma doula então foi uma coisa meio que multidisciplinar assim sabe, todo mundo ajudou um pouco e a gente fez, foi bem legal assim, dentro do possível do nosso parto que foi um caos assim, bem distante do que a gente imaginava, foi bem humanizado sabe, dentro daquilo que a gente esperava e tinha colocado no plano de parto.

Entrevistadora: Uhum, depois se tu quiseres contar também de como foi teu parto, porque tu achas que foi caótico, que tu achas que influenciou assim, foi no teu preparo, o que que aconteceu?

E22: Não teve nenhuma relação com o nosso preparo porque a gente tava, inclusive eu acho que o fato de estarmos tão preparados fez com que a gente passasse pela situação que a gente passou da melhor forma possível né, mas o que aconteceu foi que eu comecei depois das 35 semanas a ter pressão alta e a gente ficou monitorando, não era nada assim, a princípio não era nada preocupante mas tinha que ficar monitorando porque a minha pressão sempre foi muito baixa e do nada ela subiu bastante e aí nisso a minha obstetra ficou avaliando e começou a subir subir subir ela disse "Ó vamo esperar até 39 semanas e a gente vai ter que, se tu quer muito parto normal, a gente vai ter que induzir" porque a minha obstetra e super humanizada de esperar até 41 semanas tudo certinho mas por conta dessa questão da pressão ela falou "Vamo induzir porque não vai dar pra esperar" e aí com 38 semanas 5 dias, passei bem mal assim e a minha obstetra falou "Ó então...", isso num sábado, "...amanhã, domingo 6 da manhã a gente se encontra na maternidade pra gente começar a indução" e nisso a minha doula tava lá em casa e a minha doula é naturóloga sabe e aí ela faz acupuntura essas coisas e aí a gente começou a fazer também umas acupunturas, umas coisas e eis que eu entrei em trabalho de parto naquela madrugada, super espontâneo assim 2h30 da manhã, me acordei com uma sensação de que queria ir no banheiro né, e aí eu lembrei que eu tinha lido muito sobre isso, que às vezes uma das sensações é essa e de fato eu ia no banheiro e nada, voltava pra cama e tinha a mesma vontade e voltava e fiquei nessa até que começou a se intensificar e acordei o meu marido e falei "Ó vamo, liga pra doutora porque eu vou entrar no banho e eu sei que se não diminuir né é porque eu to em trabalho de parto ativo, mais ou menos por ai pelo que a gente tinha tido de orientações e tal e realmente, tava bem, o espaçamento tava cada vez menor, tava mais intenso e não diminuía nem no banho assim as contrações nem nada e aí ela falou "Ah, vou te avaliar e a gente se encontra na maternidade" e eu cheguei na maternidade ela disse que jurava que ia me avaliar só pra me ver e me mandar embora só que eu já estava com seis de dilatação, aí a gente já ficou ali internado e tal e o parto tava evoluindo bem, aí eu fui pra água pra aliviar, tava na banheira e aí eu percebi que tinha uma coisa assim meio na partolândia eu já tava mas consegui perceber que tinha alguma coisa, que elas ficavam sempre monitorando o batimento cardíaco da minha filha e a minha obstetra perguntou se eu fazia questão que ela nascesse na água daí eu falei "Não, não faço" dai ela meio que orientou, pediu pra eu sair sabe, e aí eu fui pra aquela cadeirinha de cócoras,banquinho né e aí ela falou assim que o batimento dela tava caindo, que a gente ia ter que, eu ia ter que fazer bastante força na próxima contração porque ela precisava nascer e enfim, ela disse que ia tentar usar o vácuo mas não deu certo, não sei porque, também não me

lembro de detalhes eu sei que não deu, não conseguiram usar o vácuo e aí ela falou "Na próxima contração tu faz toda tua força porque ela precisa nascer" e aí eu fiz, a [nome da filha] nasceu com duas circular de cordão e bem apagada assim bem apagadinha eu lembro só da gente perguntar "Ela ta bem, ela ta bem" e eles só "Não, mas vai ficar" e aí elas super queridas eu falo da questão de ser humanizado porque ela ainda no meio daquele turbilhão ela falou "Olha, pai você não vai poder cortar o cordão, conforme ta no seu plano de parto, porque a gente precisa fazer aqui os procedimentos" mas assim, tudo com muito respeito né, e aí logo graças a Deus ela logo respondeu, só que aí nessa questão de fazer força muito rápido, de não poder respeitar o corpo né, porque até então tava respeitando, respirando e tal, meu útero não contraia mais e eu fiquei com uma hemorragia muito horrível assim e daí apaguei, tive que ir pro centro cirúrgico sei lá o que, fiquei duas semanas repondo ferro na veia, a gente não fez transfusão de sangue porque teve algum problema lá na maternidade mas a indicação era transfusão então assim, foi um pouco difícil né, primeiro até acho que mais pro meu marido coitado, primeiro ele achou que a filha tinha nascido morta daqui a pouco achou que a mulher ia morrer né.

E22: Foi assim um pouco fora do que a gente imaginava.

Entrevistadora: Nesse momento ele pode te acompanhar então pelo que tais falando?

E22: Sim, ele pode. A gente tava com bastante dúvida porque até então nem o parceiro poderia entrar em alguns lugares eu acho que no HU não podia né, tinham algumas mães no grupo que estavam bem desesperadas, eu se tivesse sozinha tinha enlouquecido.

Entrevistadora: A tua doula te acompanhou?

E22: É a minha doula entrou mas é porque no fim como é particular tu acaba conseguindo né, assim, ela é uma doula que sempre presta serviços pra eles então assim acaba que já tem um protocolo que a maternidade tava seguindo que tinha algumas pessoas, alguns profissionais que estavam cadastrados, que tinham feito um curso de segurança sabe teve tudo isso, eu lembro que ela teve que fazer um curso especial mas ela pode entrar então eu tava com a minha equipe, a única que tu não pode levar é a pediatra né que daí tem que ser da maternidade mas eu tava com a minha obstetra, com a enfermeira obstetra dela, com a minha doula e o meu marido graças a Deus, então pelo menos esse ponto né. Mas assim, a gente não pode receber uma visita, a gente nem comentou que tinha sido difícil pra ninguém, nem pros meus pais nem pros dele porque eles iam ficar muito mal, sem poder fazer nada sabe, todo mundo se sentindo impotente, não podia receber visita, ele não podia sair se não ele não podia voltar, tinha muita coisa acontecendo ao mesmo tempo né ali né na questão da pandemia que dificultou um pouquinho assim.

Entrevistadora: É, imagino… Assim pra finalizar se quiseres trazer, qual é para ti a importância do preparo pro parto?

E22: Olha, eu acho que tu tem que tá preparado porque tudo pode acontecer sabe, tu que saber que um parto natural é um parto natural mas que em algum momento podem ter intervenções e tu tem que saber o que pode acontecer em termos de intervenções que são realmente necessárias que não são questões de violência obstétrica sabe, se tu tá preparado, se tu sabe realmente aqui que tu podes esperar do profissional, porque eu sei que eu sou uma privilegiada eu levei a minha obstetra mas que a maioria das pessoas vai pra um plantão de um hospital público então assim essa questão da violência obstétrica é bem grave e ainda bem recorrente agente sabe, e acho que se preparar pro parto é se preparar emocionalmente muito porque tem gente que romantiza demais né, também saber que se for necessária uma cesariana a cesariana ela ta ai pra salvar vidas, então tu também não é menos mãe porque não conseguiu fazer um parto normal, acho que é tudo assim na verdade, a primeira etapa pro teu maternar é tu tá preparada pro teu parto.

E22: Eu fiz questão de participar porque assim o grupo realmente foi uma coisa especial, uma pena ter sido online, mas apesar disso foi, ele realmente foi , somou bastante aqui pra gente sabe

finalização

ENTREVISTA 23

Data da entrevista: 02/11/2021
DATA DO PARTO: 18/08/2020

E23: Foi na primeira alta assim da pandemia né, quando o [nome do filho] nasceu foi a primeira vez que deu assim 90% de ocupação nos leitos assim né, então como era tudo muito novo, muito recente, da um susto bem grande assim né, eu me lembro bem direitinho do dia que eu parei de trabalhar, foi no dia 17 de março de 2020 né, que o governador do Estado pediu pra que tudo fechasse porque foi aquele "BUM" assim, chegou no Brasil, tinha chegado em Florianópolis né, e aí eu me lembro que assim a parte grávida da história, eu me lembro que eu ja tava achando o máximo exibir a minha barriga, ir pro trabalho exibir, eu nunca fui muito de sair a gente nunca saiu muito assim né mas eu achava o máximo assim já usar roupa justinha pra mostrar minha barriguinha que já tava aparecendo né, daí no dia 17 disseram assim "Ah, então todo mundo vai trabalhar em casa" nossa aquilo caiu como um peso, aí que eu fui ter a noção do negócio, que eu ia ter que ficar em casa talvez até ele nascer, a gente não sabia né, foi assim sabe. E aí eu me lembro que eu comentava com a minha família e meu pai dizia assim "Ah até o [nome do filho] nascer tudo já passou" imagina o [nome do filho] ta com um ano e quatro meses e ainda não passou né e aí assim, outra coisa que me marcou assim muito né, foi que eu teria que ir pra maternidade e o [nome do filho] ia nascer e não ia ter sabe aquela imagem da família vendo a criança pela primeira vez no espelho da maternidade, no vidro lá da maternidade, e eu tinha mandado fazer lembrancinhas pra ele, mandei fazer umas quantas lembrancinhas e depois eu não pude receber ninguém sabe, ai isso foi bem angustiante assim.

Entrevistadora: E aí como que era pra ti se preparar pro parto, como que tu pensavas o teu parto?

E23: Eu achei que, eu me inscrevi no grupo de gestantes pensando em ter aquela interação né, eu tinha uma amiga que já tinha feito o curso e ela tava super me aconselhando a fazer, e aí eu imaginava que eu ia participar do grupo de gestantes pra ter uma, realmente assim um grupo de apoio, de amigas mesmo assim que a gente pode se conhecer pra depois encontrar os filhos né, e assim ó eu, uma coisa uma dificuldade assim que eu percebi né que por exemplo quando eu cheguei na maternidade a minha bolsa rompeu tá, rompeu em casa numa sexta-feira de noite e aí a gente foi pra maternidade né e aí eu cheguei lá já tive que entrar sozinha sabe, o [nome do companheiro] não pode entrar comigo né, ficou no carro e aí eu fui pra sala de preparo ali fazer aquele exame cardiotoco , fiquei sozinha né, daí eu tive que tomar um antibiótico antes porque eu tinha aquela bactéria sabe e aí de bolsa rompida, fazendo antibiótico na veia, numa sexta pra sábado de madrugada sabe, sozinha, pode de cansada né e aí só depois que eu fiz o cardiotoco, que eu fiz o antibiótico, isso foi sei lá acho que pela uma ou duas horas da manhã que aí eu fui pro quarto e aí o [nome do companheiro] pôde ir comigo, ele ficou todo esse tempo lá fora esperando sabe, por causa dessas restrições que tinha. E aí ele foi comigo dai ta, dai o [nome do filho] nasceu às quatro e trinta e nove da

madrugada, foi cesárea, ele tava sentado né, mas pelo menos o [nome do companheiro] pôde acompanhar, mas depois a gente ficou literalmente isolado dentro do quarto porque por exemplo assim eu inchei muito depois de ter ele sabe, eu teria que ter caminhado pra desinchar e eu não podia fazer nada disso sabe, porque pensa um quarto pequenininho de maternidade né, então tu tinha sei lá uns metrinhos só pra caminhar sabe e ninguém podia entrar, pra ti ter uma ideia a gente chegou e o [nome do companheiro] esqueceu o carro com a luz ligada e ele não podia nem sair e ver se tava tudo bem com o carro sabe, o carro ficou na rua e quando agente foi embora a gente saiu da maternidade com o [nome do filho] no carro e tinha acabado a bateria e aí todas essas coisas assim né, a ninguém pôde ver, trazer uma florzinha uma coisa assim, nada disso, foi tudo muito frio sabe e ao mesmo tempo tudo com muito medo né, desde o dia 17 de março quando tudo parou quando eu fiquei trabalhando em casa e eu me lembro que eu vi uma notícia que tinha uma grávida no Ceará que tinha morrido, me apavorei né e aí pronto, aí eu fiquei em casa trancada em casa, não ia em lugar nenhum só o [nome do companheiro] que ia no mercado, quando ele chegava a gente lavava tudo, passava álcool em tudo, sabe essa função assim né.

Entrevistadora: Mas assim né na tua gestação, como é que tu descobrisse o grupo, como é que tu te preparavas pro parto?

E23: Eu descobri o grupo por essa minha amiga que ja fez e assim eu vou te dizer que eu era uma grávida bem relax assim sabe, eu pensei assim que eu não ia ler muita coisa porque como eu sou ansiosa né, aí eu ficava pensando assim que isso talvez ia piorar minha situação sabe, então eu não fui assim de ler muito eu pensava assim ó "Não eu vou gerenciando a medida que as coisas forem acontecendo", então eu participei das aulas ali do grupo né, tinha umas que eu não conseguia ver às vezes pela internet né, mas uma que eu me lembro assim bastante que eu tirei bastante dúvida até foi justamente aquela do parto em si, do que que ia acontecer primeiro sabe, que eu não sabia assim se podia romper a bolsa, se podia, sabe eu não sabia assim a ordem das coisas, se contraia primeiro, se rompia a bolsa primeiro, não sabia de nada assim né e que cor que eram os líquidos que iam sair né, o que que a gente tinha que cuidar, essas coisas assim eu não sabia absolutamente nada então essa aula de aprto eu me lembro que eu participei do início até o final e tirei bastante dúvidas sabe e isso pra mim foi bom né, que dai eu chamava o meu marido, os dois trabalhando em casa, falava "Ó ó" pra cuidar também né (risos). Mas assim como o [nome do filho] tava sentando e ele ficou sentado a gestação inteira né, eu já sabia que ia ser cesárea mas eu tinha na minha cabeça assim que mesmo sendo cesárea né eu ia esperar ele dar um sinalzinho pra não tirar ele sei lá, com menos tempo ou alguma coisa assim né, ia esperar ele, e assim foi ele veio de 37 semanas (perda de conexão) mas foi porque rompeu a bolsa né, e aí foi o sinal que ele me deu que ele tava pronto.

Entrevistadora: Perfeito, nossa, ele nasceu no tempo dele né.

E23: No tempo dele é, isso era uma coisa que eu sempre pensava, que eu ia deixar ele no tempo dele sabe, que eu não ia querer forçar nada ou sabe, isso eu tinha assim certeza né que

eu ia fazer, era a única coisa que eu tinha bem assim em mente e como eu já sabia que ia ser cesárea né, daí então foi isso.

Entrevistadora: E como é que foi as tuas consultas de pré-natal?

E23: Foram bem tranquilas eu fiz tudo bem certinho né, todos os acompanhamentos né, desde o início sempre ficava ansiosa pelo próximo ultrassom, aquela ansiedade boa que dá né, de saber se ta tudo bem, se ganhou peso né, mas foi uma gestação assim bem tranquila, eu tava engordando assim ganhando peso bem como tem que ser, teve um mês só que eu ganhei três quilos assim, em uma consulta só dai a médica disse assim "Ó, vai com calma, porque se tu ganhar mais três quilos todos os meses né, vai ganhar muito peso" mas aí no fim eu ganhei doze quilos no total e dai quando ela me falou aquilo dos três quilos eu cuidei, se não me engano foi no sexto ou no sétimo mês, ainda tinha bastante gestação pela frente pra ficar ganhando três quilos todo mês né, e aí cuidei, comi coisas com qualidade mas não tanta massa assim né, eu cuidei bastante da minha alimentação durante assim né, principalmente a parte de vitaminas e suplementos sabe, fiz acompanhamento com nutricionista por causa da suplementação.

Entrevistadora: Sim, e achas que por ser assim pandemia, teve alguma redução do número de consultas, algum dano na qualidade das consultas?

E23: Ah eu acho que assim, eu não reduzi o número de consultas ta, pra mim foi, consegui ir em todas tudo tranquilo mas uma coisa que eu senti muito muito muito é não ter a presença do [nome do companheiro] nas consultas sabe, porque dai assim ó ficou uma coisa assim ó, eu sabia de tudo, eu tava aprendendo as coisas e ele não sabe esse afastamento assim, parece que isso era até como assim o filho é só meu, sabe? Eu sabia de tudo e ele não participava de nada né e aí eu fazia consulta depois marcava o exame de ultrassom e ia fazer na outra clínica, dai ele também não podia entrar e querendo ou não assim ó, sendo que assim como eu estava na consulta e no exame tirando as minhas dúvidas, tirando os meus questionamentos, interagindo sabe com o médico, conversando e vendo, aquela coisa assim né eu acho que isso vai crescendo o conhecimento da gente, crescendo o interesse pela gestação, pela fase sabe, aquela coisa que te motiva né e o [nome do companheiro] não pode participar de nada disso, eu acho que ele foi em uma ou duas consultas só antes de março ali e aí então eu percebi assim que parece que criou um certo distanciamento dele assim sabe, em relação ao processo todo assim, dai essa foi uma dificuldade assim que eu percebi sabe, dai o [nome do filho] dai ele me ajudou bastante na maternidade mas depois eu sentia ele assim bem despreparado, um pouco é questão de maturidade também da pessoa né, ele não teve maturidade suficiente e por acho não ter tido toda essa interação durante a gestação, eu acho que isso comprometeu um pouco sabe o nascimento da paternidade dele.

Entrevistadora: Vocês chegaram a fazer um plano de parto?

E23: Não, nem sei o que que é isso pra falar a verdade.

Entrevistadora: Plano de parto é um documento né da mulher, que ela coloca todos os seus desejos, o que ela deseja durante o parto mas é um documento assim legítimo mesmo que tem, ele é válido que as vezes é um pouco desconhecido, no grupo é abordado não sei se tu te recordas mas é um documento bem importante assim, não tinhas conhecimento?

E23: Até assim agora que tu tocou nessa questão vou te falar uma coisa que eu pra assim foi bem, foi um pouco sofrido ta, que eu não imaginava. Assim ó, eu sempre soube que essa minha médica era um pouco mais fria assim sabe mas eu não sou daqui, tenho pouca gente que eu conheço daqui, já tinha ido pra um outro obstetra não tinha gostado, ja tinha ido pra outro obstetra também não tinha gostado, então já tava na terceira ne, cheguei nessa que minha amiga me indicou, ai quando vê a colega de trabalho do [nome do companheiro] também ia nessa também gostou sabe, então tipo foi mais ou menos assim, não foi aquela coisa "Ai eu ja conhecia ela há muito tempo" sabe, foi meio assim antes eu só tinha ginecologista, tive que procurar uma obstetra né, então foi aquela coisa assim, coisa meio de cidade grande, eu que sou de cidade pequenininha é bem diferente sabe. E aí então tá cheguei nela, durante as consultas ela respondia as minha dúvidas, sempre foi muito querida e tal só que na hora do meu parto eu achei ela tão fria sabe, eu chamei ela dai pra ela ir fazer meu parto e ela fria, tinha uma médica plantonista lá mais fria ainda né, eu tinha a chance de fazer o parto com o plantonista se eu quisesse né, mas eu achei aquela plantonista meu Deus do céu quase que uma bruxa, muito grossa sabe, aí eu digo "Não, vou chamar a doutora né" liguei já tinha contratado ela né, e aí eu achei assim que sei lá, eu pedi na verdade porque eu tava com muito medo, eu entrei na sala de cirurgia lá sozinha assim né, depois que o [nome do companheiro] veio e eu tava com muito medo assim, sei lá né tudo novo na vida da gente, naquela hora que rompe a bolsa é uma alegria, uma choradeira, uma tremedeira, tudo ao mesmo tempo né e aí eu dizia pra ela assim "Ai doutora segura a minha mão" quando tava fazendo anestesia né daí ela ta segurava a minha mãe aí depois eu disse pra ela "Por favor vai me falando tudo que tu tá fazendo, pra mim ir acompanhando" sei lá, se ela ta cortando, se ta fazendo não sei o que, eu queria pelo menos saber que estágio que tava né, e ela não me falava nada sabe, e aí simplesmente a anestesista ficou do meu lado né, e eu comecei a escutar aquele barulho de bisturi e eu "Ai meu Deus será que ja tão me cortando? Será que ja tão" ai eu ai meu Deus, gente isso me deu uma sensação tão ruim, tão ruim, presa amarrada naquela máquina, naquela cama, sem a tua médica falar contigo, a anestesista que eu nunca tinha visto na frente falando comigo sabe, o [nome do companheiro] do lado coitado acho eu apavorado também com tudo que tava vendo, ele disse que tava vendo tudo né, não falava direito também né, aquilo me deu um nervoso, nervoso, nervoso, uma ansiedade eu dizia assim "Ai me tira daqui" eu dizia pra anestesista, queria que ela colocasse um sedativo assim pra me desligar dai ela disse assim "Não, não não, eu não vou fazer isso na melhor hora da tua vida, depois eu vou te dar um calmante pra ti se acalmar" ela falou né, ai ta o [nome do filho] nasceu ai ela me mostrou primeiro ele assim, baixou aqueles panos me mostrou ele, depois ela veio e trouxe ele do meu lado pra botar o rosto comigo assim, e aí acho que a anestesista deve ter me dado um sedativo, porque dia eu não me lembro mais sabe, então aquela parte de pesar a criança isso foi tudo o [nome do companheiro] que acompanhou eu lembro que eu tinha dito pro [nome do companheiro] assim ó "Quando eles tirar o [nome do

filho] de dentro de mim tu não desgruda do [nome do filho]" sabe aquele medo de mãe de trocar na maternidade, fazer alguma coisa que não pode né, e aí ele foi e colou no [nome do filho] e eu acho que elas meio que me desligaram assim, mas ele não mamou naquela primeira hora, não nada sabe, então todas essas coisas assim que depois eu fui lendo e na verdade vendo que isso aí faltou comigo sabe, talvez eu não, pelo fato de eu não ter lido nada sobre isso, não ter cobrado da médica antes né, foi um erro meu também ela achou bom, que ela ia fazer do jeito que ela achava melhor né, só que depois eu fui lendo e vi que "Ai eu não tive isso, ai eu não tive isso" sabe aquela coisa, ai eu lembro que eu fui pra recuperação ne e aí não sei assim, eu não lembro quanto tempo depois, diz o [nome do companheiro] que foi logo depois, que a enfermeira colocou o [nome do filho] pra mamar né e aí aquele momento eu já tava bem acordada, eu ja tava participando de tudo assim de novo né.

E23: Uhum, e foi assim, e aí a cesárea não foi acho que muito fácil sabe pelo que eu entendi o [nome do filho] tava muito alto, eu não sei como que isso pode acontecer em cesárea mas ela chamou uma outra médica, a outra médica plantonista ajudou dai no meu parto sabe porque sei lá, acho que porque era um neném que tava vindo antes do tempo, não sei, que tava sentado não sei, enfim, chamou ela pra ajudar e aí eu uma coisa que eu me lembro é que eu deitada naquela maca lá e ela subiu em cima de mim três vezes sabe, aqui assim nas minhas costelas assim sabe, com as mãos assim, com os braços sabe, três vezes bem aqui em cima e aí ela foi a primeira vez nada, foi a segunda vez nada, foi a terceira vez eu não sentia assim uma dor horrível mas assim tipo por mais eu estava anestesiada eu ainda senti sabe, sabe eu senti que ela pegou assim nos meus ossos sabe ai eu ai credo sabe, que horror, eu já tava assim meio nervosa né e ainda fazem isso sabe, ai eu senti falta daquela coisa assim "Ó…", né sei lá, "o [nome do filho] ta muito alto" eu não sei explicar em termos técnicos o que que elas me diriam entende mas assim pra mim elas podiam ter me dito "Ó, o bebê tá lá em cima,a gente vai ter que empurrar ele um pouquinho" eu não sei sabe, eu não sei o que se diz, se pode fazer isso ainda ou não sabe, mas assim.

E23: Então tipo isso foi o que me marcou assim sabe, então assim ó pra ti falar a verdade foi cesárea e o [nome do filho] tava sentado e eu sempre disse que eu ia fazer cesárea igual sabe, porque às vezes eu achava que o parto normal não é tão normal, eu tinha medo do parto normal, só que tendo em vista assim que a minha cesárea não foi nada fácil assim ó me faz pensar até se eu não vou tentar um parto normal se eu tiver um segundo filho sabe, porque eu nunca imaginei que eu ia demorar tanto pra me recuperar, que ia ser uma recuperação tão difícil, gente a minha mãe ficou comigo por 45 dias porque a minha mãe não ia embora enquanto eu não tivesse bem, eu fiquei fraca fraca fraca, com muita dor, com muitos gases, completamente inchada sabe, as minhas fotos meu rosto era o dobro sabe, e daí eu tive dificuldade com amamentação no início também sabe, então foi bem difícil assim pra mim, eu fui dar o primeiro banho no [nome do filho] quando ele tinha sei lá acho que um mesinho já, porque eu não conseguia me inclinar, o porquinho que a gente precisa se inclinar pra dar banho na criança naquelas banheirinha alta sabe, eu já não conseguia né, então não foi muito fácil, não foi muito agradável.

E23: Foi bem angustiante assim sabe. Dai ela se contradiz um pouco dizendo que “Ah, não vou te sedar” mas por outro lado não se comunica né, ai fica difícil. Aí eu escutava elas as médicas falando, porque assim ó eu tive um problema também no útero, eu acho que tinha uma variz pelo que ela me explicou, uma veia meio grande eu não sei como que é sabe e eu escutava ela dizendo “Ah, mas isso aqui parece que ela tem quadro de endometriose” umas coisas assim, falando assim entre elas e eu “Ai, será que sou eu” sabe aquela coisa assim né, "Aí será que sou eu, será que elas tão (perda de conexão)”. Eu fiquei assim completamente no vácuo, sabe, “Será que era comigo, será que elas tão falando de mim? Será que elas tão falando de outra coisa? Será que” sabe aquela coisa, que eu me lembre assim foi tudo muito rápido mas ao mesmo tempo que foi rápido foi meio que assustador assim sabe.

Entrevistadora: É, eu te entendo assim né, que não deve ter sido fácil, mas assim só porque eu não quero mais tomar teu tempo né já vou finalizar.

E23: Não imagina, fica tranquila aqui eu vou gerenciando.

Entrevistadora: Mas só pra se quiseres abordar um pouco né, sobre qual, essa pergunta eu faço pra todas as mulheres né, qual tu achas que é a importância do preparo pro parto?

E23: Hoje eu faria tudo diferente (risos) tá, assim primeira coisa eu tava até aconselhando uma amiga minha que vai ter bebê agora próximo do natal, assim ó primeira coisa que eu faria, eu na minha cabeça eu tava numa fase bem "Workaholic" da minha vida tá, trabalhando, trabalhando, trabalhando, tanto assim que eu fui muito cansada ter o [nome do filho] sabe, os primeiros dias pra mim foram bem assim desgastantes porque eu estava cansada de tanto que eu tava trabalhando, então assim, hoje eu faria completamente diferente tá, eu iria trabalhar mas eu ia pedir pra sair de licença um pouco antes né, claro no caso do [nome do filho] 37 semanas então né normalmente as mulheres tem com 38 né, mas eu ia ter me programado pra sair um pouquinho antes né e ia ter me programado agora que eu conheço, agora que eu vi, agora que eu consegui ler um pouco mais sobre isso depois né, que nem eu te falei um erro meu também né, mas enfim, mas eu ia ter pedido pra que fosse um parto um pouco mais humanizado, pra que conversassem comigo sabe, pra que me deixasse ficar um pouco mais pertinho dele não só o rostinho dele, que colocassem ele realmente em cima de mim, sabe aquela coisa assim do primeiro contato né? Pelo menos uma coisa eu gostei que elas respeitaram que elas me perguntaram se eu queria que (perda de conexão).

Entrevistadora: Desculpa, se puderes repetir só essa última partezinha que cortou.

E23: Ata, elas me perguntaram se eu queria que desse banho nele, e eu falei que não, então isso elas me respeitaram que eu queria que ele ficasse com aquela proteção né, o vérnix ali por causa da pele dele né. Mas realmente hoje eu ia fazer tudo diferente, eu ia sair mais cedo do trabalho pra ter aquele tempo pra mim, pra descansar, pra ti ter uma ideia eu acabei de terminar a mala do [nome do filho], a malinha de maternidade, na quinta-feira, nas sexta

estourou a bolsa tu acredita? (risos) tipo assim ó, eu tava muito só trabalho, trabalho, trabalho sabe, então claro isso foi um erro muito grande meu e mas enfim, isso também são coisas que a gente leva né.

E23: Sabe o que, outra coisa que eu faria completamente diferente, completamente diferente, que até foi uma amiga minha que tá morando em Porto Alegre que ela me dizia assim " Ai.." ela teve depressão pós-parto essa minha amiga né dai ela disse assim "Ai, vai lendo um pouco sobre puerpério pra ti se preparar o que muda na tua vida e não sei o que" e sério eu não sei onde é que eu tava com a cabeça, eu pensava assim ó "Eu não vou ler, capaz que isso vai acontecer comigo, não eu to tão bem, eu me sentia uma grávida tão bem, tão bem, tão bem, eu era tão feliz de tá grávida, tão grata porque eu achava que eu não ia mais conseguir engravidar, que eu não via problema em nada, meu astral tava lá em cima todos os dias sabe, então por isso que eu não queria ler sobre problemas, eu não queria pensar em problemas de tão bem que eu tava sabe, e eu dizia assim ó "Capaz, eu não vou ter problemas com essas coisas no puerpério, capaz isso não é pra mim gente, eu to muito bem eu sempre quis isso" sabe aquela coisa assim, aah guria, veio o negócio e veio valendo sabe, primeiro porque assim eu sempre fui muito dorminhoca e de repente eu me vi sem dormir né, e aí eu me vi que nem eu te falei com o [nome do companheiro], que me decepcionou um pouco pra falar a verdade sabe, na questão de não me ajudar, de não participar muito sabe, meio que veio a minha mãe e ele deixou pra minha mãe ajudar, não sei se a minha mãe também tomou o espaço dele, sabe aquela coisa que às vezes a presença da sogra também atrapalha um pouco o nascimento do casal como pais e isso aconteceu aqui em casa também sabe, então hoje eu já ia fazer tudo diferente, eu já ia conversar sobre o parto, eu ia planejar essas coisas sabe, eu ia ler mais sobre o puerpério, sobre a questão psicológica da mulher no puerpério sabe, até depois as minhas amigas me indicaram um livro, cinquenta dias de neblina não sei se tu ja ouviu falar, ai eu naquele ápice do puerpério né, sem sair de casa, trancada dentro de casa com uma criança, o [nome do filho] mamava praticamente de hora em ora gente sabe, uma loucura e dai só a minha mãe, ninguém veio me ver, nenhuma amiga, nem ninguém por causa da pandemia, eu não podia nem botar o pé na esquina sabe, pra não sair de casa, tinha escada no meu prédio, então eu com aquela cesárea que já foi toda meio complicada, me doía tudo pra descer e subir escada né, já tinha que às vezes descer pra levar o [nome do filho] pra consultar então eu evitava de descer e subir, enfim eu evitava de sair de casa e não encontrar ninguém que pudesse estar doente também né e aí a minha amiga fotografava páginas daquele livro pra mim né.

*finalização**
