# Supplementary material for: Adaptation strategies for preparing for childbirth in the context of the pandemic: Roy’s Theory
Source: Rev Bras Enferm. 2024 Jul 29;77(3):e20230159. doi: 10.1590/0034-7167-2023-0159 (PMC11290742; doi:10.1590/0034-7167-2023-0159)
Supplement: Supplementary file 2 [file 0034-7167-reben-77-03-e20230159-suppl02.pdf]

5 MÉTODO

5.1 CARACTERIZAÇÃO DO TIPO DE ESTUDO

Trata-se de um estudo exploratório-descritivo com abordagem qualitativa.

Na pesquisa com abordagem qualitativa, a realidade é múltipla e subjetiva, construída em conjunto com o pesquisador e pesquisado, privilegiando a compreensão, interpretação e sentido que as pessoas estudadas atribuem às suas experiências. Os pesquisadores compreendem que não há neutralidade e que se inserem no processo de pesquisa, a qual não se parte de uma teoria exclusiva, mas esta é construída a partir das contribuições dos sujeitos envolvidos (PATIAS; HOHENDORFF, 2019).

De acordo com Minayo (2017) a pesquisa com abordagem qualitativa trata da intensidade dos fenômenos e suas singularidades. Nesse sentido, trabalha com destaque em dimensões socioculturais expressas por meio de crenças, valores, opiniões, representações, relacionamentos, simbologias, usos, costumes, comportamentos e práticas.

A pesquisa exploratória é utilizada quando o conhecimento acumulado sobre determinado assunto é limitado, na qual visa explorar ainda mais domínios de conhecimentos. É uma estratégia adotada quando o conteúdo publicado sobre o objeto de estudo ainda é insuficiente. Portanto, abordagens exploratórias proporcionam uma familiaridade inicial dos indivíduos a problemas e fenômenos. Mais adiante à uma exploração inicial, a pesquisa descritiva acrescenta valor à construção do conhecimento, visto que, descrever envolve o estudo de um fato ou fenômeno de alguma maneira. Dessa forma, os fatos são observados, registrados, classificados e interpretados da maneira que são percebidos (GOMES; GOMES, 2019).

5.2 CONTEXTO DO ESTUDO

O contexto deste estudo foi o Grupo de Gestantes e Casais Grávidos da Universidade Federal de Santa Catarina, um projeto de extensão criado em 1996 por docentes do departamento de enfermagem da UFSC em parceria com profissionais da maternidade do Hospital Universitário Polydoro Ernani de São Thiago (HU-UFSC).

O grupo é um espaço gratuito, educativo e interdisciplinar de promoção à saúde, cuidado humanizado e autonomia direcionado às gestantes e seus acompanhantes. Se fundamenta nos eixos básicos da humanização do cuidado, autonomia da mulher e a interdisciplinaridade. Tem como um de seus objetivos favorecer o protagonismo, autoconhecimento e empoderamento das

gestantes no enfrentamento do período gravídico-puerperal. Proporciona a troca de saberes e experiências entre as famílias, acadêmicos e profissionais sobre gestação parto e nascimento (VIEIRA *et al*., 2019).

Os encontros antes realizados presencialmente, foram reajustados frente à pandemia, ou seja, suas atividades foram reorganizadas para serem desenvolvidas na modalidade virtual. Inicialmente, as gestantes realizam uma pré-inscrição pelo contato de telefone e uma semana antes de dar início às atividades realiza-se uma confirmação da participação via aplicativo de mensagens. Todas as gestantes preenchem um formulário de cadastro com dados pessoais dos participantes, em seguida são criados dois grupos via aplicativo de mensagens, um destinado à interação entre a equipe e participantes e outro à postagem de materiais educativos.

Ao total são realizados sete encontros semanalmente, às quintas-feiras das 14 às 16 horas, por videoconferências. A definição dos temas a serem discutidos é realizada de acordo com as necessidades elencadas pelas gestantes e seus acompanhantes através do preenchimento de um formulário, envolvendo aspectos do ciclo gravídico-puerperal e medidas de prevenção da Covid-19. Os conteúdos são debatidos por meio de um diálogo entre as mulheres, seus acompanhantes e pela equipe composta por uma psicóloga, enfermeiras, educadora perinatal, docentes e discentes, além de acadêmicas de enfermagem e de psicologia contempladas por bolsas de extensão e pesquisa. Além disso, realiza-se uma visita virtual à maternidade do HU-UFSC por meio da apresentação de um vídeo que apresenta os setores da maternidade.

Após encerrar as atividades do grupo os participantes têm a oportunidade de avaliarem, por meio de um formulário, as atividades desenvolvidas, relatando as contribuições para o processo de gestação, parto, nascimento e para o enfrentamento dessa vivência na pandemia. Também podem ser apontadas críticas e sugestões para o melhor desenvolvimento das atividades do projeto.

Após cerca de um mês do nascimento do último bebê do grupo é realizado um reencontro entre pais e bebês para socialização e compartilhamento de experiências das famílias em relação ao parto e pós-parto. Com autorização, os depoimentos são devidamente gravados em mídia digital, transcritos e armazenados no banco de dados do grupo para posteriormente servirem de amostra para pesquisas. A comunicação dos participantes com a equipe se mantém por volta de seis meses promovendo um espaço direto para sanar as inúmeras dúvidas que surgem nesses primeiros meses. Sendo assim, o projeto acompanha essas famílias por um período, proporcionando a consolidação de alguns vínculos permanentes entre os envolvidos.

As barreiras físicas ultrapassadas pelos meios digitais possibilitam a participação de pessoas de diversas regiões, mas majoritariamente residentes do município de Florianópolis.

Em um ano são realizados quatro novos grupos em que são ofertadas 25 vagas em cada um deles, porém, é possível flexibilizar esse número nessa modalidade à distância. Os grupos são identificados por números e já foram realizados um total de 105 grupos, com participação de 2.111 gestantes e 1.550 acompanhantes, até o momento. Destaca-se que no período de pandemia foram realizados 10 grupos virtualmente, com participação de um total de 304 mulheres e 281 acompanhantes. As inscrições são abertas para mulheres com idades gestacionais semelhantes, pois assim os participantes se identificam com o momento da gestação que vivenciam e podem se beneficiar pela troca de experiências entre si.

5.3 PARTICIPANTES DO ESTUDO

Foram convidadas a colaborar com este estudo mulheres participantes do 96° e 97° grupo de gestantes e casais grávidos na modalidade virtual, realizados durante os meses de março a junho de 2020. Tais grupos foram selecionados pois os participantes vivenciaram a gestação e o parto em um momento inicial da pandemia, onde ainda era um cenário desconhecido para a população. Como critério de inclusão foi considerado: mulheres maiores de 18 anos que tiveram seus bebês entre os meses de março a dezembro de 2020, ou seja, no primeiro ano de pandemia.

Os critérios de exclusão definidos foram: Mulheres que não estavam na lista de contatos participantes do grupo de Whatsapp do grupo 96 e 97. Foram contatadas 45 mulheres e 23 delas aceitaram participar da pesquisa.

5.4 COLETA DE DADOS

A estratégia de coleta de dados se deu por meio de duas técnicas, em entrevistas semiestruturadas e em base documental.

Inicialmente, foi realizado um convite às mulheres participantes do grupo 96 e 97 através de um grupo no aplicativo de mensagens *WhatsApp*® (previamente formado ao iniciar as atividades do grupo de gestantes) para participarem da pesquisa em questão. Em seguida, a mensagem foi enviada de maneira individual a cada uma das mulheres reforçando este convite. Na mensagem do convite, foi esclarecido o título da pesquisa, objetivo e a maneira como seriam realizadas as entrevistas.

Após as participantes aceitarem participar da pesquisa, foi dado início a coleta de dados em base documental no banco de dados do Grupo de Gestantes e Casais Grávidos da UFSC. Os

arquivos dos formulários de cadastro preenchidos pelas participantes foram acessados e os dados sociodemográficos e obstétricos foram coletados a fim de caracterizar essas mulheres. Dentre as 45 mulheres contatadas, 23 aceitaram participar da pesquisa e as demais não retornaram o convite.

As entrevistas foram agendadas conforme data e horário de disponibilidade das participantes, na qual foram realizadas via plataforma virtual *Google Meet*® e *WhatsApp*®. Destaca-se que a autora principal deste estudo recebeu um treinamento prévio para técnica de coleta de dados.

As entrevistas individuais transcorreram no período de 25 de outubro a 02 de dezembro de 2021, tendo duração média de 30 minutos cada. Contou com a participação da autora principal deste estudo, no papel de entrevistadora, e das mulheres que estavam em maioria acompanhadas de seus filhos. Ainda neste momento foi comunicado que a entrevista seria conduzida em um ambiente privativo e utilizado o recurso de gravação de áudio para posteriormente realizar a transcrição de suas falas, mantendo o sigilo de suas informações restrito à pesquisadora.

De maneira introdutória, visando estabelecer uma maior aproximação com as entrevistadas foi realizada uma breve apresentação pessoal e agradecimento pela disponibilidade prestada, seguida por um esclarecimento da temática e objetivo da pesquisa. O roteiro que orientou as entrevistas semiestruturadas buscou respostas ao objetivo deste estudo com as seguintes questões norteadoras: "Pensando no contexto de pandemia, como foi sua preparação para o parto? "; "Qual a data do seu parto"; "Conte-me sobre seu parto. Quais eram seus sentimentos? " e "O que significou participar do grupo de gestantes em relação à preparação para o parto? ". Durante a coleta de dados houve a pretensão de não realizar interferência ou influenciar as respostas, utilizando perguntas abertas e dando espaço para a fala das mesmas que demonstraram estar confortáveis com a estratégia utilizada.

Os dados de identificação já haviam sido preenchidos através da ficha de cadastro ao iniciarem as atividades no GGCG, portanto, como pergunta fechada adicional foi questionada apenas a data do parto. Demais questionamentos sobre o tema não foram fornecidos previamente aos participantes, e não houveram entrevistas repetidas nesse processo.

Como proposta, durante as entrevistas foi sinalizado a possibilidade de entrarem em contato novamente em caso de desejo de complementar seus relatos com alguma informação que possam ter esquecido, mas não houveram retornos posteriores.

A saturação de dados é utilizada para estabelecer a quantidade de dados e entrevistas suficientes uma vez que os dados se tornam incidentes, ou seja, quando nenhuma nova informação ou tema for registrado, possibilitando a interrupção da coleta (MOURA *et al.*, 2022). Os dados foram saturados na vigésima primeira entrevista, portanto, foi optado por entrevistar as 23 mulheres que se dispuseram a participar deste estudo. Ao finalizar as entrevistas, os áudios foram identificados e arquivados em um dispositivo eletrônico para serem transcritos na íntegra.

5.5 ANÁLISE DE DADOS

A análise de dados corresponde ao momento de organizar e interpretar os dados obtidos a fim de responder os questionamentos da pesquisa e alcançar os objetivos propostos, expandindo o conhecimento acerca do tema. A abordagem qualitativa, por apresentar uma variedade de metodologias de pesquisa, permite maiores possibilidades relativas ao processo investigativo, as técnicas utilizadas em cada uma de suas etapas são definidas a partir da necessidade da pesquisa e escolha do autor (ROSA; MACKEDANZ, 2021).

Partindo das informações coletadas nas entrevistas e nos registros de identificação das participantes, o procedimento analítico do presente estudo foi baseado na proposta de Minayo (2014) adaptada para o propósito da presente investigação. Tal estratégia consiste em desvendar os núcleos de sentido que integram uma comunicação, cuja frequência ou presença dos mesmos atribui significado ao objeto analítico. Neste método a análise de dados qualitativos é operacionalizada nas seguintes etapas: pré-análise, exploração do material e tratamento dos resultados obtidos e interpretação.

Segundo Minayo (2014), a **pré-análise** consiste na etapa inicial de análise de dados, em que são escolhidos os materiais a serem analisados, retomado os objetivos, questionamentos e pressupostos iniciais da pesquisa. Neste momento foi realizada uma leitura flutuante para compreensão geral do conteúdo e designação dos rumos da análise. Foram delimitados os conceitos teóricos que orientaram a análise dos dados e a forma de categorizá-los. Após transcritas, as entrevistas foram lidas na íntegra de modo a estabelecer o primeiro contato inerente ao processo interpretativo, conhecer as palavras chaves ou frases, delimitação do contexto de compreensão, as unidades teóricas e recortes do texto relacionados ao tema. Os dados de identificação dos participantes, inerentes da coleta em base documental, foram armazenados em uma planilha com a frequência absoluta e a frequência relativa em porcentagem.

A fase de **exploração do material** é definida por Minayo (2014) pela estruturação de recortes considerados relevantes no texto e sua categorização. O referencial teórico escolhido para guiar a análise dos dados desse estudo foi o Modelo de Adaptação de Callista Roy, visto que a teórica apresenta uma visão peculiar e oportuna para descrever a interatividade da pessoa com o ambiente e seu processo de adaptação. Nesse sentido, situa a enfermagem como promotora da saúde, de qualidade de vida e/ou de morte com dignidade (ROY, 2009; SILVA, R. *et al.*, 2020). Após uma leitura exaustiva, sob a ótica do referencial teórico, foram definidas as categorias analíticas do estudo, sendo essas: “Estímulos focais, contextuais e residuais no preparo para o parto”; “Modos adaptativos: grupo de gestantes como facilitador do processo de adaptação” e “Feedback positivo ao preparo para o parto”. Os trechos das falas foram identificados por códigos, agrupados por temáticas e inseridos em um documento, analisando as semelhanças e divergências nos textos de modo a criar conexões que possibilitem a interpretação dos resultados alcançados.

Para finalizar esse processo, conforme Minayo (2014) através do **tratamento dos resultados obtidos e interpretação** foi realizado uma leitura na íntegra para então dar continuidade ao processo de reflexão sobre o material empírico e associação com o referencial teórico definido. Assim, ocorreu o processo de inferências e interpretações inter-relacionando com o referencial teórico adotado e a literatura estudada.

A figura 2 apresenta uma síntese da coleta e análise de dados.

Figura 2 – Fluxograma da coleta e análise de dados

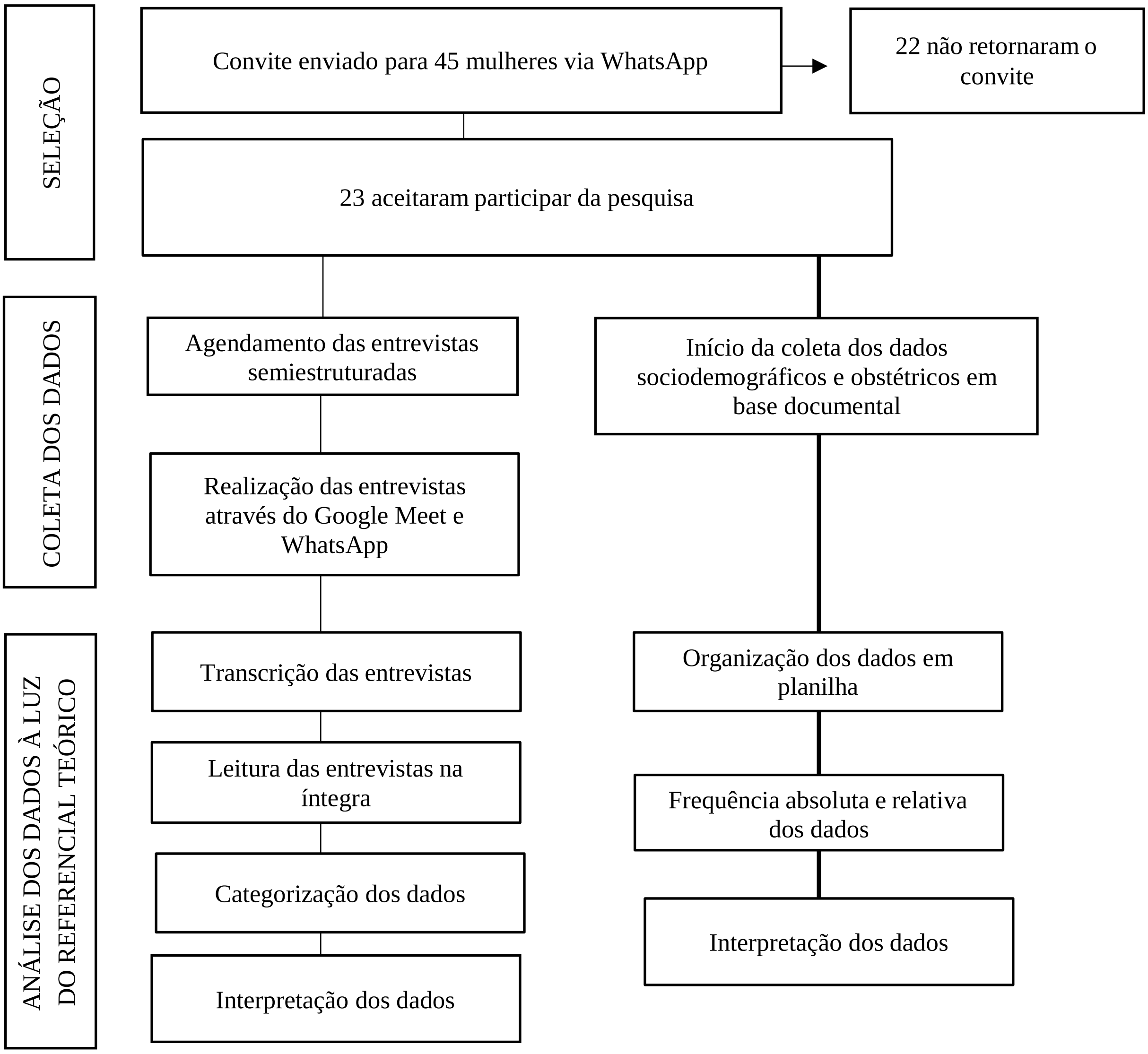

Fonte: Elaborado pela autora (2022)

5.6 ASPECTOS ÉTICOS DA PESQUISA

O presente estudo está vinculado ao macroprojeto de extensão intitulado: “20 anos do Grupo de Gestantes e Casais Grávidos: trajetória histórica, perfil, impacto, percepções e contribuições para os envolvidos”, submetido e aprovado no Comitê de Pesquisa com Seres Humanos da Universidade Federal de Santa Catarina, cumprindo os termos da Resolução 466/2012, sob parecer n. 2.051.643, CAAE 63797417.4.0000.0121.

Esta pesquisa não expõe os participantes a qualquer risco de vida, de sua saúde ou integridade. Não há nenhum ônus ou recompensa financeira ao participar da pesquisa. Todos os dados coletados são confidenciais e utilizados somente para este fim, preservando o anonimato dos envolvidos.

Ao recepcionar as mulheres com câmera e áudio ativados na plataforma *Google Meet®*, foi reforçado a assinatura prévia do Termo de Consentimento Livre e Esclarecido (TCLE) que haviam sido realizadas autorizando a participação na pesquisa, enfatizando a não obrigatoriedade desta atividade. Este documento é apresentado aos participantes desde o primeiro encontro para autorização e coleta das assinaturas daqueles que decidirem participar voluntariamente da pesquisa. Logo no início, são esclarecidos os objetivos da pesquisa e garantia da possibilidade de desistência a qualquer momento se assim for a vontade do participante, sem nenhum tipo de prejuízo. Os documentos são apresentados em duas vias, sendo uma do participante e outra do pesquisador.

Para o desenvolvimento desta pesquisa foram respeitadas as resoluções que guiam os aspectos éticos sobre as pesquisas com seres humanos 466/2012 e 510/2016, assim como o Ofício Circular Nº 2/2021/CONEP/SECNS/MS que apresenta as orientações para procedimentos de pesquisa em ambiente virtual.

Garantindo o anonimato das participantes, todas foram nominadas pela letra E de entrevistada, seguida de um número ordinal (1 a 23), de acordo com a ordem das entrevistas. Desta forma, a primeira entrevistada foi nomeada de E1 e a última de E23.

REFERÊNCIAS

GOMES, Alex Sandro; GOMES, Claudia Roberta Araújo. Classificação dos tipos de pesquisa em Informática na Educação. In: JAQUES, Patricia; PIMENTEL, Mariano; SIQUEIRA, Sean; BITENCOURT, Ig (ed.). **Metodologia de Pesquisa Científica em Informática na Educação**: concepção de pesquisa. [S.I.]: Sociedade Brasileira de Computação, 2019. Cap. 18. p. 1-535. Disponível em: https://metodologia.ceie-br.org/wp content/uploads/2019/06/livro1_cap4.pdf. Acesso em: 24 jan. 2022.

MINAYO, Maria Cecília de Souza. Amostragem e saturação em pesquisa qualitativa: consensos e controvérsias. **Revista Pesquisa Qualitativa**, São Paulo, v. 5, n. 7, p. 1-12, abr. 2017. Disponível em: https://editora.sepq.org.br/rpq/article/view/82/59. Acesso em: 07 set. 2021.

MINAYO, Maria Cecília de Souza. **O desafio do conhecimento**: pesquisa qualitativa em saúde. 14 ed. São Paulo: Hucitec. 2014. 407. ISBN 978-85-271-0181-3.

MOURA, Cleson Oliveira de et al. Percurso metodológico para alcance do grau de saturação na pesquisa qualitativa: teoria fundamentada. **Revista Brasileira de Enfermagem**, [S.L.], v. 75, n. 2, p. 1-9, 2022. FapUNIFESP (SciELO). http://dx.doi.org/10.1590/0034-7167-2020-1379. Disponível em: 83 https://www.scielo.br/j/reben/a/h6skK6tnvW4phBYzvxpWJ3Q/?format=pdf&lang=pt. Acesso em: 22 jun. 2022.

PATIAS, Naiana Dapieve; HOHENDORFF, Jean Von. Critérios de qualidade para artigos de pesquisa qualitativa. **Psicologia em Estudo**, [S.L.], v. 24, p. 1-14, 21 nov. 2019. Universidade Estadual de Maringa. http://dx.doi.org/10.4025/psicolestud.v24i0.43536. Disponível em: https://www.scielo.br/j/pe/a/BVGWD9hCCyJrSRKrsp6XfJm/?lang=pt&format=html. Acesso em: 07 set. 2021.

ROSA, Liane Serra da; MACKEDANZ, Luiz Fernando. A análise temática como metodologia na pesquisa qualitativa em educação em ciências. **Atos de Pesquisa em Educação**, [S.L.], v. 16, p. 1-23, 2021. Disponível em: https://bu.furb.br/ojs/index.php/atosdepesquisa/article/view/8574/4963. Acesso em: 26 jan. 2022.

ROY, Sister Callista. **The Roy adaptation model**. Third edition. Upper Saddle River, New Jersey: Pearson, 2009. 553 p.

SILVA, Roger Rodrigues da et al. As teorias de enfermagem de Roy e Orem Intrínsecas à sistematização da assistência de enfermagem para promoção da saúde. **Brazilian Journal Of Development**, Curitiba, v. 6, n. 7, p. 52049-52059, 2020. Disponível em: https://www.brazilianjournals.com/index.php/BRJD/article/view/14001#:~:text=As%20teorias%20de%20enfermagem%20atuam,para%20a%20promo%C3%A7%C3%A3o%20da%20sa%C3%BAde. Acesso em: 19 mar. 2022.

VIEIRA, Amanda Nicácio et al. Grupo de gestantes e/ou casais grávidos: um processo de construção coletiva (1996-2016). **Escola Anna Nery**, [S.L.], v. 23, n. 2, p. 1-8, 2019. 90 FapUNIFESP (SciELO). http://dx.doi.org/10.1590/2177-9465-ean-2018-0221. Disponível em: https://www.scielo.br/j/ean/a/KpGLdNf8DFX9zbqvbTVwgZw/?format=pdf&lang=pt. Acesso em: 21 jun. 2021.
