# Supplementary material for: Adaptation strategies for preparing for childbirth in the context of the pandemic: Roy’s Theory
Source: Rev Bras Enferm. 2024 Jul 29;77(3):e20230159. doi: 10.1590/0034-7167-2023-0159 (PMC11290742; doi:10.1590/0034-7167-2023-0159)
Supplement: Supplementary file 4 [file 0034-7167-reben-77-03-e20230159-suppl04.pdf]

Título do conjunto de dados:

Data for: REBEn-2023-0159: Estratégias de adaptação no preparo para o parto no contexto de pandemia: teoria de Roy

Pesquisadora correspondente:

Letícia Pickler

Universidade Federal de Santa Catarina, Florianópolis, Santa Catarina, Brasil.

E-mail: picklerleticia@gmail.com

Data de coleta dos dados:

Dados referentes a coleta de dados realizados entre o período de outubro a dezembro de 2021.

Visão geral dos dados e arquivos:

- Documento em formato pdf. nomeado “Entrevistas na íntegra”: Trata-se de um documento contendo a transcrição das 23 entrevistas realizadas no período de 25 de outubro a 02 de dezembro de 2021 com as participantes da pesquisa.

- Documento em formato pdf. nomeado “Temas que surgiram durante as entrevistas”: Este documento foi elaborado durante a análise dos dados, que transcorreu no primeiro semestre de 2022. Foram selecionados os trechos das entrevistas relacionados ao tema de modo a estabelecer um direcionamento do contexto de compreensão. Logo, este documento abarca alguns trechos das entrevistas que foram selecionados e distribuídos por temas para melhor compreensão e análise dos dados.

- Documento em formato pdf. nomeado “Perfil das participantes - manuscrito”: Trata-se de uma planilha elaborada através da plataforma Excel, durante o período de coleta de dados (outubro a dezembro de 2021). De modo a caracterizar o perfil das participantes, após confirmação do convite para participar da pesquisa, foram acessados os arquivos do formulário de cadastro contendo dados sociodemográficos e obstétricos através do banco de dados do Grupo de Gestantes e Casais Grávidos. Os dados foram apresentados com a frequência absoluta e a frequência relativa em porcentagem.

- Documento em formato pdf. nomeado “Metodologia – REBEN”: Trata-se de um documento contendo a descrição detalhada de coleta de dados para realização desta pesquisa, elaborado no período de 2021 à 2022.

Descrição dos métodos de coleta ou geração dos dados:

A coleta de dados ocorreu por meio de duas técnicas, em base documental e entrevistas semiestruturadas, no período de outubro a dezembro de 2021. As mulheres foram contatadas através do WhatsApp® e, após o aceite do convite, foram acessados os arquivos do formulário de cadastro contendo seus dados sociodemográficos e obstétricos através do banco de dados do Grupo de Gestantes de modo a caracterizar o perfil das participantes.

As entrevistas foram conduzidas pela autora principal da pesquisa e ocorreram por meio das plataformas Google Meet® e WhatsApp® em data e horário de preferência das participantes, com duração média de 30 minutos. A maioria das participantes estavam acompanhadas de seus filhos, no entanto, não houve interferência na privacidade das respostas. Durante todo o processo de coleta de dados, as mesmas foram informadas que a qualquer momento poderiam solicitar a interrupção da entrevista.

Durante as entrevistas foi utilizado um roteiro com perguntas abertas, em que foram abordados assuntos relativos à percepção e experiência da mulher sobre o preparo para o parto e parto e sua participação no grupo de gestantes. Para captura dos dados foi utilizado recurso de gravação de áudio. Nenhum participante manifestou desconforto durante a entrevista. As perguntas não foram fornecidas previamente às participantes e não houveram entrevistas repetidas.

Os áudios foram transcritos na íntegra pela autora principal e armazenados em documentos identificados com o nome das participantes. Os dados de identificação foram armazenados em uma planilha do Excel com a frequência absoluta e a frequência relativa em porcentagem.

Descrição dos métodos usados para o processamento dos dados:

O procedimento analítico do presente estudo foi baseado na proposta de Minayo, adaptada ao propósito da presente investigação, na qual incluiu as seguintes etapas: pré-análise; exploração do material e tratamento dos resultados obtidos e interpretação. Ainda, o referencial teórico definido para guiar a análise de dados foi a teoria da adaptação de Callista Roy.

Durante a pré-análise, as entrevistas transcritas foram lidas na íntegra de modo a estabelecer as etapas iniciais do processo interpretativo. Foram selecionados os trechos

relacionados ao tema de modo a estabelecer um direcionamento do contexto de compreensão.

Durante a exploração do material, foram definidas as categorias analíticas com base no referencial teórico adotado. No tratamento dos resultados foi realizada uma leitura na íntegra visando estabelecer conexões e inferências dos dados, dando continuidade ao processo analítico em correlação com o referencial teórico definido previamente.

Após as entrevistas serem criteriosamente analisadas, os dados foram agrupados em três categorias guiadas pelo referencial teórico de Roy: “Estímulos focais, contextuais e residuais no preparo para o parto”; “Modos adaptativos: grupo de gestantes como facilitador do processo de adaptação” e “Feedback positivo ao preparo para o parto”.

Informações específicas dos dados:

Os trechos das entrevistas foram codificados por letras “E” de entrevistadas seguidas de um número ordinal (1 a 23), de acordo com a ordem das entrevistas.
