# Supplementary material for: Adaptation strategies for preparing for childbirth in the context of the pandemic: Roy’s Theory
Source: Rev Bras Enferm. 2024 Jul 29;77(3):e20230159. doi: 10.1590/0034-7167-2023-0159 (PMC11290742; doi:10.1590/0034-7167-2023-0159)
Supplement: Supplementary file 5 [file 0034-7167-reben-77-03-e20230159-suppl05.pdf]

1. **PREPARO PARA O PARTO:**

E1: Em junho eu entrei em trabalho de parto e em contato com o obstetra, em contato com a fisio, eu tive uma aula de parto com ela online e aí lendo né. Minha preparação foi leitura, vendo vídeos, é , o grupo também né, proporcionou ali até o final as aulas, então a gente trocando muita ideia com as meninas do grupo, assim que uma ia ganhando, ia parindo né, cada uma compartilhava o relato, falava dos medos, falava das angústias, de como que foi, se pode entrar, quem não pode entrar, se teve acompanhante.

E2: A maior parte mesmo foi através do grupo, eu fiz antes desse do HU, nós os dois participamos do grupo do IFSC porque era a noite e ele podia ir, nesse do HU ele não ia poder participar então a gente participou do grupo do IFSC e aí elas passaram bastante vídeo, bastante informação assim, então pra ele a maior parte da informação foi essa assim, aí depois eu participei sozinha porque era de tarde os encontros do HU e o resto assim, elas colocavam materiais ali pra nós, porque a gente fez um grupo de gestantes também né, esse grupo se formou com as gestantes e a gente trocava informação através dali, bastante informação eu seguia muita obstetra também no instagram, grupo de parto então eu buscava bastante informação ali também.

E2: Desde a gestação, eu fazia todos os dias, eu inspirava soltava o ar e relaxava o períneo, eu fazia as respirações associadas ao relaxamento do períneo e fazia as vocalizações também. Tem uma vocalizações, eu não sei se tu conhece mas tem umas vocalizações no youtube que eu acho que é canto carnálico se eu não me engano, que é assim associada também, faz a vocalização associada a respiração e aí relaxa. E foi o que me ajudou assim, na hora da dor mesmo.

E3: A minha preparação já desde de, desde bem cedo assim foi dupla né, que era frequentando o obstetra e fazendo as consultas com a parteira, ai isso seguiu normal assim e aí eu fui me cercando de orientação, tanto de amigas que seguiam a mesma linha que eu queria que já tinham passado por aquilo, quanto de profissionais de internet que eu fui descobrindo e que eu fui tendo uma afinidade né, toda essa preparação ela foi muito tranquila assim, ela foi bem encorajadora, eu tava, antes de eu engravidar, até quando eu me engravidei assim, na semana que eu descobri que eu tava grávida eu lembro que eu fui ver uns vídeos de parto assim porque nunca tinha visto realmente né, tipo agora tava em mim, tava ali o neném ia ter que sair e eu fui ver, eu pensava meu Deus nunca que eu vou fazer um parto normal, nunca que eu vou deixar um negócio daquele tamanho passar por aquele buraco ali e aí essa minha preparação foi bem engrandecedora do meu potencial enquanto mulher, de gerar, confiar na minha natureza e no que eu sou capaz de fazer né, que hoje eu considero assim, que a nossa natureza é perfeita.

E4: O que eu fazia pra me preparar… aí fisicamente eu não fazia quase nada, eu só caminhava, caminhava bastante, ficava caminhando caminhando, e aí o próprio, procurar o curso né, já foi também uma, um passo assim pro preparo e fazia muita leitura, muita muita

leitura na internet mesmo né, e assistir vídeo, tudo que é possível assim, isso até foi uma questão que a gente acabou ficando em home office e tudo se voltou pra frente de uma tela, então assim, a enxoval, assista vídeo de enxoval, a anestesia, assiste vídeo sobre anestesia, a parto normal, assiste vídeo sobre parto normal (risos). Tudo então foi muita leitura e video na internet mesmo né, aí o grupo também a gente só teve um encontro presencial né, todos os outros foram remotos, também foi muito útil assim e basicamente foi isso assim, e pesquisar sobre maternidade, ficar pesquisando sobre vários temas, eu acho que foi isso assim.

E5: Ai eu fiz tanta coisa, a gente busca muita informação na internet também isso eu não posso negar, principalmente eu sou assim praticamente viciada em internet e instagram, então assim, comecei a seguir muita gente no instagram, de amamentação, de parto, de fisioterapia pélvica e até enxoval, de tudo tudo tudo, a gente fica doidinha assim, começa a procurar informação de tudo quanto é canto, e da internet então. E eu fiz fisioterapia pélvica no meu pré-natal que eu acho que também foi fundamental pra me preparar pro trabalho de parto.

E7: Tentei me preparar lendo, vendo vídeos, o grupo de gestantes ajudou bastante. Fazia minha atividade física em casa mesmo. Porém em virtude da pandemia acabei não buscando ajuda profissional. Acho essencial uma preparação. São muitos sentimentos e você se sentir entendendo pelo menos o processo ajuda.

E8: Existem poucas coisas que eu planejei na minha vida inteira. Mas ser mãe sempre foi a maior delas. Desde muito pequena eu sempre soube que, casando ou não, eu seria mãe de alguém. Isso pulsou sempre muito forte dentro de mim. [...] A [nome da filha] foi completamente planejada. [...] Desde o quinto mês de gestação a gente esteve com a nossa maravilhosa doula, que com muito afeto e dedicação nos conduziu por uma jornada de encontros e estudos muito maravilhosa. Em casa a gente assistia todos os documentários, filmes, lia livros e tudo o mais possível pra se preparar pra esse momento. Acompanhamos no posto exames, fizemos ultrassons.[...] Mudei toda a minha dinâmica alimentar, fiquei um mês e meio sem comer açúcar, quase sem frutas, controlando cada detalhe, fazendo alongamentos específicos, massagem no períneo, internalizações, kegel, cócoras e tudo mais que poderia me ajudar na hora do parto. Quando começaram as minhas contrações na quinta à noite, eu me senti calma, preparada e amparada.

E8: Estudei tudo que eu podia, li vários livros, eu tinha doula e fiz toda a preparação física, mental e psicológica que eu podia fazer, me preparando pra essa questão da maternidade. Minha gravidez foi completamente planejada né, a gente, eu realmente decidi para com o anticoncepcional e a gente tava tentando, a gente tentou por uns dois mesinhos e eu já fiquei grávida, então a gente fez bastante essa preparação,

E9: Eu estudei sobre parto durante a gestação e fiz exercícios pensando em um parto normal, mas sabendo que não sou eu quem controla tudo para escolher isso, ou seja, poderia ser de outro jeito e estava tudo bem. [...] A preparação [...] foi com estudo e relato de uma amiga que já teve duas bebês por parto normal. Conversei com ela para saber bem como era e tirar minhas dúvidas.

E10: De preparação pro parto, eu e meu marido a gente sempre leu muito, a gente buscou também algumas fontes confiáveis, algum material do IFSC e também depois o material que a gente recebeu, material de suporte do HU também, nós fizemos parte também do grupo de gestantes lá no IFSC que daí foi antes da pandemia né, e quando a gente tava começando a gente fez um encontro presencial com o HU né, e aí depois a gente precisou interromper porque a gente entrou em lockdown, a gente não pode dar continuidade.

E10: Bom, durante toda a minha gestação eu me preparei, eu fiz o curso, eu treinei durante toda minha gestação, já treinava antes então continuei treinando até a gente entrar em lockdown eu continuei treinando assim, a minha alimentação também, fazia monitoramento com o nutricionista então tava tudo bem certinho é, eu tava bem relaxada assim, mentalmente também, eu meditava algumas vezes e conversava muito com a [nome da filha] também, lia algumas coisas, alguns livros sobre criação de filhos que ajudaram bastante, então esse foi assim um preparo do meu corpo para o momento ali, é um trabalho mental bem intenso, eu quis desistir em alguns momentos mas é bom que a dor vem, a dor vai e a gente logo muda de ideia, então foi mais tranquilo.

E11: O que eu fiz foi estudar bastante o assunto, tipo o grupo em si me ajudou demais, demais mesmo, coisas que eu nem imaginava sabe falaram lá e material que foi enviado tudo, eu acho que o meu principal a minha principal preparação foi isso e daí por fora eu tentava pesquisar um pouco, tentava manter a calma, não pensar que nem todo mundo pensa que “ah vai ser horrível, não vou aguentar, não sei o que” eu já penso o contrário, eu penso “não o corpo foi feito pra isso, então vai dar tudo certo” era esse o tipo de coisa que eu fazia mais pra me preparar sabe.

E12: Eu comecei a assistir partos e só chorava, chorava, chorava, e eu comecei a ler algumas coisas sobre, livro sobre parto, sobre essa questão do parto ativo, eu lembro que eu li o livro “parto ativo”, li um livro sobre a “eutonia do parto” que é um livro que foi escrito por uma argentina sobre o trabalho da eutonia no processo de gestação e de parto, a questão da vocalização no parto, enfim, e essa coisa do parto humanizado, e meu companheiro foi junto também a gente fez um curso pra pais sobre gestação, parto e puerpério, que era pra homens, mas eu acompanhei ele algumas vezes, a gente leu todos os processos do parto. [..] eu sou uma pessoa muito agilizada, que estuda, que gosta de saber o que tava, então eu estudei muito, eu li muita coisa.

E14: Eu fiz o curso do HU, eu participava de uma roda de gestantes de São Paulo também que daí como teve a pandemia eles acabaram fazendo virtualmente e daí possibilitou que pessoas de outros lugares participassem, e essa roda de gestantes é com pessoas que trabalham com medicina antroposófica então tem uma perspectiva bem do parto humanizado né, e também participei de rodas de gestantes de parto humanizado aqui de Floripa, assisti alguns vídeos, aí em casa eu fazia um pouco de yoga, fazia um pouco de dança, mas tudo dentro de casa né, sem sair e é isso assim, sempre tentando estudar, lia um pouco mas foi isso que eu fiz de preparação assim, nada muito específico.

E15: Pra me preparar pro parto eu li bastante, eu pesquisei bastante, li livro, pesquisava, o grupo de apoio do HU que teve todo aquele respaldo, foi online, foi ótimo, além disso pesquisa, pesquisa, me preparei pro parto de maneira fisioterapia pélvica, em casa, tipo bem autodidata, eu fui atrás das informações além do que vocês já tavam passando né, livros e tentei não focar na pandemia, por mais que aquele momento tivesse ali existindo né, a única coisa que eu tentava entrar mais por dentro era se tinha alguma coisa em relação a gravidez, a parto, os riscos e naquela época bem no começo não tinha tanta informação, aí foi isso que eu fiz pra tentar uma gravidez mais tranquila, não via jornal, tentava não focar porque sabia que o psicológico ia abalar né.

E17: A minha preparação foi basicamente o curso da UFSC que foi online, com a minha doula que foram consultas online, ai ela passou um monte de material pra eu ler e eu fiquei em casa só estudando, entrei em grupo de mães pela UFSC, pelo grupo da UFSC e por conta da minha obstetra que ela me adicionou num grupo, a minha doula também me adicionou num grupo de whatsapp, então ai tudo online tudo meio virtual sabe. [...] mas também tudo online e estudando em casa, lendo livros e material em casa.

E19: Então, eu acho que essa questão eu refleti muito durante todo o processo da gestação e depois principalmente nos grupos assim né, eu participei do grupo da UFSC e também eu fiz parto domiciliar então eu tinha outro grupo da preparação pro parto domiciliar né, então eu tive meio que duas preparações assim e eu refleti muito sobre a questão de que as pessoas gastam muito tempo na questão do enxoval, de preparar o quarto, de preparar toda essa parafernalha que no geral a gente não usa e não se prepara emocionalmente né. [...] Então, essa parte de lidar com a parte hormonal assim né, as mudanças, eu não me reconhecia eu não sabia o que tava acontecendo com o meu corpo né, isso foi muito assustador, foi horrível essa parte e por mais que, que eu soubesse que eu ia passar por aquilo parecia tipo, era algo desconhecido né, então foi bem, eu acho que a palavra melhor é assustador mesmo né, mas ao mesmo tempo tinha seus lados bons né, ver ela mexer, conversar com ela e ela responder, tipo eu falava em ela e ela mexia era muito gostoso esse processo assim e eu acho que preparar pra essas questões das oscilações o puerpério assim tipo, saber que eu ia ter esse momento pós-parto também foi bem importante né, acho que a preparação emocional foi o que mais, o que mais fez diferença pra mim nesses grupos né e o parto em si eu tinha com a questão da pandemia né, nesse processo do parto, eu pensava em ter em hospital e coisa e tal quando eu pensava na possibilidade de, na época que eu pensava na possibilidade de engravidar, ai me vi grávida na pandemia, pensar em hospital "e se acontece alguma coisa comigo? e se eu pego covid? e se ela pega covid?" tipo aquela, porque tava muito incerto né naquela época, a gente não sabia nada sobre nada. [...] No parto assim e eu me preparei muito pra, pra tipo saber que eu dava conta assim, eu acho que a melhor, a melhor preparação é isso, de saber que eu dou conta, dou conta se tiver que ir pro hospital, dou conta se tiver que, eu queria parto normal né, eu consegui, mas também me prepararei pra ter cesariana, me preparei pra ir pro hospital, me preparei pra essas possibilidades assim, eu não sonhei, eu acho que tem diferença sim, penso né, que tem muita diferença entre as outras mulheres que eu conversei assim de idealizar como que vai ser seu parto assim, eu não idealizei sabe. [...]

Eu tinha uma insegurança muito grande porque eu tenho questões com o coco assim, e aí tinha uma insegurança muito grande de fazer coco na hora do parto. [...] então eu tive esse momento também de me preparar pra isso, de lidar com essas questões né, tipo meio que tabu pra mim, de eu fazer coco na frente de outra pessoa e eu me senti muito acolhida assim nesse processo, ter esses grupos, de ter as dúvidas, das angústias poder compartilhar foi bom assim, uma coisa que fez muita diferença pra mim.

E19: Eu acho que essa questão de desmistificar alguns ideias assim é o principal ponto, sabe, tipo por exemplo a questão da dor, a lógica de sentir prazer durante o parto não é uma coisa trabalhada e muitas mulheres sentem né, eu acho que esses pontos não são muito falado, não pra criar um ideal né, porque dai as mulheres podem ir pra um caminho tipo "Ah, eu preciso sentir prazer durante o parto" e não né mas, pra ver que não é uma coisa assim nossa, porque muitas mulhere seu acho que não tiveram um parto bom muito por essa questão de não ter seu tempo respeitado sabe. [...] por exemplo a minha mãe quando me pariu né, ela disse que tipo tinha um monte de gente em cima dela e cada um dizendo um negócio tipo "Ai, respira assim. Aí anda" tipo as pessoas indicando o que que ela devia fazer e não ela sentindo o que que ela devia fazer, o sentir o próprio corpo eu acho que é uma coisa essencial assim. Mas também tem um ponto de autoconhecimento assim né, eu acho que eu não sei o quanto que só a informação muda tudo isso, tipo porque como eu te falei eu tinha muito conhecimento sobre o meu corpo então foi muito assustador o começo da gestação porque eu não conhecia mais meu corpo né tipo "O que que é isso que eu to sentindo, o que é essas sensações, isso tudo é novo assim" e tem pessoas que não se dão conta assim do que tão sentindo de como que ta, de saber como é o funcionamento do corpo assim né, então acho que tem vários pontos né, mas eu acho que desmistificar esses ideais eu acho que é o principal sabe, trabalhar tipo por exemplo, trabalhar que às vezes tem que ser cesárea né, pra mulheres que idealizam um parto normal e "eu preciso ter parto normal" assim como o contrário, pra mulheres que "não, meu Deus eu vou morrer, tipo de dor, não quero passar por isso" que na verdade o parto normal pode ser prazeroso, pode ser um momento bom e todos os benefícios né, tanto pra mulher quanto pra criança né do parto normal, ter os dois contrapontos de tudo assim pra tá preparada pras duas situações.

E20: Eu acabei fazendo fisioterapia pélvica também e me ajudou a ter o conhecimento da região, de conseguir poder trabalhar a musculatura, acredito que seria bem importante pro momento do parto assim, acho que se pudesse ter mais esses momentos do SUS seria bem legal pras mulheres sabe, o que a gente orienta ainda é pouco e com certeza a partir de agora com o que eu tive posso orientar diferente né, acho fundamental ter esse preparo.

E21: Ah, o que eu fiz na verdade foi um programa que eu ia uma vez por semana, o nome é PPM, Physical não sei o que lá mommy uma coisa assim, que é já no preparo pro parto, um serviço que tem aqui em Joinville que é o nome do lugar é espaço mãe bebê então trabalha com a fisioterapeuta né pra fazer fortalecimento da musculatura tudo essas coisa né, só que não trabalhava tanto, trabalhava um pouco o assoalho pélvico mas era mais em geral assim né, alongamento e tudo mais, fiz também a fisio pélvica mas eu fiz poucas sessões porque eu ficava com receio de ir né por conta da pandemia, dai também não botei muita fé que ia fazer

algum resultado daí achei que seria mais exposição do que tá ajudando assim, ficar saindo de casa naquele momento.

E22: Não romantizei muito nada a respeito do parto mas a gente se informou muito assim, a gente estudou bastante e queria que fosse parto normal né, por todos os benefícios pra ela e pra mim teoricamente né. [...] nos preparamos e dentro do possível, com todas as orientações da minha obstetra e com os grupos de apoio. [..] A gente gosta de ler, gosta de se informar então assim a partir tanto dos grupos que foi formado pela minha obstetra [...] a gente trocava ideias também, ela mesma passava algumas bibliografias assim a título de sugestão, é muito louco porque tu começa a seguir uma ou outra no instagram dai ja vai aparecendo outras coisas quando tu vê tu ja ta seguindo só perfil sobre isso, médicos e pessoas que falam sobre maternidade, e aí no grupo também acabo, não lembro quem sugeriu algumas leituras, e no fim tu vai ver são quase sempre algumas dessas leituras coincidem sabe, é bem engraçado, tem alguns livros que são meio que clássicos da maternidade.

E23: Eu vou te dizer que eu era uma grávida bem relax assim sabe, eu pensei assim que eu não ia ler muita coisa porque como eu sou ansiosa né, aí eu ficava pensando assim que isso talvez ia piorar minha situação sabe, então eu não fui assim de ler muito eu pensava assim ó “Não eu vou gerenciando a medida que as coisas forem acontecendo”, então eu participei das aulas ali do grupo né, tinha umas que eu não conseguia ver às vezes pela internet né, mas uma que eu me lembro assim bastante que eu tirei bastante dúvida até foi justamente aquela do parto em si, do que que ia acontecer primeiro sabe, que eu não sabia assim se podia romper a bolsa, se podia, sabe eu não sabia assim a ordem das coisas, se contraia primeiro, se rompia a bolsa primeiro, não sabia de nada assim né e que cor que eram os líquidos que iam sair né, o que que a gente tinha que cuidar, essas coisas assim eu não sabia absolutamente nada então essa aula de parto eu me lembro que eu participei do início até o final e tirei bastante dúvidas sabe e isso pra mim foi bom né, que dai eu chamava o meu marido, os dois trabalhando em casa, falava “Ó ó” pra cuidar também né.

2. IMPORTÂNCIA DO PREPARO PRO PARTO

E3: Graças a Deus existe a ciência que possibilita que em situações como a minha em que a minha bebê não tava bem, podia ser que se tivesse seguido o plano talvez tivesse seguido tudo certo, mas talvez não desse, então a gente pode escolher não correr aquele risco, mas que eu vejo assim e ouço de algumas amigas é as cesáreas desnecessárias, a má informação, a gente vê que há um interesse financeiro no controle do tempo, pra não ficarem muito tempo lá em trabalho de parto ocupando uma sala, um quarto no hospital, e aí isso, e aí eu ter tomado consciência disso pra mim já desde o início da minha gestação isso já me deixou muito forte assim, tanto por exemplo pra eu não querer que meu médico me acompanhasse porque eu sei que ele era muito nessa linha da cesárea.

E3: Então a preparação pro parto é bem importante eu acho, que a gente ter consciência, a gente ter esse empoderamento de que a gente pode estar a frente, tomar as decisões, ele é muito importante, mas entender que imprevistos acontecem e a gente não vai ta no controle

de tudo também é importante, e eu levei um tempo pra isso. E eu considero assim, diante de tudo que eu te falei, eu considero que a pandemia teve um efeito negativo assim na minha gravidez porque eu tava bem, tava saudável, tava sob controle, tava bem monitorada e aí assim essa mudança, esse estresse muito grande, toda essa mudança de cenário e essa insegurança que me causou eu acho que potencializaram o meu mal estar da pressão, que resultou no que resultou [...] Eu acho que a preparação pro parto ela tem que ser pro melhor, não pro que eu desejo, não pra um formato né, ela tem que ser pro melhor.

E5: Ai meu Deus eu acho que foi fundamental, acho que precisa muito se preparar, precisa muito muito mesmo, fisicamente essa questão da fisio pélvica foi fundamental pra mim, eu sempre achei assim que eu era uma pessoa que eu tinha uma boa relação com meu corpo de me conhecer e saber sinais assim né, mas a fisio pélvica faz muita diferença, muita diferença, na hora das contrações, na hora do relaxamento do períneo, como fazer a força, posicionamento durante o parto, as posições melhores pro bebê passar no estreito superior no estreito inferior da pelve, foi muito muito muito importante.

E5: E a parte mais psicológica assim de, eu usei até um aplicativo pra meditação né durante a gestação que chama "gentle birth" tipo uma hipnoterapia que usa pra hipnose mesmo e que são afirmações positivas e muita conversa com a minha família sobre o que que eu queira do parto, o que eu imaginava, com meu esposo, com minha mãe, né, que eu queria tentar muito o parto normal até quando desse, então foi um acordo mesmo que a gente fez, equipe, eu, meu marido, então foi bem importante essa preparação de planejar mesmo, de planejar como que ia ser essa indução, até quando ia aguentar, como é que ia ser se precisasse de analgesia, todos os passos assim, as possibilidades dentro do trabalho de parto, então isso me deixou muito tranquila na hora.

E6: A questão do parto assim, como o meu primeiro foi uma cesárea também e eu hoje eu percebo que não foi necessária, foi uma cesária muito induzida pelos médicos “porque chegou a idade, já tá na ta hora, não tem porque ficar mais aí, vamo tirar e tal” então foi muito assim nesse sentido, porque esse segundo a gente procurou se preparar melhor, eu me preparei fazendo curso, a gente procurou fazer o curso online e buscar todo tipo de orientação pra que pudesse ser um parto normal né, era o meu desejo assim, era o meu desejo desde que eu soube que eu tava grávida eu falei assim “não dessa vez eu gostaria de ter um parto normal” e foi um processo de aprendizado assim, eu acompanhei muitas lives, participei de grupo de gestantes da UFSC e de outro também, de outro grupo, e procurei orientações a gente fez um curso online como eu já te disse sobre o parto normal, todo processo, o que poderia acontecer e o que não poderia, como lidar com as situações.

E6: Eu achei muito importante sabe, assim é psicologicamente a gente se viu numa situação totalmente nova né, durante a gestação e eu acho que o fato da gente ter se preparado e ter tido esse contato com pessoas que tavam na mesma situação, ter tido contato com as enfermeiras, com as psicólogas do grupo que nos deram uma acalmada, vai dar tudo certo, enfiam a gente sentiu que tava mais seguro assim, apesar de toda situação que não foi fácil. Eu acho que o desfecho realmente era uma possibilidade sabe, era uma possibilidade que tava

clara pra gente desde o início, sabe, poderia ser uma cesárea, então assim, desde sempre todo mundo foi, passou essa informação, não é que você se prepara tanto que vai dar no que você quer, não é assim a gente não sabe como vai ser, então assim apesar do desfecho ter sido uma cesárea eu acho que é isso que me tranquiliza assim, que me deixa feliz sabe.

E13: É eu acho que faltou esmiuçar melhor o momento ali do parto, porque até exercícios que a gente poderia fazer para facilitar o parto né, então depois eu sabia porque alguém falou "nossa mas tem um movimento ali dos pés que tu vai abrir ou fechar o teu quadril pra facilitar a passagem do bebê e tal" e eu não sabia fazer isso, então acho que essas coisa são muito importantes, eu acho que facilita, porque hoje tem doulas, tem maternidades que realmente tem muita experiência que acompanha e tudo mais mas ali comigo não tinha e nem podia ter, era proibido ter qualquer outro tipo de acompanhante então assim, eu tava no escuro, eu tava pensando minha natureza vai me ajudar, mas cara se alguém te der uma orientação ali facilita, é menos sofrimento pra mãe, pro bebê, acho que as coisas vão ser mais rápidas talvez, não sei, e assim, preparação eu digo desde os movimentos, até a roupa assim sabe, porque a gente podia ficar de top mas tem mãe que fica sem porque elas, na maternidade elas não te orientam com relação a isso, elas só falam que tu tem que tirar a roupa e deu, e eu queria ficar de top porque ela nasceu no inverno e era muito frio, então eu queria sei lá, se eu posso ter um jaleco, eu não sabia de nada disso, também ninguém orientava e foi uma semana anterior a neve que teve então, imagina, claro que ali no calor a gente vai trabalhando e não percebe tanto mas são coisas assim mínimas que a gente não sabe assim, pode tomar água ou não pode tomar água? pode comer ou não pode comer? eu não sabia, eu não sabia de nada disso, claro eles me davam uma água de vez em quando mas eu também não tinha essa instrução, será que é bom ou ruim? Esses detalhes são bem válidos, acho que bem importantes, pra gente ter segurança. E outra coisa, o médico realmente não vai fazer o parto, ele vai assistir e tal, mas deixar a mãe tranquila, ele vai ali ele vai te orientar, nesse sentido, elas falam mas eu acho que na aula pode esmiuçar um pouco mais o momento do parto, acho que seria bem válido

E16: Eu acho fundamental, porque assim é uma experiência talvez única na vida de uma mulher e para além digamos das questões técnicas, sei lá, dessas questões médicas, eu acho que você precisa entender a transformação pela qual você vai passar, que não é só física, que é muito importante, não só emocionalmente, financeiramente, ninguém fala pra você que ter um filho, claro todo mundo fala que vai mudar a vida mas isso é retórico, ninguém fala como de certo sua vida vai mudar e especificamente com essa questão do parto eu acho fundamental que a mulher saiba como que isso vai acontecer, quais as opções que ela tem, que ela saiba que ela é sim capaz de parir, eu acho que isso está mudando, mas é como se uma coisa que acontece historicamente tivesse que ficar nas mãos dos especialistas que no caso são os médicos mas, nós mulheres estamos totalmente preparadas para isso. [...] eu acho que o processo deveria se encaminhar pro contrário, deixa a mulher que ela experimente todo o processo e caso seja necessário então que venha a intervenção médica, e não que seja a intervenção médica que decida como que a mulher vai parir, eu acho que é isso, por isso eu acho que a informação é fundamental, porque se você se sabe essas coisas você vai lá na maternidade e pode se posicionar e pode falar diretamente com as pessoas "olha, eu quero

isso, eu desejo meu parto de tal forma". […] Mas é só informação que faz a diferença, se você não sabe disso você fica nas mãos do pessoal que faz um ótimo trabalho, também não estou dizendo que eles tivessem me tratado mal ou coisa para nada, mas você fica muito a mercê do que acontece lá o tempo todo, coisas que são procedimentos corriqueiros para eles, mas para gente é um momento que muda a vida né, então é isso.

E17: Eu acho que informação, informação é tudo né, eu acho que empodera muito, o fato de você saber o que tá sentindo, o que vai sentir, o que vai vir, é muito, alivia muito, alivia muito saber o que vai vir. [...] acho que é fundamental a gente ter conhecimento do nosso corpo, do que vai acontecer, porque acho que ninguém te ensina isso isso não aparece em nenhum lugar e muitas vezes a consulta pré-natal é muito rápida, você tem muita dúvida de muita coisa, não da pra aprofundar cada coisa né. [...] ai não da pra tirar todas as dúvidas né, dai a gente fica meio que tentando perguntar muita coisa e não conseguindo aprofundar em nada, então o bom é que a gente consiga entender muita coisa, do que vai acontecer e do que esperar, isso ajuda também no emocional né, porque a gente fica nervosa mais ainda numa pandemia, então ajuda pra gente se acalmar, pra gente se preparar psicologicamente também, porque eu acho que informação é isso, ajuda né, empodera né, e fica melhor quando a gente sabe o que vai acontecer, porque já é desconhecido né, já ta fora do nosso controle né então ter o mínimo de conhecimento ajuda com certeza. Por isso eu recomendo e fiz tudo quanto é curso que tinha por aí. [...] Acho que isso que fez a diferença porque eu sabia que o que os médicos estavam me falando não era verdade sabe, não ia ter, até porque além de eu ter estudado tudo isso tinha também o "pacape" da minha doula que tava falando comigo o tempo inteiro, então eu sabia que não ia, então é.

E20: Olha, é fundamental porque eu senti muito pouco as contrações mas tive momentos que já sentia ela e eu acho que a mulher tem que acreditar assim como na amamentação que tem que ter esse apoio de alguém pra lembrar ela que no momento de maior desespero ela vai conseguir sabe, que é uma onda eu acho que quando falam que é uma onda é exatamente isso é uma onda que ela vem intensamente mas ela passa mas, tu saber isso e tu ter esse preparo antes é fundamental porque eu fico pensando quem simplesmente só chega e acontece eu acho que deve ser muito difícil sabe, não é instintivo, tem gente que diz que é instintivo mas eu acredito que não, precisa de um trabalho assim.

E21: Eu queria que fosse um parto normal né, com mínimo de intervenção possível, se eu quisesse que tivesse analgesia e tudo mais mas eu tinha diabetes gestacional né então muita gente já ficava me alarmando pra isso mas mesmo assim eu tava bem decidida. O próprio sistema ele trabalha muito de forma que a gente caia numa cesárea porque eu fazia acompanhamento no alto risco e inclusive numa das últimas consultas a médica falou "Ah, a partir de agora se não controlar tua glicemia daí a gente vai marcar pra cesárea" dai ela falou assim já e eu falei "Não, mas eu não quero cesárea, eu vou induzir" dai ela "Ah tá, é é pode ser" tipo assim já, ai eu "É é, eu vou querer induzir", "Ah então tá tá bom, a gente fala cesárea porque a maioria quer cesárea" em vez dela fazer o contrário né, não, se eu não fosse com a cabeça preparada né, isso deveria ser um movimento contrário delas falar "Ah, você

poderia ta induzindo se não der certo você vai pra cesárea" mas não fosse partir de mim ela já queria agendar.

E21: É importante a gente ter conhecimento, nem que seja ou não profissional, estar informada ali bastante mas infelizmente isso não garante também nada, infelizmente tu vai tá a mercê dos profissionais que vão te atender mas o bom é pra tu ter uma ideia, como eu falei, eles já estavam indicando a cesárea né, então com tantas semanas a gente vai interromper, se eu fosse uma mãe qualquer eu ia falar "Não, então tá, o médico falou que tem que fazer cesárea né, por causa da minha diabete" e eu não ia saber "Ah, mas dá pra induzir né".

E22: Olha, eu acho que tu tem que tá preparado porque tudo pode acontecer sabe, tu que saber que um parto natural é um parto natural mas que em algum momento podem ter intervenções e tu tem que saber o que pode acontecer em termos de intervenções que são realmente necessárias que não são questões de violência obstétrica sabe, se tu tá preparado, se tu sabe realmente aqui que tu podes esperar do profissional, porque eu sei que eu sou uma privilegiada eu levei a minha obstetra mas que a maioria das pessoas vai pra um plantão de um hospital público então assim essa questão da violência obstétrica é bem grave e ainda bem recorrente agente sabe, e acho que se preparar pro parto é se preparar emocionalmente muito porque tem gente que romantiza demais né, também saber que se for necessária uma cesariana a cesariana ela ta ai pra salvar vidas, então tu também não é menos mãe porque não conseguiu fazer um parto normal, acho que é tudo assim na verdade, a primeira etapa pro teu maternar é tu tá preparada pro teu parto.

3. CONSULTAS PRÉ-NATAL

E1: Era tudo me deixando na porta e eu entrava via tudo né, consultava tudo e ele também não conseguiu se fortalecer sabe. Ele também tinha muito medo do parto e ele não conseguiu se… também ter a segurança da médica, sabe, porque a gente não teve esse ao vivo ali né, até o final ali.

E2: Pra mim foi tudo tranquilo assim o pré-natal, claro que quando entrou na pandemia eles deram uma parada né, mas aí depois voltaram aos atendimento e aí foi tudo bom, tudo bem assim. Não tinha problemas no pré-natal.

E5: Algumas consultas, por exemplo, ali no fim de 2019 eu fiz algumas consultas presenciais né, com o obstetra e depois quando começou a pandemia as consultas passaram pra online, então eu fui la mais no final mesmo, quando eu tinha que fazer o exame de BCF, o exame físico mesmo. Mas a parte de orientação e exames tudo ela me passava online.

E11: Eu acho que acabou sendo mais difícil, pelo fato de estar na pandemia, porque já começa por consulta, consulta já era mais difícil, tinha lugares que não atendia ainda bem no comecinho da pandemia foi quando eu descobri minha gravidez então eu queria logo fazer a ultrassom, queria logo começar o pré-natal e ninguém tava atendendo, o posto não atendia,

dizia que não tinha nem previsão pra isso, foi bem difícil assim.sabe e daí nas consultas também não podia ter acompanhante, meu marido queria participar mas não tinha como.

E15: Ela foi planejada né, o que não foi planejado foi o Covid né, a pandemia, então eu tive certeza mesmo foi, fiz o exame em janeiro e aí logo em março a gente entrou em pandemia né aí fechou tudo, todo mundo pra casa, e eu tava bem no começo da gestação então eu peguei ali o começo de pré-natal em janeiro, fevereiro, e março já não teve, poucas, não teve consulta, fechou e a gente retornou acho que ali finalzinho ali de abril, no meio de abril. [...] Eu já chegava nas consultas, como a pessoa que pesquisa, vai atrás, sabe o que que vai ter, o que que ta preparando, eu ja chegava pedindo exame tal, quero esse exame, então foi bom por isso, tu não chega esperando que o médico te falei ou a enfermeira te fale que te atenda, tu já chega questionando, perguntando qual o próximo passo, o que que precisa o que não precisa, esses exames, tal, tal, quero fazer, então eu acho que nem só em tempos de pandemia mas em qualquer gravidez sem ser pandemia eu acho que as mães deveriam fazer isso né. [...] Informação é tudo, porque eu já chegava no posto ja com as coisas que eu queria fazer né, pedia ultrassom, pedia exame de isso ou daquilo, mesmo não sendo normal do SUS, não ser padrão, isso eles falavam pra mim "Isso não é padrão mas se tu quer" eu falava "Não, eu quero fazer", inclusive exame da clamídia gonorreia né, que coisas que eles não pedem a não ser que eles tenham desconfiança, e aí foi isso também me atrapalhou um pouquinho a pandemia mas foi, num contexto geral foi tranquilo.

E16: Quando veio a pandemia precisamente eu tinha marcado uma consulta no dia que mudou tudo assim, eu fui lá acho que era 16 de março por aí e a enfermeira falou "não, desculpa mas não vamos conseguir te atender, a gente esta se organizando pela questão da pandemia, vamos tentar fazer os atendimentos pela telemedicina, computador enfim" e aí parou, só que nesse mesmo momento eu estava com muitas infecções urinárias, eu tive várias na gestação e me encaminharam para ginecologista, obstetra, não me lembro bem, e aí eu fiquei em alto risco, então meus atendimentos mudaram daqui do Campeche pro Continente pra, não me lembro como se chama, a UPA do Continente eu acho que chama mas passaram a ser muito mais espaçadas assim, as consultas normais eram uma por mês e depois eu passei a ter consultas a cada dois meses, muito muito espaçadas e isso foi um pouco complicado. [...] acabou que meu pré-natal foi muito, enfim, eu quase não tive consultas no final por exemplo a primeira ecografia que é pra ser com 12, 13 semanas eu fiz com quase 22 semanas, imagina, ou seja, muito muito avançada a gestação e até perigoso né porque se, ainda bem que o bebê tava tudo certo com ele e tudo mais mas, como também financeiramente não tinha como arcar o pré-natal no privado e tal, então ficou meio assim complicado para ter os atendimentos em tempo, foi mais ou menos isso. [...] No último mês eu consegui voltar nos atendimentos aqui no posto porque eu me queixei que os atendimentos estavam sendo muito espaçados, que não estava tendo um atendimento bom, considerando que estava próxima do parto, então voltei aqui no posto aí eu tive que fazer novos exames, enfim e comecei a marcar consultas semanais

E17: Chegou uma época que eu tive que fazer consulta online também, que também é bem diferente, eu não tinha nem balança em casa pra me pesar por exemplo, eu não tinha nem

maquininha de pressão pra medir a minha pressão, então eu tinha que comprar ou ir na farmácia pra pagar pra me pesar ou pra que tomarem minha pressão, tomarem, medirem não sei, por que eu não tinha como fazer isso pra acompanhar sabe. [...] Meu marido não conseguiu ficar por dentro de nada, nem das consultas, nem das ultrassons, tudo isto foi eu sozinha, eu acho que foram só duas ou três que foram online e o resto já foi tudo presencial, até porque acho que no finalzinho da gestação não tem como muito fazer online né, tem coisas que tem que ser feitas bem presencialmente né.

E19: Eu fui atendida ali no posto da trindade, meu pré-natal foi todo ali e na verdade tipo eu acho que teve uma ou duas que não foi todo mês assim que a enfermeira e a médica meio que optaram, deixaram aberto pra gente, se a gente sentisse a necessidade né de ir ia né, e eu acho que a gente teve uma ou duas que pulou mas no geral continuei indo certinho tipo todas as consultas, fiz todos os exames, ali não alterou muito essa questão assim tipo, eles meio que deram uma prioridade pras gestantes, não sei se aconteceu isso em todos os postos né mas ali teve até uma época que eles criaram, separaram assim, fizeram uma parte isolada assim só pra gestante mas o pré-natal foi ótimo assim nesse sentido.

E22: Algumas consultas foram online também e aí é muito diferente, não tem como tu comparar, tu não faz um exame físico né tu te pesa e passa pra ela, passa as informações mas ela não ta te avaliando então assim, foi um período bem ruím assim, um período que eu fiquei bem insegura sabe, bem insegura mesmo. Mas logo depois ela resolveu assumir o risco que ela viu que não tinha condições de cuidar de uma gestante à distância, muito complicado, logo depois a gente voltou então a gente ficava vindo de Rancho Queimado só pras consultas mesmo.

4. PLANO DE PARTO

E2: Fiz, mas na hora eu nem apresentei, eu nem cheguei a apresentar, ficou dentro da minha carteirinha assim, eu não cheguei a entregar em mãos pra médica, porque foi tudo tão assim rápido e a gente fica tão em função assim, sei lá.

E3: Fiz junto com a parteira, mas nem precisou né, porque ai tudo mudou porque ai quem ia me acompanhar na indução do trabalho de parto era a [nome da profissional] que dai ja tinha muito a linha do que eu queria, inclusive ela até brincou comigo ela assim “O pessoal aqui da Santa Helena sabe que quando eu vou pra cesárea é só porque realmente precisa, porque não adianta a mãe dizer que quer a cesárea porque eu não vou pra cesárea só se a mãe quer, não vou botar uma mãe numa cirurgia e depois ter que cuidar de um bebê toda cortada porque ela quer, eu só vou se realmente for necessário né”, então assim não… eu acho até que tava acho que a gente levou impresso naquele dia e ficou na mala porque não precisou.

E5: Não, cheguei a fazer um plano formal, porque como te falei que e fiz o pré-natal particular então já sabia mais ou menos a linha de atuação da obstetra, da equipe dela e da enfermeira que participou do parto, então já tinha combinado préviamente como ia ser, então não foi nada escrito assim.

E6: Sim, chegamos a fazer, desde o primeiro a gente já tinha feito, porque no primeiro a gente já tinha feito, porque no primeiro a gente pesquisava, procurava saber, mas não foi uma coisa mais tão profunda como a gente fez dessa vez sabe, porque a gente fez o curso, que a gente acompanhou com as gestantes com o grupo de gestantes, então primeiro a gente, eu pesquisei muito mas não foi realmente uma coisa tão dedicada, eu acho que se eu tivesse me dedicado mais eu teria sabido lidar mais com as pressões né de entrar realmente, de fazer a cesárea por motivo de tempo sabe então eu acho que o segundo a gente tava mais preparado e sabia mais o que dizer no plano de parto sabe. Até teve no grupo de gestantes orientação de como fazer o plano de parto, teve muita informação muito importante, sabe. [...] A gente já tinha consciência de muita coisa, foi muito importante, muito bom, muito importante pra gente, tranquilizou bastante.

E10: Cheguei, cheguei a fazer mas não deu tempo nem de imprimir (risos), não deu mas a gente já tinha assim tudo muito claro né, do que a gente queria e do que a gente não queria, é uma coisa muito louca porque assim, porque quando chega na hora também a gente se abre pra todas as opções porque a gente começa a vivenciar aquilo de uma forma que ok se a gente se prepara, eu me preparei né, pra um parto natural mas ok se tiver que vir uma anestesia, se tiver que vir uma interferência enfim pra acelerar o parto enfim, a gente se abre pra isso também, porque a gente sabe o que ta acontecendo com o corpo né. Mas enfim, não precisou, a gente não precisou de ocitocina, não precisou induzir, foi bem tranquilo, não precisou anestesia também, mas a gente sabia desses recursos né, por conta do preparo do plano né, a gente se informou disso.

E16: Ali no curso com o pessoal do HU foi sugerido que a gente fizesse um plano, inclusive passaram um material, um modelo de plano de parto e a gente fez, a gente imprimiu e naquele plano a gente colocou o que queria, depois eu fui o mais natural possível, então eu pedi ali especificamente para só intervir em caso de necessidade, evitar remédio, eu não queria tomar epidural por exemplo e eu passei por muita dor, ou seja, só naquele momento você sabe quanta dor você é capaz de suportar. E eu senti, passei por muita dor, muita, muita mesmo mas assim, até o final eu abri a mão dessa opção de aliviar a dor com medicamento, cesárea era uma coisa que tava bem específica ali no plano de parto, eu não queria cesárea porque assim, enfim, me parece uma cirurgia muito complexa a recuperação é mais demorada, enfim, e eu tava, como a gente é estrangeiro aqui nossa rede de apoio é mínima e numa pandemia, imagina, muito mais, e eu tive que pedir pra minha mãe vir da Colômbia e ela veio em um voo humanitário porque naquele momento não tinha voo comercial então sabe, muitas coisas envolvidas que estavam ali naquele plano de parto, com a ideia de tentar fazer o mais leve possível aquele processo todo. E eu tava lá, meu plano de parto tava lá com meus documentos, com todas as minhas coisas nas pasta que a gente sempre carrega na gestação mas assim, quando eu fui internada eu pensei em esperar para passar pelo quarto e tal e meio que eu fui esquecendo do plano sabe, porque eu vi, é como se eu tivesse me entregado aos procedimentos que estavam predispostos e ninguém também me perguntou se eu tinha plano de parto, ninguém me perguntou “Você quer isso, você quer aquilo”, eu fui

muito bem tratada o tempo todo, mas isso foi uma coisa que ninguém me perguntou se eu queria isso, se eu queria aquilo, se a indução eu preferia vaginal, não sei qual outra forma existe mas se tinha outra opção, enfim essas coisas então o plano ficou lá na pasta o tempo todo, eu voltei para casa com o plano na pasta, guardado, e simplesmente não sei, depois eu pensava nossa mas porque eu permiti, eu devia ter manifestado isso né, mas como a gente fica, como eu te falei, presa no medo, você não sabe o que vai acontecer os médicos às vezes eles ficam em cantos assim falando baixinho e você não sabe se estão falando coisa boa ou coisa ruim, então como que você fica condicionado ao que eles escolhem pra você.

E20: Eu cheguei a fazer plano de parto também, então eu fiz uma pasta com as músicas pra colocar no parto e eles deixaram eu colocar, a luz não tava excessiva também e foi algo bem tranquilo assim sabe, apesar de saber da cesárea foi planejado, foi com plano de parto e foi bem aceito, não tive dificuldades com eles.

E21: Um cuidado que eu tive no meu plano de parto, de colocar que se meu bebê precisasse de complemento que eles primeiro falassem comigo, isso era pra ser uma decisão em conjunto equipe e comigo, que eu não autorizava que dessem leite pra ele que não fosse o meu. [...] o que eu tinha muito medo que eu coloquei no plano de parto era a questão da episio mas o ruim é isso que não depende da gente, depende muito do profissional, dai eu falava muito pro meu marido “Não deixa me cortarem, não deixa me cortarem” dai ele ficava ansioso também com isso ele falava “Meu mas o que que eu vou fazer, vou saber se tem que cortar, não tem que cortar”.

E22: A gente fez um plano de parto, de parto e de pós-parto, eu fiz um curso, um outro curso falando sobre um plano de pós-parto que achei super legal também, que todo mundo só fala do parto né mas o perrengue mesmo vem depois e aí a gente fez, eu acho que no grupo eles disponibilizaram também um modelinho né e na verdade eu peguei vários modelos e adaptei pro nosso assim sabe e aí junto com a nossa obstetra também, a gente também tinha uma doula então foi uma coisa meio que multidisciplinar assim sabe, todo mundo ajudou um pouco e a gente fez, foi bem legal assim, dentro do possível do nosso parto que foi um caos assim, bem distante do que a gente imaginava, foi bem humanizado sabe, dentro daquilo que a gente esperava e tinha colocado no plano de parto.

5. INFLUÊNCIA DA PANDEMIA:

E1: A gente foi pego de surpresa pela pandemia. Em março, eu tinha acabado de começar a fisioterapia pélvica e tava começando a me preparar para o parto normal mas, sempre com a clareza assim, eu tinha medo da dor, das possíveis intervenções que poderiam acontecer, mas eu queria muito esperar o tempo do meu bebê né?! Entrar em trabalho de parto, esperar um dia dela chegar… isso era muito claro pra mim, não importava como ela viesse, eu não fazia questão, não “tinha que ser parto normal”, não! E eu não queria optar pela [cesárea] eletiva, agendada assim sabe. Eu queria entrar em trabalho de parto, deixar a coisa acontecer, eu sempre falava isso “eu quero deixar acontecer”. E aí em março a gente entrou na pandemia e foi muito difícil, tanto pra terminar a preparação para o parto, porque eu não consegui

terminar a fisioterapia porque a gente [perda de conexão]. E aí, não e aí assim, os médicos toda equipe de apoio tava começando a aprender a lidar com as gestantes na pandemia, agora ja ta mais ou menos todo mundo acostumado, os médicos já atendem online e o pessoal ja ta vacinado.

E1: Eu assisti muita aula assim, procurava no youtube… mas ao mesmo tempo eu fugia um pouco, porque eu tinha medo do parto normal então eu não conseguia ver video do parto normal, eu tinha muito medo assim, de não ter o apoio ali sabe, talvez se fosse em outro contexto e eu pudesse procurar mais ajuda, né, pudesse conversar com a doula, eu lembro que na época eu fazia terapia e a minha psicóloga falava "olha, quem sabe tu não marca uma consulta com a doula, uma conversa" e eu assim "não, eu não posso sair de casa", eu tinha muito medo de sair de casa, porque realmente né. E então, tudo dificultou pra eu procurar outros meios pra me preparar sabe.

E3: Quando veio a pandemia em março né, mais assim final de março acho que foi meado de março aí eu já logo parei de trabalhar [...] eu parei de fazer exercício, na época eu fazia pilates e tava fazendo pilates e yoga, ai parei de fazer os exercícios e parei de ter vida social e aí entrou num modo de preocupação intensa né, porque hoje, não que hoje esteja menos perigoso mas hoje a gente tem mais informação né, hoje a gente tem mais conhecimento do que que dá pra fazer, até onde dá pra ir, quais são os verdadeiros riscos né e naquela época não se tinha, então eu lembro assim que eu fiquei sem ver a minha família até depois da [nome da filha] nascer. Ai eu e o meu maridos fomos pra, a gente mora num apartamento aí nós fomos pra nossa casa de praia da minha família e aí ficamos os dois lá sozinhos com meu cachorro e aí assim, uma solidão profunda né, final de gravidez assim, barriguda queria ver todo mundo e as coisas não foram possíveis né, e assim por sorte e eu agradeço, eu já tinha feito o chá de fraldas e já tinha feito books de gestantes então essas duas lembranças assim eu tive na hora certa porque muita gente não teve né, e aí eu lembro assim, até que minha mãe passou lá na praia um dia pra me entregar um bolo uma coisa assim e aí eu toquei na mão dela assim de longe, e logo já corri pra lavar a mão e acho que ela da mesma forma porque a gente não sabia né, eu não sabia se ela era doente, eu não sabia se, como é que aquilo ia funcionar pra mim e aí eu até me emociono de lembrar (choro), e aí foi difícil!

E4: Já desde fazer mala, tipo, ah pega máscara, pega um monte de máscara, pega álcool em gel, pega álcool líquido e não é comum. E tipo, ah mas lá eles devem oferecer, mas a gente não tem certeza, então leva né, aí lá assim, mesmo que a gente saiba que os hospitais tomam todos os cuidados, tudo que eles davam pra gente de alimentação tudo a gente passava álcool em tudo, a gente tinha já esse cuidado de ficar pensando em tudo isso né, desses cuidados sanitários assim.

E5: E também pra mim uma lado, eu não posso negar que um lado bom da pandemia foi que eu fiquei afastada do trabalho então, por ser gestante né, como eu trabalho em hospital, então foi um período que eu fiquei bem dedicada assim pra mim, fiquei em casa, convivendo mais com a minha família, então foi um período relativamente bom assim, né, dentro desse

contexto louco que a gente viveu foi um período que eu pude me concentrar mais no meu corpo e perceber as coisa e me dedicar só pra isso, então pra mim foi bom.

E6: [...] foi um momento muito, acho que foi um momento muito único mesmo, porque é você praticamente ter uma gestação e guardar só pra si, é essa sensação que eu tenho, que eu guardei só pra mim e pro meu marido e pro meu filho, assim sabe, parece que ninguém, de repente apareceu um bebê na família assim, na família de meu pai minha mãe, meus irmãos, de repente surgiu um bebê pra eles, porque embora tivesse chamada de video, foto, a convivência não foi possível sabe, a convivência foi muito muito restrita, foi uma vez que eu fui na casa da minha mãe assim, e a gente ficou muito pouco tempo e eu tava de barrigão, então assim, ela não teve esse vivência de me acompanhar, assim também com a minha sogra e de repente surgiu um bebê pra eles, foi bem diferente assim.

E7: Com 31 semanas, mesmo com todo meu cuidado, eu tive covid, foi um susto grande. Tive o acolhimento e ajuda da enfermeira e da psicóloga, do grupo de gestantes, que foram essenciais neste período. Fiz meu isolamento de 14 dias e apesar do desconforto, tosse, e cansaço físico e mental elevados, após esse período estava tudo bem. Porém, às 34 semanas minha bebe resolveu nascer. E acredito que o covid tenha sido determinante pra isso.

E7: O fato da gravidez, por mais que você se prepare e que já tenha ouvido muito de outras experiências, é bem complexo, pois você sabe que a mudança vai acontecer e qualquer mudança tem seu lado "assustador". A tensão já existia, com a pandemia, tentei encarar de forma positiva, consegui trabalhar em home office , consegui acompanhar o grupo de gestantes, que sem a pandemia não teria conseguido. Porém, o contato com a família ficou mais distante e o acompanhamento médico tb, tinha receio de sair e acho que fui um pouco prejudicada pela pandemia nesse sentido.

E8: Isso foi muito traumático porque além de todo cansaço e de toda idealização que eu tive, de toda preparação que eu tive, tanto no grupo de gestantes né, que a gente continuou conversando, quanto na questão da minha preparação de antes de tudo que eu fiz, tinha o medo dessa questão da pandemia, a minha doula não pode entrar tinha toda essa questão de medo e eu não conseguia ficar muito tempo de máscara né, tava tudo começando então a gente tava com muito medo.

E11: Eu acho que pelo fato da gente ter se isolado também dificultou um pouco, porque a é um turbilhão de emoções na gravidez né e tu tá ali meio sem ninguém, tu ta totalmente fora da tua rotina, nossa passa um monte de coisa pela cabeça, e eu fiquei muito pra baixo no começo da gravidez e eu acho que isso influenciou muito. Até porque eu tinha uma visão diferente de quando eu tivesse grávida, de coisas que eu queria fazer de sabe, de estar na minha rotina de barrigão sabe, eu dançava por exemplo então eu queria ta fazendo dança com barrigão e não aconteceu nada disso sabe. Eu acho que nessa parte assim foi mais complicado, sabe.

E13: Eu tinha muito desejo de fazer o parto normal, de correr tudo bem e me preparar e fazer, tentar fazer hidroginástica e eu tentar fazer vários exercícios pra eu estar super preparada, e quando veio a pandemia o pânico de acontecer qualquer coisa comigo, com meu bebê era superior a tudo, então assim, a minha preparação foi "não, vou esperar acontecer de acordo com o que a natureza me proporcionar e vou me cuidar o máximo, não vou arriscar" eu pensei em não arriscar de fato, não saia de casa, não encontrava ninguém nada, eu ia até o médico e voltava, tava trabalhando em home office ainda estou minha empresa me proporcionou isso, mas vi a minha família só um núcleo muito pequeno e outras pessoas da minha família eu avisa não posso encontrar ninguém, não posso ver ninguém porque eu não posso correr risco, então foi bem limitada a minha preparação, com muito medo, confesso pra ti o medo era gigante, e acho que todo mundo tinha muito medo no início né, claro que agora a gente vê que a situação já tá melhorando e tudo mais mas não tinha vacina, não tinha nada.

E14: No início da gravidez começou a pandemia então isso gerou muita ansiedade né de quando veio o lockdown eu tava completando três meses, cerca de 12 semanas, então não tem como responder essa pergunta sem falar do período que a gente viveu como pandemia né, porque hoje quando a gente fala da pandemia parece uma coisa mais tranquila, mas quando chegou foi um susto muito grande mesmo, no primeiro momento assim foi muito impactante todo mundo trancado em casa e a gente com muitas incertezas.

E14: Teve os dois lados, teve o lado do medo, da incerteza da ansiedade, de uma frustração muito grande porque a gente engravida a gente quer passear com a barriga na rua, quer tirar foto, quer ta com as pessoas que a gente gosta e daí eu fiquei meses sem poder ver os meus pais e os meus irmãos, a gestação inteira eu não vi uma amiga, eu não compartilhei com ninguém sabe, então teve esse lado, que eu não pude sair pra fazer foto, enfim, teve esse lado todo das restrições. Mas por outro lado também privilegiou eu tá em casa focada quase que cem por cento na gravidez porque por mais que eu trabalhasse no remoto porque eu sou professora e ano passado até eu ganhar as meninas eu tinha 40 horas semanais na prefeitura de Florianópolis mas com o ensino remoto isso se flexibilizou bastante e ficamos eu e meu esposo só em casa cuidando, curtindo essa gravidez, que se não fosse por essa pandemia ia ta cada um no seu emprego, só se encontrando e vivenciando muito pouco né, então o foco seria outro, então assim a gente conseguiu se cuidar, se curtir e aproveitar, eu conseguia todos os dias pegar sol na barriga, cantar pras minhas filhas e eram coisas que se fosse a rotina normal né trabalhando até próximo ao parto eu não conseguiria, então acho que tem esses dois lados da pandemia.

E14: A minha preparação foi muito privilegiada por justamente também pelo período da pandemia porque daí eu pude trabalhar em casa, eu acho que o trabalho remoto privilegiou isso, o fato de eu tá em casa eu pude me dedicar então a procurar lives, palestras, cursos e nisso eu cheguei no curso do HU né, foi um dos cursos que eu fiz, o curso mais sistematizado assim que eu fiz mas eu fiz outros cursos eu assisti várias palestras e eu acredito que esse período de preparação tenha sido um lado bom digamos assim, desse período da pandemia né, de ta em casa e ter mais tempo pra olhar pra minha gestação, diferente do que se fosse o

momento que eu não tivesse a pandemia e eu tivesse trabalhando, eu acho que eu não conseguiria ter me dedicado tanto a esse lado da espera das meninas.

E16: Eu comecei então diante da situação da pandemia que eu estava impossibilitada de sair pra procurar atendimento, e acima de tudo eu tinha muito medo e tava muito recente a questão do vírus, a gente não sabia, tinha medo de pegar, tinha medo de passar pro bebê, tinha medo de morrer, então eu comecei a procurar ferramentas aqui em casa para tentar levar uma gestação o mais saudável possível aí comecei a fazer yoga, assim de tutoriais no Youtube que uma cunhada passou pra mim, ela é médica mas eu sou da Colômbia e ela mora lá na Colômbia e ela me passava tutoriais e eu comecei a fazer.

E17: A gente queria se preparar muito só que por conta da pandemia tudo mudou né, tudo que a gente queria fazer até o curso da UFSC, que eu tava super empolgada que ia ser presencial e tudo mudou de um dia pro outro, a gente teve que se adaptar às novas condições, eu tinha doula e com ela eu tava me preparando né, com a minha doula, porque assim eu sou mãe de primeira viagem, então eu não conhecia nada não sabia nada, e até as consultas com a doula eram online, então a gente teve que se adaptar a essa nova modalidade de fazer tudo online em um contexto de muita incerteza com o primeiro filho que ja traz muita incerteza, muita coisa é nova. No início foi meio difícil né, porque cada vez a gente tinha esperança que ia mudar, que ia melhorar e não aconteceu, então a gente teve que se virar com o que a gente tinha nesse momento né.

E18: Aí veio a pandemia, mais uma coisa que me deixou um pouco mais né, a gente sempre fica sensível na gravidez, com a pandemia não foi diferente e eu fiquei também mais sensível mas não tanto quanto eu fiquei quando eu descobri que eu tava grávida, eu sabia que ia ter modificações em várias coisas e apesar de tudo tava participando do grupo e tinha várias coisas que poderiam ajudar na parte do emocional para relaxar que era a meditação, fazer um pouco de yoga, então tinha várias outras coisas que eu fazia pra evitar um pouco essa tensão e esse estresse.

E18: Quando fechou tudo que ai não pode sair de casa pra nada, só uma pessoa, tira a roupa na volta, toma banho, isso traz uma ansiedade, uma sensação esquisita, eu lembro que me trouxe uma sensação de me sentir presa, só que eu fui trabalhando o meu psicológico. [...] Eu usei muito meditação no youtube, yoga, fiz muita yoga do youtube, tinha também eu lembro que passaram no grupo uma cartilha de exercícios também pra fazer em casa, que eu acho que também eram exercícios de yoga mas eram exercícios pra próximo do parto, ou desde o começo, acho que desde o começo da gravidez ja podia fazer assim, lá depois dos quatro meses é claro, eu fiz esses exercícios e esses exercícios me acalmavam muito, principalmente quando eu fazia assim meditação com a yoga, então eu fazia os dois, fazia a meditação e depois alguns minutinhos de exercícios pra yoga.

E21: A pandemia afetou muito isso né porque até na escolha de profissional né, porque dai tu fica meio assim de ficar peregrinando por ai né, já mal queria sair pra ver um médico quem dirá ficar olhando vários né, pegando uma segunda opinião e tudo mais. E o próprio preparo

perineal tudo né, porque como eu te falei eu fiz um pouco mas dai lá pelas tantas já interrompia porque eu ficava com medo, eu acho que eu não fui em quatro sessão de, bem pouco assim, de fisio, porque me dava medo de sair de casa assim. [...] Teve outras coisas que eu queria ter feito na gestação tipo a hidro né, que o pessoal fazia hidroterapia, essa aula ainda que eu fiz do PPM que eu te falei eu fiz porque era né seco, dava pra usar máscara dai na hidro o pessoal não usava máscara, tinha um pouco de receio mas eu sempre tive expectativa de fazer tudo isso quando ficasse grávida né mas dai não, dai a drenagem que eu ia lá fazer toda semana eu pedi pra elas irem na minha casa fazer porque eu fiquei com medo de sair e mesmo assim PPM eu fiz muito pouco porque eu ficava acompanhando na tv como é que tava o mapa de santa catarina dai eu ficava com medo, uma hora eu cancelava outra hora voltava, ficava assim, toda hora muito ansiosa.

E23: Foi na primeira alta assim da pandemia né, quando o [nome do filho] nasceu foi a primeira vez que deu assim 90% de ocupação nos leitos assim né, então como era tudo muito novo, muito recente, da um susto bem grande assim né, eu me lembro bem direitinho do dia que eu parei de trabalhar, foi no dia 17 de março de 2020 né, que o governador do Estado pediu pra que tudo fechasse porque foi aquele "BUM" assim, chegou no Brasil, tinha chegado em Florianópolis né, e aí eu me lembro que assim a parte grávida da história, eu me lembro que eu ja tava achando o máximo exibir a minha barriga, ir pro trabalho exibir, eu nunca fui muito de sair a gente nunca saiu muito assim né mas eu achava o máximo assim já usar roupa justinha pra mostrar minha barriguinha que já tava aparecendo né, daí no dia 17 disseram assim "Ah, então todo mundo vai trabalhar em casa" nossa aquilo caiu como um peso, aí que eu fui ter a noção do negócio, que eu ia ter que ficar em casa talvez até o [nome do filho] nascer, a gente não sabia né, foi assim sabe.

6. INCERTEZAS EM RELAÇÃO AO PARTO

E1: Especialmente ali no primeiro ano de março do ano passado até o final do ano de dezembro, foi muito difícil, de não ter a rede de apoio, de tu não pode compartilhar a gestação, de tu não ter a segurança de talvez procurar o profissional mais adequado, até assim coisa pequena, do medo na maternidade sabe, de não sentir segurança, sabe [...], porque ainda era tudo muito incerto assim, desconhecido, acho que com o tempo as coisas vão melhorar assim, mas pra gente que pegou primeiro momento ali foi muito cruel, foi muito difícil.

E1: Até hoje eu fico me, vendo às vezes né, no instagram algumas mães ali video ali e relatos assim, e eu fico pensando se era mesmo o momento de eu ter ido, porque o que que eu fiz, eu fiquei controlando no aplicativo né, controlava ali a contração quando iniciava e quando parava, quando iniciava e quando parava, aí chegou o momento que o aplicativo começou a dizer "prepare-se, vá até a maternidade" [...] teve uma hora que deu uma contração muito forte ali uma dor muito forte, daí eu comecei me ajeitar e fui, não cheguei a ir pra chuveiro e nada, porque eu fiquei com medo de dai acontecer alguma coisa e eu não ter ninguém, não ter suporte, só eu e ele, completamente inexperientes ali né, a pandemia atrapalhando, ai a gente foi pra maternidade, foi o único suporte que eu tive, foi assim.

E6: Eu senti que tava evoluindo, eu senti que as contrações tavam vindo com mais frequência, e senti que tava chegando a hora sabe, até pedi pra tomar um banho pra relaxar um pouco porque eu ja tava ficando um pouco assim, um pouco aflita de, eu não tinha passado pelo parto normal ainda, não sabia como era, e pra mim imaginar era tudo muito, a não sei, meio tenso, como será, será que vai dar tudo certo, será que eu vou conseguir então, tava tudo isso passando pela minha cabeça sabe. Embora eu tivesse uma tranquilidade em saber que as pessoas estavam ali auxiliando eu tinha esse receio assim de saber como seria sabe, e era novo né.

E18: Não tive uma boa experiência no segundo parto, foi um pouco difícil foi um parto normal mas foi um parto difícil e eu sempre tive muito medo do final né, da parte do parto, sempre foi uma coisa que me deixou ansiosa. [...] Então quando foi na parte do parto era uma parte que eu tinha muito medo mas, com o tempo, com as conversas, aquilo foi diminuindo meu medo, porque eu vi que eu poderia ter um parto realmente humanizado aqui, então foi uma experiência boa, eu não tive uma experiência de parto ruim, foi uma experiência muito boa.

7. FRUSTRAÇÃO PELA IDEALIZAÇÃO DO PARTO

E3: A minha preparação pro parto também ela foi, logo depois que a [nome da filha] nasceu eu vivi uma frustração, porque eu me preparei muito pra um parto natural, muito, e aí foram muitos meses, todos esse processo que eu te falei, e aí não ter vivido aquilo num primeiro momento eu me senti assim, pô eu não fui capaz, pra mim é triste te dizer que eu sou mãe e eu não sei nem qual é a dor de um trabalho de parto, porque eu não passei né, tive uma contraçãozinha ou outra e tal mas, a maternidade ela já vem assim avassaladora desde o primeiro momento então assim, é a natureza que decide e a minha natureza naquele momento foi de que a minha filha não tava bem e tinha que nascer, e por sorte, por Deus, pela ciência, por tudo, eu tive oportunidade de que ela nascesse em boas mãos, no momento certo.

E8: Eles começaram a me encaminhar pra cesárea. Eu não aguentava mais, mas eu não conseguia acreditar. Justo eu, que sempre foi pró parto natural sem intervenções, que sempre acreditou que a cesárea era uma cirurgia que servia só para complicações no parto... eu, agora sozinha, estava indo. Eu me sentia vazia, eu queria gritar, eu queria que a dor passasse, eu queria sumir. Depois de ter muita dificuldade de ficar parada pra receber a anestesia epidural, eu me deitei ali, na sala de cirurgia, sem esperança de nada. Eu não acreditava mais nem que teria uma filha no final de tudo isso, por mais que ela estava sendo monitorada o tempo todo.

8. PRESENÇA DO ACOMPANHANTE

E1: E aí a questão do emocional pegou muito ali na primeira fase, ali em março abril que a gente não sabia como é que ia acontecer eu não sabia se eu ia continuar tendo atendimento da obstetra, quando foi chegando perto as maternidades fechando, sem poder entrar acompanhante, acho que em questão de dias assim teve um monte de rumor assim “Ah, lá na Itália as mães tão parindo sozinhas, na Holanda…” eu me lembro assim, bem disso, dos

outros ter que ganhar o bebê sozinho, aquilo me apavorava assim, eu entrei numa crise ansiedade bem forte nessa fase e aí eu ganhei ela em junho, então foi um processo, até acostumar, ali em março, abril, às clínicas juntarem, acostumar fazer o ultrassom do bebê sem olhando, sem o pai acompanhando, sem poder visitar minha família. Todo mundo deve relatar isso assim, dessa parte do apoio assim, foi muito difícil, de não poder mostrar, compartilhar a gestação, de como estava sendo legal… Então, nessa parte o meu emocional ficou muito abalado, até ganhar ela.

E2: Tava aquela história de um dia podia entrar acompanhante outro dia não podia, um dia podia e o outro não podia e uma das meninas do grupo tinha dado entrada no dia assim sabe, durante o dia, e não podia entrar acompanhante, então eu tava bem nervosa assim por causa disso. **[...]** Aí o nosso medo era esse de na hora do parto ele não poder me acompanhar né, porque nós os dois estávamos assim estudando bastante pra entender tudo assim, sabe, e aí eu falava pra ele “sozinha eu tenho medo de ficar sozinha né, eu preciso de alguém pra me ajudar, me acompanhar" e ele tava junto comigo ele sabia tudo que que eu queria, e era isso que tinha medo de ele não poder entrar na hora porque a gente tava ouvindo ali alguns relatos de alguns casos que o acompanhante não pode entrar, mas aí pôde.

E3: E aí começou uma onda de não permitirem os maridos a entrar nos partos, começou muito forte no HU e aí eu soube até de uma conhecida que não pode o marido entrar, só entrou, eu acho que só entrou quando o bebê nasceu. Tinha algumas outras até, acho que algumas do grupo de mães que durante todo o trabalho de parto que um determinado momento ela já tinha ido pro hospital e ele só entrou quando começou praticamente o expulsivo assim, quando já tava saindo, então todo aquele momento ela ficou sozinha então vai assim, afetando a gente né, e aí imagina que eu tinha toda uma preparação pra ser domiciliar, pra ser na minha casa, na minha família com meu marido assim e aí daqui a pouco eu não ia ter nem meu marido, não sabia nem onde que eu ia poder fazer, quem que ia ta comigo, aquilo me deixou bem bem mal.

E4: Na verdade o ponto principal dessa questão assim pra mim durante a gestação, tinha o medo né primeiro do vírus em si, tanto pra mim quanto pro bebê, e a segunda coisa que foi assim, que era o baque maior de tudo isso era a questão dos acompanhantes, que foi o que mais me deixava nervosa se eu ia ficar sozinha, se ia poder ficar o meu marido, ai as vezes, “Ah, o hospital tal não ta deixando nem o marido, aí só pode o marido mas não pode doula, aí minha família não vai poder visitar”, daí minha mãe ficou bastante chocada que ela não poderia entrar na maternidade pra me acompanhar, depois pra ver o bebê, depois aquela coisa de quem entra na primeira vez não sai mais e tal… era mais essa questão assim que me preocupava dos acompanhantes né, e o medo assim do vírus de tu pegar ou acabar o bebê pegando, ai ainda muitas notícias assim aleatórias sem comprovação científica, e aí tu ficava meio nervosa assim, né. Mas foram essas duas questões que pegaram mais pra mim durante o parto mesmo né. Aí depois tem outras questões assim de isolamento né, ali no começo de não pode sair, não poder o bebê, a família não pode conhecer, essas coisas que foram bem diferentes né do que é o normal.

E4: Nesse sentido de acompanhamento né, nem nas consultas e ne no ultrassom eu podia ter acompanhante. Essa questão do ultrassom também foi outro impacto, porque tem toda aquela coisa né, de ouvir coraçãozinho, de ver o bebê e assim, meu marido não pode ver nenhum ultrassom, minha mãe também queria ver algum só depois viam as imagens assim, mas eles não conseguiam me acompanhar, as clínicas não deixavam entrar, nem na recepção assim. Até depois, quando eu tava fazendo aqueles ultimos ultrassons com barrigão, que teoricamente eu não poderia nem ta dirigindo, dai meu pai me levava assim e não podia nem subir assim, não podia acompanhamento mesmo.

E5: É eu lembro que algumas semanas, um mês antes dele nascer foi bem na época que as maternidades proibiram a entrada dos acompanhantes, algumas maternidades aqui de Florianópolis, e isso me deu uma ansiedade assim muito grande, porque a gente vem assim de uma vida inteira planejando um parto, pensando que vai ter acompanhante, que vai ter o esposo, a minha mãe eu queria muito que tivesse comigo, então isso foi nossa, um choque muito muito grande de ter a possibilidade de ter que parir sozinha né, com uma equipe, apesar de eu ter parido com uma equipe que eu já conhecia presencialmente, mas de estar num ambiente desconhecido e ainda sem a presença do acompanhante, que eu acho que foi uma das partes fundamentais pra mim.

E6: O meu marido pode ficar até a hora, [...] durante o parto ele pode ficar, ele que ficou com o bebê logo que o bebê nasceu e aí eu tive que fazer os procedimentos de costurar e tudo, e ele ficou com o bebê o tempo todo, acompanhou todo processo, acompanhou todo procedimento com o bebê assim e isso foi muito bom, coisa que eu não tive no primeiro filho assim, que foi numa outra maternidade que a gente ficou separado, a gente só viu ele depois de algumas horas, eu fui sedada, e lá no HU eu não tive essa questão, não fui sedada, então fiquei o tempo todo acordada, pude acompanhar o tento que eu podia a presença do bebê, a presença do meu marido, então foi muito melhor, muito melhor.

E7: O acompanhante eu consegui ter mas era uma preocupação pois estava mudando o protocolo diariamente.

E10: Acompanhou, isso, mas uma semana antes eu acho, a gente teve aquele decreto que não podia entrar doula né, a doula enfim, um terceiro acompanhante, segundo acompanhante, mas meu marido pode me acompanhar sim, ele ainda pode me acompanhar, algumas do grupo acho que ainda tiveram que ir sozinha, não sei, não sei como é que foi, mas ele pode ir nesse começo, fazia muito pouco tempo que a gente já tava em lockdown então ele pode me acompanhar durante esse período.

E11: Então eu achei a questão do parto eu achei muito difícil, o que tranquilizou foi que pelo menos ele pode estar comigo do lado na hora do parto, então ajudou porque tranquilizava porque tinha massagem enfim, tudo isso né. Aí foi, em partes foi horrível, em partes foi maravilhoso, depois que ele nasceu eu esqueci de tudo na mesma hora.

E13: Eu pedia pra ele fazer pressão né na minha bacia porque isso ajuda bastante realmente a aliviar as dores da contração, porque por mais que seja rápido mas aquele minuto ele é muito intenso, ele fazia esse movimento me ajudava bastante, e ele conseguia me ajudar super bem.

E17: Bom, eu assim, o que eu tinha me preparado né, minha expectativa é que fosse um parto normal, só que eu também sabia que grande parte da situação não tava no meu controle né, a gente quer, a gente se prepara mas se na hora o bebê decidir vir de outro jeito ele vai vir de outro jeito né, minha única assim preocupação era eu ter que parir sozinha, acho que isso me gerava um medo sabe, porque eu senti que aquele suporte, aquele apoio do meu marido ia ser fundamental né pra conseguir aquele parto que eu queria, normal, sem ter a força e bom, graças a Deus deu certo ele conseguiu entrar mas eu não sei se teria sido do mesmo jeito se eu tivesse que parir sozinha porque chegou um momento que naqueles grupos de mães, até no whatsapp, até no grupo da minha doula, não estavam permitindo nem doula, nem acompanhante, nem ninguém, então tipo começou a crescer esse caos, aquela sensação de "putz, e agora a gente faz o que né”.

E18: Como já tava nessa parte bem restrita meu marido não pode subir comigo, ele teve que ficar lá embaixo me esperando enquanto eu fazia essas caminhadas, ele só pôde subir pra acompanhar esse processo do parto quando eu já fui pro quarto, ai sim que ele pode subir pra acompanhar, aí ele se trocou e ele subiu, fizemos o processo deixamos as coisas e fomos pro quarto. [...] eu fiquei feliz porque pelo menos ele tava do meu lado [..] nosso terceiro filho foi o único filho que ele pode assistir o parto, os outros dois ele não teve essa oportunidade.

E22: A gente tava com bastante dúvida porque até então nem o parceiro poderia entrar em alguns lugares eu acho que no HU não podia né, tinham algumas mães no grupo que estavam bem desesperadas, eu se tivesse sozinha tinha enlouquecido. [...] A minha doula entrou mas é porque no fim como é particular tu acaba conseguindo né, assim, ela é uma doula que sempre presta serviços pra eles então assim acaba que já tem um protocolo que a maternidade tava seguindo que tinha algumas pessoas, alguns profissionais que estavam cadastrados, que tinham feito um curso de segurança sabe teve tudo isso, eu lembro que ela teve que fazer um curso especial mas ela pode entrar então eu tava com a minha equipe.

E23: Eu cheguei lá já tive que entrar sozinha sabe, o [nome do companheiro] não pode entrar comigo né, ficou no carro e aí eu fui pra sala de preparo ali fazer aquele exame cardiotoco , fiquei sozinha né, daí eu tive que tomar um antibiótico antes porque eu tinha aquela bactéria sabe e aí de bolsa rompida, fazendo antibiótico na veia, numa sexta pra sábado de madrugada sabe, sozinha, pode de cansada né e aí só depois que eu fiz o cardiotoco, que eu fiz o antibiótico, isso foi sei lá acho que pela uma ou duas horas da manhã que aí eu fui pro quarto e aí o [nome do companheiro] pôde ir comigo, ele ficou todo esse tempo lá fora esperando sabe, por causa dessas restrições que tinha.

E23: Uma coisa que eu senti muito muito muito é não ter a presença do [nome do companheiro] nas consultas sabe, porque dai assim ó ficou uma coisa assim ó, eu sabia de tudo, eu tava aprendendo as coisas e ele não sabe esse afastamento assim, parece que isso era

até como assim o filho é só meu, sabe? Eu sabia de tudo e ele não participava de nada né e aí eu fazia consulta depois marcava o exame de ultrassom e ia fazer na outra clínica, dai ele também não podia entrar e querendo ou não assim ó, sendo que assim como eu estava na consulta e no exame tirando as minhas dúvidas, tirando os meus questionamentos, interagindo sabe com o médico, conversando e vendo, aquela coisa assim né eu acho que isso vai crescendo o conhecimento da gente, crescendo o interesse pela gestação, pela fase sabe, aquela coisa que te motiva né e o [nome do companheiro] não pode participar de nada disso, eu acho que ele foi em uma ou duas consultas só antes de março ali e aí então eu percebi assim que parece que criou um certo distanciamento dele assim sabe, em relação ao processo todo assim, dai essa foi uma dificuldade assim que eu percebi sabe.[...] ele me ajudou bastante na maternidade mas depois eu sentia ele assim bem despreparado, um pouco é questão de maturidade também da pessoa né, ele não teve maturidade suficiente e por acho não ter tido toda essa interação durante a gestação, eu acho que isso comprometeu um pouco sabe o nascimento da paternidade dele.

9. O PAPEL DAS DOULAS/PARTEIRAS

E1: Com a pandemia a maternidade não tava cadastrando novas doulas e fsioterapeutas pélvicas, então só aquelas que tavam cadastradas, eles não tavam pegando novas, então eu não consegui o acompanhamento dela, eu entrei em trabalho de parto, tive contração, peguei o médico plantonista assim que o atendimento foi bem ruim e me mandou de volta pra casa, mas eu tava com muita dor e nenhuma dilatação. Até que no meio da dor assim, em contato com ela, eu liguei pro meu médico e a gente fez a cesária, intraparto ali né.

E3: A ajuda das parteiras também foi bem legal porque aí tipo, de fazer o exercício, tipo ó "Pegue esse caminho aqui", por exemplo, quando eu descobri que eu tive pressão alta a [nome da profissional] me falou assim "Come proteína a cada duas horas", isso é passado entre parteiras, porque quando tu tem pré-eclâmpsia se eu não me engano tu começa a perder proteína na urina, e aí quando tu repõe aquilo a cada duas horas tu pode minimizar esse efeito, então isso foi bem legal.

E12: Ela e mais uma enfermeira obstetra me acompanharam, a gente fazia uma vez por semana um pré-natal com elas, elas ensinaram o meu marido a escutar o batimento cardíaco da [nome da filha], eu também fazia acompanhamento pelo SUS, mas elas nos deram muita autonomia pra entender todo processo que tava acontecendo, elas nos ensinaram a sentir a posição da [nome da filha] na minha barriga, o meu marido marcava os batimentos cardíacos e ela ia contando, foi um processo maravilhoso, ela ensinou ele a entender como ela tava posicionada e fazer o desenho na barriga, do meu bebê, que ficou lindo, e depois a gente teve só um encontro presencial, elas tinham sumamente cuidado por causa da pandemia, então foi tudo muito, muito, muito cuidado mesmo e a gente se encontrou toda semana até o dia do parto. E o dia do parto foi aqui em casa.

E17: Em tanta incerteza eu acho que ela era o meu porto seguro sabe porque eu escolhi ganhar com um obstetra de plantão porque não estava em condição de pagar minha obstetra

que ela me acompanhou eu todo meu pré-natal, mas como eles cobravam a parte o parto e tudo né, a gente não tava em condições de pagar então em tudo isso a doula virou meu porto seguro sabe, que essa pessoa conhecida né, que aliás eu não me arrependo, ela foi muito boa.

E19: Eu conversava muito com a parteira de tipo, das coisas que eu pensava, tipo ela colocava algumas sugestões assim tipo desmistificava algumas coisas [...] ela falou que acho que não criar esse ideal também que tu vai se apaixonar nesse primeiro momento também foi muito importante assim pra mim né, de pensar o parto, eu pensava na questão das posições tipo, uma das coisas que eu pensava assim, mas como eu te falei eu não tinha um ideal e aí eu tinha dúvida assim de qual que era a melhor posição, tipo mais confortável coisas nesse sentido e aí parteira falava "ai, isso na verdade vais é muito do momento, é muito instintivo assim, é como tu vai se sentir confortável, tem mulheres que parem em pé, tem mulheres que é de cócoras, deitada, enfim, cada uma vai de um jeito assim".

E19: Ressignificar o parto que eu fui parida assim, a minha mãe participou eu parindo né, e ela também tava muito insegura assim e aí elas, as parteiras conversaram com ela assim, do que que é essas angústias, quais os medos, quais os receios e tipo se colocaram a disposição pra ela também, de tá ali ali tá todo mundo meio que junto pra se dar apoio assim, eu acho que se colocar nessa escuta ativa também é importante assim tipo, acho que escutar a mulher é uma coisa essencial nesse processo de construção do parto, às vezes vão ter angústias que a gente nem imagina né.

10. A ESCOLHA DO LOCAL DE PARTO

E1: Não podia entrar, na época tava fechado os espaços ali na sala de parto, tava fechado para visita. Eu tinha plano de saúde então a opção que eu tinha era ali ou a Santa Helena e ali no Ilha eu ia ficar no apartamento sem custo nenhum né, por isso que eu optei pelo Ilha né. E aí, eu preferia pagar se eu precisasse, ah se fosse pra pagar alguma coisa eu preferia pagar o médico assim, do que pagar uma equipe pro HU… porque tava tudo muito incerto sabe, muito.

E2: Já tinha escolhido o HU por ser um hospital humanizado, referência em parto humanizado no Estado.

E3: Eu me planejei fisicamente, emocionalmente pra ter um parto bem natural assim, cheguei a inclusive cogitar o parto domiciliar, tava em acompanhamento com minha médica obstetra mas também com uma parteira que tava me acompanhando por fora né.

E4: As meninas do curso elas fizeram um tour pelo HU, pela maternidade do HU assim filmando, foi muito legal esse, eu não conhecia a maternidade do HU assim, foi bem legal esse vídeo pra conhecer como é que era, toda estrutura, bem legal.

E6: Dessa vez a gente não pode fisicamente né, mas eles mandaram fotos, contaram um pouco do que que acontecia em cada lugar, isso ajudou a visualizar assim, imaginar como seria, a e foi bom assim, no final teve, acho que a informação foi suficiente.

E8: Uma longa jornada nos fez escolher o parto domiciliar como a opção mais segura de todas, inclusive por causa da pandemia mundial. [...] O que eu tinha mente eu sempre quis um parto domiciliar e eu tinha uma amiga da minha doula, ela era médica, ela é médica e a gente tinha planejado de fazer o parto aqui em casa na água né, que era o que eu desejava fazer então esse foi o meu planejamento, um pouco antes da, ai logo em seguida teve a questão da pandemia que foi uma coisa bem complicada mas eu fiquei tranquila porque eu já tinha planejado de fazer o parto em casa então não tinha essa coisa do hospital.

E12: Fazia pouco tempo que eu morava aqui em floripa então eu não conhecia muito mas eu fui pesquisando assim a respeito do parto humanizado, eu tava muito afim de parir e eu tava muito afim de parir com esse lugar de respeito, de carinho, de cuidado, surgiu pela minha cabeça fazer um parto em casa então eu visitei o grupo “humanascer” mas vinha o medo de a ter que ir pra neonatologia e sair de casa correndo, recém-parida com o bebê… aí decidimos fazer no HU, ai eu não tinha plano de saúde, eu comecei a fazer tudo pelo SUS e achei ótimo e referências do HU cada dia eram melhores. [...] Daí começou tudo o negócio da pandemia, isso que era em março, eu ia ia parir em maio e eu fiquei apavorada, e daí eu olhei pro meu companheiro e falei “Eu não vou parir no hospital”, porque naquela época já estava se falando que não podia ter acompanhante, foi bem no começo, então assim, foi um pânico, eu lembro que ele saia pro mercado parecia um extraterrestre assim (risos), era tipo um negócio muito louco, aí eu falei assim “não, eu sozinha, parindo no hospital no meio de uma pandemia, não” e daí a gente decidiu ter um parto em casa. [...] Então a gente fez um parto domiciliar planejado, que nesse momento foi a melhor decisão que eu consegui tomar e realmente foi maravilhoso, foi um trabalho muito, muito, muito, com muita dedicação, com muito carinho, com muito respeito, com muito profissionalismo.

E15: Essa pandemia uma coisa que me deixou bem ansiosa ali no final foi que a gente tava no próprio grupo com informações que os esposos não tavam podendo entrar, só na hora do, e depois ficariam sozinhas e daí foi um dos motivos que eu escolhi a maternidade lá de Biguaçu que tava podendo ficar, ficava um dia se fosse normal ai o que eu optei também, foi uma das coisas, então aquilo ali me deixou um pouco ansiosa em tempo de pandemia, imagina né primeiro filho tu tá sozinha, não é que tu ia ta sozinha ia ter ali a equipe mas depois o enfermeiro ia sair tu ia se virar né, isso me deixou muito ansiosa.

E18: A gente chegou a conhecer só pelas fotos e vídeo que foram apresentados no grupo, que não podia fazer visita por causa da pandemia e aí apresentaram as fotos no grupo pra gente poder conhecer né, que antes falaram que poderia fazer as visitas né com a gestantes só que por conta da pandemia não da, ai a gente viu nos video e fotos que foram apresentados.

E19: Acho que a preparação emocional foi o que mais, o que mais fez diferença pra mim nesses grupos né e o parto em si eu tinha com a questão da pandemia né, nesse processo do

parto, eu pensava em ter em hospital e coisa e tal quando eu pensava na possibilidade de, na época que eu pensava na possibilidade de engravidar, ai me vi grávida na pandemia, pensar em hospital "e se acontece alguma coisa comigo? e se eu pego covid? e se ela pega covid?" tipo aquela, porque tava muito incerto né naquela época, a gente não sabia nada sobre nada e aí pensar no parto domiciliar foi meio tipo "tá, é mais seguro tá em casa né" tipo nesse sentido.

11. A VIA DE PARTO

E1: Tava tudo muito incerto, sabe, muito. Tudo isso somou pra eu tomar a decisão pra eu optar pela cesárea, o medo assim de daí eu voltaria pra casa mas também ficaria eu e meu marido ali sozinho, a gente não sabia… sabe? Tinha um medo de chamar a fisio em casa, nossa foi horrível, horrível, foi uma experiência péssima.

E2: Eu tive apendicite, eu tive que fazer a cirurgia [...] e o pós-cirúrgico foi bem dolorido pra mim, foi bem complicado. E eu ja queria fazer, já pensava em fazer parto normal né [...] como o pós-cirúrgico foi bem complicado pra mim foi mais uma questão pra eu dar preferência, assim tentar né, fazer o possível pra que o meu parto fosse natural também, porque eu ficava pensando assim, eu não vou conseguir passar por outra cirurgia né, de cesária, e com o bebê recém-nascido, foi bem complicado mesmo pra mim.

E14: Eu tenho muito medo de sentir dor né, então uma das coisas que eu pensava era como é que eu ia fazer um parto de gêmeas, porque eu sou da área da educação e penso muito essas questões do desenvolvimento humano e é importante que seja um parto normal, assim é, pra saúde, pro bem-estar, então a gente também tem uma romantização em relação a isso né então eu pensava muito que eu queria ter, sempre quis né, a quando engravidar ter um parto normal e aí com gêmeos já, opa, tem que pensar diferente, vamos ver o que que vai acontecer, então ja me fez pensar um pouco diferente e eu sentia muito medo do parto assim, eu queria me preparar, eu também sou da educação, sou da área da pesquisa eu tinha essa coisa de querer me preparar ao máximo e depois se eu contar o que aconteceu, não teria preparo nenhum que resolvesse a situação, porque às vezes as coisas acontecem então todo preparo não é suficiente nessas horas.

E19: Parecia que passar por isso seria, apesar de ter as dores, sei que não seria fácil a questão das contrações tudo mas, eu gostaria muito de ter passado, só que ao mesmo tempo o fato de eu saber que já ia ser cesárea me tranquiliza do que nos casos de às vezes ter tido todo trabalho de parto, todo preparo e de repente ter que necessariamente ir pra uma cesárea sabe, eu acho que ali talvez eu ficaria mais frustrada, como eu sabia que seria uma cesárea eu fui mais preparada então eu sentia a contração, a bolsa rompeu e isso pra mim foi fundamental sabe, pelo menos teve todo esse processo mesmo.

12. GRUPO DE GESTANTES/ REDE DE APOIO

E1: Então, o grupo foi bem importante pra gente manter essa troca de experiências assim. Como assim, a gente não tava podendo, tipo eu via minha mãe de março até junho eu via minha mãe pouquíssimas vezes, porque dai a gente já escutava muito relato de gestante internada… Não via ninguém assim, ao ar livre mesmo, acho que tu lembra como que foi ano passado até junho assim, a gente não saia de casa mesmo assim, ninguém saia. Então eu não tinha essa troca com mulheres mais velhas, com tias, com primas, claro a internet ajuda mas é diferente. Então ali a gente criou um laço muito forte, muito assim, a gente conversa coisas que às vezes a gente não conversa com marido, com pai nem com a mãe, porque cada uma sabe que a outra tá passando pelo mesmo processo, então foi bem importante. O grupo de gestantes eu digo que foi fundamental nesse contexto de pandemia, não sei se fora a pandemia, nos grupos anteriores se as mães mantinham essa troca forte assim sabe, se conseguiu manter um laço tão duradouro como a gente ta tendo assim, cada etapa a gente consegue, primeiro foi o parto, depois foi o puerpério, amamentação, depois introdução alimentar… agora assim as bebês tão indo pra creche, as doenças da creche, então tá sendo bem legal pra gente trocar essas ideias assim.

E2: O meu objetivo de participar desse encontro né, uma que todo mundo já me dizia que era o melhor grupo de gestantes [...] e aí como eu queria também informação, queria fazer parto normal, foi um dos motivos que me levaram assim né, pra mim foi ótimo assim porque durante os encontros elas explicaram tudo né, tudo tudo tudo, tiraram todas as dúvidas assim, foram super incansáveis mesmo né. Como a gente não pode fazer a visita elas mostraram a sala de parto, pré-parto e pós-parto, então assim eu sabia tudo tudo tudo né, porque eu queria muito que meu parto fosse natural e aí meu parto foi realmente, foi bem natural, evoluiu super rápido assim.

E3: Olha, vou ser bem honesta assim, porque como o nosso grupo ele não foi o presencial e eu já entrei no grupo de gestantes eu acho que eu ja tava com 34 semanas que foi ali em março né, a primeira reunião foi em março, então eu acho que assim, na minha preparação para o parto não foi tanto, mas no meu pós-parto e até hoje assim, ele mudou o meu maternar assim, eu acho que eu fui uma mãe melhor graças ao grupo. E assim, todas as gestantes que estão próximas a mim, todo mundo eu recomendo que faça, até as que moram em outras cidades assim eu falo "ah ve se vai continuar no formato online, porque se tu puder participar participa", porque a rede de apoio ali aquelas mulheres que, tanto as profissionais que nos auxiliam que trocam ideia ali, que nos dão conselhos e tal, elas são muito, ai assim elas passam uma confiança, tu vê que sabem do que tão falando, que tem experiência, que tem "know-hall", que tem um olhar assim de empatia com a gente, quanto as mães que tão vivendo a mesma dor, ou que já passaram ou que vão passar por aquilo, aquela troca ali é muito boa, muito boa assim, foi maravilhoso, um dos presentes da minha gestação foi o grupo. Ai muito bom.

E4: Ai nossa, esse grupo foi assim, muito maravilhoso (risos)! Porque principalmente porque a [nome da filha] ela é a caçula do grupo então assim eu vi todos os relatos possíveis de parto ali no grupo, então assim, ali nas horas das contrações a questão da respiração eu tentava né, isso foi uma coisa que elas me passaram do grupo, tanto as colegas participantes quanto as

organizadoras ali né, pra relaxar o máximo possível, a questão da respiração, aí assim logo que lá nasceu né, o meu direito de ficar com ela, de logo ter contato com ela, de botar no peito, tudo isso assim, de várias questões ali que a gente tem direito assim né, ainda ali na sala de parto, de cesárea né pra garantir esses meus direitos.

E5: Eu lembro que por ser enfermeira eu já tinha algum conhecimento sobre parto, fisiologia do pato, mas o grupo foi fundamental pra eu ter mais conhecimento e poder trocar informações, experiências [...] E o grupo assim foi uma rede de apoio importante, não só nessa parte de conhecimento técnico mesmo, mas de apoio mesmo entre as mulheres, sabe, de saber tipo ai as outras também tinham azia, sabe, os sintomas, de poder compartilhar desses incômodos da gravidez com as outras mulheres, e ver que a gente é normal que às vezes assim, eu como passei pela primeira gestação é tudo muito novo né, a gente apesar de saber que é normal a gente quer assim se identificar com o outro saber que também tem alguém vivenciando aquilo que a gente tá passando, então eu acho que a minha preparação foi mais nesse sentido assim, a parte teórica né, que eu tinha nas consultas pré-natal e essa parte emocional assim com o grupo e é isso e com a minha família também.

E5: Ah, eu acho que foi fundamental, se eu não tivesse participado eu estaria bem mais perdida assim. Apesar de eu já, antes de eu entrar no grupo eu já tinha decido assim que ia parir numa clínica particular que eu ia continuar com o pré-natal que eu já tava fazendo mas o grupo foi muito legal, inclusive porque a gente tinha um grupo no Whatsapp depois, então ainda é uma rede de apoio muito muito legal, a gente criou amizade mesmo entre os participantes e me fortaleceu sabe, eu acho que o grupo me fortaleceu pra decisões, até pra compartilhar informações de como é que estão as maternidades e pandemia e sabe, vacina e tudo mais, todas as informações em relação aos bebês e pandemia e tal. Foi bem importante.

E5: Eu lembro muito das técnicas de alívio da dor que foi uma aula que a gente teve online já, que era sobre as massagens e meu esposo participou dessa aula também e ele se sentiu bem motivado a participar das massagens durante o trabalho de parto, até na gestação a gente treinou depois da aula, foi bem legal assim, acho que mais a parte dele foi bem importante assim, porque eu como tu disse eu já tinha esse conhecimento mais técnico assim né e pra ele participar de todos os encontros foi muito importante. [...] Ele não ia nem nos ultrassons, não ia nem nas consultas de ouvir BCF, era tudo por chamada de vídeo assim, então a parte do grupo pra ele eu acho que foi bem bem importante.

E5: Esses encontros online do grupo que a pandemia trouxe, eles possibilitaram que mais mulheres que não moram em Florianópolis, que não são daqui enfim, que não tem fácil acesso a universidade, participassem do grupo, então eu acho que isso é um ponto positivo de ser online e de estar sendo online, só pra deixar registrado, que talvez seja uma possibilidade daqui pra gente em disponibilizar esses encontros online.

E6: Ah sim, eu acho que foi isso, de dar uma tranquilidade pra gente, de saber, tinhas as informações, as últimas informações com relação o que tava acontecendo, não só com relação ao que tava ocorrendo com relação covid mas o que tava acontecendo na maternidade e isso

foi dando uma certa tranquilidade pra gente de saber que tinha, como é que tava sendo o procedimento com relação às mulhere que tavam tendo o parto naquele momento né, e isso foi, essa notícia sabe, essas notícias que tavam vindo, porque tudo era muito novo naquele momento né, a gente não sabia bem o que era esse vírus, o que podia ocorrer com a gente, principalmente com as gestantes, com o bebê e enfim, era tudo muito, então elas passavam pra gente, e sabendo o que tava acontecendo elas iam passando pra gente, isso nos tranquilizou bastante, sabe. E também outras mulheres né, compartilhar com outras gestantes aquele momento.

E7: O grupo ajudou muito pois pincelaram vários assuntos , pudemos trocar experiências e o que restava dúvida, tinha dicas de onde buscar mais. Pra mim foi essencial.

E8: Em relação ao grupo de gestantes, foi bem legal, [...] o grupo de whatsapp virou uma rede de apoio muito boa, a gente conversa e principalmente no começo, a gente conversa até hoje bastante assim, pra trocar ideias e falar sobre as coisas e tirar dúvidas e desabafar, fiz umas amizades muito boas ali, principalmente no começo quando a gente é mãe de primeira viagem a gente acha que tudo tem que ir pro hospital e essa era a parte mais difícil porque às vezes eu ficava pensando "será que realmente eu preciso ir pro hospital e não to indo e aí tem essa coisa da pandemia, eu posso ir lá e pegar covid" então era uma das coisas mais absurdas assim, mas do medo era de não ir no posto ou não ir no médico com medo da pandemia e achar que tava negligenciando algum cuidado que realmente precisasse né, um cuidado médico.

E9: O grupo foi mais uma fonte de conhecimento onde pude conhecer e tirar dúvidas sobre os momentos, os mecanismos... foi onde mais detalhadamente ouvi sobre todas as fases e pude fazer meu marido ouvir também para estar preparado.

E10: Do grupo de gestantes nossa, foi muito bom depois porque tava todo mundo no mesmo barco né e então teve uma troca, uma partilha muito grande, em relação ao parto foi assim que a gente se preparou, eu vi outros relatos também, outras partilhas.

E11: Ajudou pela questão do grupo de gestantes porque daí eles mandavam material, explicavam como fazer inúmeras coisas e ele sempre participava comigo então ele tava ciente de tudo. Tinha coisas que eu nem lembrava e ele vinha puxar tipo "ah não, lembra que foi falado isso" e daí essa parte ajudou muito assim, porque por exemplo massagem essas coisas eu não pesquisava, posição de segurar coisa aqui na barriga de lençol, toalha enfim, alguma coisa pra diminuir o peso, eu nunca tinha visto isso e foi a coisa que mandaram e ajudou muito sabe então, foi mais parte do grupo mesmo que veio isso.

E11: Nossa, eu não sei nem te dizer porque eu sou muito grata sabe, porque realmente ajudou muito, foi meu principal apoio, do geral. [...] Nossa, o grupo pra mim foi muito muito importante mesmo, eu inclusive até indico, todas as minhas amigas engravidaram, muitas eu não sei se acham que é besteira mas é que realmente pra mim foi muito bom, muito bom mesmo.

E12: O grupo pra mim de whatsapp que até hoje a gente se fala, minha filha ja ta com um ano e meio praticamente, meu Deus como passa rápido, mas é uma rede até hoje, hoje eu já tô um pouco cansada, mas assim é uma rede importante, pra tudo, pra tudo assim, não só pra coisas de bebês, às vezes a gente conversa sobre outras questões e é importante, porque o que mais faltou nessa questão da pandemia foi isso, não ter uma rede de apoio, assim como pais de primeira viagem e é muito difícil. [...] então a mãe precisa ser maternada nesse momento, essa é a palavra, a mãe precisa de alguém que materne ela pra ela poder maternar solidamente seu bebê, porque a gente tá quebrada mesmo, a gente tá renascendo, a gente teve um bebê junto assim , então eu acho que, eu por exemplo eu sou Argentina e nunca senti tanta vontade de estar perto da minha família assim, sabe de ter a minha mãe do lado que eu não tive, de minhas tias, todo esse mundo de mulheres assim, por isso o grupo foi tão importante, ter essas mulheres do lado, por que os homens é um outro lugar e a gente precisa dessa ressonância com outras mulheres que passaram por isso, e os homens aprenderem muito mais a cuidar e abrir mão dos próprios cuidados, abri um pouco mão por um tempo das coisas pra eles.

E13: Com relação às informações foi muito válido, muito, super válido assim, essa questão de controlar contrações, saber até que ponto eu poderia ir, no plano de parto eu não fiz plano de parto mas na minha cabeça eu sabia falar lá o que eu queria e o que eu não queria então assim. [..] Eu anotei, muitas das aulas eu fiz várias anotações né, dai recapitulei dias antes e assim foi super válido [...] As aulas são muito válidas, todo conteúdo super importante, pra mim foi ótimo.

E13: [...] Eu não fazia nenhuma preparação porque de novo, eu tinha medo por causa da pandemia, eu tinha medo de sair e aí na aula talvez elas pudessem ter acalmado a gente também, claro que é uma decisão muito pessoal. a pandemia também é muito nova, todo mundo tinha medo e tudo mais, mas pra gente decidir e pesar, será que não era importante sair e caminhar ao ar livre, não esbarrar com ninguém? né, que pra gestação era muito importante, que iria ajudar no trabalho de parto, essas orientações também são muito válidas, eu acho que seria muito importante ter, então pra preparação e tudo mais eu acho que faltou assim sabe, de novo é tudo novo pra gente e o óbvio precisa ser dito e às vezes a gente nossa tinha medo assim de sair, mesmo que hoje a gente sabe que não ao as livre com todo cuidado não teria problema, lá a gente tinha muito medo.

E13: A orientação foi fundamental sem dúvida, alguns pontos que eu tô citando pra ti que podem ser aprofundados mas no geral é maravilhoso eu achei super importante. [...] Vivência, experiência, então muito mais válido, uma pena que foi online né, a gente perde muito com certeza trocas e tudo mais, a gente acaba não tendo tempo pra perguntar acaba atrapalhando e ali no ao vivo a interação é muito mais rica, mas mesmo assim eu achei muito bom, muito válido, eu super recomendo assim, quem tá gestante tem que fazer, orientações básicas que a pessoa tem que ter sabe, a rede de apoio ela ajuda mas às vezes a rede de apoio ela tem conhecimentos muito ultrapassados e tudo mais e é bom a gente ouvir pessoas com experiências e vivências mais atuais também.

E14: O grupo pra mim assim [...] foi bem importante porque embora eu participasse desses outros eventos e rodas eu sentia que o curso do HU ele me ancorava mais, ele trazia mais conhecimento científico, mais conhecimento técnico, até por eu ser ali da Universidade então eu sempre me sentia muito segura naquele ambiente que eu tava lidando com pessoas que são da área, que pesquisam, desse vínculo da pesquisa, o ensino e a extensão, então eu me sentia segura nesse ambiente falando com pessoas que são professoras da Universidade ou bolsistas como vocês, vocês que estão ali na ponta no HU mas ao mesmo tempo pesquisam, estão atualizadas, então ali eu me sentia mais segura do que participando de outros eventos que eu participava né. E o acolhimento, [..] inclusive no privado assim várias vezes. [..] Então assim eu acho que o curso em si possibilitou esses conhecimentos que eu me sentia segura durante as aulas, eu tenho tudo anotado, eu acho que eu anotei umas 30 páginas do que era conversado ali e de vez em quando ainda consultava mas assim também esses contatos assim, tu saber que poderia contar com essas pessoas de certa forma como uma rede de apoio depois né, [...] o grupinho que se formou ali com as outras mães também que a gente, agora tá bem parado assim, mas eu lembro que nos primeiros seis meses depois do curso o grupo foi bem movimentado, tinha muita troca de informação, pessoal mandava foto dos bebês, depois vai dispersando acho que é normal, mas o início ali que é bem importante que a gente tá se sentindo sozinha e foi legal porque mesmo a gente não ter se encontrado pessoalmente né, ninguém se conhece pessoalmente mas o grupo ficou um grupo forte assim. O material disponibilizado também pelas facilitadoras do curso no outro grupo que tem de envio de material, esse material era muito bom, algumas coisas me ajudaram bastante, então pra mim assim eu recomendo pra todo mundo o curso do HU. Foi bem significativo, eu acho que, é porque é como eu falo, não tem como eu falar dessa gestação sem falar da pandemia sabe, a pandemia deixou a gente muito sozinha, muito isolada, então aquele momento, aquela quinta-feira a tarde do curso era meu Deus eu to com outras pessoas sabe e essas pessoas que tão compartilhando o mesmo momento que eu era, fico até emocionada, porque era um momento que tu conseguia ter um pouco de contato, acesso, sei lá alguma coisa que te tirava daquele momento de tanta incerteza que a gente tava tendo da pandemia, acalmava, trazia uma serenidade, trazia informação de qualidade, então foi uma âncora mesmo.

E14: Eu acho que o curso teve essas três questões de influência, a questão de vir da Universidade que eu por ser uma pessoa da Universidade valorizo muito assim, então nesse sentido era meu porto seguro, que eu sabia que tava recebendo informação de qualidade, de confiança, atualizada né, essa ponte da pesquisa, ensino e extensão, então o momento do curso acontecendo em si, esse momento dos contatos que o curso abre, possibilita e a forma que as facilitadoras, não sei se chama assim enfim que nem todas são professoras né, facilitadoras ali que ministram as aulas, a forma super acolhedora como elas se disponibilizaram a cuidar da gente, eu não sei as outras meninas mas eu me sentia cuidada, amparada por elas, tanto nos encontros quanto nesse pós encontro quando eu precisei eu realmente encontrei ajuda ali nas situações pontuais que eu precisei e o grupo de mães que se forma, as que continual ali ainda trocam bastante informação então tem esses três pontos assim que são pontos de destaque.

E15: Aquele material de massagem pélvica, outra que vocês mandaram agora, massagem pélvica, fisioterapia pélvica, aquilo ali meu Deus, aquilo ali acho que foi perfeito, no caso eu não tava conseguindo arranjar uma fisioterapia pélvica, fisioterapeuta pélvica, então eu mesma fui autodidata, pesquisa vídeo, ver vídeo e material mesmo tava bem claro.

E15: Na verdade, o grupo de gestantes me deu bastante apoio e informações tudo, o plano de parto eu já sabia que existia porém o que me encorajou a levar um plano de parto pra um hospital público foi o grupo, porque né, a gente tirou todas as dúvidas, perguntou se podia, tirou cada aula que tinha ali a gente esclarecia nossas dúvidas, então foi um grupo bem ativo, foi um grupo de troca de muita informação. [...] O grupo de gestantes, inclusive foi eu que fui atrás né do grupo de gestantes, deu todo um respaldo por trás, mesmo que tu pesquise, leia livro né, que eu tava lendo alguns livros sobre essa parte ali, depois eu te mando alguns livros que eu pesquisei fora a internet, o grupo em si são mais detalhados né, com certeza, era um grupo só pra isso, na consulta tu acaba só exames, coisas padrão como eles falam né, tira uma dúvida ou outra que tu possa perguntar, mas a própria enfermeira lá do posto que ela já chegava "Meu Deus se todas as gestantes fossem que nem tu, que pesquisasse que quer exame que quer tudo, seria uma maravilha" eu dizia a deveriam né, deveriam tá todo mundo num grupo de gestantes, eu até passei o número do grupo do HU dessa parte de grupo de grupo pra ela passar pra outras mães, que ela também não tinha.

E16: Quando começou o grupo foi legal, mas também né a gente fica meio desapontado porque não é o mesmo que se ter o contato direto com as pessoas, a questão da virtualidade também muito, às vezes é muito supérfluo assim, você tá ali tá na tela a interação é outra coisa então você acaba também perdendo coisa que talvez no presencial teria e a gente aproveitou, foi bem bacana o espaço ali com vocês, mas eu sempre senti falta de uma coisa mais, sei lá mais ao vivo. [..] A forma como o curso está estruturado era muito boa assim, porque em cada encontro era uma temática diferente, um material de apoio também, a gente lia tudo aqui em casa, porque claro eu ia ser mãe de primeira viagem então a gente tá com todos os medos em cima, então líamos os materiais e sobre a questão do parto especificamente eu sentia que estava super preparada assim com as informações que tinha, a gente já tinha decidido que o bebê ia nascer lá no HU e também por toda essa questão de ser uma referência em parto humanizado e tudo mais, a gente queria ter uma experiência dessas, evitar passar por uma cesárea, enfim, qualquer coisa que fosse mais natural possível e acho que essas informações ajudaram muito pro momento do parto.

E18: Olha, muitas coisas do grupo mesmo sendo terceiro filho eu nem sabia. Plano de parto então, gente, o que é plano de parto? Eu não ia saber nunca na vida o que é um plano de parto, eu fui saber pelo grupo, aí que eu fui pesquisar e falei "olha, existe plano de parto?". [...] Eu gostei muito, o grupo auxiliou de todas as formas, por exemplo teve uma parte no final do meu parto que eu tive uma dúvida dai eu procurei a enfermeira no privado e ela me ajudou bastante também, eu acho que eu tava começando a ter os sinais do parto, e eu não tava associando com sinal do parto e acho que passei uma semana assim, com dor de barriga mesmo assim e tava tendo diarreia e eu "sei lá, isso é normal?" ela falou "olha, provavelmente isso é um sinal do parto" eu nem imaginei que isso poderia ser um sinal do

parto, ela também prontamente respondeu minha dúvida e o grupo acho que ele é realmente pra isso, ele é pra tirar suas dúvidas, ele é pra te dar o apoio umas às outras, contar a experiência como ta sendo e te orientar nas coisas que você não sabe, eu não sabia muitas coisas e eu fui ver que eu não sei onde é que eu tava pra não receber essas informações.

E18: É, a gente pode até pensar que tá preparada mas acho que mesmo a pessoa mais preparada do mundo ela precisa de um grupo desse onde ela se sente inserida, onde ela vê que tem outras pessoas que estão lá pra te dar apoio, como foi o caso que eu falei com a enfermeira, mas também pelas orientações que a gente tem, acho que é muito importante a gente trabalhar essa parte em grupo. Olha, nunca tive a experiência de “ah, vou conhecer a maternidade antes de ter o bebê” eu não tive essa experiência antes, mesmo por fotos “vou te apresentar mesmo que for por fotos” eu não tive essa oportunidade, já cheguei pra ter todos os dois e isso é bem, tanto por exemplo a orientação pra exercícios e até mesmo porque conhecimento nunca é demais “ah eu já tive" se fosse por exemplo “ah não, já tive dois filhos, eu não vou entrar nesse grupo” não, eu vou entrar, eu preciso mais ainda porque ja fazia cinco anos que eu tive meu segundo filho eu disse “não, eu vou entrar, pode ter mudado alguma coisa, tem coisas que eu não sei” orientação a mais nunca é demais, podem ter coisas que no grupo deve ter passado lá que eu ja sabia mas a gente fica na nossa, fica guardado e pega a orientação de novo, pra mim não tem problema e aprendi muita coisa que eu não sabia do plano de parto por exemplo é uma delas, que eu podia aprender com o grupo e foi muito bom, não tenho o que reclamar do grupo, pena que a gente não conseguiu fazer presencialmente nenhum encontro, só a única coisa que eu fico triste é esse encontro que a gente não pode ter, assim de se conhecer pessoalmente.

E20: Foi legal ter mantido o grupo ta, vire e mexe, eu não sou tão participativa mas eu vejo que é muito legal porque tem muitas dúvidas daí as mães trocam muita experiência ali e nesse momento de ser mãe é muito legal essa troca, então tem muita troca de conhecimento e é bem legal, pequenas coisas tipo “ah, meu filho não tá dormindo a noite” aí todo mundo fala “ ah, mas isso é normal, aqui em casa acontece também”, só o fato de dizer que isso é normal nas dificuldades eu acho que nos estimula a seguir adiante sabe, porque tem dias difíceis, tem dias bem difíceis que pensa assim “ Meu Deus, como que eu vou aguentar mais duas noites sem dormir” mas as noites melhoram (risos), elas melhoram.

E21: O grupo foi bem legal, eu tinha participado já como bolsista quando tava na faculdade então eu já conhecia, sabia como é que funcionava, foi muito bom pra poder trocar ideia com outras mães, com outras famílias, o grupo de Whatsapp que eles criaram.

E22: O grupo do HU foi uma pena ter sido online porque eu busquei o grupo justamente porque tinhas ótimas referências né, inclusive por que acaba sendo, as pessoas se conhecem acaba formando uma rede de apoio legal, no nosso caso a gente tem o grupo ali mas como ninguém se conhece pessoalmente acaba não sendo tão bacana né assim, mas foi a título de informação com certeza né, a gente teve bastante esclarecimento. [...] Embora sabe fosse muito melhor se fosse pessoalmente a gente vendo né, ensinando a banho, isso aquilo, assim, são coisas que tu busca na internet, tu vê video mas saber que tem uma pessoa ali com

propriedade, profissional e tal te falando, uma pessoa que trabalha com isso né diretamente, te da um pouco mais de segurança assim né e assim, não foi tão agregador quanto eu imaginava mas por isso porque a gente ja tava indo muito atrás de informação, a gente fez outros cursos nesse mesmo estilo assim né, eu acho que a diferença maior é essa assim, o HU é uma instituição super respeitada, é saber que são profissionais dali que trabalham com isso há anos, então ipo as informações te dão uma segurança maior sabe.

13. OS REFLEXOS DO PREPARO PRO PARTO NO MOMENTO DO PARTO

E2: Comecei a fazer muita força, muita muita, ai eu já não tinha mais dor, dai eu comecei a fazer força, dai eu me dei de conta "eu acho que eu ja to no expulsivo, eu acho que ela já vai nascer né" aí a gente foi pro quarto e chamou a médica, aí quando eu cheguei no quarto e já me sentei no banquinho de cócoras, era a melhor posição pra mim.

E2: Eu acho que o que foi importante é que eu já tava familiarizada com tudo, elas já tinham me falado como que era, que a enfermeira vinha de tanto em tanto tempo alí, eu já conhecia a sala, eu já sabia o que que ia acontecer ali sabe. E tudo isso mais ou menos eu já tava familiarizada, sabe, foi isso assim. Eu fazia bastante as respirações assim, que a gente tinha conversado né, foi o que me ajudou muito foi ter feito as respirações, aí isso uma das colegas do grupo passou também umas vocalizações que eu fazia e foi o que me ajudou muito assim a ficar tranquila, relaxada, eu fiquei super tranquila.

E2: A gente sabia de tudo assim, eu sempre falava pra ele se vierem colocar acesso por algum motivo a gente não quer, a gente não quer nada nada nada farmacológico assim, eu queria totalmente natural. E aí o que me ajudou também, o que eu acho que me ajudou muito foram as respirações e as vocalizações que eu fazia na hora das contrações, isso eu já vinha treinando antes do parto. [...] a contração é como uma onda, ela chega no ápice da dor e aí quando ela chegou no ápice da dor ela começa a diminuir, então na hora eu colocava, aquilo ali era o que eu mentalizava assim sabe, eu ia aguentando aguentando aguentando e quando ela chegava no auge da dor eu pensava assim "ah agora ela vai diminuir" e aí eu ia fazendo as respirações e as vocalizações e ia relaxando, relaxando, relaxando. Eu fiquei bem concentrada, muito muito concentrada. [...] Eu não fiquei racionalizando nada sabe, eu fui só tentando levar, quando vinha a dor eu só pensava em tentar aliviar aquela dor, e deixei, não fiquei prestando atenção "ai quando tempo será que eu ja to aqui, será que ela já vai nascer será que não vai", tanto que quando eu comecei no expulsivo eu comecei a fazer força força dai que eu me dei de conta de, peraí, eu acho que já não to mais nas contrações, eu acho que ja ta na hora dela nascer, então eu ia indo muito assim sabe, eu não racionalizei nada eu fui levando.

E2: Quando a gente fez o curso aí elas falaram que a posição que eu ia ganhar a [nome da filha] era a melhor posição que fosse pra mim, eu escolhia a posição né, elas mostraram que tem disponível lá, isso no curso, elas mostraram que tinha disponível o banco de cócoras, que tinha a maca, mostraram fotos da maca como a gente não pôde conhecer a sala elas fizeram um vídeo e tudo né. e o tempo todo que eu tava em trabalho de parto a melhor posição pra

mim era sentada no vaso, eu sentava e ficava relaxando um tempão assim e depois eu ia pro chuveiro, então na hora que eu saí, que eu tava no banheiro eu tava no vaso e eu fazia muita muita muita força, ai com certeza eu tava no expulsivo. E aí quando eu fui pro parto eu só conseguia sentar no banco de cócoras, eu não conseguia nem sentar na bola a melhor posição pra mim foi no banco de cócoras aí quando a médica chegou ela falou assim "ah agora quando aliviar tua contração a equipe vai chegar e tu vai te deitar na maca" ai eu falei pra ela assim "não pra maca eu não vou ir, eu não vou conseguir ir pra maca" e ai ela assim "vai sim, a gente vai te ajudar e vai te levar pra maca" aí eu falei assim " não, daqui eu não saio e daqui ninguém me tira, eu vou ganhar ela aqui" aí ela pegou e disse assim "ah a tua sorte é que eu to com o joelho bom, traz lençol" aí mandou trazer lençol e ajeitou ali, então isso foi uma informação que me empoderou assim, porque uma que eu não ia sair dali porque eu não tinha condições realmente, ela ja tava quase nascendo era a melhor posição ali pra mim, eu não conseguia ficar deitada nem sentada na poltrona e outra também que eu já sabia que eu poderia escolher a posição que fosse melhor pra mim né.

E3: Eu tava muito consciente assim do que que eu queria, muito consciente assim, tanto que eu me senti forte o suficiente pra chegar lá na hora e dizer "Não, eu não vou com ele, eu vou chamar a [nome da profissional], eu vou aceitar a indução do trabalho de parto" e me senti tranquila pra receber o diagnóstico dela e saber que eu tava em boas mãos, porque essa insegurança em relação aos médicos eu também vivi e por isso que naquele momento que a coisa não tava no caminho que eu tinha planejado né, do natural, ai eu achei melhor ir nas mãos de uma profissional de confiança.

E4: Na verdade eu já fiquei assim uns cinco dias sentindo contração só que elas não ritmavam, aí eu, das minhas leituras né (risos), eu sabia que valeria a pena ir pra maternidade só quando elas tivessem ritmadas. Aí então na quarta-feira ela começou assim às seis da noite, começou a dar a contração de 5 em 5 minutos e bastante contração assim, aí foi quando a gente decidiu ir pra maternidade mesmo, foram esses sinais da contração que eu, que na verdade elas pegaram ritmo né, foi o maior sinal assim que eu senti que era hora.

E6: Eu senti que naquele dia o bebê tava se mexendo menos e aí eu fui pra lá pra poder ver se tava tudo bem, tava aguardando um pouco mais porque eu queria entrar em trabalho de parto, eu queria que as contrações tivessem ritmadas pra poder ir né, por causa da questão da segurança com relação covid mesmo e enfim, o que foi orientado foi isso da gente esperar o ritmo das contrações pra poder ir pro hospital, mas aí naquele dia eu achei que ele tava mexendo menos e eu fui mais cedo. [...] Eu tinha muitas contrações de madrugada e durante o dia elas ficavam espaçadas de hora em hora, assim sabe, eu conseguia descansar, conseguia comer e vinha uma e parava então a decisão foi mesmo assim, vamo esperar vir as contrações mais ritmadas, mas próximas eu acho que de 15 ou de 10 minutos não sei, e quando chegar esse momento a gente vai pro hospital, porque o medo também da covid de pegar sabe, então a gente não queria dar bobeira de ficar ali exposto e como eu entrava no HU eu ficava sozinha ali naquela sala de espera eu ficava muito preocupada, eu fiquei bem neurótica sabe, eu fiquei como já chamam por aí a "louca do álcool" eu ficava com muito medo de pegar, de passar pro bebê, de não saber como seria com ele e comigo. Ai a gente esperou ficar mais

próximo, mas quando esse dia amanheceu na sexta-feira no dia 5 eu realmente senti uma, que ele não tava mexendo tanto e foi isso, esse momento que a gente decidiu que, como as contrações já tavam mais próxima e ela não tava mexendo tanto a gente achou por bem que fosse a horra de ir pra maternidade definitivamente pra ver se, sobre uma avaliação, o médico a médica achasse que era a hora sabe.

E9: Como estava com analgesia, não sentia a dor da contração, só a bebê descendo. Não era uma sensação agradável, mas também não tão ruim assim. Fiz bastante força. Vi nessa hora que ter feito exercício físico a gestação inteira até o final e trabalhado perna, braço e a musculatura da região pélvica ajudou demais. Cada força que eu fazia ela descia bem. Tanto que não chegou a durar 1 hora essa parte.

E10: Mas toda essa informação a gente pode colocar em prática, muita muita muita informação que a gente tinha, com relação a posição, com relação a não entrar em desespero quando a bolsa estoura né, se manter mais centrado durante o trabalho de parto também, tudo isso foi muito bom, tanto pra mim quanto pro meu marido.

E10: Mas o que mais eu lembro de ter aproveitado nos encontros as meninas explicando sobre a fisiologia do parto e sobre as posições, o que a gente poderia optar né, elas falaram da posição de quatro apoios que era uma posição muito adequada que facilitava muito, mas que as mulheres hoje em dia tem optado bastante pela cócoras pelo banquinho e tudo e aí na hora que eu fui pra sala de parto o médico perguntou, ele ofereceu o banquinho e aí eu disse "não, eu vou primeiro tentar então ficar de quatro apoios" e aí isso foi assim uma das informações que deu super certo pra mim, porque então eu fiquei de quatro apoios estava super relaxada, meu marido tava ali comigo e aí eu me senti bem confiante nessa posição e ela nasceu assim. [...] A gente teve laceração de grau 1 não precisou dar ponto, não precisou de nada, foi bem tranquilo, então essas informações foram muito úteis que eu consegui colocar em prática né durante o trabalho de parto.

E10: A gente recebeu o positivo e começou a estudar as pensar nessas possibilidades do parto, mas foi assim primordial porque sem informação acho que tudo seria muito mais difícil, do ponto de vista de conseguir contornar as dores, de conseguir se manter estável sabe e até depois, talvez se eu tivesse optado por uma cesárea eu não tivesse conseguido dar tanta assistência pra ela né, então, então foi nossa muito bom, primordial.

E13: Fui pra casa da minha mãe almoçar bem de boa, e as contrações começaram a acelerar, então o negócio começou a pegar, não tive estouro de bolsa nada nesse momento, aí fui pra casa comecei a acompanhar de acordo com as orientações do curso que a gente fez né, pra acompanhar o ritmo, o tempo das contrações, utilizei um aplicativo porque na hora a gente não consegue pensar e contar, não existe isso, é muito difícil e o aplicativo dava essas orientações, chega um certo momento que elas tavam próximas e ritmadas que ele dizia "vai pra maternidade, vai pra maternidade" mais ou menos assim, mas eu fiquei segurando porque era bem suportável assim as contrações, não era nada absurdo.

E15: Tentei fazer o parto mais ativo possível, cavalinho, bola e tudo mais, peguei mesmo todas as dicas, todas as informações de estudo e tentei fazer um parto mais ativo possível, meditação, meditação muito na hora ali né, pra tentar amenizar a dor e posições eu escolhi, não foi um parto, não foi deitada, foi meio de cócora, eu sabia até a hora de pedir anestesia, eu sabia que eu podia apelar pra anestesia.

E15: A importância pro meu trabalho de parto fez total diferença pra que fosse do jeitinho que eu queria, foi tudo do jeito que eu queria, a única coisa que foi induzido que né, era uma parte que eu queria que fosse mais natural ali, mas foi perfeito, e cheguei com meu plano de parto que foi falado no grupo levei o plano de parto mesmo sendo um hospital público e eles respeitaram totalmente assim né, [...] não me traumatizou, muitas se traumatizam com o parto, pré-parto né, não me traumatizou nenhum um pouco assim, não foi traumatizante, lógico que eu saberia que ia doer mas justamente o preparo nesse período foi essencial, como no grupo a gente falava a dor é inevitável mas sofrer aquilo ali que não é o legal né, não é legal nem pro bebê. Então aproveitei bastante cada momento, tanto do parto, tentei ser o mais ativa possível.

E16: As contrações foram e as horas foram se incrementando e eu comecei a ja me sentir muito desconfortável, aí eles me recomendaram para começar a caminhar ali pelo espaço que tinha disponível eu fui tomar banho de chuveiro, tava fazendo movimentos na bola, todas essas coisas que passavam no pré-natal que era pra fazer eu fiz.

E19: Eu lembro tipo do grupo trazer várias questões nesse sentido, que tu falou, de reforçar as questões ideais vamos dizer assim, a questão do parto normal tipo de optar porque muitas mulheres ficam com medo de parir e aí optam pela cesárea só por medo, não por alguma situação necessária pra ela ou pro bebê e também lidar com essas questões assim tipo de que não é assustador, não é assustador não, é que a gente tem que enfrentar né mas, não é essa coisa horrorosa tipo pra mim de toda minha maternidade a experiência mais legal que eu tive foi o parto assim, tipo eu tenho uma lembrança muito boa sabe, pra mim eu me senti renascendo mesmo é uma entrega muito, muito única assim, eu não teria, eu não penso em ter outro filho de barriga assim mas eu queria muito parir de novo, a experiência foi muito boa. [...] eu realmente consegui sentir e fui muito respeitada assim no meu processo né, isso também fez muita diferença assim, é uma das coisas que eu penso é como seria no hospital assim, porque tipo eu vivi muito meu tempo, tipo assim "Ah, agora eu quero deitar. Agora eu quero tomar banho. Agora eu não quero que ninguém fale. Agora eu quero escutar música." tipo eu fiz tudo no meu tempo, em como eu fui sentindo meu corpo assim né, então não sei se no hospital, eu já escutei vários relatos que tem uma pressa então acaba não sendo tão no teu ritmo assim, e aí isso é uma das coisas que eu também eu acho que também por eu ter uma boa lembrança, um dos fatores de eu ter uma boa lembrança.

E21: Tocou minha playlist que eu já tinha feito uma playlist pro parto, pro trabalho de parto e eu pedi pra eles colocar desde quando começou a montar os campos já tava tocando a minha playlist, na hora de ele nascer tudo e ter o pele a pele tava tocando as minhas músicas então fiquei bem feliz com isso.

E23: Hoje eu faria tudo diferente (risos) tá, assim primeira coisa eu tava até aconselhando uma amiga minha que vai ter bebê agora próximo do natal, assim ó primeira coisa que eu faria, eu na minha cabeça eu tava numa fase bem "Workaholic" da minha vida tá, trabalhando, trabalhando, trabalhando, tanto assim que eu fui muito cansada ter o [nome do filho] sabe, os primeiros dias pra mim foram bem assim desgastantes porque eu estava cansada de tanto que eu tava trabalhando, então assim, hoje eu faria completamente diferente tá, eu iria trabalhar mas eu ia pedir pra sair de licença um pouco antes né, claro no caso do [nome do filho] 37 semanas então né normalmente as mulheres tem com 38 né, mas eu ia ter me programado pra sair um pouquinho antes né e ia ter me programado agora que eu conheço, agora que eu vi, agora que eu consegui ler um pouco mais sobre isso depois né, que nem eu te falei um erro meu também né, mas enfim, mas eu ia ter pedido pra que fosse um parto um pouco mais humanizado, pra que conversassem comigo sabe, pra que me deixasse ficar um pouco mais pertinho dele não só o rostinho dele, que colocassem ele realmente em cima de mim, sabe aquela coisa assim do primeiro contato né? Mas realmente hoje eu ia fazer tudo diferente, eu ia sair mais cedo do trabalho pra ter aquele tempo pra mim, pra descansar. [...] eu me sentia uma grávida tão bem, tão bem, tão bem, eu era tão feliz de tá grávida, tão grata porque eu achava que eu não ia mais conseguir engravidar, que eu não via problema em nada, meu astral tava lá em cima todos os dias sabe, então por isso que eu não queria ler sobre problemas, eu não queria pensar em problemas de tão bem que eu tava sabe. [...] então hoje eu já ia fazer tudo diferente, eu já ia conversar sobre o parto, eu ia planejar essas coisas sabe.

14. EXPERIÊNCIAS DO PARTO:

E13: Tava bem lotada e eu não tava na sala de parto eu tava num quarto nesse momento sabe, aí como eu queria parto normal né, eles tentaram achar uma sala de parto, não tinha disponível aí eu fui pro centro cirúrgico sentar no chão pra tentar fazer o parto, só que isso a gente vai ficando tensa, eu confesso que eu fui ficando bem tensa porque não tinha nenhuma acessoria assim, era eu e meu marido e a equipe de enfermagem me acompanhando e tal, e aí fui aquilo ali me deixou bastante tensa em virtude da pandemia, foi então assim, eu tinha muita neura da sujeira do chão, dessas coisas na minha casa eu cuidava muito, imagina eu tentar ter um parto no chão do centro cirúrgico então eu tava assim bem bem nervosa

E13: Quando eu tava lá embaixo eles me ofereceram a analgesia, eu acho que tudo pela minha tensão e tudo mais, até então eu não ia aceitar mas acabei aceitando, muito pelo meu nervosismo da situação, daí cessou as dores só que dai eu não conseguia fazer com que elas evoluíssem né, daí fui pra sala de parto ela foi voltando gradativamente o ritmo das contrações e tudo mais, enfim assim, acho que até às três e meia da manhã eu cheguei a nove de dilatação, nisso tava rolando um outro parto normal um ou dois ao mesmo tempo só tinha um médico obstetra na sala ao lado, e eu fui ficando muito tensa, ficando mais tensa ainda né, pandemia e tudo mais e aquilo ali foi me deixando super nervosa, eu já não sabia mais trabalhar com a dor, fazer a força e tudo mais, trabalhei várias posições e nada nada e nada e

chegou uma hora que eu gritei “Eu quero que alguém faça uma cesárea!” mesmo com nove de dilatação.[...] A pandemia deixa a gente bem enlouquecida assim sabe, daí eu tomei a decisão eu falei “Não, muito tempo cara, eu não sei mais o que fazer” eu não sabia mais que movimento fazer pra me ajudar a ter o parto normal. Eu sentia dor e eu não sabia fazer a força, se eu soubesse fazer a força eu acho que eu iria finalizar tranquilamente, mas o meu emocional por eu estar sozinha, sem nenhum apoio, meu marido não sabia como me ajudar, o médico mandando fazer força de cocô, ridículo assim que a gente sabe que não é assim, e eu falei e eu gritei “Eu não faço força pra fazer cocô” eu gritei na hora, porque eu fiquei muito chateada assim, porque a gente sabe que pô isso é padrão falar e ele não é mulher ele não sentiu. [..] Eu acabei ficando muito nervosa, e a pandemia foi algo que me deixou, foi a gota d'água porque eu sempre me cuidei bastante na pandemia, a gestação foi uma gestação muito desejada e tudo mais então ela me deixou bastante tensa assim, aí eu optei por fazer a cesárea, fomos pro centro cirúrgico, fiz a cesárea e de tão cansada de tanta força que eu fiz ela nasceu, minha bebê nasceu foi aquela emoção eu vi e apaguei, eu cheguei a roncar na mesa assim, apaguei de tão exausta e eu não tive aquela hora mágica, dourada e tal, contato com ela pele a pele e tal, não tive, fui ter na sala de recuperação e eles colocando ela ali mas eu não tinha força nem pra segurar, eu não conseguia, meu marido ficou ali tentando e ele ficou com ela, a gente tentou dar o peito amamentar ali mas eu realmente não tinha força e meu marido graças a Deus ficou com ela, daí depois fui pro quarto, ai depois sim, ai eu fui voltando, fui recuperando as energias, devo ter tirado um cochilo de sei lá, quarenta minutos assim, aí seguiu aí voltei, mas essa é minha experiência de parto.

E15: Então, como foi induzido a partir de 40 semanas o médico começou a dizer “Ò, tem que começar a ir na maternidade”, como eu já sabia até a maternidade que eu queria ganhar que era lá, eu comecei a ir lá, lógico que tem médicos bons e médicos não tão acolhedores, médicos frios, e aí logo que eu cheguei lá eu peguei um médico mais desse e os outros todos não tenho o que reclamar, nem enfermeiro nem nada. Então, ele queria que eu fizesse cesárea, ele queria que eu chegasse e ja fazer uma cesárea e disse assim “Se tu chegar aqui com 41 e se tu não quer induzir tu nem vem pra maternidade e tal tal” bem assim desse gênero.

E16: Era muito novo assim, e aquelas, claro eu estava com esse pacote de informações do curso, as coisas que tinha ali, eu até tinha levado um plano de parto, eu tinha colocado que eu não queria remédio sabe, que eu não queria coisas mas no final como estava indo muito devagar com a dilatação eles ficaram colocando ocitocina e por exemplo, eu não queria mas acabei aceitando sabe, porque a gente também não sabe como lidar com os médicos, não sabe se posicionar porque você pensa que são eles que sabem né, que você não sabe e isso é um erro porque a mulher sabe, a mulher sabe parir, mas você não sabe que sabe e acha que tem que deixar tudo nas mãos deles, o tempo todo, então eu senti isso depois, que eu poderia ter me, talvez me posicionado mais, talvez deixar mais claro meus desejos sobre o parto, teve momentos que eu me senti muito vulnerável assim, quando me induziram o parto foi via vaginal e sabe, eu não perguntei nada mas eu deixava que eles fizesse porque era isso né, eu também não tinha como perguntar se aquilo tava sendo certo se não, então você fica muito nas mãos deles,e você vai com uma ideia de como quer seu parto, como quer que aconteçam as coisas mas também às vezes por causa mesmo do processo do parto isso muda muito, e

você não tá no controle e eu acho isso uma pena porque deveria ser um pouco ao contrário, deixar a mulher estar no controle e ali os médicos intervirem caso precisar e não ao contrário. Eu acho que tá ainda muito ao contrário, a questão do médico que tá as coisas no controle e a mulher meio que fica quieta, deixa que a gente sabe, um pouco isso.

E16: a gente tem medo, a gente não sabe, é a primeira experiência né, eu acho que agora se eu fosse ter outro filho aí eu ia saberia muitas mais coisas, eu diria não, não quero isso, quero isso, sabe. Mas como é a primeira experiência você tá presa no medo, você realmente sabe que deve deixar nas mãos deles, pensa que deve deixar nas mãos deles e é muito difícil que aconteça o que você está esperando que aconteça, então mas assim eu acho que o momento foi uma experiência boa, o meu filho digamos que nasceu bem, aquela coisa do desconforto foi só no momento dele nascer porque eu demorei muito pra parir né, ele ficou muito tempo no canal de parto e parece que isso gerou alguns desconfortos nele mas no dia seguinte eu já estava lá com ele, e essa questão do pós-parto foram coisas que tempo depois com mais informação, porque o bebê nasce a gente continua na trilha do conhecimento né, aí eu já me dei conta que aconteceram coisas que também eu quisesse ter evitado sabe, porque por exemplo ele ficou na UTI naquele primeiro dia e ninguém me falou que era pra eu ir lá para amamentar ele, para tirar o colostro, sabe, não, eles deram fórmula, e eu não queria isso sabe. Então coisas assim, depois eu fiquei, é pra você sair da maternidade depois um ou dois dias depois, o bebê nasceu na madrugada de domingo e eu fiquei até meio dia da quarta-feira porque não tava conseguindo amamentar ele de uma maneira efetiva, então o pessoal lá não me dava a alta por causa disso, mas assim aconteceram outras coisas que também com mais informação talvez não tivesse permitido.

E17: Quando eu cheguei tava super lotado, aí eu comecei com contrações mas super de leve, nada muito forte, nada, super de leve, mas eu fui mesmo porque eram sete e meia da manhã, minha bolsa tinha estourado uma e meia então já tipo, tinha que saber se tava tudo certo e enfim, só que tava super lotado, super, super, não dava pra ficar um metro de distância com ninguém e o cara que tava ali, o obstetra que tava ali na emergência que me fez um check up de tudo falou “sim, tua bolsa estourou só que você ainda não ta suficientemente dilatada, ainda vai começar a evoluir e eles fizeram um cardiotoco meu filho tava super bem só que eles não tinham como me internar porque não tinha vaga, não tinha leito, não tinha nada, eles falavam que eles queriam tipo me ajeitar ali no centro cirúrgico, me deixar ali num lugar que eu não ia ter nem banheiro, nem conseguia comer e não podia fazer nada, só pra me deixar ali porque eu tinha que ingressar só que não tinha leito e eu não sei falei “cara, meu parto não vai evoluir, não vou ter um parto normal que eu quero num lugar que eu não vou ter nem banheiro, nem vou conseguir comer, nem vou conseguir fazer nada, então dai eu liguei pra minha doula minha doula falou “não aceita isso pelo amor de Deus, liga pro teu convênio” e aí começaram a brigar e nessa briga que minhas contrações pararam né, meu trabalho de parto não evoluiu mais porque a [maternidade] não queria me liberar depois o obstetra foi fazer uma cesariana, depois voltou, imagina que ele só me liberou a uma da tarde e eu tinha chegado sete e meia da manhã, eles queriam fazer meu marido assinar, aí eles perceberam que eu era estrangeira, acharam que ai eu não podia entrar com acompanhante tinha que entrar sozinha, dai começou a falar que meu filho ia ter sequelas porque eu devia ficar

esperando ali, ele não podia garantir que ele iria nascer bem que eu tinha que ingressar mas, eu comecei a ficar meio revoltada e chamei meu marido [...] porque o convênio precisava de um documento assinado pelo médico que falasse que eu precisava se internar só que não tinha leito, só que o cara não queria assinar isso, acabou assinando mas eu acabei chegando duas da tarde no Ilha e aí o cara falou “tem doze horas que você estourou a bolsa, teu trabalho de parto não evoluiu então você vai ter que fazer com indução mesmo” [...] não sei se tivesse evoluído sem essa briga mas por conta dessa briga tudo parou eu cheguei tinha um centímetro ou dois quando chegou no Ilha então não ia evoluir mais e depois de doze horas o protocolo mesmo era induzir.

E17: Não, e o pior de tudo é que velho eu acho que foi uma violência obstétrica né, o cara me falar que meu filho poderia nascer com problema por conta disso, que eu deveria esperar, eu sinto que realmente ele queria ganhar com o parto, que ele queria ganhar o dinheiro de eu ganhar meu filho ali, de ter o [nome do filho] ali, então eu comecei a me sentir assim, que o que ele quer é que eu ganhe porque pra ele é um cheque, mais um parto, só que ele nunca olhou pra mim, nunca olhou pra questões importantes que eu não tinha um lugar, não ia ter um banheiro, estava toda molhada, tava com um absorvente pós-parto só que vazando inteiro, eu tava super desconfortável, não dava pra ficar um metro de distância de ninguém numa pandemia que a gente não conhecia muito como ia reagir a grávida e o bebê, porque ainda tava sendo estudado tudo isso, a gente tinha que ficar com essa máscara que não conseguia respirar o tempo inteiro, tudo sabe, e até a questão de um minuto né que eles me trataram como se eu não tivesse entendendo, eu falava pro meu marido “eu sou estrangeira mas não tenho problema de entendimento” sabe, até a enfermeira falava “é você não vai evoluir, você vai, quase que teu parto não vai evoluir, você não vai ter contração, você não vai dilatar” e eu “cara, vocês não tem porque falar isso pra mim” .

E23: eu tava com muito medo, eu entrei na sala de cirurgia lá sozinha assim né, depois que o [nome do companheiro] veio e eu tava com muito medo assim, sei lá né tudo novo na vida da gente, naquela hora que rompe a bolsa é uma alegria, uma choradeira, uma tremedeira, tudo ao mesmo tempo né e aí eu dizia pra ela assim “Ai doutora segura a minha mão” quando tava fazendo anestesia né daí ela ta segurava a minha mãe aí depois eu disse pra ela “Por favor vai me falando tudo que tu tá fazendo, pra mim ir acompanhando” sei lá, se ela ta cortando, se ta fazendo não sei o que, eu queria pelo menos saber que estágio que tava né, e ela não me falava nada sabe, e aí simplesmente a anestesista ficou do meu lado né, e eu comecei a escutar aquele barulho de bisturi e eu “Ai meu Deus será que ja tão me cortando? Será que ja tão” ai eu ai meu Deus, gente isso me deu uma sensação tão ruim, tão ruim, presa amarrada naquela máquina, naquela cama, sem a tua médica falar contigo, a anestesista que eu nunca tinha visto na frente falando comigo sabe, o [nome do companheiro] do lado coitado acho eu apavorado também com tudo que tava vendo, ele disse que tava vendo tudo né, não falava direito também né, aquilo me deu um nervoso, nervoso, nervoso, uma ansiedade eu dizia assim “Ai me tira daqui” eu dizia pra anestesista, queria que ela colocasse um sedativo assim pra me desligar. [...] então todas essas coisas assim que depois eu fui lendo e na verdade vendo que isso aí faltou comigo sabe, talvez eu não, pelo fato de eu não ter lido nada sobre isso, não ter cobrado da médica antes né, foi um erro meu também ela achou bom, que ela ia

fazer do jeito que ela achava melhor né, só que depois eu fui lendo e vi que “Ai eu não tive isso, ai eu não tive isso” sabe aquela coisa. [...] eu escutava elas as médicas falando, porque assim ó eu tive um problema também no útero, eu acho que tinha uma variz pelo que ela me explicou, uma veia meio grande eu não sei como que é sabe e eu escutava ela dizendo “Ah, mas isso aqui parece que ela tem quadro de endometriose” umas coisas assim, falando assim entre elas e eu “Ai, será que sou eu” [...] Eu fiquei assim completamente no vácuo, sabe, “Será que era comigo, será que elas tão falando de mim? Será que elas tão falando de outra coisa? Será que” sabe aquela coisa, que eu me lembre assim foi tudo muito rápido mas ao mesmo tempo que foi rápido foi meio que assustador assim sabe.
