# Supplementary material for: Adaptation strategies for preparing for childbirth in the context of the pandemic: Roy’s Theory
Source: Rev Bras Enferm. 2024 Jul 29;77(3):e20230159. doi: 10.1590/0034-7167-2023-0159 (PMC11290742; doi:10.1590/0034-7167-2023-0159)
Supplement: Translation

# Translation: Estratégias de adaptação no preparo para o parto no contexto de pandemia: Teoria de Roy

**DOI:** 10.1590/0034-7167-2023-0159pt

**Authors:** Letícia Pickler, Margarete Maria de Lima, Ariane Thaise Frello Roque, Laís Antunes Wilhelm, Felice Curcio, Dionara Guarda, Roberta Costa, Isadora Ferrante Boscoli de Oliveira Alves

**Affiliations:** I Universidade Federal de Santa Catarina. Florianópolis, Santa Catarina, Brasil; II Universidade de Estudos de Sássari. Sássari, Sardenha, Itália

**Keywords:** Gravidez, Educação em Saúde, Parto, Pandemia, Estratégia de Adaptação.

## Abstract

**Objetivos:**

conhecer o processo de adaptação ao parto durante a pandemia de covid-19, a partir da perspectiva de um grupo de gestantes.

**Métodos:**

realizou-se um estudo qualitativo, descritivo-exploratório com 23 mulheres. Os dados foram coletados entre outubro e dezembro de 2021, através de documentação e entrevistas semiestruturadas, analisados conforme a metodologia de Minayo e o Modelo de Adaptação de Roy.

**Resultados:**

identificaram-se diversos tipos de estímulos - focais, contextuais e residuais - que influenciaram a preparação para o parto. O grupo online mostrou-se essencial para facilitar a adaptação das gestantes, oferecendo um suporte significativo e gerando um feedback positivo para o preparo para o parto.

**Considerações Finais:**

identificou-se a importância de grupos de gestantes como estratégia para melhorar a adaptação ao parto, destacando a eficácia deste suporte tanto entre profissionais e participantes quanto entre as gestantes, fortalecendo a rede de apoio e a preparação para o parto durante um período desafiador como uma pandemia.

## INTRODUÇÃO

A pandemia causada pelo Coronavírus 2019 tornou-se um dos grandes desafios do século XXI, alcançando abruptamente um número elevado de infectados e de óbitos. As gestantes foram incluídas no grupo de risco, pois estão mais vulneráveis a desfechos desfavoráveis devido às alterações fisiológicas decorrentes do período. Assim, novos protocolos de atendimento foram implementados para reduzir a exposição delas e dar continuidade à assistência pré-natal^(1-2)^.

A gestação é um período marcado por intensas mudanças e está permeado por diversos sentimentos. Em um contexto pandêmico, há alterações significativas na estrutura social, culminando em uma quebra brusca da expectativa traçada por essas mulheres, principalmente em relação ao parto e ao nascimento^(3)^.

O parto, considerado um evento repleto de significados socioculturais construídos ao longo dos anos, para muitas mulheres está associado à dor e ao sofrimento^(4)^. Entretanto, com o auxílio de políticas públicas e diversos movimentos sociais, temos no Brasil um movimento de resgate da autonomia e do protagonismo da mulher na cena do parto, buscando consolidar um novo modelo de assistência ao parto no país^(5)^.

Grande parte dos receios, inseguranças e ansiedades em relação ao parto está associada à ausência de informação durante o pré-natal^(4,6)^. Mulheres que recebem orientações de qualidade e suporte contínuo desde a gestação vivenciam o processo de parturição com mais segurança e conscientização^(7)^. No entanto, o contexto da pandemia exacerbou medos e angústias nas gestantes, principalmente em relação ao parto, necessitando que essas mulheres buscassem mecanismos de adaptação para uma vivência positiva do parto e do nascimento^(8-9)^.

Callista Roy considera, em sua Teoria da Adaptação, que o indivíduo constitui um sistema holístico adaptável que, por meio de estímulos, ativa mecanismos de enfrentamento para desencadear respostas adaptativas ou ineficientes, que podem servir como reentrada no sistema^(8)^. O sistema é definido como um conjunto de partes que se unem para funcionar integralmente por meio da interdependência. Holístico é o termo que considera o seu funcionamento como um todo e não como soma de suas partes. Adaptável significa a capacidade de se ajustar às mudanças do ambiente e, dessa forma, também afetá-lo. Os sistemas apresentam entradas, processos, comportamentos e saídas, logo, a percepção da pessoa como um sistema adaptativo engloba quatro elementos: estímulos; mecanismos de enfrentamento; modos adaptativos e as respostas^(8,10)^.

Nessa dialética, tal teoria propicia a compreensão de como as mulheres enfrentaram esse duplo desafio de gestar e parir, especialmente no primeiro ano de pandemia, e como vivenciaram o processo de adaptação por meio da necessidade de se ajustar às novas mudanças. Assim, ampliar os conhecimentos acerca do tema é importante para reconhecer a contribuição das atividades educativas no período gravídico e a relevância da sua manutenção em situações de crise epidemiológica, destacando o grupo de gestantes como uma via de socialização e suporte para as mulheres e incentivando novas estratégias de educação em saúde voltadas para o preparo para o parto.

## OBJETIVOS

Conhecer o processo de adaptação ao parto durante a pandemia de covid-19, a partir da perspectiva de um grupo de gestantes.

## MÉTODOS

### Aspectos éticos

O estudo foi aprovado pelo Comitê de Ética em Pesquisa com Seres Humanos de uma Universidade Federal do Sul do Brasil. Os participantes assinaram o Termo de Consentimento Livre e Esclarecido (TCLE). Foram respeitadas as resoluções que fundamentam os aspectos éticos das pesquisas com seres humanos, 466/2012 e 510/2016, assim como o Ofício Circular Nº 2/2021/CONEP/SECNS/MS, que apresenta as orientações para procedimentos de pesquisa em ambiente virtual^(11-13)^.

### Tipo de estudo

Pesquisa descritiva e exploratória com abordagem qualitativa. Para orientação do rigor metodológico, foi utilizado o instrumento *Consolidated Criteria for Reporting Qualitative Research* (COREQ).

### Cenário do estudo

O cenário deste estudo foi um projeto de extensão de educação em saúde para gestantes e casais grávidos, vinculado a uma Universidade Federal e a um Hospital Universitário do Sul do Brasil. O projeto visa ao compartilhamento de experiências sobre o ciclo gravídico e puerperal, à divulgação de informações baseadas em evidências científicas, à promoção de uma rede de apoio e ao preparo para o parto. A equipe multiprofissional é composta por docentes, enfermeiros, uma psicóloga e acadêmicos de enfermagem e psicologia. Durante a pandemia, as atividades foram desenvolvidas em ambiente virtual, em resposta à necessidade de cumprimento das medidas de prevenção da doença. Geralmente, as mulheres são agrupadas por semelhança da idade gestacional; tais grupos são numerados e, até o momento, já somam 107 grupos desenvolvidos, sendo que 10 desses grupos foram realizados virtualmente, com a participação de um total de 304 mulheres e 281 acompanhantes.

Os temas relativos aos cuidados materno-fetais foram discutidos ao longo de sete encontros, além do acompanhamento das participantes pela equipe por meio de um grupo no *WhatsApp* ^®^, o que permitiu maior interação entre as participantes. Os participantes recebem materiais educativos em um grupo de mensagem para interação entre as famílias.

### Fonte de dados

Mulheres participantes do 96º e 97º grupo de gestantes e casais grávidos, na modalidade virtual realizados durante os meses de março a junho de 2020, foram convidadas a contribuir com esta pesquisa. Após contato com 45 mulheres, 23 aceitaram participar da pesquisa. Vinte e duas mulheres não retornaram o contato.

Critério de inclusão estabelecido: mulheres maiores de 18 anos que tiveram seus bebês entre os meses de março e dezembro de 2020, primeiro ano da pandemia. Critério de exclusão: mulheres que não estavam na lista de contatos do *WhatsApp* ^®^ dos participantes dos grupos 96 e 97.

### Coleta e organização dos dados

Os dados foram coletados por meio de duas técnicas: base documental e entrevistas semiestruturadas, no período de outubro a dezembro de 2021. O contato com as mulheres foi realizado por meio do *WhatsApp* ^®^, onde foram esclarecidos o tema, o objetivo e o direcionamento da pesquisa. Após o aceite do convite, foram acessados os arquivos do formulário de cadastro contendo seus dados sociodemográficos e obstétricos por meio do banco de dados do Grupo de Gestantes, de modo a caracterizar o perfil das participantes.

As entrevistas ocorreram por meio das plataformas *Google Meet* ^®^ e *WhatsApp* ^®^, em datas e horários de preferência das participantes, com duração média de 30 minutos. Foram conduzidas pela autora principal, na época estudante de graduação em Enfermagem, que recebeu treinamento para a técnica de coleta de dados e já possuía vínculo com as participantes devido a suas atividades no projeto de extensão. As mulheres estavam majoritariamente acompanhadas de seus filhos e não houve interferência na privacidade das respostas. Foi informado às participantes que poderiam solicitar a interrupção da entrevista a qualquer momento.

Utilizou-se um roteiro com perguntas abertas, no qual foram abordados assuntos relativos à percepção e experiência das mulheres sobre o preparo para o parto e a participação no grupo de gestantes, além de recurso de gravação de áudio para captura dos dados. Nenhum participante manifestou desconforto durante a entrevista. As perguntas não foram fornecidas previamente às participantes e não houve entrevistas repetidas.

A saturação dos dados ocorreu na vigésima primeira entrevista, contudo, optou-se por entrevistar as 23 mulheres que aceitaram participar do estudo. Os áudios foram transcritos na íntegra pela autora principal e armazenados em documentos identificados com o nome das participantes, enquanto os dados de identificação foram armazenados em uma planilha com a frequência absoluta e a frequência relativa em porcentagem.

### Análise dos dados

O procedimento analítico do presente estudo foi baseado na proposta de Minayo, adaptada ao propósito desta investigação. A análise de dados incluiu as seguintes etapas: pré-análise, exploração do material, tratamento dos resultados obtidos e interpretação^(14)^.

Durante a pré-análise, as entrevistas transcritas foram lidas na íntegra, visando estabelecer as etapas iniciais do processo interpretativo. Foram selecionados os trechos relacionados ao tema, a fim de estabelecer um direcionamento para o contexto de compreensão. A teoria da adaptação de Callista Roy foi o referencial teórico definido para guiar a análise dos dados, situando a enfermagem como facilitadora do processo de adaptação do indivíduo^(8)^.

Na etapa de exploração do material, após uma leitura exaustiva, as categorias analíticas foram definidas com base no referencial teórico adotado. Os trechos das falas foram codificados por letras “E” de entrevistadas, seguidas de um número ordinal (1 a 23), determinado pela ordem das entrevistas. No tratamento dos resultados, realizou-se uma leitura completa para estabelecer conexões e inferências dos dados, dando continuidade ao processo analítico em correlação com o referencial teórico.

Após as entrevistas serem criteriosamente analisadas, os dados foram agrupados em três categorias guiadas pelo referencial teórico de Roy: “Estímulos focais, contextuais e residuais no preparo para o parto”; “Modos adaptativos: grupo de gestantes como facilitador do processo de adaptação”; e “*Feedback* positivo ao preparo para o parto”.

## RESULTADOS

As mulheres apresentavam idade entre 19 e 39 anos, sendo 4% na faixa etária de 19 a 25 anos, 35% de 26 a 32 anos e 61% de 33 a 39 anos, resultando em uma média de idade de 32,9 anos. Em relação ao estado civil, 22% eram solteiras, 61% casadas e 17% não responderam a essa pergunta.

Quanto aos dados relativos à gestação, 78,3% estavam gestando pela primeira vez, 8,7% estavam na segunda gestação e o mesmo percentual na terceira gestação, enquanto 4,3% não responderam a essa pergunta. Em relação ao número de partos, 91,3% das mulheres eram nulíparas, 4,3% haviam tido um parto e 4,3% haviam tido dois partos até o momento. Quanto ao planejamento da gestação, 74% das participantes a planejaram. Todas as mulheres relataram ter realizado acompanhamento pré-natal, com 39% na rede pública, 57% na rede privada e 4% em ambas as redes.

### Estímulos focais, contextuais e residuais no preparo para o parto

Esta categoria aborda os estímulos focais, contextuais e residuais, conforme caracterizados por Roy, que estiveram presentes no processo de preparação para o parto dessas mulheres. Para as participantes, o ambiente foi fortemente influenciado pelo contexto da pandemia, gerando sentimentos de estresse e insegurança.

*A pandemia teve um efeito negativo na minha gravidez porque eu estava bem, estava saudável, sob controle, bem monitorada e aí assim essa mudança, esse estresse muito grande, toda essa mudança de cenário e essa insegurança que me causou eu acho que potencializaram o meu mal-estar da pressão* [...]. (E3)

Apesar de utilizarem ferramentas para manutenção desse contato, a restrição física foi relatada como um desafio para essas mulheres, que tiveram que lidar com este momento sem a participação idealizada de pessoas de sua referência.

[...] *foi um momento muito único mesmo, porque é você praticamente ter uma gestação e guardar só para si, é essa sensação que eu tenho.* [...] *Embora tivesse chamada de vídeo, foto, a convivência não foi possível,* [...] *foi muito restrita* [...]. (E6)

Além do afastamento da rede de apoio, os sentimentos de incerteza quanto à presença do acompanhante no momento do parto também foram considerados uma nova fonte de preocupação.

*Minha única preocupação era eu ter que parir sozinha, isso me gerava um medo porque eu senti que aquele suporte, aquele apoio do meu marido ia ser fundamental pra conseguir aquele parto que eu queria* [...]. (E17)

O parto apresenta significados diferentes para cada mulher; em algumas falas, o medo estava associado às experiências negativas anteriores ou, ainda, ao fato de ser uma experiência desconhecida.

[...] *a gente lia tudo aqui em casa, porque claro eu ia ser mãe de primeira viagem então a gente está com todos os medos em cima.* (E16)

*Não tive uma boa experiência no segundo parto, foi um pouco difícil* [...] *e eu sempre tive muito medo do final, da parte do parto, sempre foi uma coisa que me deixou ansiosa* [...]. (E18)

O medo da contaminação pelo vírus, o desconhecimento da doença, além da insegurança em buscar auxílio profissional, foram considerados alguns dos fatores limitantes no preparo para o parto.

*A pandemia afetou muito isso porque até na escolha de profissional, tu fica meio assim de ficar peregrinando* [...]*. E o próprio preparo perineal* [...] *eu fiz um pouco, mas daí lá pelas tantas já interrompia porque eu ficava com medo* [...]. (E21)

[...] *quando veio a pandemia o pânico de acontecer qualquer coisa comigo, com meu bebê era superior a tudo* [...] *então foi bem limitada a minha preparação, com muito medo* [...]. (E13)

### Modos adaptativos: grupo de gestantes como facilitador do processo de adaptação

Esta categoria contempla os quatro modos adaptativos (fisiológico, autoconceito, desempenho de papel e interdependência) descritos por Roy como comportamentos observáveis do processo de adaptação. O modo fisiológico refere-se à maneira que a pessoa responde como um ser físico aos incentivos ambientais. Conhecer a fisiologia do parto, os exercícios de relaxamento, posições confortáveis e métodos de enfrentamento da dor foram estratégias positivas para as mulheres.

[...] *aquele material de massagem pélvica,* [...] *foi perfeito, no caso eu não estava conseguindo arranjar uma* [...] *fisioterapeuta pélvica, então eu mesma fui autodidata, pesquisar vídeo, ver vídeo e material mesmo estava bem claro.* (E15)

[...] *ter feito exercício físico a gestação inteira até o final e trabalhado perna, braço e a musculatura da região pélvica ajudou demais. Cada força que eu fazia ela descia bem. Tanto que não chegou a durar uma hora essa parte* [período expulsivo]. (E9)

O autoconceito envolve aspectos psicossociais do indivíduo através do eu físico (sensação de autoimagem corporal) e do eu pessoal (auto ideal, autoconsistência e eu ético-moral-espiritual). Algumas mulheres definiram as mudanças enfrentadas nesse período como assustadoras. Também, referiram um desejo de compartilhar essas mudanças corporais e suas expectativas para a gestação.

[...] *lidar com a parte hormonal, as mudanças, eu não me reconhecia, eu não sabia o que estava acontecendo com o meu corpo. Isso foi muito assustador, foi horrível essa parte e por mais que soubesse que eu ia passar por aquilo era algo desconhecido,* [...] *acho que a preparação emocional foi o que mais fez diferença para mim.* (E19)

[...] *eu tinha uma visão diferente de quando eu estivesse grávida, de coisas que eu queria fazer, de estar na minha rotina de barrigão, eu dançava por exemplo, então eu queria estar fazendo dança com barrigão e não aconteceu nada disso* [...]. (E11)

O modo interdependência envolve as relações interpessoais e necessidades afetivas. Nesse aspecto, o apoio de familiares, parceiros, amigos e de outros sistemas de apoio é fundamental para o bem-estar do indivíduo.

[...] *eu nunca senti tanta vontade de estar perto da minha família, de ter a minha mãe do lado que eu não tive, minhas tias, todo esse mundo de mulheres, por isso o grupo foi tão importante, ter essas mulheres do lado* [...]. (E12)

[...] *O grupo foi bem importante pra gente manter essa troca de experiências.* [...] *Não via ninguém ao ar livre mesmo* [...]*. Então ali a gente criou um laço muito forte,* [...] *porque cada uma sabe que a outra está passando pelo mesmo processo* [...]. (E1)

[...] *fico até emocionada, porque era um momento que tu conseguias ter um pouco de contato, acesso, alguma coisa que te tirava daquele momento de tanta incerteza que a gente estava tendo da pandemia, acalmava, trazia uma serenidade, trazia informação de qualidade, então foi uma âncora mesmo.* (E14)

Roy considera que o desempenho de papel é o processo de se reconhecer em relação ao outro, logo, as pessoas desempenham papéis primários, secundários ou terciários. Desde o planejamento da gestação as mulheres apresentaram atitudes para a concretização do papel materno. Assim, ressaltaram a importância de os acompanhantes participarem de todo esse processo de aprendizado.

[...] *fiz toda a preparação física, mental e psicológica que eu podia fazer, me preparando pra essa questão da maternidade. Minha gravidez foi completamente planejada* [...]. (E8)

*A gente recebeu o positivo e começou a estudar as pensar nessas possibilidades do parto,* [...] *sem informação acho que tudo seria muito mais difícil* [...]. (E10)

[...] *de várias questões ali que a gente tem direito assim né, ainda ali na sala de parto,* [...] *pra garantir esses meus direitos.* (E4)

[...] *Ele participar de todos os encontros foi muito importante.* [...] *Ele não ia nem nos ultrassons, não ia nem nas consultas de ouvir BCF, era tudo por chamada de vídeo* [...]. (E5)

### *Feedback* positivo ao preparo para o parto

Esta categoria abrange a visão das mulheres frente às respostas adaptativas na preparação para o parto. De acordo com o Modelo de Adaptação de Roy, respostas adaptativas são aquelas que promovem a integridade da pessoa.

Nas entrevistas as mulheres relataram que apesar de vivenciarem a gravidez e o parto em um contexto pandêmico, buscaram seu potencial como mulher percebendo a preparação para o parto como uma atitude importante.

[...] *São muitos sentimentos e você se sentir entendendo pelo menos o processo ajuda.* (E7)

[...] *você precisa entender a transformação pela qual você vai passar, que não é só física, que é muito importante, não só emocionalmente, financeiramente* [...] *por isso eu acho que a informação é fundamental* [...]. (E16)

As mulheres consideram que a informação e o preparo emocional são fundamentais para o enfrentamento desse processo. Ainda, abordaram que se sentiram mais familiarizadas pois tiveram acesso à informação previamente.

[...] *se preparar para o parto é se preparar emocionalmente,* [...] *na verdade, a primeira etapa pro teu maternar é tu estar preparada pro teu parto.* (E22)

[...] *foi importante é que eu já estava familiarizada com tudo, elas já tinham me falado como que era, que a enfermeira vinha de tanto em tanto tempo, eu já conhecia a sala, eu já sabia o que ia acontecer ali sabe* [...]. (E2)

Quando o ambiente em mudança estimula a pessoa a criar respostas adaptáveis um feedback positivo é emitido ao sistema permitindo que a mesma decida aumentar ou diminuir seus esforços para lidar com os estímulos.

[...] *eu não ia ler muita coisa porque como eu sou ansiosa, aí eu ficava pensando que isso talvez ia piorar minha situação.* [...] *Hoje eu faria completamente diferente,* [...] *eu já ia fazer tudo diferente, eu já ia conversar sobre o parto, planejar essas coisas* [...]. (E23)

Após vivenciarem esse processo, na visão das mulheres, o parto pode ser considerado um momento prazeroso e uma experiência positiva quando a mulher se sente preparada e amparada.

[...] *o parto normal pode ser prazeroso* [...]*. De toda minha maternidade a experiência mais legal que eu tive foi o parto, eu tenho uma lembrança muito boa sabe* [...]. (E19)

[...] *tem que ter esse apoio de alguém pra lembrar ela que no momento de maior desespero ela vai conseguir,* [...] *é uma onda que ela vem intensamente mas ela passa, mas saber isso e ter esse preparo antes é fundamental* [...]*, não é instintivo* [...]. (E20)

## DISCUSSÃO

A Covid-19 alterou a organização dos serviços de assistência materna, o que ocasionou dificuldades no acompanhamento pré-natal, parto e puerpério^(15)^. O cenário pandêmico submeteu as gestantes e puérperas a restrições e agentes estressores que impulsionaram uma necessidade de adaptação. Todas as pessoas são adaptáveis e reagem a estímulos internos e externos e utilizam mecanismos de enfrentamento para responder às alterações do ambiente. Logo, a interação desse ambiente em constante modificação com o indivíduo e a coletividade estimulou respostas adaptativas^(1,8,15)^.

A gravidez se configura como um período de intensas transformações que desencadeiam vários sentimentos, desafios e conflitos internos. No entanto, as mulheres que vivenciaram a gestação e o parto no primeiro ano de pandemia não foram sensibilizadas somente pela gravidez, mas também por influências externas que interferem no processo de adaptação^(1,16)^. Diante dos relatos, a pandemia e a gestação emergem como estímulos focais por terem sido caracterizados como situações de forte impacto.

Os estímulos contextuais podem afetar o comportamento das mulheres, suas respostas frente aos desafios e ainda, a capacidade de adaptação ao ambiente. Nessa perspectiva, os comportamentos das mulheres podem influenciar sua autoestima e bem-estar, transformando também o ambiente^(8,10,16)^. As alterações fisiológicas intrínsecas ao período gestacional foram somadas às mudanças relacionadas às medidas de prevenção contra a Covid-19, produzindo um estresse adicional e interferindo no processo de adaptação^(8,17)^. Portanto, gestar no contexto de pandemia foi definido como um fator potencializador de medos, incertezas, inseguranças, estresse e ansiedade^(18)^.

Entre as diversas culturas, observa-se que existem crenças específicas sobre o momento do parto por estar repleto de significados transmitidos ao longo das gerações^(19)^. Roy considera estes aspectos como estímulos residuais constituídos de peculiaridades presentes nas pessoas, cujos impactos causados nos indivíduos são indefinidos^(8)^. Portanto, conforme constatado, experiências anteriores ou situações desconhecidas atribuem relevância no enfrentamento do parto.

Ao considerar que um mesmo estímulo pode desencadear comportamentos diferenciados nos indivíduos, a partir de seu nível individual de adaptação, a teoria de Roy permite reconhecer que pessoas que enfrentam situações desafiadoras podem despertar respostas, ora adaptativas, ora ineficientes^(20)^. No entanto, os estímulos ambientais que as mulheres receberam inicialmente provocaram exacerbação das respostas negativas, partindo disso, potencializaram seus esforços para a preparação para o parto, como um mecanismo adaptativo.

A capacidade de enfrentamento de uma pessoa é proporcional ao nível de adaptação e ao momento da vida em que se encontra. Alguns mecanismos de enfrentamento são heranças genéticas, já outros são aprendidos. Tais mecanismos são expressos por meio de comportamentos, caracterizados como modos adaptativos, que se dividem em quatro categorias: fisiológico; autoconceito; desempenho de papel e interdependência^(8,21)^.

Mulheres que recebem suporte contínuo durante o período perinatal são mais propensas a desenvolver efeitos psicológicos positivos e, consequentemente, respostas adaptativas^(22)^. No decorrer do pré-natal, a mulher grávida é instruída acerca da gestação e do parto. Ao levar em consideração seus próprios valores e desejos, formula preferências e toma decisões informadas em relação às práticas obstétricas a serem seguidas ou evitadas durante o seu parto. Esse processo de educação em saúde não apenas contribui para fortalecer a segurança, mas também fomenta a promoção da autonomia das mulheres, capacitando-as a desempenharem um papel central no seu processo de parto, exercendo o direito de tomada de decisões de maneira esclarecida^(23)^. Assim, diante de um cenário repleto de incertezas, o grupo de gestantes emerge como um facilitador essencial no mecanismo de adaptação, criando uma rede de suporte que contribui significativamente para o fortalecimento emocional e a confiança das mulheres durante o período pré-natal.

Uma maior conscientização sobre o períneo e técnicas de fortalecimento do assoalho pélvico desde a gestação constituem estratégias de proteção da integridade da região e aumento da percepção da via de parto^(24)^. Quanto ao modo fisiológico, as práticas terapêuticas utilizadas na gestação e no parto foram associadas ao aumento do conforto e autonomia da mulher no processo de parturição.

A gestação envolve um processo de reestruturação e reajustamento em várias dimensões, mudança de identidade e de papéis sociais, proporcionando novos níveis de integração e amadurecimento da própria personalidade. Todas essas transformações ocorrem com o casal e particularmente com a gestante, se intensificam pelas diversas alterações fisiológicas presentes, demonstrando a importância do preparo para o parto como estímulo à produção de comportamentos facilitadores de seus papéis parentais^(24-25)^.

As mudanças que ocorrem no autoconceito da mulher auxiliam no seu preparo para a maternidade e para o desenvolvimento de novos papéis. Portanto, o reconhecimento das mudanças corporais inerentes ao progresso da gestação envolve a imagem corporal e a maneira como a mulher se reconhece fisicamente, definido como um dos componentes do autoconceito descrito por Roy^(8,26)^.

A gravidez é um processo fisiológico planejado e desejado por muitas mulheres, no qual se criam expectativas. A pandemia e, consequentemente, o distanciamento social geraram mudanças comportamentais significativas^(3,16)^. Neste grupo de mulheres, foi possível identificar um sentimento de frustração, especialmente pelo desejo não concretizado de compartilhar o crescimento da barriga com pessoas de seu ciclo de relacionamento, ou ainda de não estarem realizando atividades previamente planejadas para este momento. O suporte familiar, especialmente do genitor e dos pais da gestante, é um pilar importante na sustentação materna. Uma rede de apoio fortalecida é primordial para uma adaptação efetiva^(25)^.

A oportunidade de dividir e trocar experiências, ser ouvida, sanar dúvidas e compartilhar sentimentos, mesmo em um contexto de restrição de presença física, ou seja, no contexto online, faz com que a gestante se sinta mais acolhida e confiante para o parto^(27-28)^. Em vista disso, a reciprocidade das relações interpessoais construídas no grupo de gestantes, caracterizado no modo de interdependência como um sistema de apoio, foi fundamental para o processo adaptativo dessas mulheres.

Entende-se que a gravidez é um fenômeno biopsicossocial que ocasiona modificações na vida da mulher^(29)^ e, diante disso, a adaptação ao papel materno pode ser um desafio para muitas mulheres, especialmente quando há carência de informações e apoio^(30)^. Nessa direção, o grupo de gestantes foi definido como um espaço tranquilizador dessas angústias por meio da disseminação de informações confiáveis, facilitando essa adaptação à maternidade.

A participação efetiva do acompanhante na gestação, além de demonstrar suporte e fortalecer o processo de desempenho de papel, repercute na satisfação da mulher com o apoio recebido durante o trabalho de parto e na sua segurança para desempenhar as ações planejadas^(31)^. Assim, o envolvimento dos acompanhantes com o grupo de gestantes teve um destaque positivo na visão das mulheres, contribuindo na construção de um parto saudável e respeitoso.

O processo de preparação para o parto envolve a incorporação de um conjunto de cuidados, medidas e atividades que possibilitem que a mulher experimente o trabalho de parto e parto como um evento fisiológico de modo a apropriar-se deste momento^(24,32)^. Dessa forma, o acompanhamento pré-natal, mediante iniciativas preventivas e ações de educação em saúde, visa assegurar o desenvolvimento saudável da gestação e facilitar o nascimento de um bebê saudável, preservando a integridade materno-infantil^(33)^.

Por meio de informações e orientações é possível enfrentar esse período com mais segurança e tranquilidade, em contrapartida, essa falta pode comprometer sua participação ativa e provocar uma visão deturpada do parto^(34)^. Nesse sentido, as mulheres que avaliaram sua preparação como insuficiente utilizaram esta experiência como um aprendizado e, a partir dessa percepção, se viam mais dispostas a participar ativamente dos cuidados com sua saúde.

Callista Roy aborda que o uso máximo de mecanismos de enfrentamento potencializa o nível de adaptação da pessoa e eleva sua capacidade de expressar respostas adaptativas frente aos estímulos. Nesse sentido, as respostas adaptativas desenvolvidas no preparo para o parto transformaram-se em um feedback positivo ao sistema, potencializando seus níveis individuais de adaptação e ampliando o alcance de estímulos aos quais possam responder positivamente com simples esforços^(8,10)^.

### Limitações do estudo

Um dos aspectos limitantes deste estudo está na natureza e abrangência da amostra utilizada. A coleta de dados foi restrita a um número específico de participantes de grupos de gestantes de uma única região, o que pode limitar a generalização dos resultados para uma população mais ampla e diversificada. Além disso, a participação voluntária pode ter introduzido um viés de seleção, pois é possível que mulheres que se sentiram mais engajadas ou beneficiadas pelo projeto tenham sido mais propensas a participar do estudo. Em estudos futuros, recomenda-se explorar a visão paterna e dos profissionais de saúde que acompanharam esse processo de adaptação. Tal integração tem o potencial de compreender a complexidade desse fenômeno e aprofundar os achados em relação à temática.

### Contribuições para a área da enfermagem e saúde

Este estudo oferece contribuições valiosas para a área da enfermagem e saúde, enfatizando a importância de manter as mulheres bem informadas para que participem ativamente de seu processo de parturição. Destaca-se a relevância da inclusão das gestantes em atividades que promovam respostas adaptativas durante o período gravídico, especialmente aquelas de natureza educativa e coletiva. Estas atividades não apenas fortalecem o conhecimento e a autonomia das mulheres, mas também incentivam a adoção de práticas saudáveis e proativas em relação à maternidade.

Ademais, espera-se que os achados deste estudo sensibilizem os profissionais da saúde, particularmente os enfermeiros, sobre a importância de criar espaços que favoreçam a interação e a aprendizagem entre as famílias. Tais espaços são essenciais para facilitar a adaptação das famílias ao ambiente de parto e puerpério, promovendo uma experiência mais positiva e integrada. Através destas iniciativas, é possível melhorar significativamente o suporte e a orientação oferecidos às gestantes, contribuindo para a saúde e bem-estar tanto das mães quanto dos recém-nascidos.

## CONSIDERAÇÕES FINAIS

Nosso estudo destaca que os modos adaptativos propostos por Roy são inter-relacionados, ou seja, a aprendizagem, percepção e processamento de informações, julgamento e emoções estiveram atreladas a esse processo de adaptação ao preparo para o parto. Logo, coloca em evidência a importância de um ambiente que estimule o uso máximo de mecanismos de enfrentamento.

O grupo de gestantes foi caracterizado como um ambiente capaz de promover respostas adaptativas, sobretudo na construção de um sistema de apoio efetivo não apenas entre profissionais e participantes, mas também entre as próprias mulheres. Todavia, é possível considerar que a participação em atividades educativas desde a gestação configura uma atitude importante para o aumento da satisfação no processo de parturição.

## DISPONIBILIDADE DE DADOS E MATERIAL

https://doi.org/10.48331/scielodata.Q5KQCH
